# HIV-1 Packaging Visualised by In-Gel SHAPE

**DOI:** 10.3390/v13122389

**Published:** 2021-11-29

**Authors:** Aaron R. D’Souza, Dhivya Jayaraman, Ziqi Long, Jingwei Zeng, Liam J. Prestwood, Charlene Chan, Dennis Kappei, Andrew M. L. Lever, Julia C. Kenyon

**Affiliations:** 1 Department of Medicine, Yong Loo Lin School of Medicine, National University of Singapore, Singapore 119228, Singapore; aaron.dsouza017@gmail.com (A.R.D.); dhivyajayaraman@gmail.com (D.J.); 2 Cancer Science Institute of Singapore, National University of Singapore, Singapore 117599, Singapore; charlenechan@nus.edu.sg (C.C.); dennis.kappei@nus.edu.sg (D.K.); 3 Department of Medicine, University of Cambridge, Level 5 Addenbrookes Hospital, Cambridge CB2 0QQ, UK; longziqi1011@gmail.com (Z.L.); jingwei.zeng@nhs.net (J.Z.); lp15@sanger.ac.uk (L.J.P.); 4 Department of Biochemistry, Yong Loo Lin School of Medicine, National University of Singapore, Singapore 117596, Singapore; 5 NUS Center for Cancer Research, Yong Loo Lin School of Medicine, National University of Singapore, Singapore; 6 Department of Microbiology and Immunology, Yong Loo Lin School of Medicine, National University of Singapore, Singapore 117545, Singapore; 7 Homerton College, University of Cambridge, Cambridge CB2 8PH, UK

**Keywords:** HIV-1, packaging, in-gel SHAPE, Gag, NC, RNA structure, dimerisation

## Abstract

HIV-1 packages two copies of its gRNA into virions via an interaction with the viral structural protein Gag. Both copies and their native RNA structure are essential for virion infectivity. The precise stepwise nature of the packaging process has not been resolved. This is largely due to a prior lack of structural techniques that follow RNA structural changes within an RNA–protein complex. Here, we apply the in-gel SHAPE (selective 2’OH acylation analysed by primer extension) technique to study the initiation of HIV-1 packaging, examining the interaction between the packaging signal RNA and the Gag polyprotein, and compare it with that of the NC domain of Gag alone. Our results imply interactions between Gag and monomeric packaging signal RNA in switching the RNA conformation into a dimerisation-competent structure, and show that the Gag–dimer complex then continues to stabilise. These data provide a novel insight into how HIV-1 regulates the translation and packaging of its genome.

## 1. Introduction

The HIV-1 genome (gRNA) is a single-stranded RNA molecule that encodes the essential structural polyproteins Gag, Gag-Pol and the envelope glycoprotein Env, together with a number of accessory factors that aid viral replication and immune evasion. gRNA thus serves as a template for the translation of the viral structural proteins found in Gag and the enzymes encoded by Pol, as well as being captured by Gag for packaging into virions. During packaging, the gRNA undergoes dimerisation, resulting in two copies of the genome being encapsidated into the budding virions. During or after budding, the Gag and Gag-Pol polyproteins are cleaved into their individual components—matrix (MA), capsid (CA) and nucleocapsid (NC)—as well as three smaller peptides [1], and the enzymes reverse transcriptase (RT), integrase (IN) and protease (PR) by PR itself. Upon infection of a new cell, RT initiates the reverse transcription of the ssRNA genome into dsDNA using a cellular tRNA^Lys3^ primer that anneals to the gRNA at some stage during viral assembly and budding. The chaperone activity of NC facilitates the annealing of tRNA to the primer binding site (PBS), and aids reverse transcription by destabilising the secondary structures that would cause the pausing or stalling of the enzyme [2,3,4,5,6,7,8]. IN then integrates the freshly synthesised proviral DNA into the newly infected host cell genome, from where it can be transcribed.

**Figure 1:**
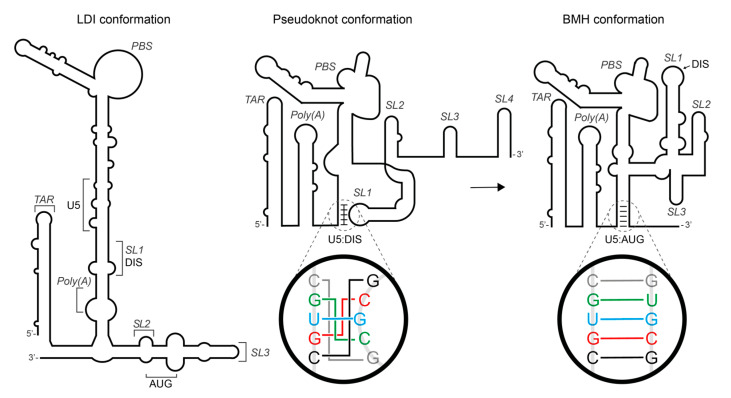
Schematic representation of two of the HIV RNA structural shift models, from the ‘Long Distance Interaction’ (LDI) conformation, or ‘pseudoknot’, containing SL4, to the ‘Branched Multiple Hairpin’ (BMH) conformation, containing the U5:AUG helix. The coloured nucleotides in the magnified circle indicate the base-paired nucleotides in the U5:DIS and U5:AUG interactions. Brackets shown on the LDI structure represent the loop region sequences for stem–loops and the two halves of the U5:AUG interaction for U5 and AUG.

A critical step in this complex viral replication cycle is the recognition and packaging of the viral gRNA. The gRNA packaging process is highly specific and represents a novel drug target [9]. It has proven hard to study in structural detail due to the transient nature of the sequential steps involved, likely involving multiple different RNA structures. The recognition of the gRNA by Gag is dependent upon sites within the highly conserved 5′ UTR [10,11,12,13]. This region consists of conserved hairpin/helical structures, including the trans-activation response element (TAR), a poly(A) sequence, the tRNA primer binding site (PBS) and the major packaging signal (Ψ) [14,15] (Figure 1). Ψ is a vital component of the dimerisation process, and is composed of three stem–loops (SL1–SL3). SL1 contains a palindromic dimer initiation site (DIS) and facilitates RNA dimerisation through an intermolecular kissing-loop interaction [16,17,18,19,20,21,22], SL2 contains the splice donor (SD) site and SL3 is a major determinant of gRNA encapsidation [23,24,25]. An additional stem–loop that spans the Gag start codon, SL4, has been proposed to regulate Gag translation by preventing interaction between the U5 region and the *gag* initiation codon by forming the U5:AUG helix [15,26,27,28].

It has been previously proposed that the shift from the translation of *gag* to gRNA dimerisation is facilitated by an RNA structural switch. There have been two predominant models for this. Firstly, a switch from a ‘Long Distance Interaction’ (LDI) conformation to a ‘Branched Multiple Hairpin’ (BMH) [16,27,29]. In the LDI conformation, the DIS is prevented from forming the kissing-loop interaction by being base-paired with the poly(A) element, resulting in the *gag* initiation codon being located within a less stable structure than within the BMH conformation, to facilitate translation. In the BMH conformation, the *gag* initiation codon is sequestered through base pairing with the U5 region (referred to as the U5:AUG interaction), releasing the DIS and allowing it to base pair with the DIS on a second gRNA [16,27,29,30]. Subsequent work has broadly confirmed the BMH model; however, mutants created to prevent the formation of the LDI conformation led to reduced dimerisation, but did not impact Gag translation [30]. More recent approaches using NMR and in-gel SHAPE suggest an alternative pseudoknot structure for the monomer [15,31]. In this structure, the DIS binds to a complementary site in the U5 region and SL4 forms; dimerisation then accompanies a switch from U5:DIS to U5:AUG (Figure 1). The precise transcriptional start site and the number of 5′ Gs the transcript contains has also been proposed to control RNA structural changes [32,33,34].

The initiation of gRNA encapsidation is generally accepted to involve a small number of Gag proteins binding to Ψ [35]. The switch to the U5:AUG interaction from U5 being in an alternative intramolecular pairing frees the DIS sequence for intermolecular base pairing via a kissing-loop interaction and the formation of ‘loose dimers’ [15,17,21,29,36,37,38,39]. In association with the NC domain of the Gag protein, the RNA molecules refold to form a more stable ‘tight dimer’ or ‘extended duplex’ [40,41,42,43], the intermolecular extent of which may extend significantly beyond SL1 itself [44]. The ribonucleoprotein complex containing a small number of Gag proteins and the gRNA traffics to the plasma membrane where additional, exposed binding sites in the gRNA allow the recruitment of further Gag proteins to form the immature viral particle [42,45,46,47,48].

We previously demonstrated an in-gel SHAPE (selective 2’OH acylation analysed by primer extension) method that was able to resolve the structures of the monomeric and dimeric HIV-1 leader sequences, without the need for stabilising mutagenesis, that identified certain key structural changes involved in RNA dimerisation [31]. SHAPE reagents such as NMIA (N-methyl isatoic anhydride) covalently react with the 2’OH of nucleotides irrespective of base, directly proportionally to the flexibility of the nucleotide backbone at that position. They therefore act as a marker of whether a nucleotide is single-stranded or base-paired. SHAPE data are used in conjunction with modelling software to derive a secondary structural model for the structure or range of structures (‘ensemble’) of the RNA. Using these reagents in a native gel matrix enables the separation and isolation of individual RNA conformers. Our previous use of this technique under native conditions demonstrated differences in NMIA reactivity within the U5, AUG and SL1 sequences that marginally favoured the pseudoknot model of the monomeric structure over other models [15].

Here, initial experiments on the well-established TAR–Tat interaction suggested that SHAPE reagents reliably report upon the structural flexibility of the backbone at each nucleotide without being strongly affected by the ‘footprint’ of the protein binding. However, the structural ensemble of monomeric TAR RNA in the absence of Tat differs from the structural ensemble of the unshifted TAR that was incubated in the presence of Tat. Effectively, the technique appears to reveal the sub population of structures within the ensemble to which the protein did not bind.

We then used in-gel SHAPE to study the 5′ region of the HIV-1 gRNA from the transcription start to within the beginning of the Gag open reading frame that contains the major sequences required for gRNA encapsidation. We sought to identify changes that occur in the structural ensembles of the HIV-1 RNA monomer and dimer RNA species upon the addition of Gag or NC during the gRNA dimerisation process.

We found that the monomeric ensemble in the absence of a ligand, as well as the structures with Gag bound, largely resemble the LDI model, with the DIS paired in a long-range interaction with the U5 region. The monomeric ensemble with NC bound was more heterogeneous but still contained many of the LDI features. Within the dimeric ensemble TAR, poly(A) and SL1 structures were frequently present, but the dimer in the absence of a ligand did not contain the U5:AUG helix; however, the shifted dimer did. Our results show the surprising diversity of the RNA structural ensembles that could potentially be formed, and how they differ when Gag or NC bind to the RNA. They also indicate the structures preferentially selected by Gag or NC, as well as how the proteins remodel the RNA.

XL-SHAPE was able to identify the initial interaction sites of Gag with the gRNA and show that these differ from those of NC at the same molar ratio of protein:RNA. Gag first interacts with the TAR region, and in doing so has structural effects on the downstream Ψ region structure. The interaction with NC alone is more promiscuous and is more reflective of how Gag interacts with the RNA when Gag is in higher concentrations. Our results suggest a mechanism by which HIV controls the switch between translating and packaging its genome, and provide insights into the RNA structures occurring during viral maturation. Interference with these critical structural transitions may have therapeutic potential.

## 2. Materials and Methods

### 2.1. RNA Preparation

RNA was transcribed in vitro from DNA templates encoding the HIV-1 genome and containing a T7 RNA polymerase promoter at the 5′ end. DNA templates were synthesised by PCR with 1× BioMix red (Bioline, Cincinnati, OH, USA), 800 ng of plasmid DNA (pSVC21) and 30 pmol of forward (5′-TAATACGACTCACTATAGGGTCTCTCTGGTTAGACCAGATCTG-3′) and reverse (5′-CTTTCCCCCTGGCCTTAACC-3′) primers, TAR forward, 5′TAATACGACTCACTATAGGCCTTCGGGC CAAGGTCTCTCTGGTTAGACC-3′; TAR reverse, 5′CACT ACTTGAAGCACTCAAGG-3′ using plasmid pSVC21 as a template, as per Kenyon et al. (2013).

PCR products were purified by gel extraction (QIAQuick, Qiagen, Hilden, Germany) according to the manufacturer’s instructions. In vitro RNA was transcribed using the MEGAscript T7 (Life technologies, Carlsbad, CA, USA). Each 20 μL transcription reaction contained 7.5 mM ribonucleotide triphosphates (NTPs), 1× reaction buffer, 800 ng of PCR products and 2 μL of T7 RNA polymerase, and was incubated at 37 °C for 3 h. DNA was degraded with 6 U of DNase (TURBODNase, Life technologies) per 20 μL reaction at 37 °C for 90 min (5′UTR-*gag* RNA), or with 3 U of DNase per 20 μL reaction at 37 °C for 45 min (TAR RNA). RNAs were purified with MEGAclear columns (Life technologies), eluted in RNase-free water and stored at −20 °C.

### 2.2. Protein Expression and Purification

Tat peptide was obtained as described in [49]. Gag Δp6 (herein referred to as Gag) was expressed in bacteria and purified as previously described, by FPLC on AKTA and GST affinity chromatography using GSTrap FF 5 mL columns (GE Healthcare, Chicago, IL, USA). The GST was removed with PreScission protease (GE Healthcare) [49]. Chemically synthesised NC was a kind gift from Rob Gorelick.

### 2.3. In-Gel SHAPE

When refolding 5-’UTR RNA, RNA (24 µg) was resuspended in 160 μL of renaturation buffer (10 mM Tris-HCl, pH 8; 100 mM KCl and 0.1 mM EDTA), heated at 85 °C for 5 min and slowly cooled inside the metal tube-holder insert by removing it from the heat block onto the bench, until it reached 29 °C. The volume was adjusted to 200 μL and a final concentration of 40 mM Tris-HCl, pH 8; 4 mM MgCl_2_; and 130 mM KCl, followed by incubation at 37 °C for 30 min.

TAR RNA (24 µg) was refolded by being resuspended in 80 μL of renaturation buffer, and being heated and slowly cooled as above. The volume was adjusted to 100 μL with a final concentration of 40 mM Tris-HCl, pH 8; 130 mM KCl; 4 mM MgCl_2_; 0.2 mM EDTA, followed by incubation at 37 °C for 30 min.

For RNA with NC samples, 15× molar excess of NC (corresponding to 1NC:27.6nt) was incubated with the RNA for 15 min at 20 °C along with 4U/μL ribonuclease inhibitor (RNasin, Promega, Madison, WI, USA) and 100 × excess of tRNA (*w*/*w*) in a binding buffer containing 65 mM Tris-HCl (pH 8), 26 mM KCl, 6.5 mM dithiothreitol (DTT), 0.13% Triton X-100 and 0.13 mM ZnCl_2_. RNA controls in the absence of protein were incubated with RNasin, tRNA and a binding buffer.

For RNA with Gag experiments, RNA and protein (15× Gag/1 Gag:27.6nt) were incubated as above, with the exception that Gag was stored and added to the RNA in a different buffer (protein elution buffer, 0.5–5 μL volume per sample, 142 mM NaCl, 12.5 mM Tris-HCl, 0.25 mM EDTA, 675 μM KCl, 2.5 mM Na_2_HPO_4_, 450 μM K_2_HPO_4_, 1 mM DTT)), hence the RNA-only controls were also incubated with an equal volume of this buffer.

Samples were mixed with native loading dye at a final concentration of 4% glycerol (*v*/*v*), an additional 7.33 mM Tris-borate, pH 7 and 0.04% orange G dye. RNAs and RNA–protein complexes were then separated by native polyacrylamide gel electrophoresis using gels prepared with 4% acrylamide and 1× Tris-borate magnesium (TBM) (89 mM Tris base, 89 mM boric acid and 0.1 mM MgCl_2_). Samples were electrophoresed at 120 V for 15 min followed by 4 h at 110 V. The first three lanes of the gel were excised, containing an RNA ladder (RNA Century Plus, Ambion, Austin, TX, USA), one RNA sample and one RNA with viral protein sample. The gel fragment was then stained with 1.3 μM of ethidium bromide in 1× TBM for 5 min and visualised. The stained gel piece was aligned with the rest of the unstained gel, and specific bands (e.g., monomeric RNA without protein bound/monomeric RNA with protein bound) were excised with a scalpel. Each excised gel piece was then divided equally into two pieces. One piece of each band was incubated with 10% DMSO in 1× TBM (3 mL), and the other piece was incubated with 10% 100 mM NMIA (in DMSO) in 1× TBM (3 mL) at 20 °C for 45 min. Gel pieces were then washed twice with 1× TBM and soaked in 3 mL of proteinase K buffer (600 μg proteinase K (Ambion); 50 mM Tris-HCl, pH 7.5; 100 mM NaCl; 1% sodium dodecyl sulfate (SDS); and 10 mM EDTA) at 55 °C for 1 h. After incubation, gel slices were diced into small pieces (maximum 1 mm^3^) and washed three times with 1× TBM, then once with 1× Tris-acetate EDTA (TAE, 40 mM Tris-acetate and 1 mM EDTA). Alternatively, during optimisation experiments, after gel pieces were diced, 1 mL of acetonitrile was added and centrifuged at 375× *g* at 10 °C for 10 min twice, and dried in a speed vac for 5 min. The gel pieces were rehydrated with 1 mL of 1× TAE. RNA was electroeluted using an Elutrap (Whatman, Maidstone, UK) overnight at 100 V and 4 °C. RNA was purified using phenol-chloroform extraction (extraction in phenol:chloroform 1:1, pH 5, then chloroform:isoamyl alcohol 24:1) followed by ethanol precipitation using 300 mM sodium acetate, pH 5.5 (final concentration), and 2.5 volumes of ice-cold ethanol.

### 2.4. Electrophoresis Mobility Shift Assay (EMSA)

RNA was in vitro transcribed using ^32^P-UTP and renatured in the same concentrations and conditions as for in-gel SHAPE. Incubation with varying amounts of Gag was done using the same total volume per lane as for in-gel SHAPE experiments, but a lower RNA concentration, of 0.35 pmoles (42 ng) per lane, and corresponding molar ratios of Gag. Samples were electrophoresed as described for in-gel SHAPE experiments above, gels were dried onto filter paper and visualised by autoradiography.

### 2.5. Reverse Transcription, Sequencing and Data Analysis

For in-gel SHAPE experiments, 200 ng of RNA of both the DMSO control (−) and NMIA-treated (+) samples was resuspended in 12 µL of 2.1 mM Tris-HCl (pH 8.0) and 42 μM EDTA. VIC-labelled (Applied Biosystems) and 6FAMTM-labelled (Applied Biosystems, Waltham, MA, USA) fluorescent primers were added to the (−) and (+) samples, respectively, with a final concentration of 5 nM. Primers were annealed at 85 °C for 1 min, 60 °C for 5 min and 35 °C for 5 min. Primer extension was initiated by the addition of 5 mM DTT, 0.5 mM dATP, dCTP, dUTP and 7-deaza-dGTP, 40 U of SuperScript III reverse transcriptase (Invitrogen, Waltham, MA, USA) and 1× SSIII buffer to each sample. Extension was continued at 55 °C for 60 min. The cDNAs of (−) and (+) reactions were combined and RNA was degraded using a final concentration of 200 mM sodium hydroxide (NaOH), followed by incubation at 95 °C for 3 min. This was cooled on ice and the NaOH was neutralised with a final concentration of 200 mM hydrochloric acid. Sequencing ladders were generated using the Thermo Sequenase Cycle Sequencing Kit (Thermo Fisher Scientific, Waltham, MA, USA) and primers with the same sequences as those used in reverse transcriptions, but labelled instead with NED^TM^ or PET^®^ (Applied Biosystems, Waltham, MA, USA).

The cDNAs and sequencing ladders were precipitated with sodium acetate and ethanol, resuspended in water and initially titrated individually onto sequencing plates to verify an amount at which maximum signal intensity and bleed-through into other channels did not occur, then combined at this amount and separated on a 3730xl DNA Analyser (Applied Biosystems). The sequences were aligned using SHAPEfinder, and mobility shift controls were performed as described in [50]. Differences in fluorophore signal means that traces are scaled in SHAPEfinder relative to one another such that the baselines overlap as much as possible; this leads to some negative reactivity values. Data were normalised and analysed as described in [31], briefly by dividing each NMIA peak area—control peak area value by the average of the top 8% of values, below the datapoint that represented the third quartile plus 1.5 times the interquartile range. Following this, to remove outliers that might otherwise skew the average, where experiments contained *n* >/= 4 all replicates were aligned in Excel and outliers at each nucleotide position were defined as datapoints that were greater than the third quartile value plus 1.5 times the interquartile range, or less than the first quartile value minus 1.5 times the interquartile range. Average nucleotide reactivity data were then used in RNA structure to generate models of the 20 lowest free-energy structures. To examine and visualise the variety of structures (‘ensemble’) the RNA forms under each condition, each group of 20 structural models was assessed for the structures adopted by nts-1–57 (TAR), 58–104 (poly(A)), 105–115 (the 5′ side of U5:AUG), 236–282 (SL1) and 283–343 (SL1–3 and the 3′ side of U5:AUG). Firstly, the number of different structures for each structural element was counted. Minor variations which differed by up to two structural elements (such as an individual helix or loop) were classified as variants of that structure, with the exceptions of smaller modifications at the base of TAR, poly(A) and U5:AUG, which were classified and counted as separate structures, as minor variations in TAR and poly(A) have been suggested to be of biological importance and U5-AUG contains only one structural element. For each of these structural elements, the proportion of the ensemble present in each different classified structure/variant was scored out of 20.

### 2.6. Cross-Linking

RNA was renatured and incubated with/without protein as above, with the inclusion of an additional sample using aprotinin as an RNase-free negative control protein of similar charge. Samples were aliquoted onto two 96-well round-bottomed plates: one was cross-linked for 2 min at standard power on ice in a stratalinker (XL-1500) UV cross-linker and the other was incubated for 2 min on ice without cross-linking. Proteinase K (1.5 µL of 20 mg/mL) and 3.75 μL of 20% SDS were added to each sample, followed by incubation at 55 °C for 60 min. RNA was purified by phenol-chloroform extraction and ethanol precipitation as above.

RNA was then reverse-transcribed as above. However, the combinations of primers used was different (as described in [49]). Firstly, the fluorophores themselves can affect the pausing pattern of the reverse transcriptase very slightly, which becomes pertinent because UV cross-linking at 254 nm is inefficient (in contrast to acylation using NMIA). The cross-linking signals we detect are therefore small. In order to account for this ‘background’ caused by the fluorophores themselves, the cross-linking experiment included extra RNA-only samples (with or without UV treatment) to be used as fluorophore controls, which were reverse-transcribed with primers labelled with either 6FAM^TM^ or VIC^®^, before being combined (6FAM- and VIC-labelled cDNAs made from RNAs that had been UV-treated were combined, and 6FAM- and VIC-labelled cDNAs made from RNAs that had not been UV-treated were combined) for capillary fractionation. The average of this background was then subtracted from the following experiments: RNA-only samples with/without cross-linking were labelled with VIC, and RNA–protein samples with/without cross-linking were labelled with 6FAM. For capillary fractionation, a 6FAM-labelled cross-linked RNA–protein sample was combined with a VIC-labelled cross-linked RNA-only sample, and a 6FAM-labelled non-cross-linked RNA–protein sample was combined with a VIC-labelled non-cross-linked RNA-only sample. The capillary fractionation gave the differences in the amounts of 6FAM and VIC cDNAs of each nucleotide length, the corresponding fluorophore control background described above was taken away from this. Following this background subtraction, the non-cross-linked RNA–protein reactivity (controlling for RNA–RNA cross-links) was subtracted from the cross-linked RNA–protein reactivity to give a final cross-linking value for each nucleotide. As described and calibrated in [49], true sites of specific RNA–protein interaction were defined as nucleotides in the top 20% of reactivity values, whose reactivity was statistically significantly different from the nonspecific control protein, aprotinin (*p* < 0.05 by *t*-test).

### 2.7. Statistical Analysis

In instances where a difference from a control is calculated, a two-tailed *t*-test was performed, assuming that the two samples have unequal variance.

## 3. Results

### 3.1. In-Gel SHAPE of an RNA–Protein Complex Accurately Reports upon Its RNA Structure

Native RNA–protein interactions often involve the binding of multiple copies of the same or different proteins to the RNA, making it especially hard to resolve the RNA structures of individual complexes from within such a heterogeneous mixture. SHAPE has been previously used to identify structural changes induced in TAR RNA upon binding by a Tat protein [49]. In these experiments it was noted that the SHAPE reagent used, NMIA, was not especially sensitive to the presence of a protein in the way that larger footprinting reagents, such as RNases, are. Instead, the NMIA-dependent acylation reflected the structural flexibility of the RNA backbone.

In-gel SHAPE has been previously used to identify RNA structural changes upon dimerisation of the HIV-1 viral RNA leader sequence [49]. Its ability to resolve the RNA structures of individual conformers under native conditions has proven helpful for investigating RNA structural changes, but it had not previously been validated on RNA–protein complexes. Therefore, firstly, the suitability of in-gel SHAPE to evaluate RNA structural changes caused by protein binding was investigated. As the site of Tat protein binding to TAR RNA and the associated RNA structural changes have been extensively documented, we used this interaction to validate the technique [49,51].

**Figure 2:**
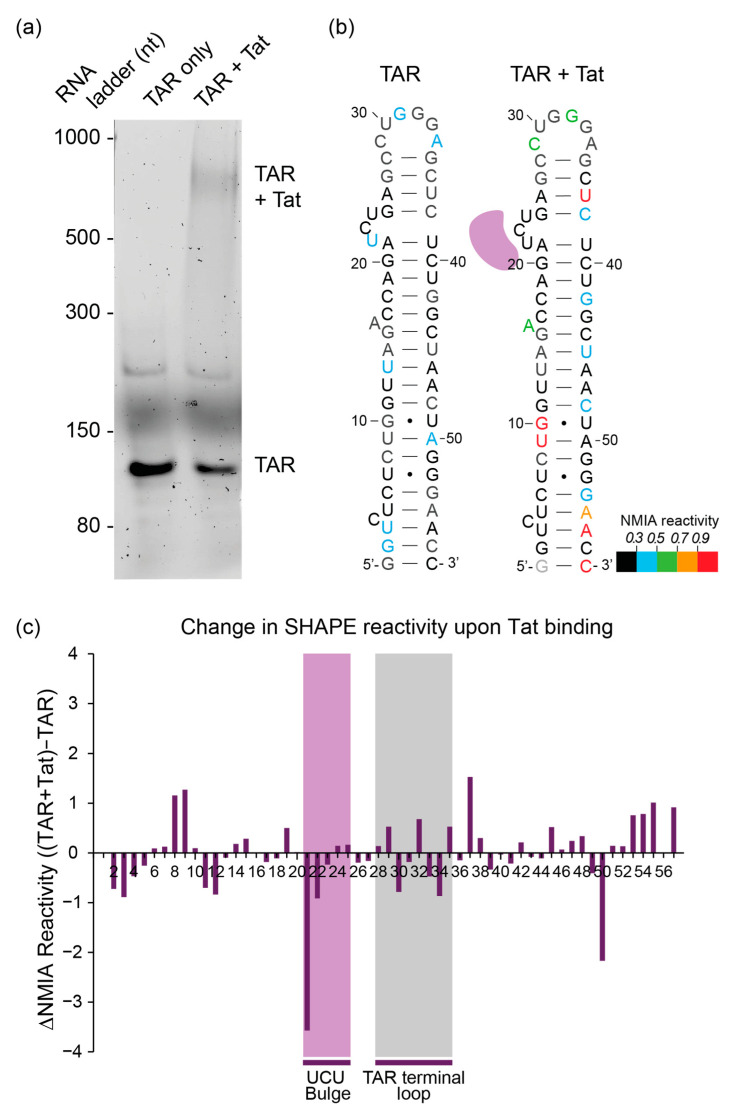
In-gel SHAPE validation using a well-described RNA–protein interaction. (**a**) Gel shift in the presence of a 1:6 molar ratio of TAR RNA to Tat protein. (**b**) Structural model of unshifted TAR and TAR with Tat bound (purple shape), based upon the NMIA reactivity data obtained from in-gel SHAPE. Standardised nucleotide reactivities (arbitrary units) are shown in colour according to the key. Black represents nucleotides with reactivity below 0.3 (rigid backbone, most likely to be base-paired) and red represents nucleotides with reactivity above 0.9 (flexible backbone, most likely to be single-stranded). TAR–Tat complex (*n* = 5) and TAR alone (*n* = 9). The poly(A) was also present in the RNA, but was used as the primer binding site; hence, no structural data for poly(A) were generated. (**c**) Change in average NMIA reactivity (arbitrary units) between the TAR–Tat complex (*n* = 5) and unshifted TAR (*n* = 9). Purple area represents nucleotides in the UCU bulge: A21-G25. Grey area represents nucleotides in the TAR terminal loop: C28-G35. N.B. black nucleotides in Figure 2b represent SHAPE reactivity values less than 0.3, many of which are negative values, leading to larger differences in reactivity than may be evident from the figure itself.

Refolded RNA containing the TAR and poly(A) sequences of HIV was incubated with a Tat peptide and complexes were separated by polyacrylamide gel electrophoresis (PAGE) using a native Tris-borate magnesium (TBM) gel (Figure 2a). Using an RNA ladder and a stained lane, the bands corresponding to the TAR alone and the TAR–Tat complexes in the unstained lanes were excised and probed in situ with NMIA. The RNA was then recovered, reverse-transcribed and analysed using our previously established in-gel SHAPE approach [31]. Because of the need to compare small differences in reactivity accurately, and the relatively high variance in SHAPE data between independent experiments, we examined six or more samples and took outliers out of the data (where an outlying nt was defined as above the third quartile plus 1.5 times the interquartile range, or below the first quartile minus 1.5 times the interquartile range, as shown previously [31]). This reduced some of the higher reactivities previously seen in in-gel experiments [31], but still recapitulated the TAR structure accurately upon modelling (Figure 2b and Supplementary Table S1). However, previous reactivity data from our 2013 paper [31] differed from the results herein, and we wondered whether the unshifted TAR band represented a different structural ensemble to an unperturbed TAR RNA population alone, with no Tat present. Unshifted TAR RNA would represent the RNA that had failed to bind its ligand. When we performed two new individual repeats of our 2013 experiments of TAR probed from within a gel in which Tat was not present, their reactivities fitted more closely with the TAR hairpin structure (Supplementary Figure S1), and statistically their reactivities were different from those of the unshifted TAR in the presence of Tat, with 15/56 or 12/56 nts in the two repeats being more than two standard deviations from the mean of the protein-exposed but unshifted population. Thus, here, the structural ensemble of an RNA that has not encountered its ligand differs from the structural ensemble of an RNA that has encountered the ligand but failed to bind it. Similarly to previous XL-SHAPE results, when Tat was bound, the SHAPE reagent showed a number of increases and decreases in RNA backbone flexibility across the TAR structure, commensurate with wide-ranging RNA structural changes effected by the binding of a protein to a specific site on the RNA [49] (Figure 2b,c). The sharp decrease in NMIA reactivity observed at A21 reflects the RNA structure at this position, as the A21:U39 base pair has previously been shown to be unstable/solvent-accessible in the absence of a ligand, and to pair stably only in the presence of a Tat peptide [51]. Thus, no clear ‘footprint’ that reports specifically on the interactions of the Tat protein with the RNA was observed at its binding site (A21-G25) under the conditions we used. Overall, the NMIA data suggest that in-gel SHAPE of an RNA–protein complex is sensitive to changes in RNA structure and flexibility induced by protein binding, but does not report specifically on the position of the protein itself upon the RNA.

### 3.2. Initial Contacts between Gag Protein and HIV-1 Leader RNA Shift the RNA Structural Equilibrium

**Figure 3:**
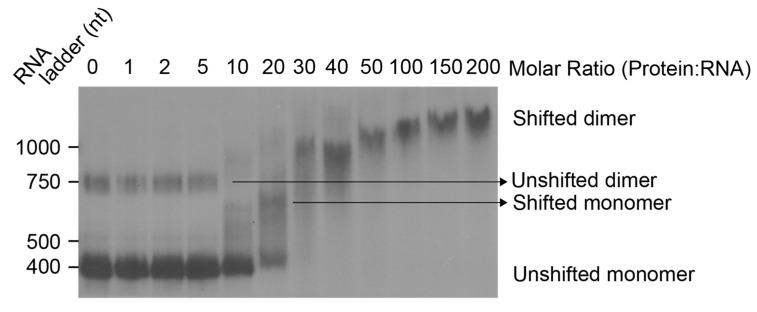
Electrophoretic mobility shift assay of Gag protein with in-vitro-transcribed viral 5′UTR RNA. ^32^P-labelled RNA corresponding to the first 411 nt of the genome was incubated with Gag protein at increasing molar ratios of protein to RNA and analysed on a native polyacrylamide gel (*n* = 4).

To identify the molar ratio at which initial contacts between Gag protein and the HIV packaging signal RNA are made in vitro, we performed an electrophoretic mobility shift assay (EMSA) (Figure 3). In the absence of Gag, an RNA dimer species is observed, but the majority of RNA exists in the monomeric conformation, previously suggested to be a pseudoknot by in-gel SHAPE [31].

At a molar ratio of between 10–20, we observe a shift in the RNA that is plausibly an intermediate conformation different from the original monomeric structure (such as the BMH structure) or the original monomeric structure (such as the LDI, pseudoknot or BMH) with Gag bound. At the lowest concentration of Gag that detectably binds to the RNA, both monomers and dimers are shifted. At a molar ratio of 30, there are no observable monomers. After this, Gag binding and the reduction in migration of the complex continues until Gag binding plateaus towards saturation. These results suggest that at low RNA concentration, initial Gag binding to the RNA induces a transition from a flexible structural ensemble of mainly monomers (with some dimers) to a more stable single-dimeric conformation via a transient intermediate monomeric conformation. Moreover, the striking mobility shift observed during successive Gag molecules binding between molar ratios of 20 and 30 suggests lower affinity binding of Gag to the initial RNA structures as a prelude to cooperative binding of Gag once the RNA converts to a more stable conformation. It must be noted that the experimental conditions used for the EMSA were optimised for a low concentration of radiolabelled RNA, and hence individual species from these gels could not be excised for in-gel SHAPE. This is because increasing the concentration of RNA to that required for in-gel SHAPE also increases the dimerisation of the RNA. In order to be able to investigate the initial effects of Gag binding upon both the monomeric and dimeric RNA, we chose a molar ratio intermediate between 10 and 20× Gag for in-gel SHAPE as it is the lowest molar ratio at which both the monomer and the dimer are reliably shifted (Supplementary Figure S2).

### 3.3. In-Gel SHAPE Suggests That Gag Remodels the Monomeric RNA Conformation but Stabilises the Dimer

In order to probe the conformational changes occurring within the RNA upon protein binding, 15× molar excess of Gag protein was incubated with folded in-vitro-transcribed 5′UTR RNA and resolved using native PAGE; monomeric or dimeric RNAs or RNA–protein complexes were isolated, probed and analysed, as for Figure 2. As this HIV-1 RNA is considerably larger than the TAR-poly(A) RNA examined in Figure 2, at 414 nucleotides for the monomer, recovery from the gel upon electroelution is lower, and due to the faint nature of the bands we could not retrieve enough unshifted monomer or dimer from the gel when we incubated the RNA with Gag/NC first, hence we performed separate experiments to look at RNA only and RNA bound to protein. This also had the advantage that in the RNA-only analysis the RNA population distribution had not been perturbed by prior interaction with a ligand, as we had shown for Tat-TAR.

**Figure 4:**
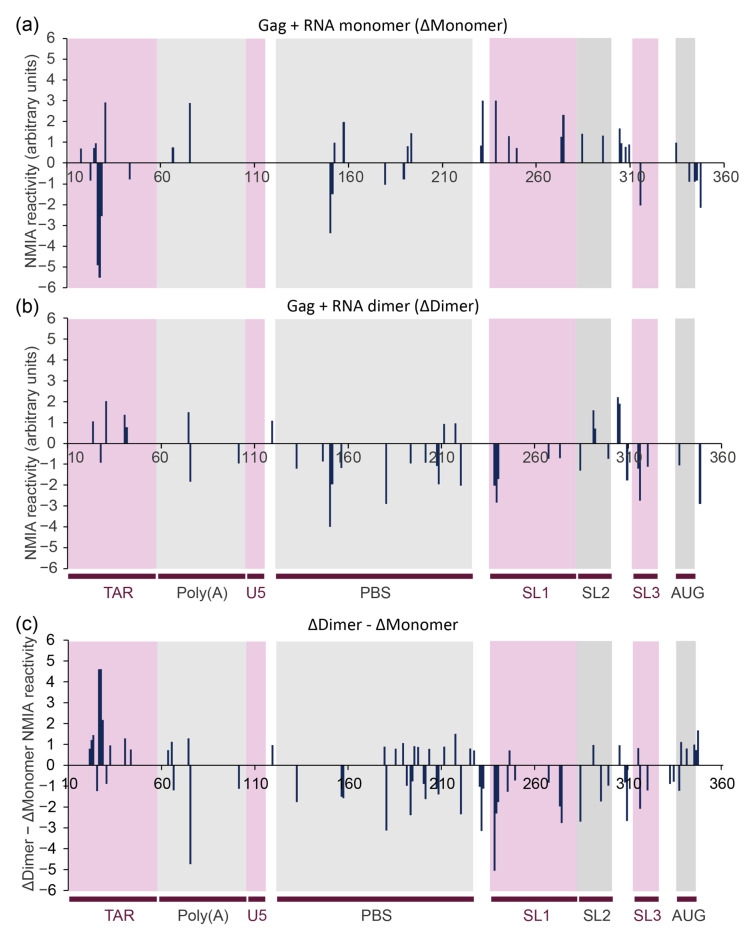
In-gel SHAPE results showing the site-specific influence of Gag on monomeric and dimeric HIV-1 RNA structures. Nucleotide numbering is shown on each graph along with the corresponding structural elements below graphs (**b**,**c**). (**a**,**b**) Greatest differences in NMIA reactivity upon Gag binding to RNA monomer (**a**) (monomer + Gag, *n* = 3; monomer only, *n* = 4) or dimer (**b**) (dimer + Gag, *n* = 3; dimer only, *n* = 5). Notable peaks in NMIA reactivity calculated by subtracting the NMIA reactivity of the RNA in the presence of Gag from the NMIA reactivity of the RNA in its absence (filtered for reactivity values >0.7 and <−0.7) are depicted. (**c**) Difference between changes to NMIA reactivity upon Gag binding to the monomer versus the dimer (filtered for reactivity values >0.7 and <−0.7).

The 414-nucleotide RNA design contained 3Gs at the transcriptional start site and 78 nts of *gag.* The difference in behaviour of the monomeric and dimer RNA upon Gag binding is shown in Figure 4. Increases in SHAPE reactivity reflect regions of the RNA that are adopting a more single-stranded structure, and/or regions of the RNA that are exposing themselves to the NMIA reagent during remodelling of the backbone into a different structure (as NMIA has a half-life of minutes in an aqueous solution, it detects regions of the RNA that are undergoing longer-term or continuous structural changes during which they transit through a single-stranded state). When analysing the shifted monomer population that has hence not dimerised even though Gag has bound, the binding of Gag protein has a predominantly destabilising effect, shown by the number of nucleotides with a substantial increase in NMIA reactivity, concordant with a remodelling of the RNA structure (Figure 4a). This remodelling is most pronounced across the 3′ end of the PBS to the 5′ end of SL3, suggesting that this region in particular differs between the structural ensemble of the ligand-naive and ligand-bound monomers.

### 3.4. NC and Gag Have Different Effects upon the RNA Structure, Commensurate with Their Roles in the Viral Lifecycle

**Figure 5:**
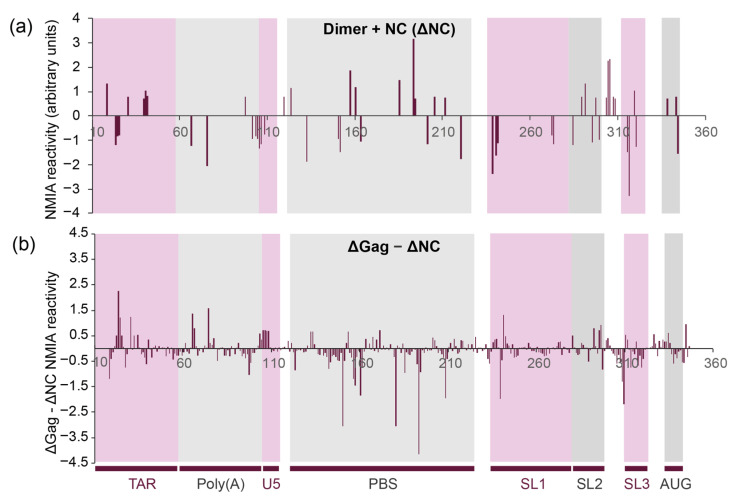
In-gel SHAPE measurement of the structural effects of NC upon binding to the dimeric HIV-1 RNA. (**a**,**b**) Larger structural effects of NC upon the dimeric HIV-1 RNA (>0.7 reactivity units). (**a**) NMIA reactivity changes in the dimeric structures induced by NC (NC + dimer, *n* = 4; dimer only, *n* = 5). (**b**) Difference between changes to NMIA reactivity in the dimeric structures caused by NC and Gag (dimer only, *n* = 5; NC + dimer, *n* = 4; Gag + dimer, *n* = 6).

Uncleaved Gag protein and its NC cleavage product serve different purposes during the formation and maturation of the viral particle as well as during the subsequent reverse transcription process. Thus, we compared the differences in RNA flexibility upon binding of uncleaved Gag protein and NC protein, as NC is only formed during viral maturation and is responsible for chaperoning later events, such as during reverse transcription. The binding of the same molar excess of NC to the RNA as Gag (15×) leads to higher NMIA reactivity in the dimer, and thus increased RNA flexibility in the TAR stem–loop, the PBS region, the SL2 stem–loop and the region between SL2 and SL3 (Figure 5a), suggesting that it is remodelling these regions, possibly to enhance reverse transcription. This is accompanied by a stabilisation of the 5′ sections of SL1 and SL3. Some of these structural effects appear to be affected by both NC and full-length Gag (comparison of Figure 4b and Figure 5a, also represented as the difference between NMIA reactivity change upon Gag or NC binding to the dimer in Figure 5b). These include the destabilisation of the TAR and SL2 stem–loops as well as the region between SL2 and SL3, and the stabilisation of SL3 and the 5′ section of SL1. However, there is a clear differential in the effect of Gag and the NC protein in their effect on RNA stability. The strongest difference is observed in the PBS region, where the binding of Gag causes many sites of RNA stabilisation relative to NC (Figure 5b), suggesting that Gag might stabilise the structure needed for tRNA to bind efficiently [52,53]. Indeed, the annealing of tRNA to the PBS has been proposed to be a two-step process, with differing roles for Gag and NC [54].

### 3.5. The Structural Ensembles of the RNA Change upon Dimerisation or Ligand Binding, and Differ between NC-Bound and Gag-Bound RNA

**Figure 6:**
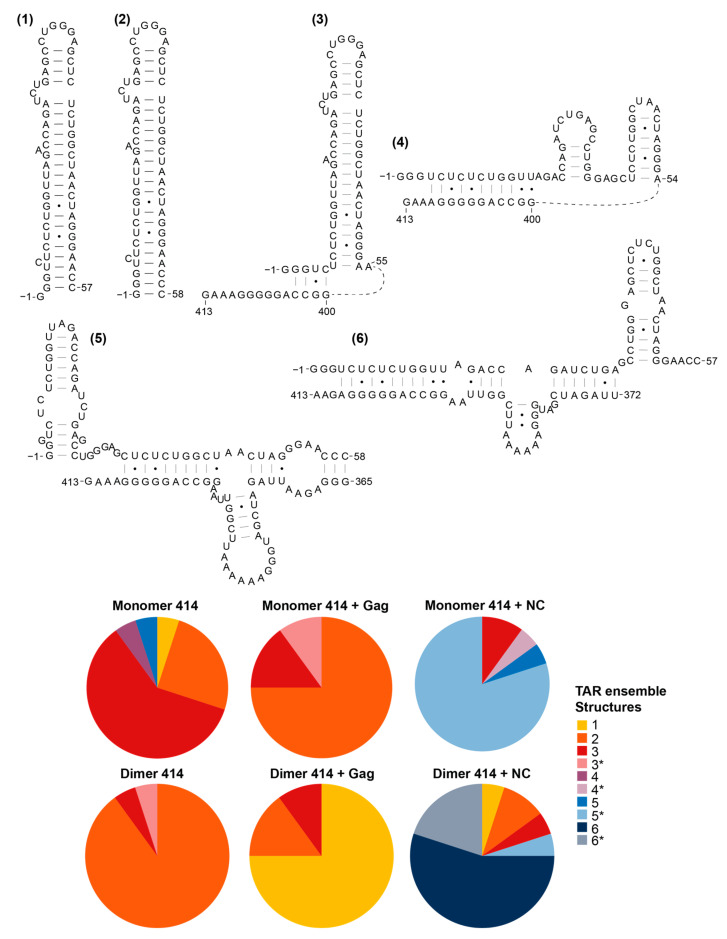
Shift in the structural ensemble of TAR in the presence of Gag or NC. Six structural conformations of TAR as modelled by RNAstructure in 20 modelled structures of the monomer 414 and dimer 414 with and without Gag or NC. The smallest slices, such as ensemble structures 1, 4 and 5, within monomer 414 represent 5% of the ensemble, in this and subsequent figures. * in the pie charts identifies variants of the structure that differ by up to two structural elements. RNA is numbered from the -1 position, as the transcriptional start site (and hence the number of Gs at the 5′ end) varies within cells.

**Figure 7:**
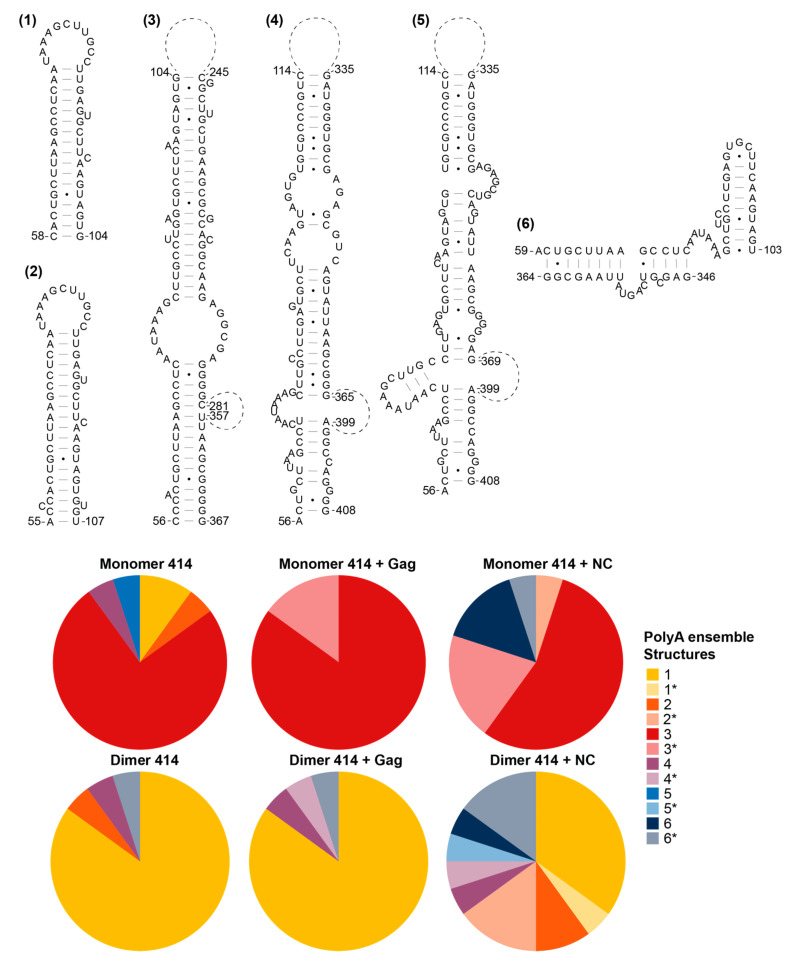
Shift in the structural ensemble of poly(A) in the presence of Gag or NC. Six structural conformations of poly(A) as modelled by RNAstructure in 20 modelled structures of the monomer 414 and dimer 414 with and without Gag or NC. * in the pie charts identifies variants of the structure that differ by up to two structural elements.

**Figure 8:**
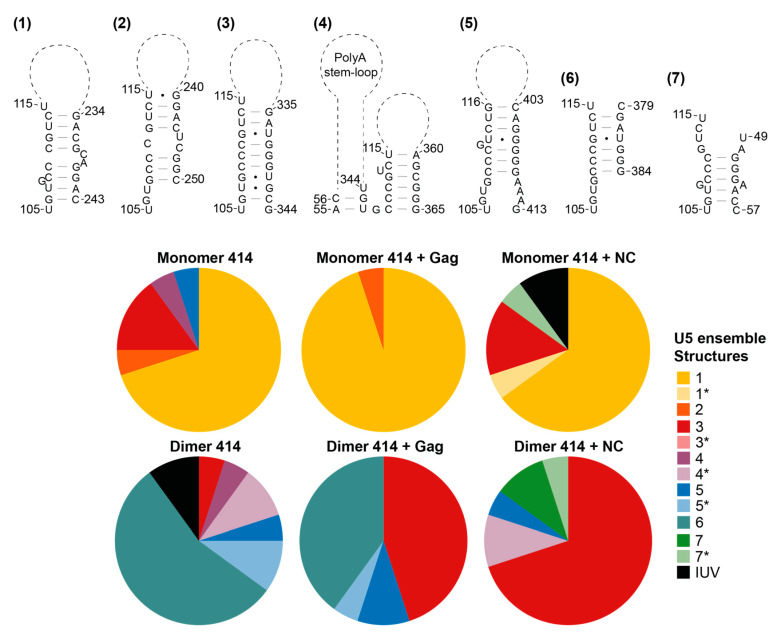
Shift in the structural ensemble of U5 in the presence of Gag or NC. Seven recurring structural conformations of U5 as modelled by RNAstructure in 20 models of the monomer 414 and dimer 414 with and without Gag or NC. * in the pie charts identifies variants of the structure that differ by up to two structural elements. IUV indicates isolated, unclassifiable variants.

**Figure 9:**
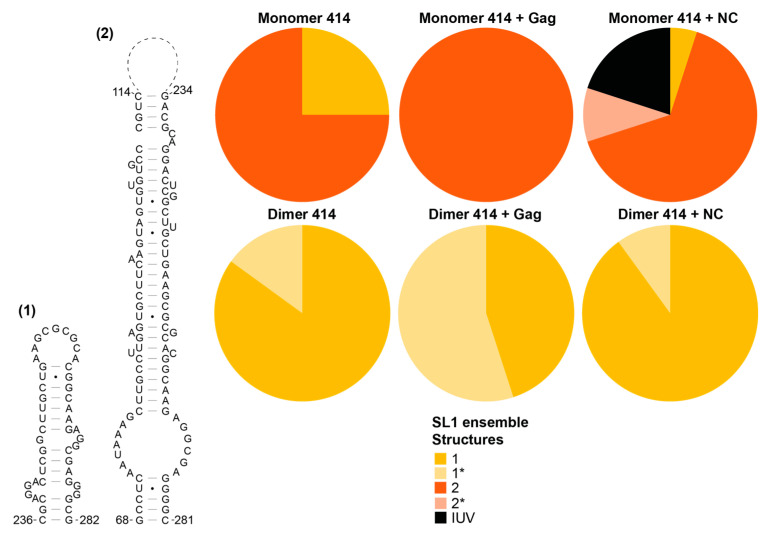
Shift in the structural ensemble of SL1 in the presence of Gag or NC. Two recurring structural conformations of SL1 as modelled by RNAstructure in 20 models of the monomer 414 and dimer 414 with and without Gag or NC. * in the pie charts identifies variants of the structure that differ by up to two structural elements. IUV indicates isolated, unclassifiable variants.

**Figure 10:**
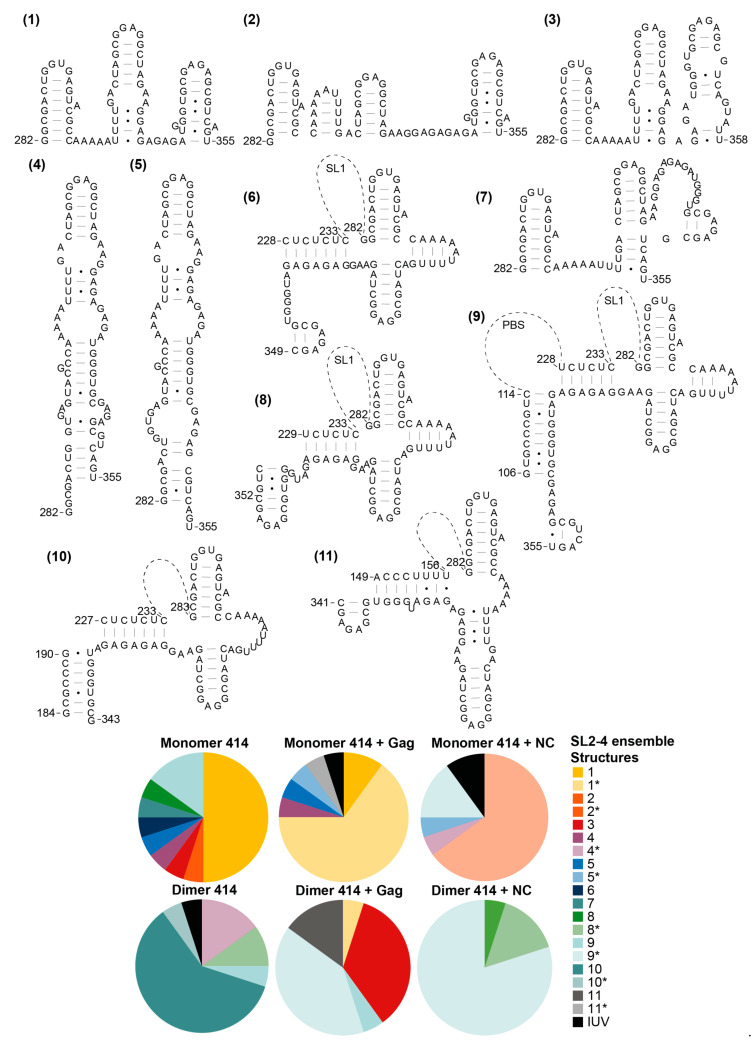
Shift in the structural ensemble of SL2, 3 and 4 in the presence of Gag or NC. Eleven structural conformations of SL2, 3 and 4, as modelled by RNAstructure in 20 models of the monomer 414 and dimer 414 with and without Gag or NC. * in the pie charts identifies variants of the structure that differ by up to two structural elements. IUV indicates isolated, unclassifiable variants.

We then went on to examine the structural ensemble of the RNA bands probed, focusing on the TAR (Figure 6), poly(A) (Figure 7), U5 (Figure 8), SL1 (Figure 9) and SL2, 3 and 4 ensembles (Figure 10). From the RNAstructure output of 20 structures of ligand-naive monomer (which are shown as Supplementary Figures S3–S8, modelled using data shown in Supplementary Table S1), each of these elements was examined to identify the range of structures within the ensemble, and for each of the populations probed (monomer and dimer, with or without Gag or NC) the structural forms of each element within the ensemble of 20 structures were counted. Where new structures were encountered in later ensembles (for example, the ligand-naive dimer), these were added and are shown within these figures. Where a slight variation in one of these structures was identified that differed in up to two structural elements (such as a single helix or a small stem–loop), this was scored as a variant of the original structure and marked as * on the pie charts. The exception to this was structures that varied by a smaller amount at the base of TAR, poly(A) or stem–loop 3, which have all been implicated in the packaging/translation switch, and were therefore depicted and counted separately.

The monomeric RNA that had not encountered Gag/NC predominantly contained a fully or partially disrupted TAR structure, poly(A) involved in an LRI with SL1, similar to the LDI model, and detectable but minimal U5:AUG formation, whereas there was significant heterogeneity in the SL2–4 ensemble: the predominant structure contained a slightly extended SL3 and an intact SL4 (Figure 6, Figure 7, Figure 8, Figure 9 and Figure 10). The monomeric RNA that had bound Gag, but not dimerised, mostly contained an intact TAR stem–loop, had poly(A) and SL1 entirely within an LDI-type structure with no U5:AUG interaction detectable and a similar SL2–4 ensemble to the unshifted monomer (Figure 6, Figure 7, Figure 8, Figure 9 and Figure 10). The monomeric RNA that had bound NC had no detectable TAR structure in the majority of the ensemble, had no detectable poly(A) stem–loop but instead a variety of long-range interactions; U5:AUG and/or SL1 were present in a small minority of the ensemble, and SL2–4 were similarly structured to the shifted monomeric ensemble with Gag, except that SL3 was shorter and an additional A-U-rich stem–loop forms between SL2 and SL3.

The dimeric ensemble was modelled as the ‘hemi dimer’, with the additional constraint of the GCGCGC DIS sequence being maintained as single-stranded to enable pairing with its counterpart in the second molecule. The ligand-naive dimer ensemble predominantly contained a stable TAR and poly(A) stem–loop, but not the U5:AUG helix, SL1, and the SL4 sequence formed long-range interactions. This ensemble conformed to the BMH model except for the U5:AUG interaction, which was instead an alternative long-range interaction. The dimeric ensemble that had bound Gag mostly formed a TAR that was not additionally stabilised at the base by the 5′G, the poly(A) stem–loop, the U5:AUG helix (although there was still considerable heterogeneity in this structural element), SL1 and SL4 forming the same long-range interactions as in the BMH. Overall, the majority of the structural ensemble was in the BMH or close to the BMH form. The dimeric ensemble with NC bound had an almost entirely disrupted TAR stem–loop, with this sequence forming various long-range interactions, considerable heterogeneity in the poly(A) ensemble, the U5:AUG helix predominantly intact, and the majority of the SL2–4 ensemble containing the same long-range interactions as are found in the BMH model.

**Figure 11:**
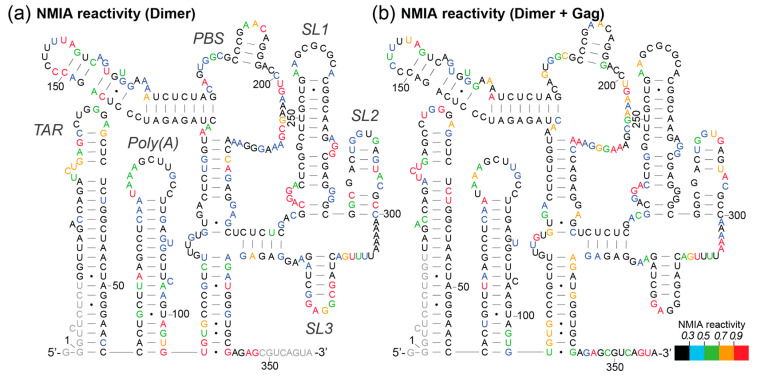
SHAPE reactivity at each nucleotide mapped onto the BMH structural model for dimeric RNA without Gag bound (**a**) and with Gag bound (**b**). Legend indicates the colour scheme for the intensity of NMIA reactivity, where black is reactivity of less than 0.3 and red is reactivity greater than 0.9. Structures were taken from Kenyon et al. (2013). (Dimer, *n* = 5; dimer + Gag, *n* = 6.)

To illustrate the narrowing of the structural ensemble into the BMH form when Gag binds to the dimer, the improved fit of the SHAPE data are shown mapped onto the BMH structural model when Gag is not/is bound to the dimer (Figure 11a,b). The increased stability of the known metastable AGG loop [55] on the 3′ side of SL1 (nts 271–273) when Gag binds to the dimer, alongside the overall stabilisation of the proximal half of SL1 (nts 238–240 and 278–279), suggests that the extent of intermolecular dimerisation (or ‘extended duplex’ [44,56]) has increased. In this structure, the helix of SL3 is also stabilised (Figure 11b), as are additional nearby structures, SL2 and the U5-AUG helix. The helix subtending SL1–SL3 is also stabilised, as shown by the lowered reactivity of A332.

Overall, the ensembles suggest that Gag either specifically selects structures that resemble the BMH model, or that it remodels the RNA into the BMH when it binds. It can bind to alternative structures, as evidenced from the shifted monomer population, but these do not then remodel into a dimerisation-competent structure within the timeframe of this experiment, at this relatively low concentration of Gag.

### 3.6. The First Gag Binding Sites Are in TAR and Poly(A), Whereas NC Reacts Extensively at Multiple Sites

**Figure 12:**
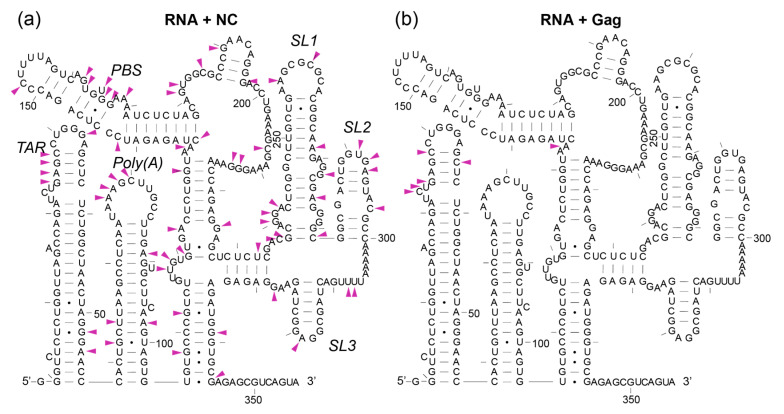
Photo-induced crosslinking followed by primer extension identifies the specific sites of NC or Gag interaction depicted on the BMH RNA structure. Purple arrowheads indicate sites of interactions as detected by XL-SHAPE (statistically significant by t-test, *p* < 0.05, and within the top 20% of cross-linking reactivity values) with (**a**) NC–RNA (cross-linked, no protein, *n* = 6; cross-linked, NC, *n* = 4; cross-linked, AP, *n* = 4; no cross-linking, no protein, *n* = 7; no cross-linking, NC, *n* = 4; and no cross-linking, AP, *n* = 4). (**b**) Gag–RNA. (cross-linked, no protein, *n* = 6; cross-linked, Gag, *n* = 5; cross-linked, AP, *n* = 4; no cross-linking, no protein, *n* = 7; no cross-linking, Gag, *n* = 3; and no cross-linking, AP, *n* = 4).

SHAPE coupled with photo-cross-linking (XL-SHAPE) has been used to identify protein binding sites and the impact of protein binding on RNA structure remodelling [49]. The advantage of this method is that, in an RNA–protein complex, the SHAPE reagent is much more sensitive to RNA structural perturbations than it is to the presence of a protein, and can thus identify changes to RNA flexibility at the protein binding site as well as across the rest of the RNA structure [49]. To distinguish structural change in the RNA from true Gag protein binding sites we performed photo-cross-linking to inform our in-gel SHAPE data [49]. We used the same molar ratio of 15× protein to RNA in both the SHAPE and cross-linking experiments (Figure 3). To identify the contribution of the NC component to the Gag–RNA interaction, we also performed cross-linking with the NC protein. This showed that NC binds the 5′-UTR promiscuously. It binds across the major packaging signal region, between nts 224-234, as seen previously, in an in virio analysis [57] and at the AU-rich single-stranded region between SL2 and SL3 (Figure 12a and Supplementary Table S1), which was seen as a Gag binding site by XL-SHAPE previously when using a 50× molar ratio of protein to RNA [49]. Binding outside of the major packaging signal region was not previously detected in virio [57], indicating greater specificity of NC for the viral RNA when it has first interacted while still part of Gag, and subsequent interactions in the cleaved form occurring within the context of the virion. The promiscuity seen here in XL-SHAPE experiments relates to the NC protein in isolation, and is commensurate with its role as a nucleic acid chaperone when it is in its cleaved form. Our results suggest that at a 15× molar ratio we detect Gag binding to the RNA dimer mainly within the TAR stem loop (Figure 12b and Supplementary Table S1), as well as close to the PBS. This raises the possibility that the initial contact sites of Gag on the RNA are within the TAR region, and that within the context of the full-length Gag, the interactions within the Ψ domain only occur at higher molar ratios, likely after RNA structural remodelling. It may also be that in these experiments NC, as a much smaller protein, is able to access sites in the RNA, such as SL3, more easily, without any remodelling of the RNA. 

## 4. Discussion

In-gel SHAPE is a precise and versatile technique that has been used to successfully separate and analyse the structure of multiple RNA conformers within a mixed population [31]. Here, we used in-gel SHAPE to separate a mixed population of structural conformers of RNA–protein complexes and identify differences in RNA remodelling in these complexes. Using this technique, it is possible to characterise the structures and structural changes selected for and promoted by the NC and Gag protein, which facilitate the shift from the RNA monomer to the dimer and the subsequent stabilisation of the dimeric BMH conformation. Moreover, we were able to visualise the influence of NC on the dimer that would occur after the proteolytic cleavage of Gag during viral particle maturation.

As Gag concentration increases and higher numbers of Gag proteins bind the viral genome, the RNA is shifted towards a new optimal dimeric conformation (Figure 3). Using EMSA, we identified that the interaction of Gag with the monomeric 5′UTR induces dimerisation at a molar ratio of 10–20 (Gag:RNA). The improved fit of the dimer NMIA reactivity data with a single structure (the BMH model) upon Gag interaction suggests that Gag structurally stabilises the RNA, narrowing the structural ensemble so that it is predominantly a stabilised BMH or BMH-like conformation (Figure 11). The extent of intermolecular interaction in the dimer is the subject of debate, but a rearrangement involving an intermolecular U5:AUG helix has been shown by NMR [44]. Our data substantiates this by the increased stabilisation of the U5:AUG helix and SL1 when Gag is bound to the dimer (Figure 11). Above a molar ratio of 30, additional Gag proteins bind the dimer up to a maximum beyond which no further binding is detectable (Figure 3). At relatively low Gag concentrations of 15×, some of the monomeric RNA ensemble appears to be ‘trapped’ in an LDI-like conformation that has not dimerised upon binding Gag (Figure 6, Figure 7, Figure 8, Figure 9, Figure 10 and Figure 11). At higher Gag concentrations, however, all of the RNA is dimeric (Figure 3), suggesting that binding of subsequent Gag molecules remodelled the LDI-like structure into a dimerisation-competent RNA. Our observations fit with a model where high-affinity binding sites for Gag on the gRNA are occluded until Gag concentrations reach sufficient levels to enable a switch from translation to packaging of the genome. Such concentrations would thus enable interaction with initial lower-affinity binding sites that effect a structural change to expose the higher-affinity sites, mediating co-operative binding of Gag onto the RNA. Our data suggest this may happen via a combination of RNA structural and protein structural change, as 15× NC binds to Ψ but 15× Gag does not. Instead, full-length Gag first binds to sites in TAR, which changes the RNA structure, presumably making it easier for the NC domain of Gag to then interact with the Ψ region. This interaction of lower concentrations of Gag with TAR may facilitate translation by recruiting translation-promoting factors or by altering RNA structure. Indeed, low levels of Gag have been shown to promote translation, and higher levels to promote packaging [58]. If Gag is to act as an early primary control protein determining the fate of the RNA, then there is a logic to it interacting initially with the first regions of the RNA to emerge from the ribosome when it is being translated, and this consideration may also be relevant during transcription [59]. NC only encounters the gRNA after Gag has been cleaved by PR, and its roles in processing the RNA fit with its binding sites being widely distributed. TAR has also previously been shown to be structurally important for RNA packaging [60,61], and the TAR and poly(A) regions were shown to cross-link with Gag inside cells to a moderate degree [62]. In addition, small differences in sequence at the base of TAR have been shown to modify not only the TAR structure, but to propagate across the Ψ region and affect packaging [33]. At 414 nt in length, the RNA studied here contained further *gag* sequences compared with previous studies of the effects of transcriptional start site heterogeneity. These 3′ nucleotides appeared to widen the structural ensemble we observed, as they were able to pair with the base of TAR, destabilising it to some extent, and hence enabling the structures previously seen in the presence of the 2G transcript to form. Our observations once again highlight the potential multifunctionality of the TAR RNA and its role in the translation/packaging switch, which make it an attractive drug target.

A specific SL3-binding drug has been shown to be an effective small-molecule inhibitor of HIV packaging, and multiple methods have identified the GGAG tetraloop at the apex of SL3 to be a binding site for NC and Gag [9,49,63,64,65,66]. However, in our cross-linking experiments using minimal Gag, binding at this region was not observed, and it was not until higher molar ratios of Gag that this interaction was detected previously [49]. Clearly, SL3 must play a specific and vital role in genome packaging, but our data suggest that that role is not as the initial site of interaction for Gag, either in the monomer or the dimer.

Our results suggest that SL3 is stabilised in the dimer in the presence of Gag, possibly by the formation of extended intermolecular interactions 5′ and 3′ of it. This can be seen in Figure 10 when comparing the ensemble for the dimer (where no extended SL3 is seen) and the dimer plus Gag, where more than 50% of the ensemble is in an extended SL3 structure. Nucleotides 5′ and 3′ of SL3 have been proposed to be important binding sites for Gag, and for the control of genome packaging [67]. They can interact to form a small helix, and such a structure is thought to be more readily adopted when the TAR helix contains one capped or two uncapped 5′Gs in the transcriptional initiation switch proposed to control packaging and translation [67]. Our data suggest that a structure such as this is stabilised by the binding of Gag to TAR, hinting that perhaps such a structure is selected by Gag and then structurally re-enforced by its binding.

Gag binding has also been shown to fully unwind SL3 RNA, whereas NC is unable to do this [68], and it may be that this interaction of Gag with SL3, and the remodelling it causes, is necessary to propagate the full extent of the necessary intermolecular interactions between the two molecules of the gRNA. In support of this theory is the observation that stabilising the UUUU:GGAG interaction at the base of SL3 that NC binds to actually reduces viral packaging [67]. In addition, the ability of a specific SL3 binding compound to stabilise the RNA structure of the whole of the RNA leader region, prevent Gag binding and effectively lower viral packaging as well as infectivity is also suggestive of the fact that the SL3 helix may need to unwind in order to form the extended intermolecular interactions on either side of it [9].

The BMH conformation found in the dimeric ensemble has a number of SHAPE reactivity differences from the monomeric ensemble structures, notably within and surrounding SL1 and the U5:AUG helix. We observed sites of stabilisation and destabilisation along the stem and bulges of SL1 upon Gag binding to the RNA, which presumably facilitate the transition to the BMH model (Figure 4). It has been previously demonstrated that the SL1 internal loop is a major binding site for Gag [65,69,70], but the XL-SHAPE results presented here suggest that it is not an initial site of contact but a later one [49]. It may be that, similarly to SL3, the structural lability that SL1 provides during Gag binding is important for RNA remodelling into a packageable structure that can later be effectively reverse-transcribed.

In the dimer, upon Gag binding, there are sites of destabilisation in the PBS: the loop region above the U5:AUG, the loop of SL2 and the AU-rich sequence 5′ of SL3 (Figure 4). These changes may make the structure more compatible for reverse transcription and the annealing of the tRNA primer required to initiate this process.

The proteolytic maturation of Gag by the viral protease during viral particle maturation is vital for gRNA dimerisation and stability. Genomic RNA extracted from immature virions shows reduced stability, similar to that of a loose dimer [36,37,71,72]. Moreover, genomic RNAs extracted from protease-deficient virions were found to be in the monomeric conformation [73,74]. We show that, comparing the changes in NMIA reactivity between NC or Gag bound to the dimer, many of the impacts upon RNA flexibility are similar. However, the NC–dimer complex shows a higher degree of instability in the poly(A) stem–loop and the PBS, which may be vital changes for efficient virion maturation, as destabilisation of secondary structures would lessen the pausing and dissociation of the reverse transcriptase. This illustrates the importance of the temporal control of the proteolytic maturation of Gag protein.

Overall, our data suggest a mechanism by which HIV-1 effects and times its packaging process to ensure that packaging only begins once sufficient Gag has been translated, and highlight the important gRNA sites and structures that allow packaging to begin. Such insights may provide new drug targets against HIV.

## Data Availability

SHAPE data are provided as Supplementary Table S1.

## Supplementary Materials

The following are available online at https://www.mdpi.com/article/10.3390/v13122389/s1. Figure S1: Structural model of the TAR hairpin in the absence of Tat, based upon the NMIA reactivity data obtained from in-gel SHAPE. Figure S2: Gel shift in the presence of 1:15 molar ratio of 5′ leader sequence RNA to NC protein (LH panel) (*n* = 4) or Gag protein (RH panel) (*n* = 4). Areas within the dotted lines represent the bands cut out of the unstained gel. Figures S3–S8: The 20 lowest free-energy models of the monomer 414 and dimer 414, without and with NC and Gag, as modelled by RNA Structure, in order of increasing free-energy. Table S1: SHAPE data.
