# Supplementary material for: HIV-1 Packaging Visualised by In-Gel SHAPE
Source: Viruses. 2021 Nov 29;13(12):2389. doi: 10.3390/v13122389 (PMC8707378; doi:10.3390/v13122389)
Supplement: Supplementary file 1 [file viruses-13-02389-s001.zip › viruses-1453260-supplementary.pdf]

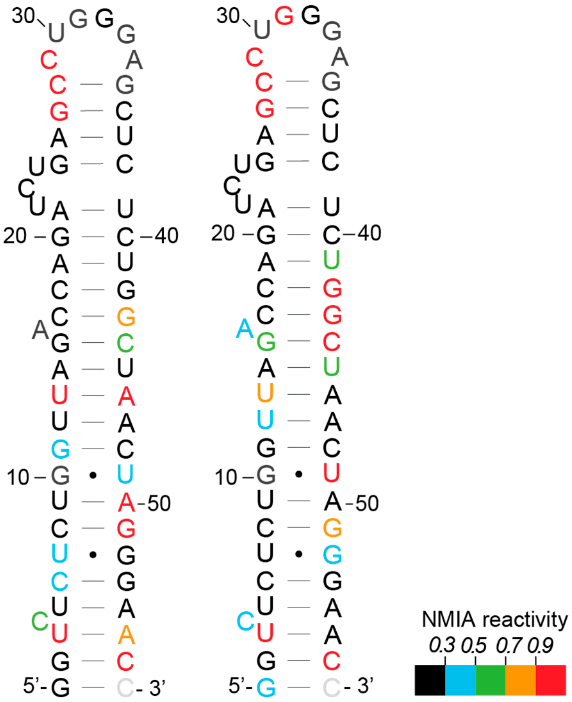

Supplementary figure 1: Structural model of the TAR hairpin in the absence of Tat, based upon the NMIA reactivity data obtained from in-gel SHAPE. The two models each represent one independent experiment. Standardised nucleotide reactivities (arbitrary units) are shown in colour according to the key. Black represents nucleotides with reactivity below 0.3 (rigid backbone; most likely to be base-paired) and red represents nucleotides with reactivity above 0.9 (flexible backbone; most likely to be single-stranded).

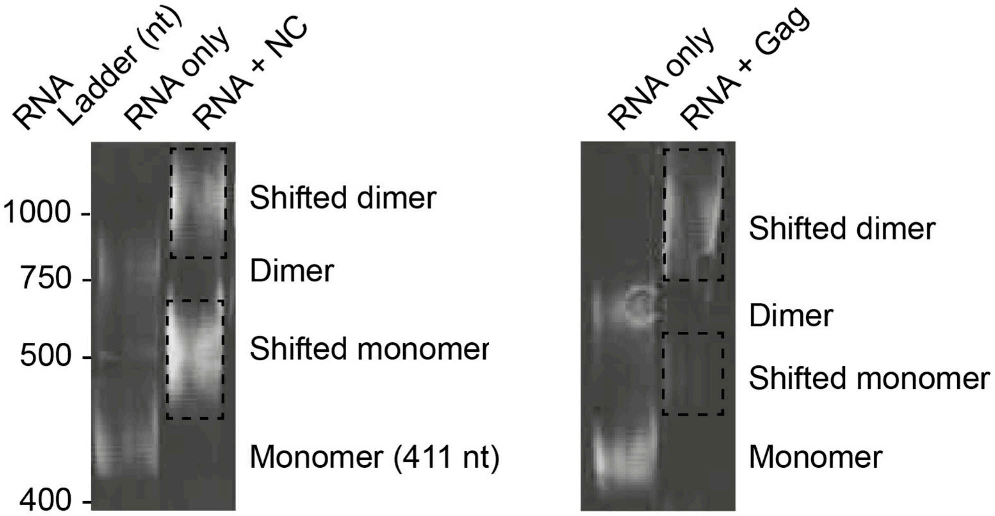

Supplementary figure 2. Gel shift in the presence of 1:15 molar ratio of 5' leader sequence RNA to NC protein (LH panel) (n=4) or Gag protein (RH panel) (n=4). Areas within the dotted lines represent the bands cut out of the unstained gel.

Supplementary figures 3-8 – The 20 lowest free-energy models of the monomer 414 and dimer 414, without and with NC and Gag, as modelled by RNAStructure, in order of increasing free-energy.

Supplementary figure 3 - Monomer 414

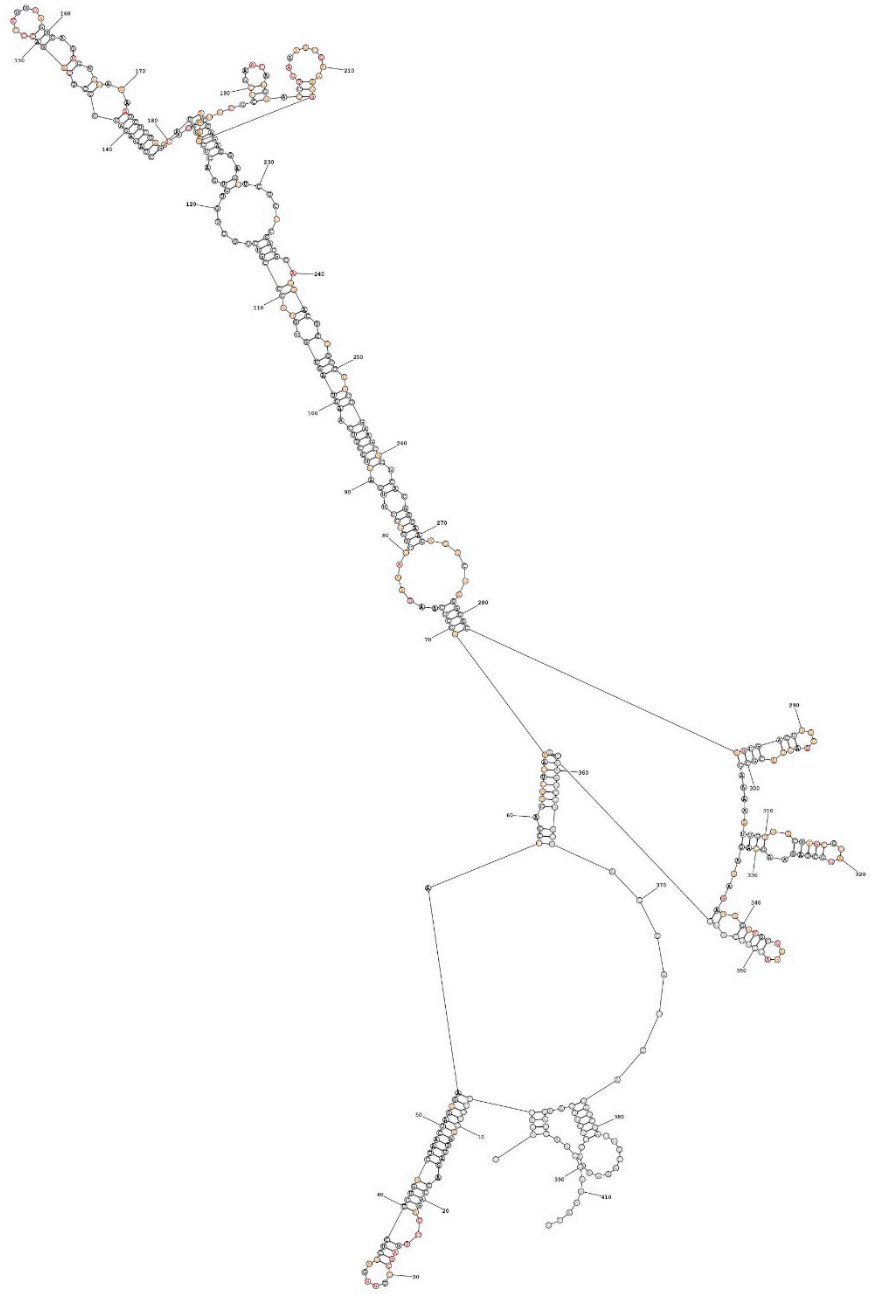

SHAPE >= 0.85

0.85 > SHAPE >= 0.4

0.4 > SHAPE

No Data

ENERGY = -185.8   Mon414

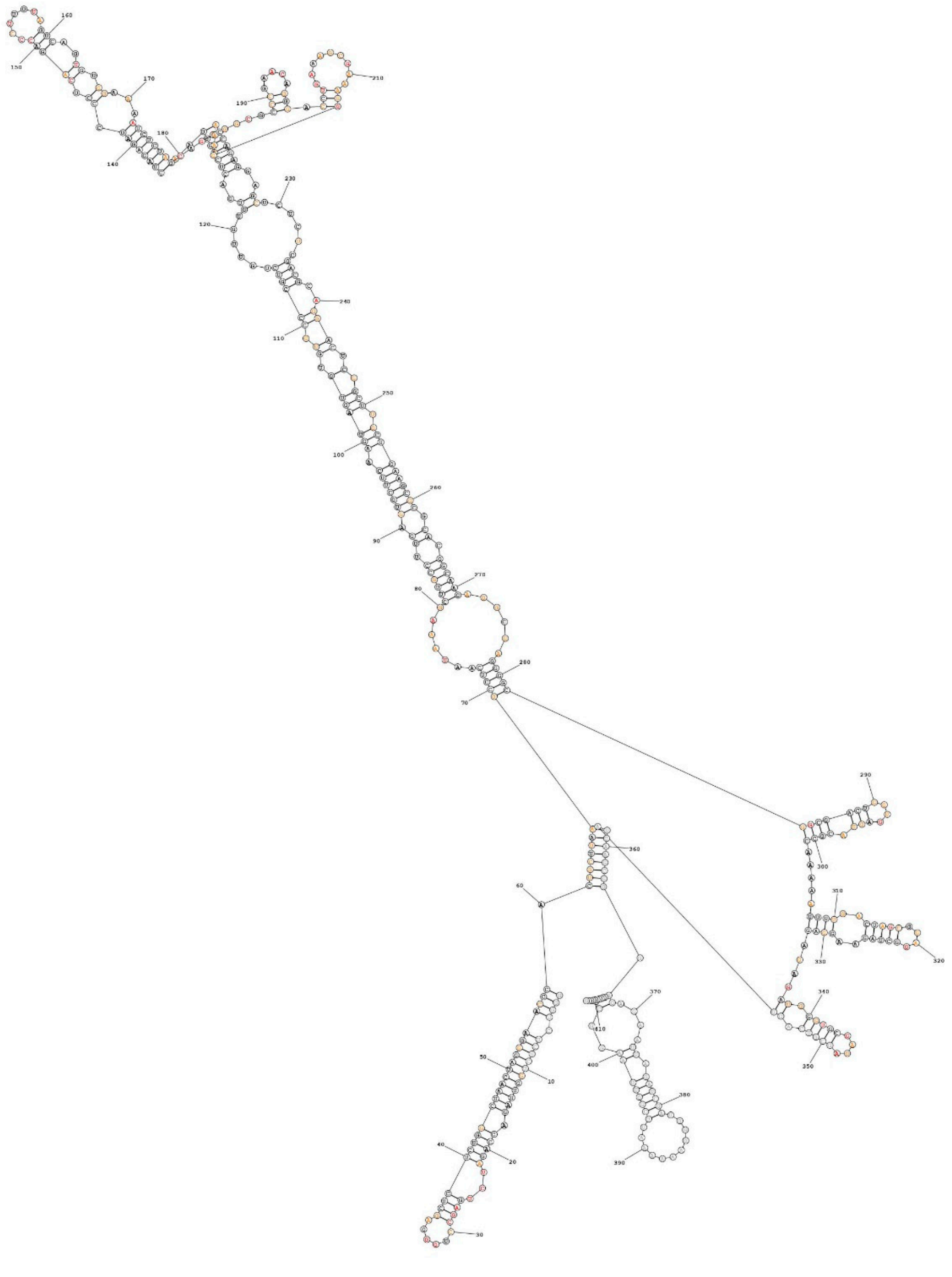

SHAPE >= 0.85

0.85 > SHAPE >= 0.4

0.4 > SHAPE

No Data

ENERGY = -185.0 Mon414

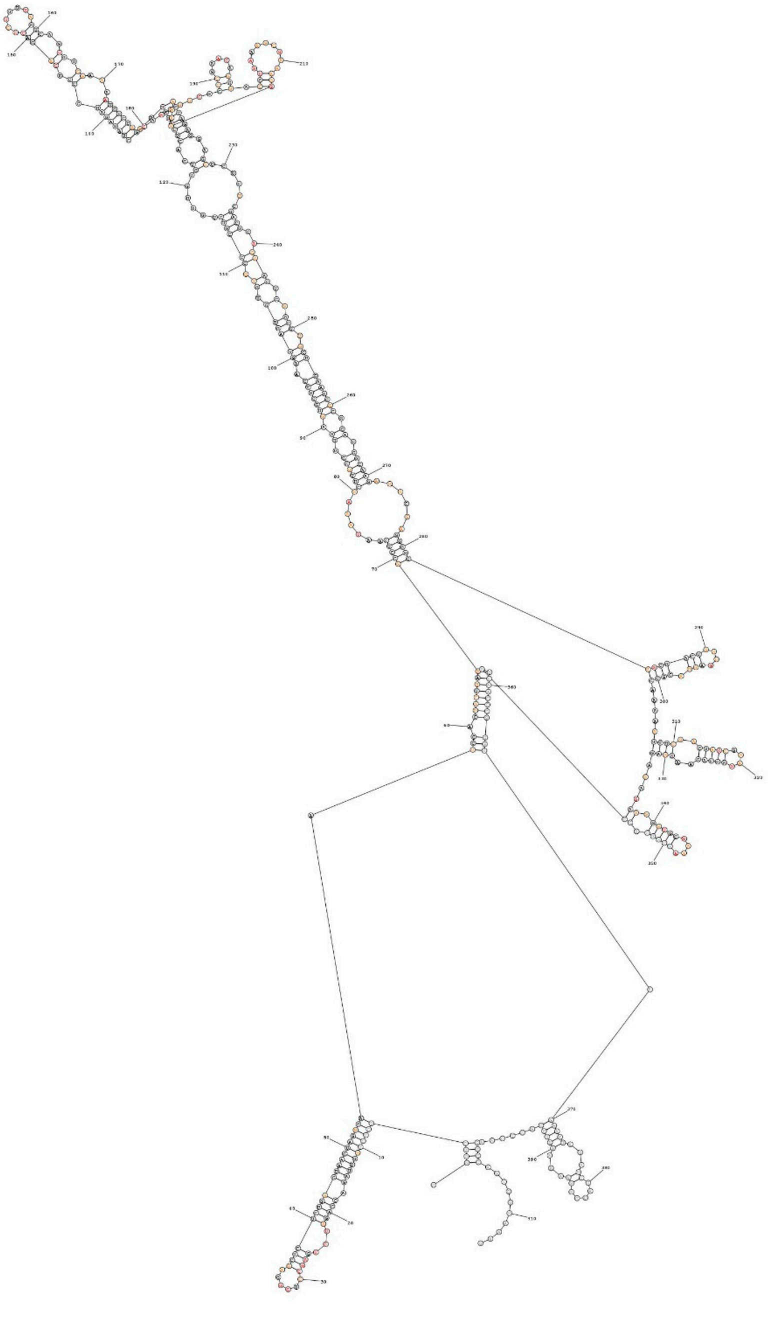

SHAPE >= 0.85
0.85 > SHAPE >= 0.4
0.4 > SHAPE
No Data

ENERGY = -184.9 Mon414

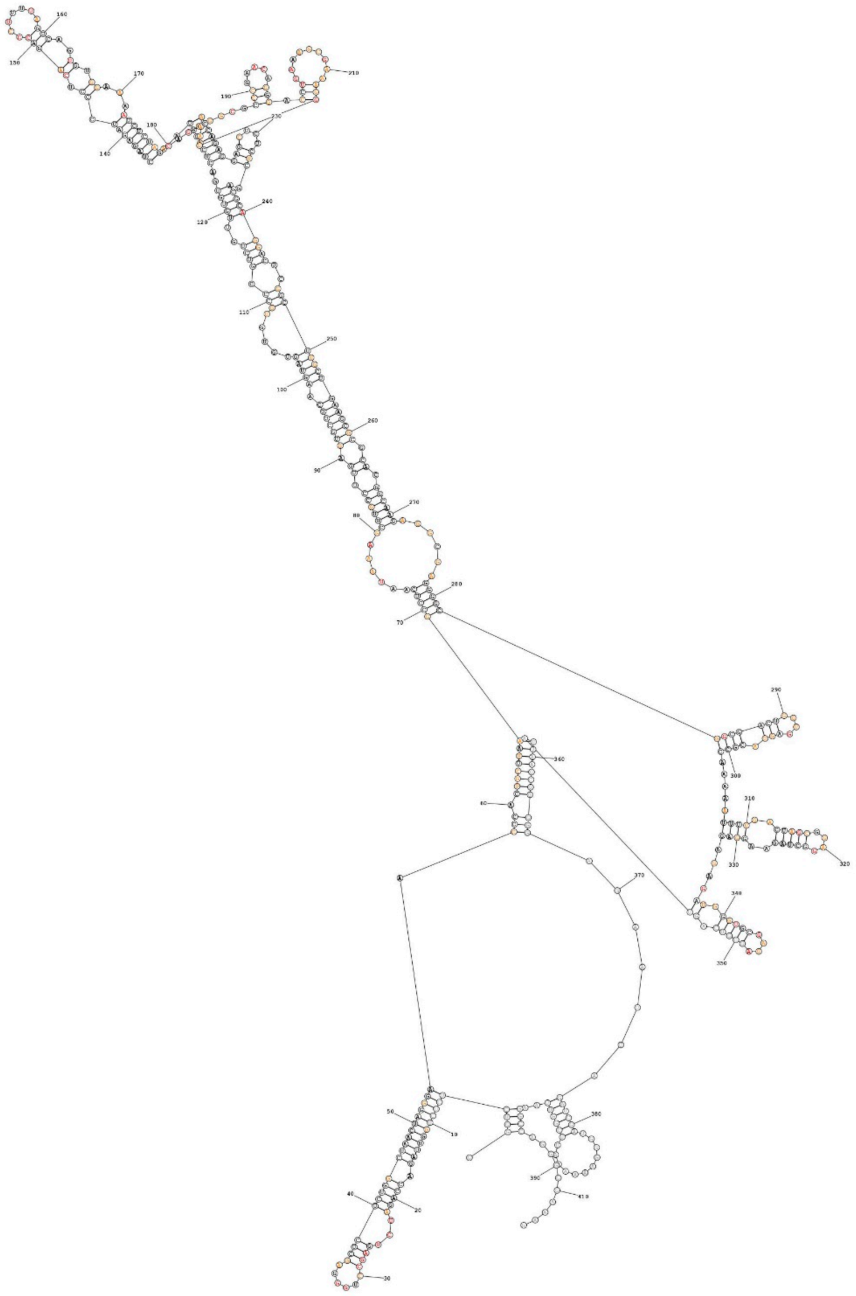

SHAPE >= 0.85

0.85 > SHAPE >= 0.4

0.4 > SHAPE

No Data

ENERGY = -184.3 Mon414

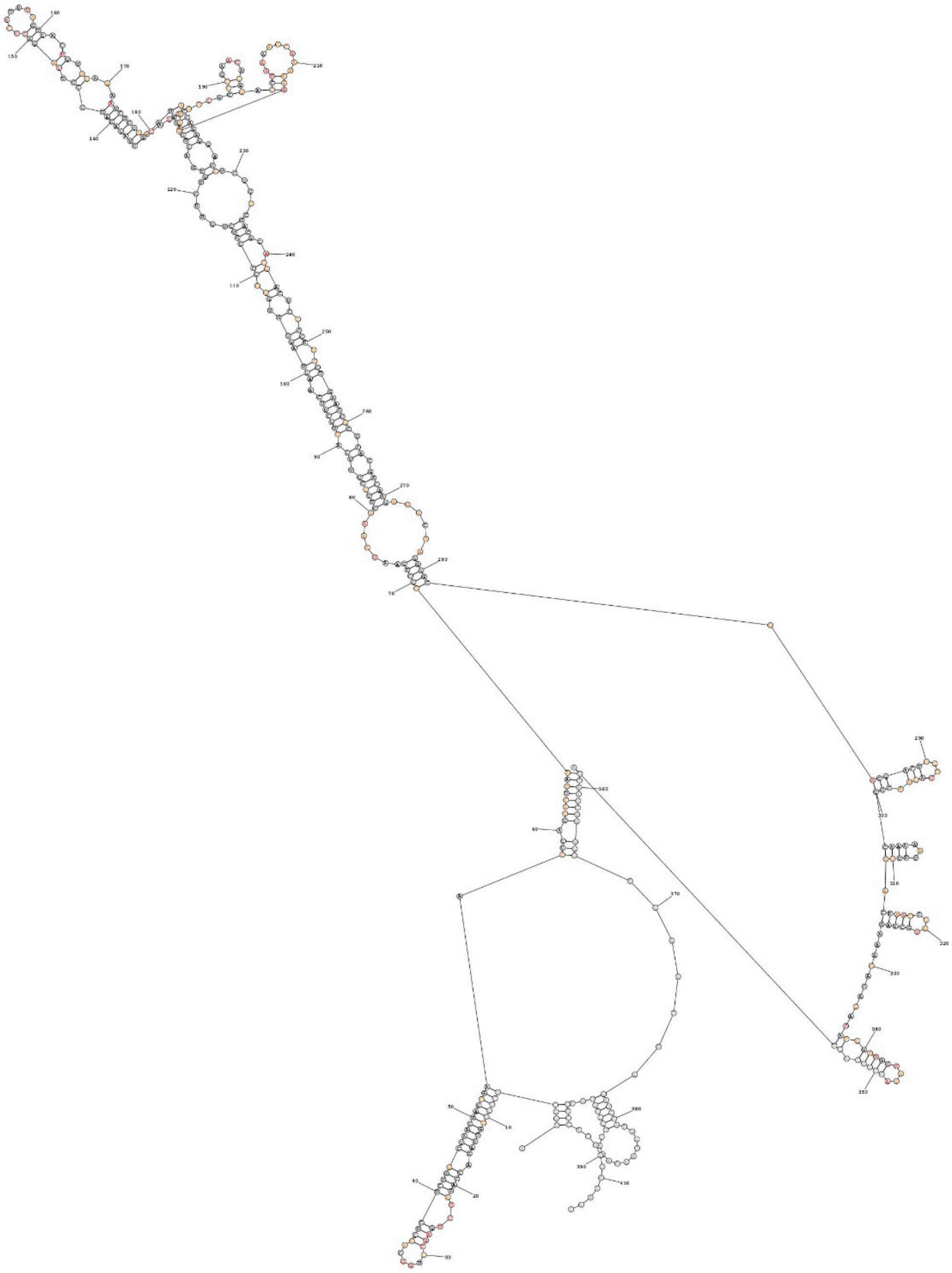

SHAPE >= 0.85
0.85 > SHAPE >= 0.4
0.4 > SHAPE
No Data

ENERGY = -183.8 Mon414

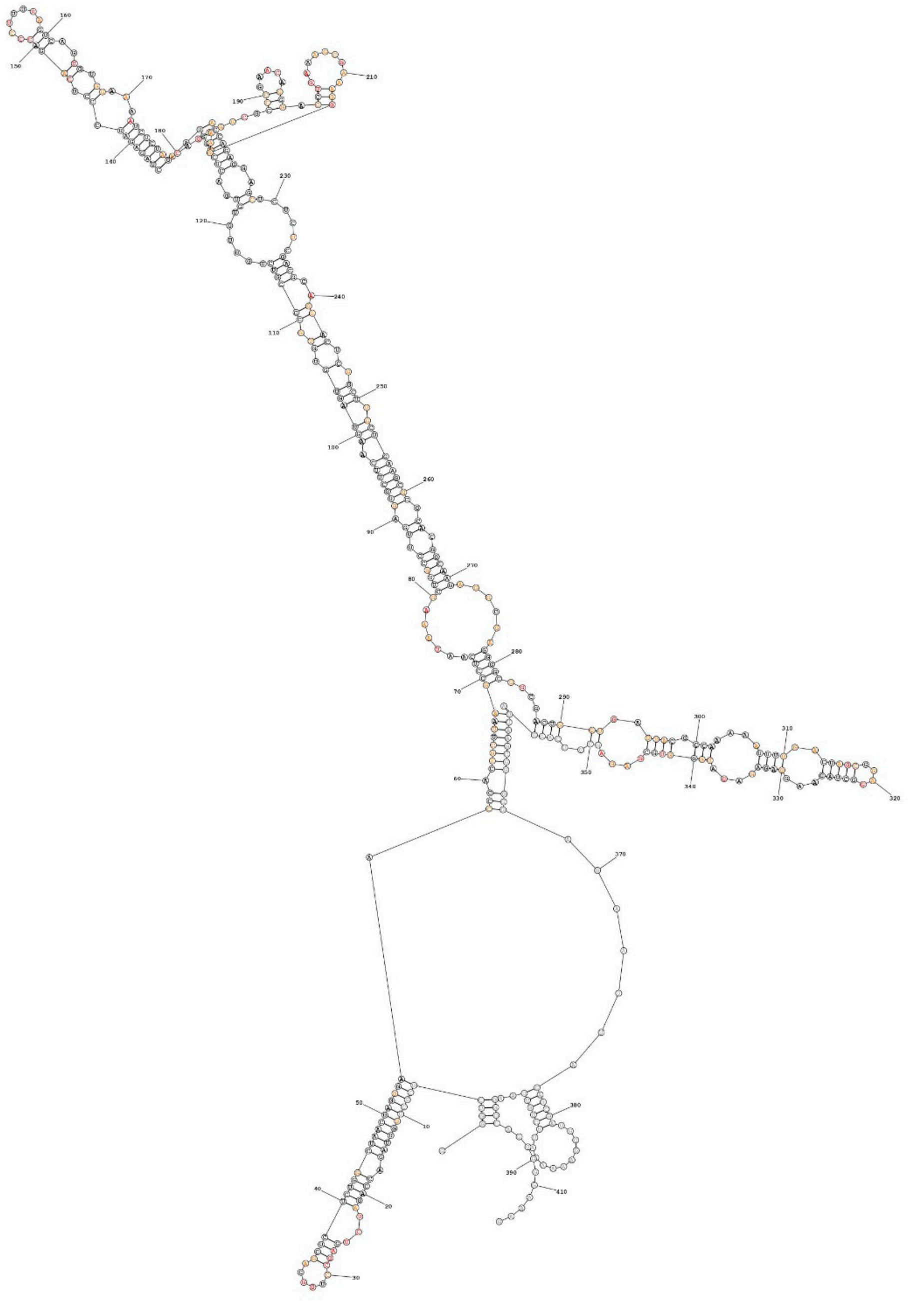

SHAPE >= 0.85
0.85 > SHAPE >= 0.4
0.4 > SHAPE
No Data

ENERGY = -183.0 Mon414

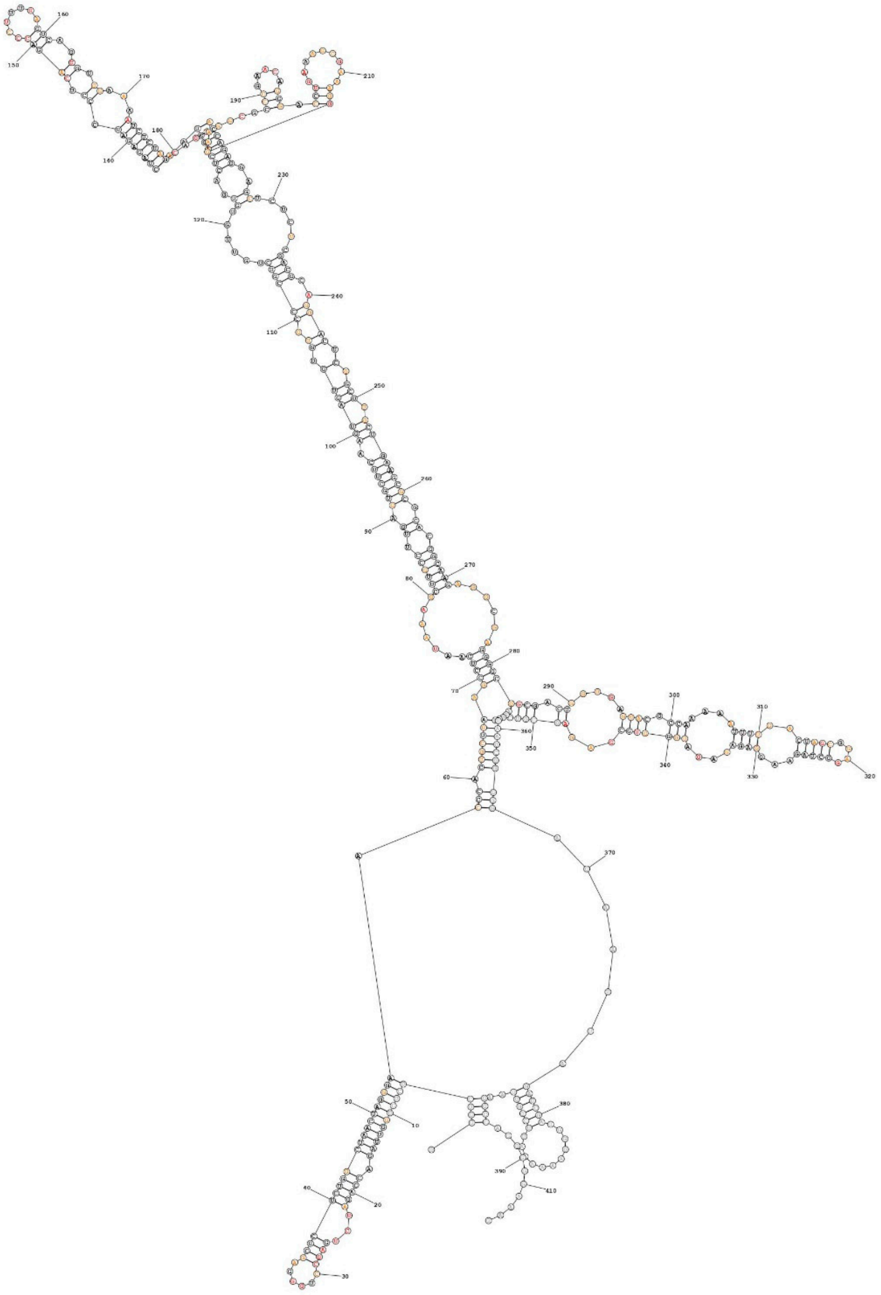

SHAPE >= 0.85
0.85 > SHAPE >= 0.4
0.4 > SHAPE
No Data

ENERGY = -182.7 Mon414

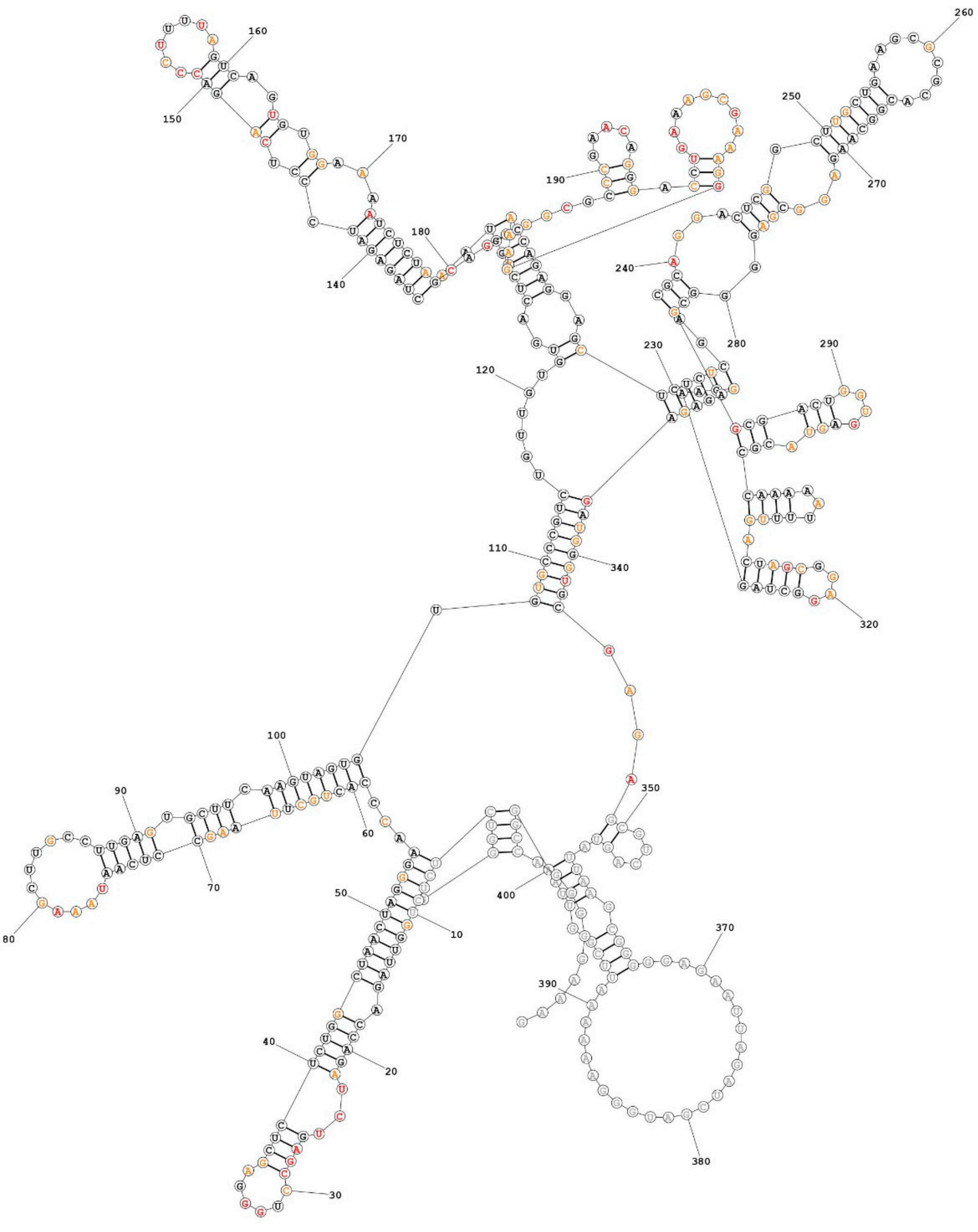

SHAPE >= 0.85
0.85 > SHAPE >= 0.4
0.4 > SHAPE
No Data

ENERGY = -182.3 Mon414

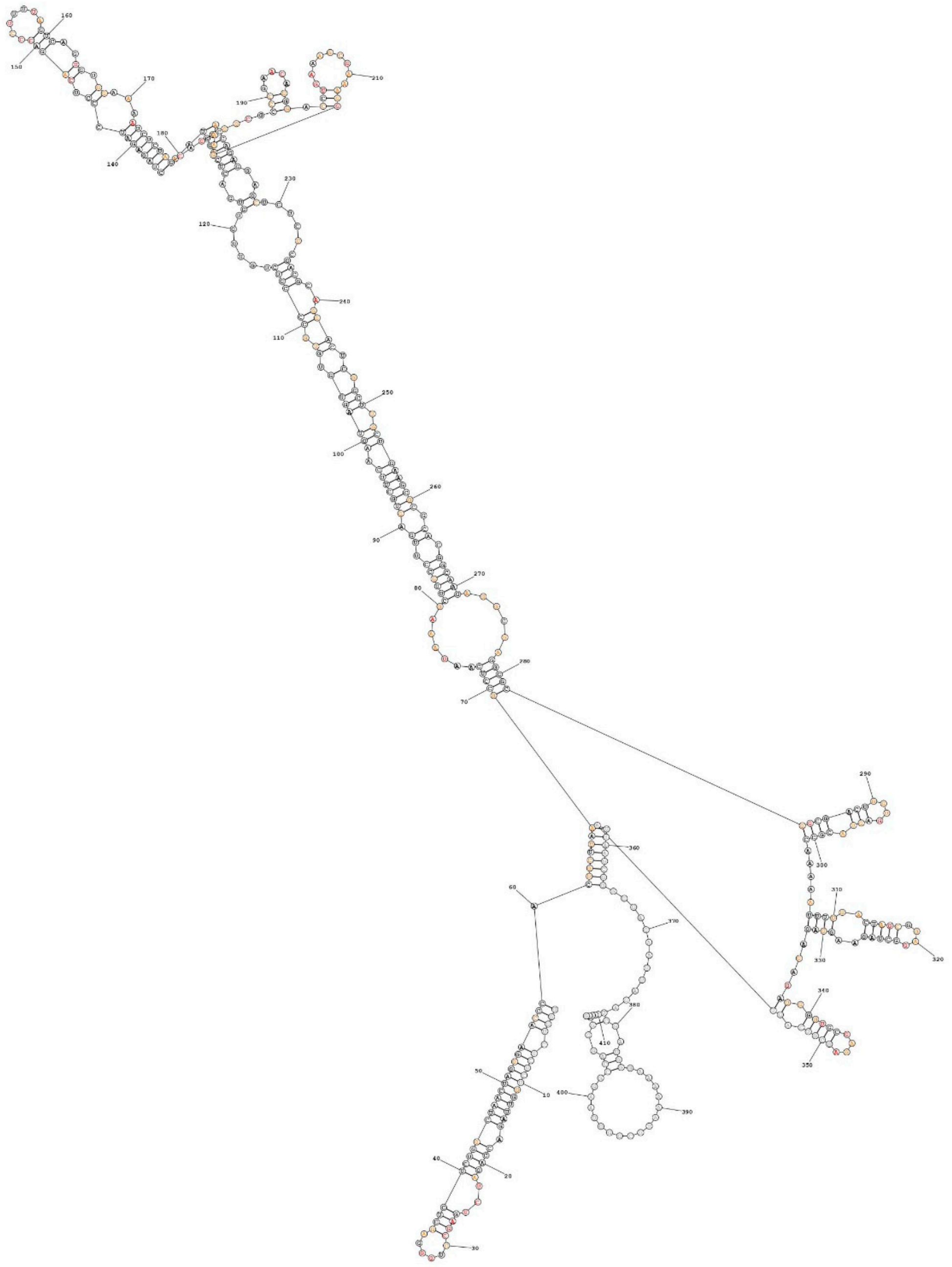

SHAPE >= 0.85
0.85 > SHAPE >= 0.4
0.4 > SHAPE
No Data

ENERGY = -182.2 Mon414

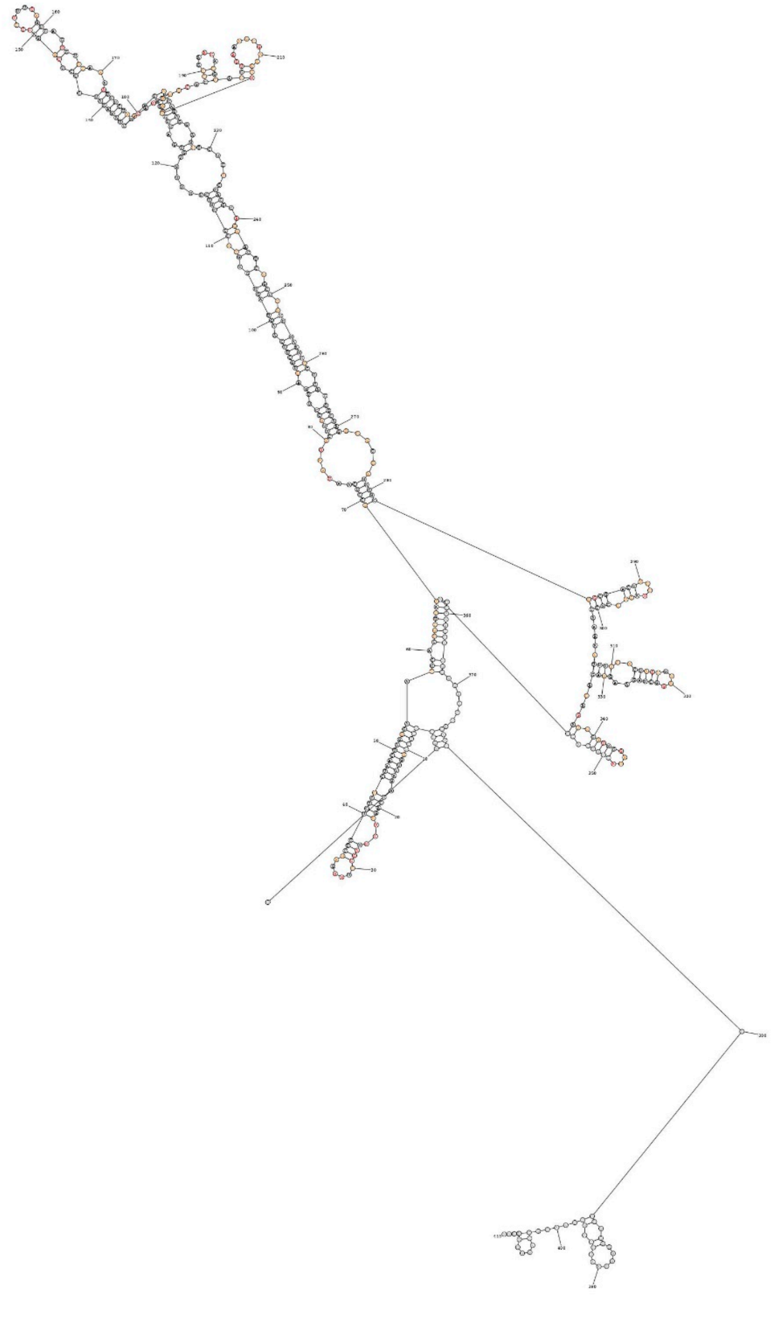

SHAPE >= 0.85
0.85 > SHAPE >= 0.4
0.4 > SHAPE
No Data

ENERGY = -182.1 Mon414

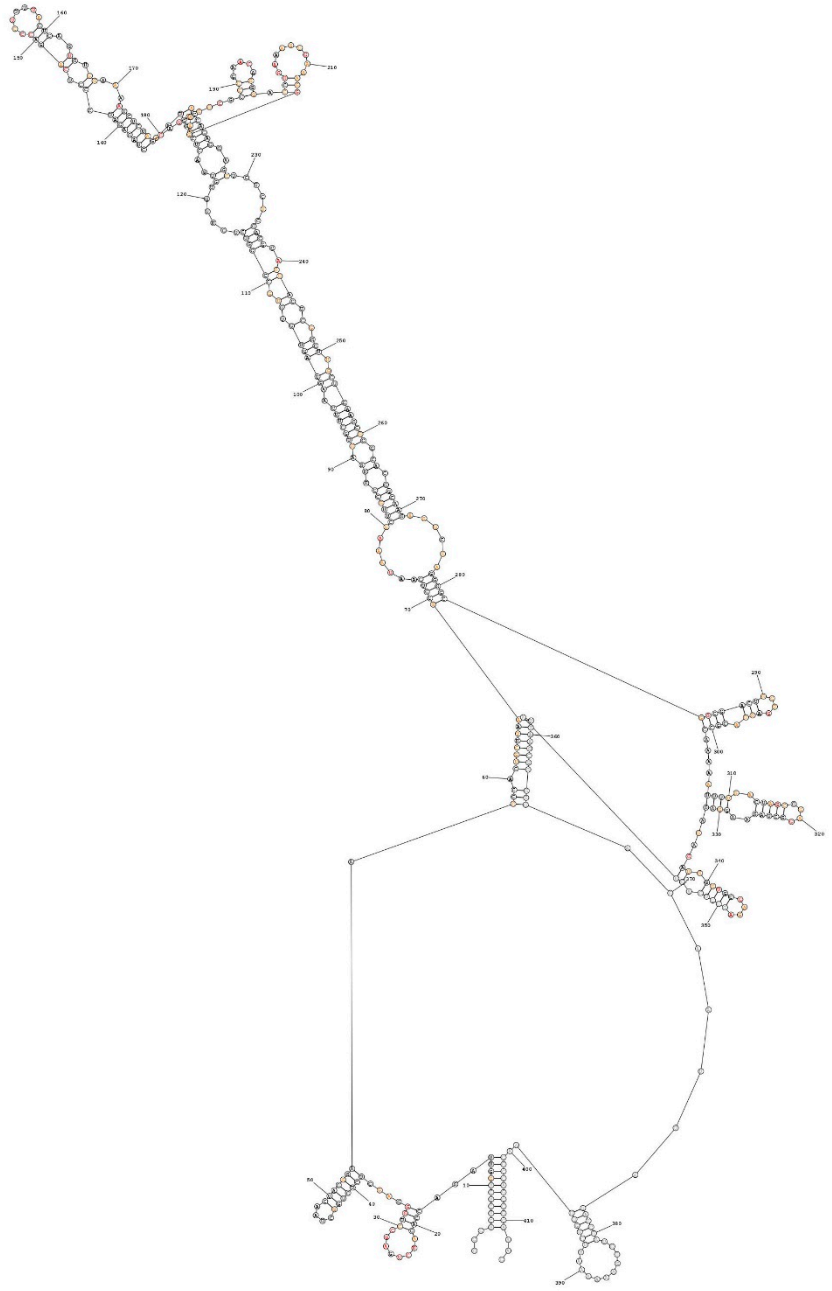

SHAPE >= 0.85
0.85 > SHAPE >= 0.4
0.4 > SHAPE
No Data

ENERGY = -181.8 Mon414

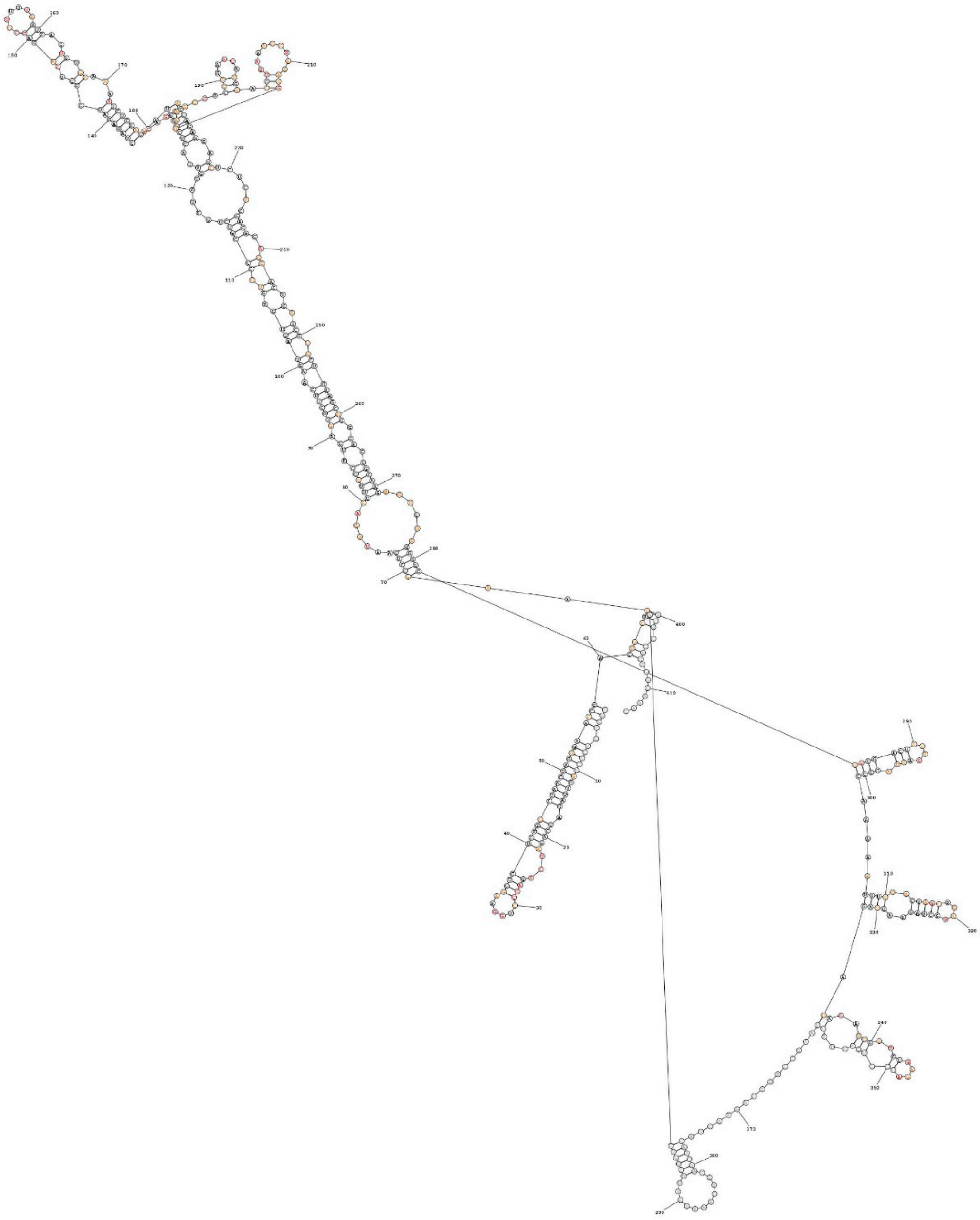

SHAPE >= 0.85

0.85 > SHAPE >= 0.4

0.4 > SHAPE

No Data

ENERGY = -181.7 Mon414

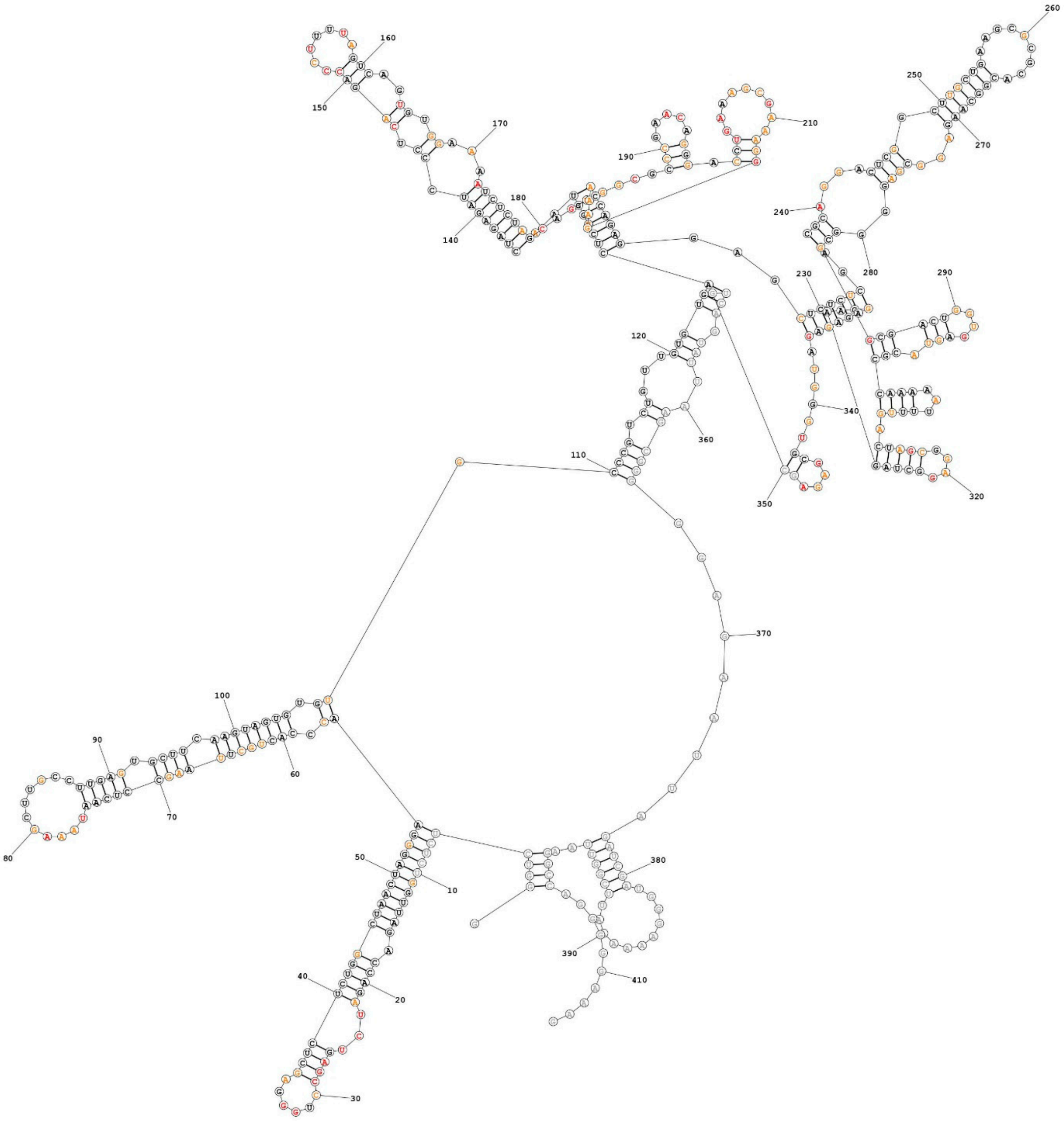

SHAPE >= 0.85
0.85 > SHAPE >= 0.4
0.4 > SHAPE
No Data

ENERGY = -181.3 Mon414

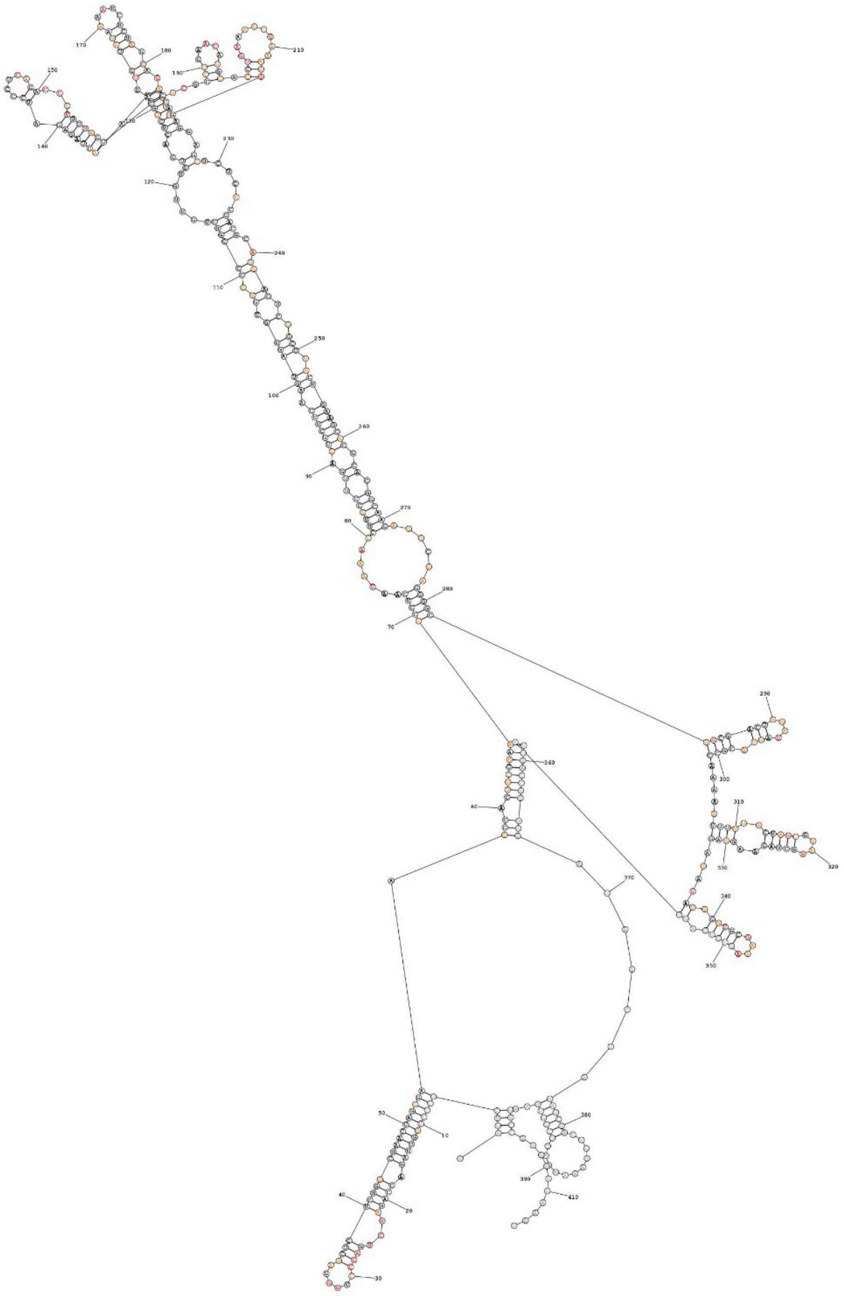

SHAPE >= 0.85
0.85 > SHAPE >= 0.4
0.4 > SHAPE
No Data

ENERGY = -181.2 Mon414

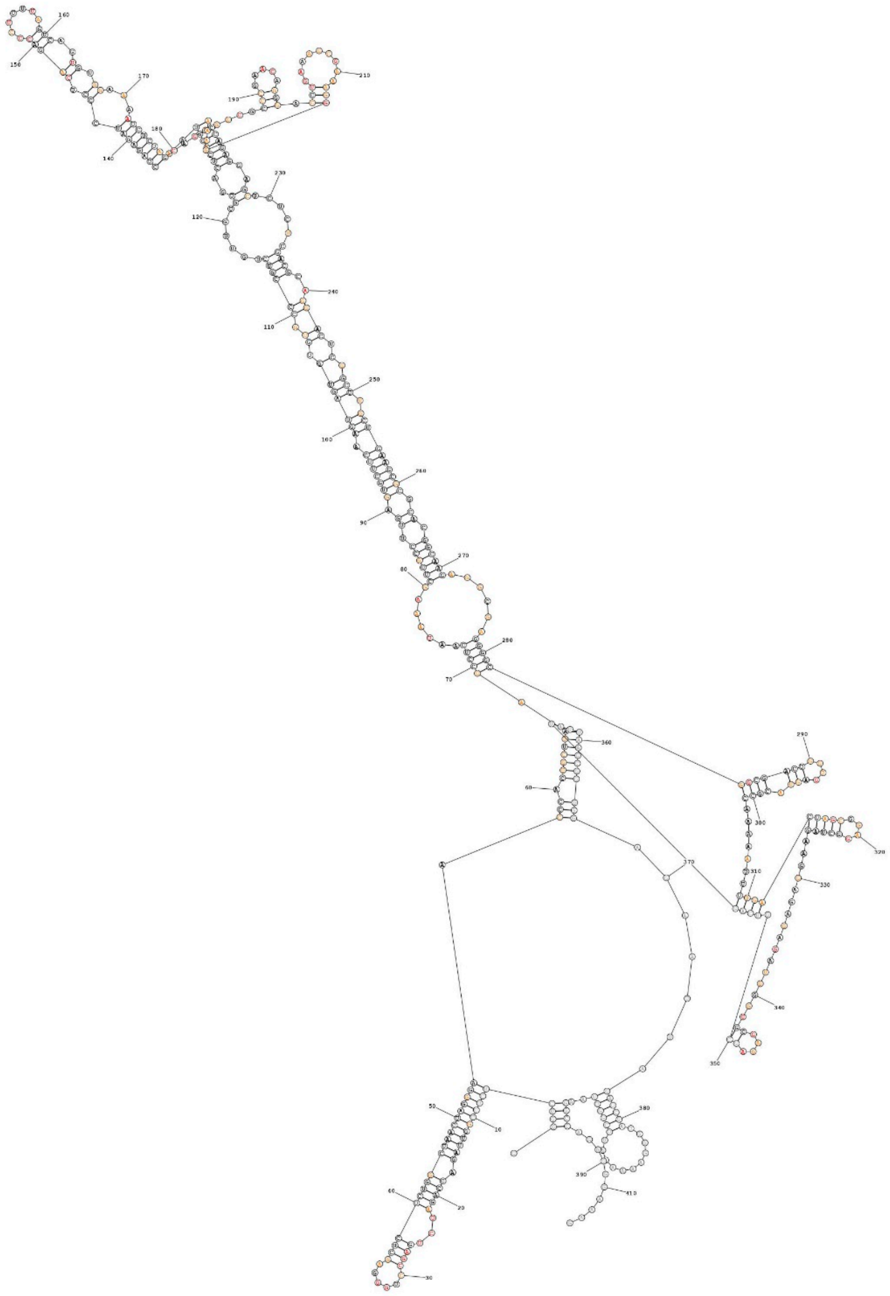

SHAPE >= 0.85
0.85 > SHAPE >= 0.4
0.4 > SHAPE
No Data

ENERGY = -181.2 Mon414

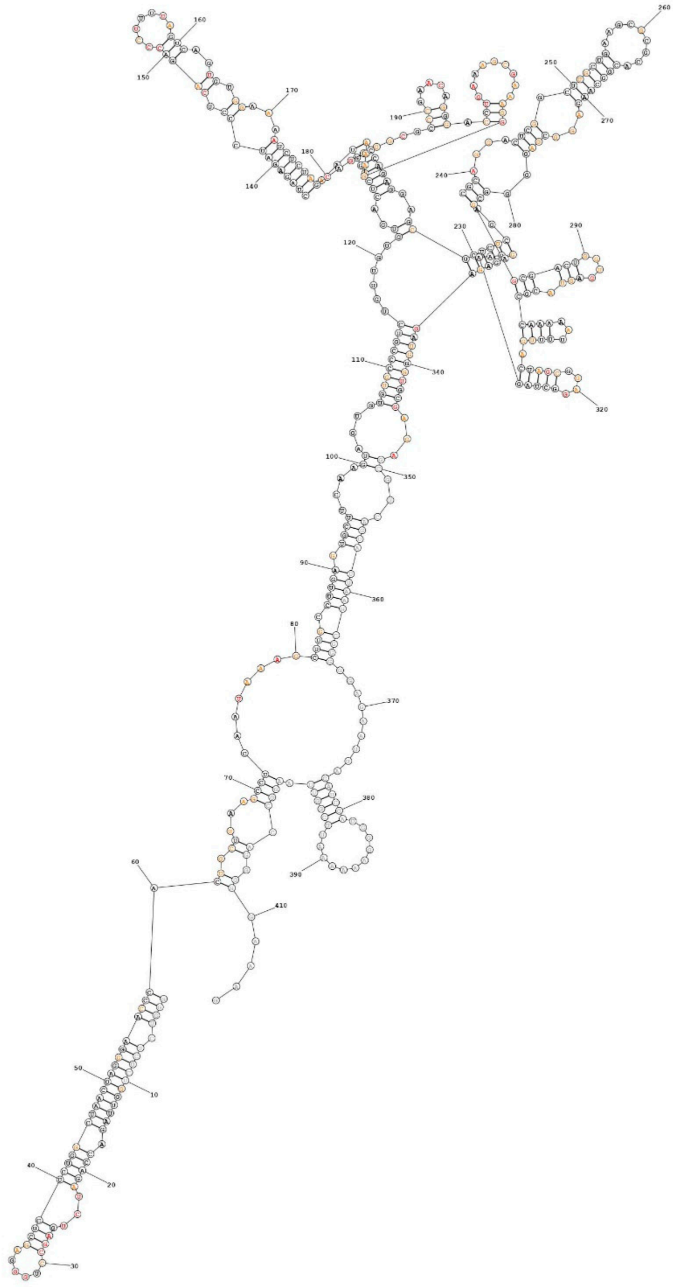

SHAPE >= 0.85
0.85 > SHAPE >= 0.4
0.4 > SHAPE
No Data

ENERGY = -180.8 Mon414

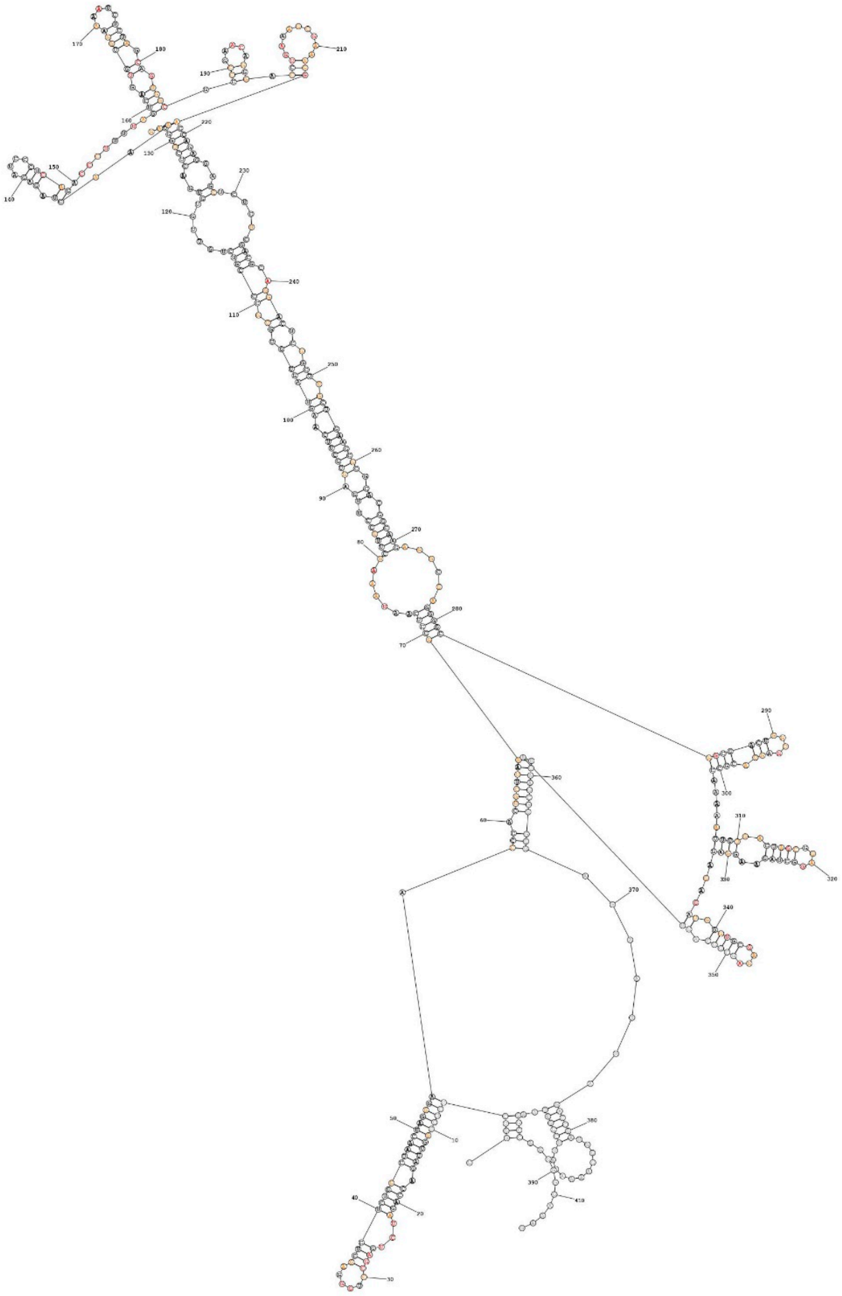

SHAPE >= 0.85
0.85 > SHAPE >= 0.4
0.4 > SHAPE
No Data

ENERGY = -180.8 Mon414

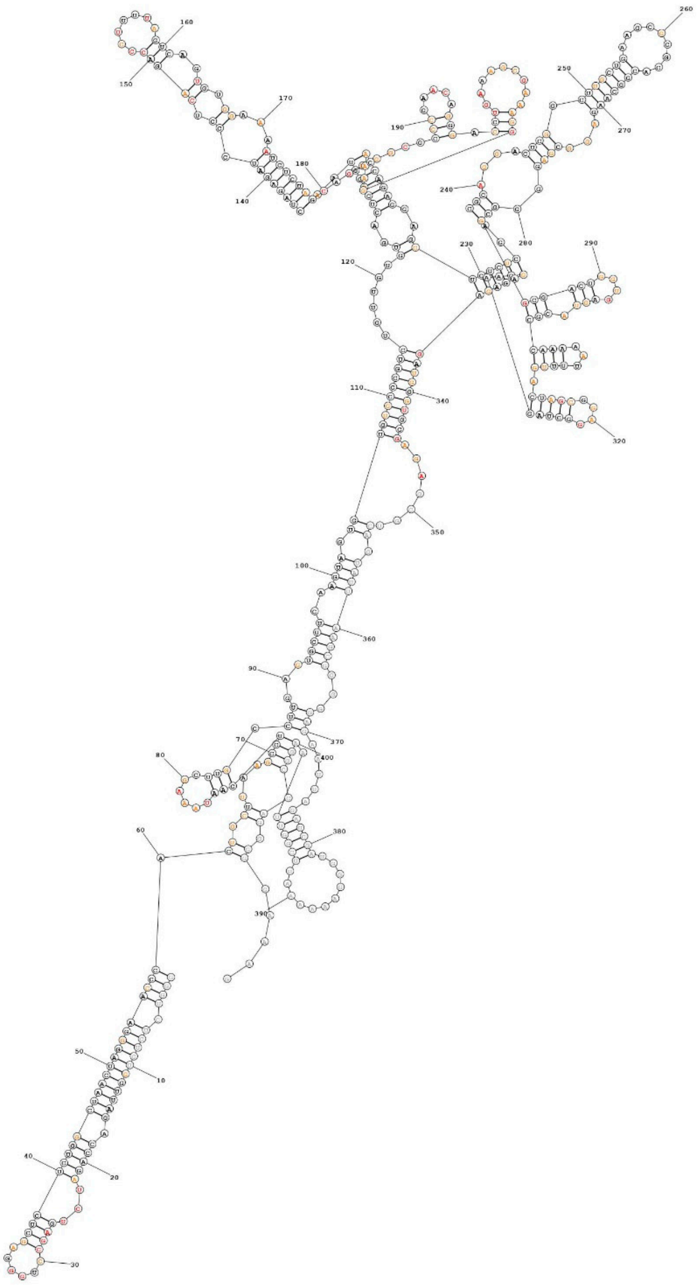

SHAPE >= 0.85
0.85 > SHAPE >= 0.4
0.4 > SHAPE
No Data

ENERGY = -180.6 Mon414

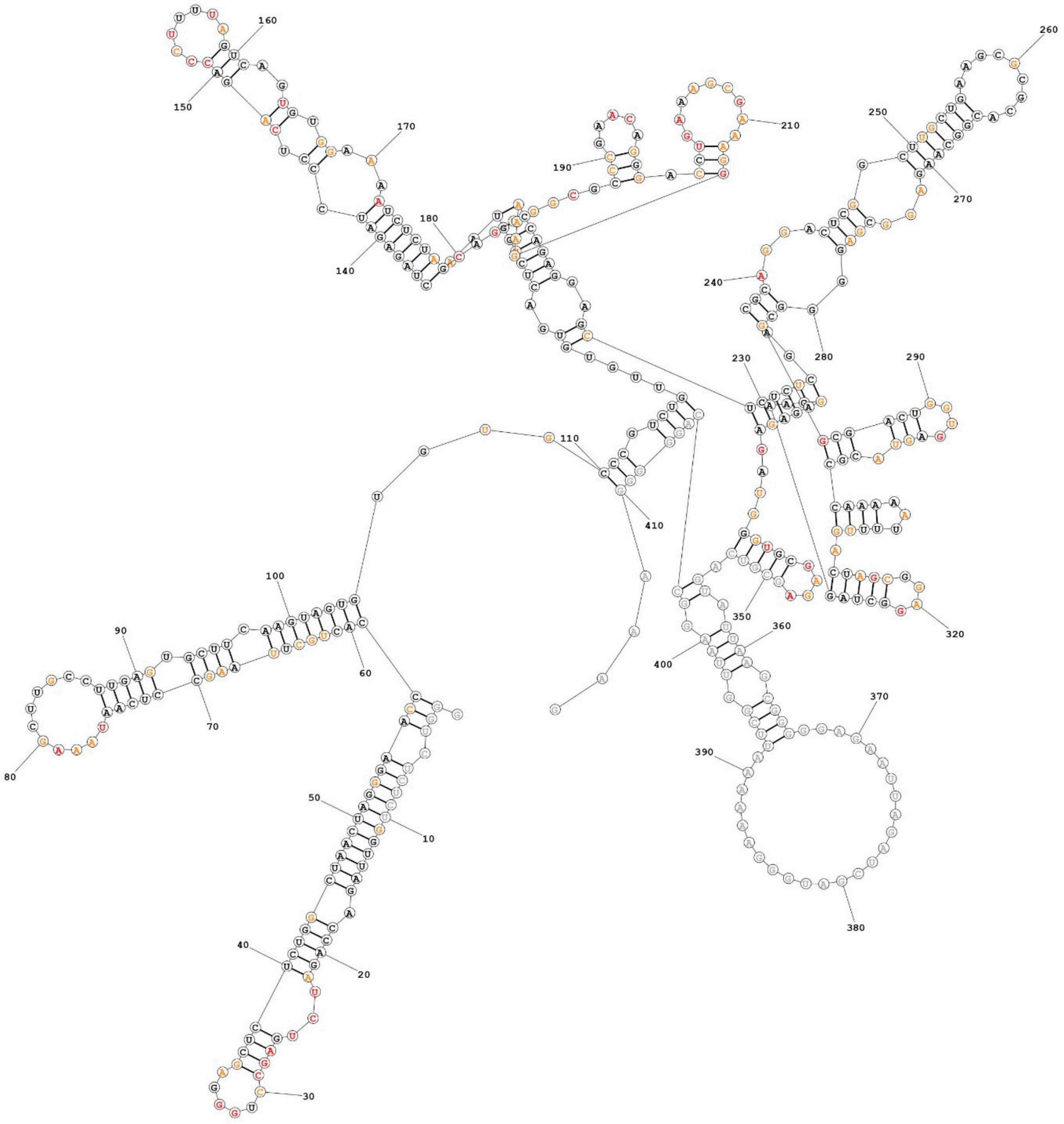

SHAPE >= 0.85

0.85 > SHAPE >= 0.4

0.4 > SHAPE

No Data

ENERGY = -180.6 Mon414

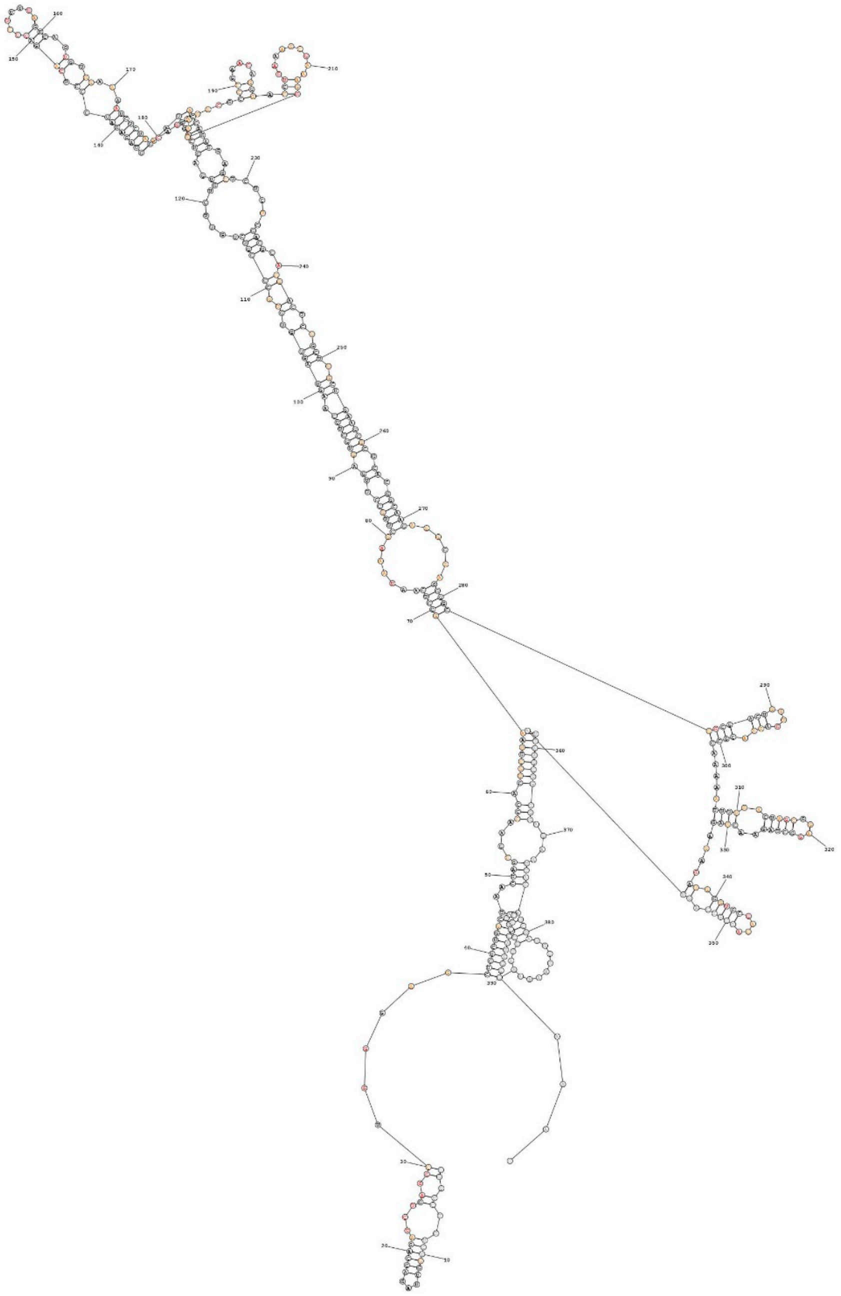

SHAPE >= 0.85

0.85 > SHAPE >= 0.4

0.4 > SHAPE

No Data

ENERGY = -180.5 Mon414

Supplementary figure 4 - Monomer 414 + Gag

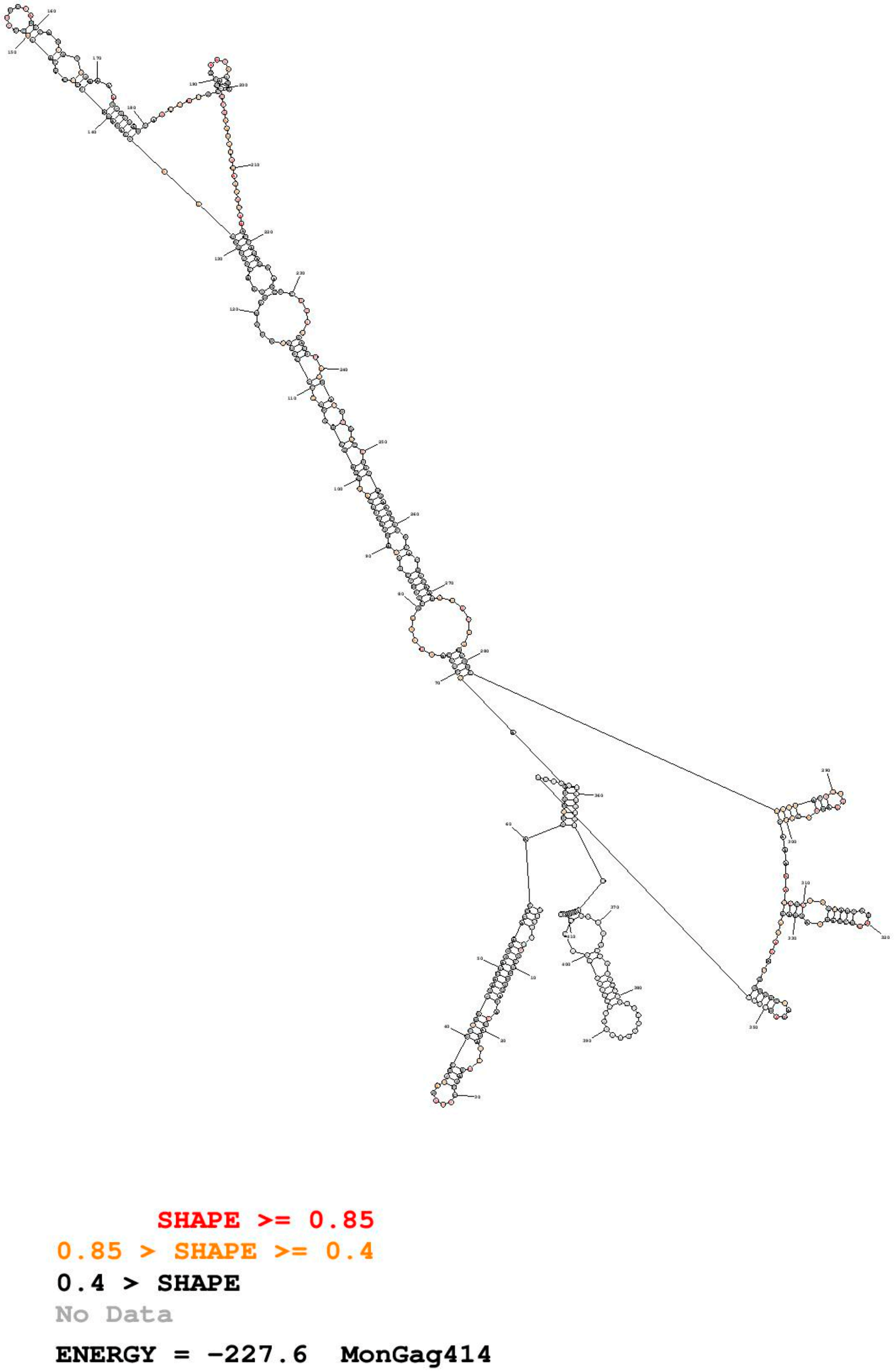

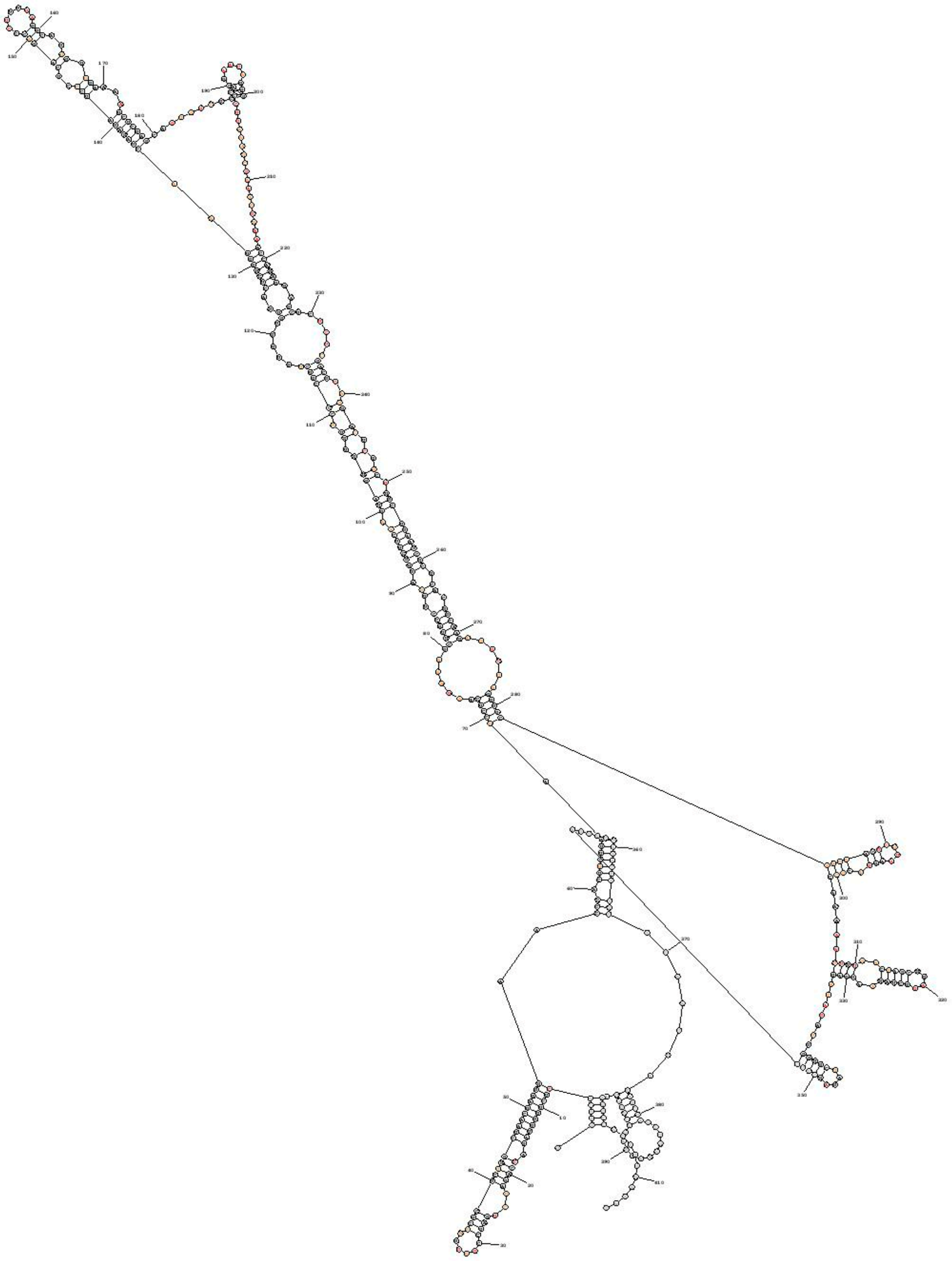

SHAPE >= 0.85
0.85 > SHAPE >= 0.4
0.4 > SHAPE
No Data

ENERGY = -227.5 MonGag414

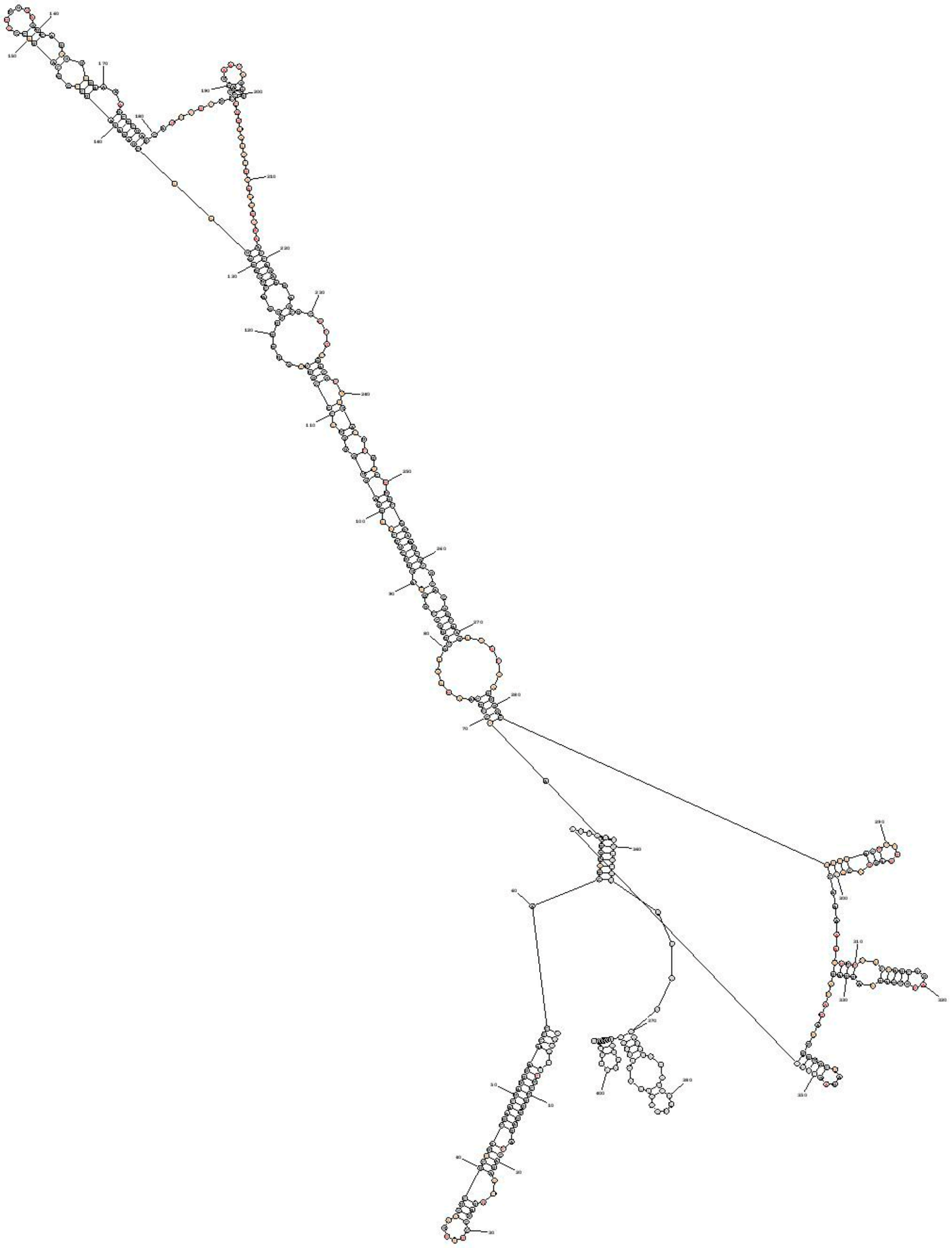

SHAPE >= 0.85
0.85 > SHAPE >= 0.4
0.4 > SHAPE
No Data

ENERGY = −226.8 MonGag414

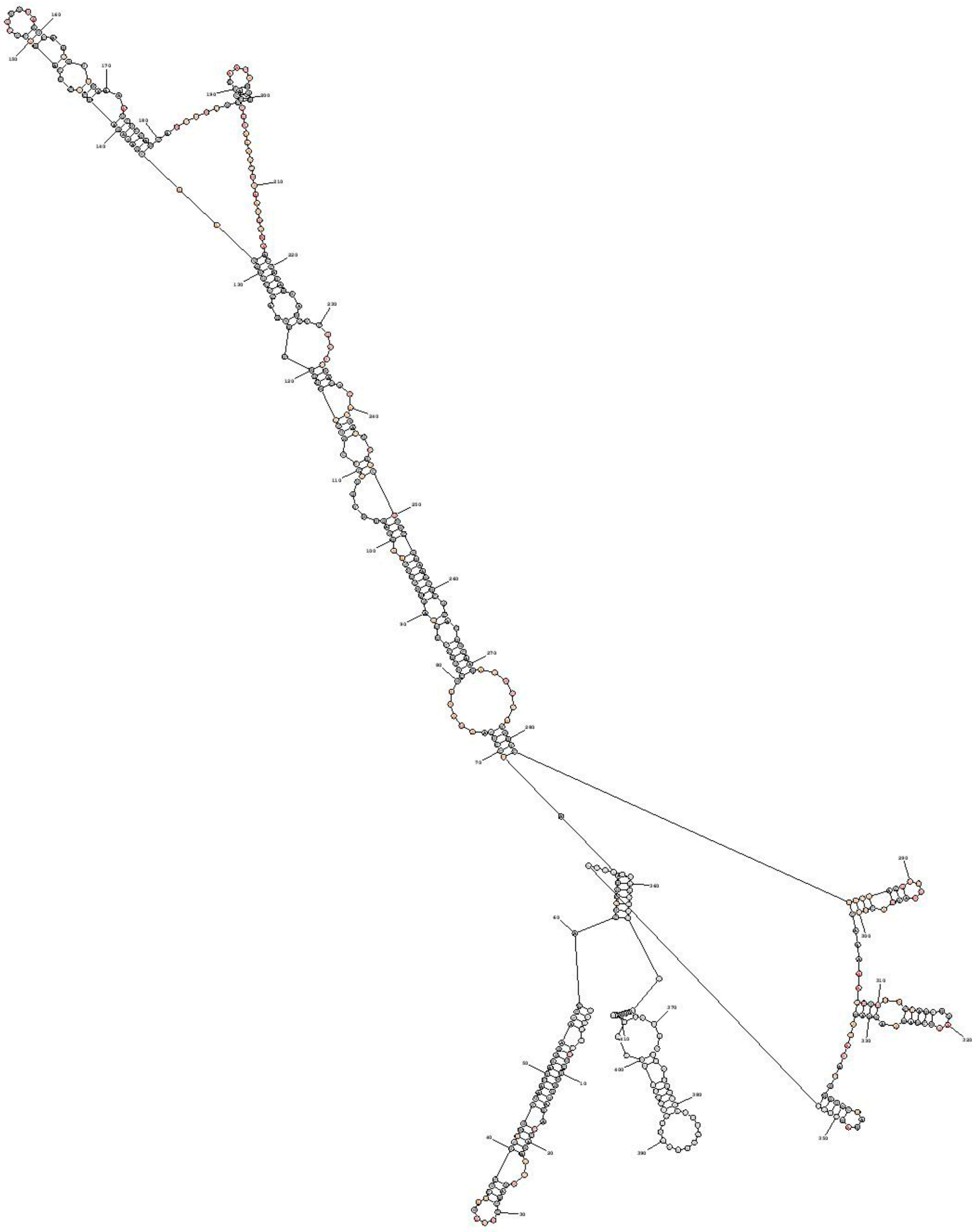

SHAPE >= 0.85

0.85 > SHAPE >= 0.4

0.4 > SHAPE

No Data

ENERGY = −226.3   MonGag414

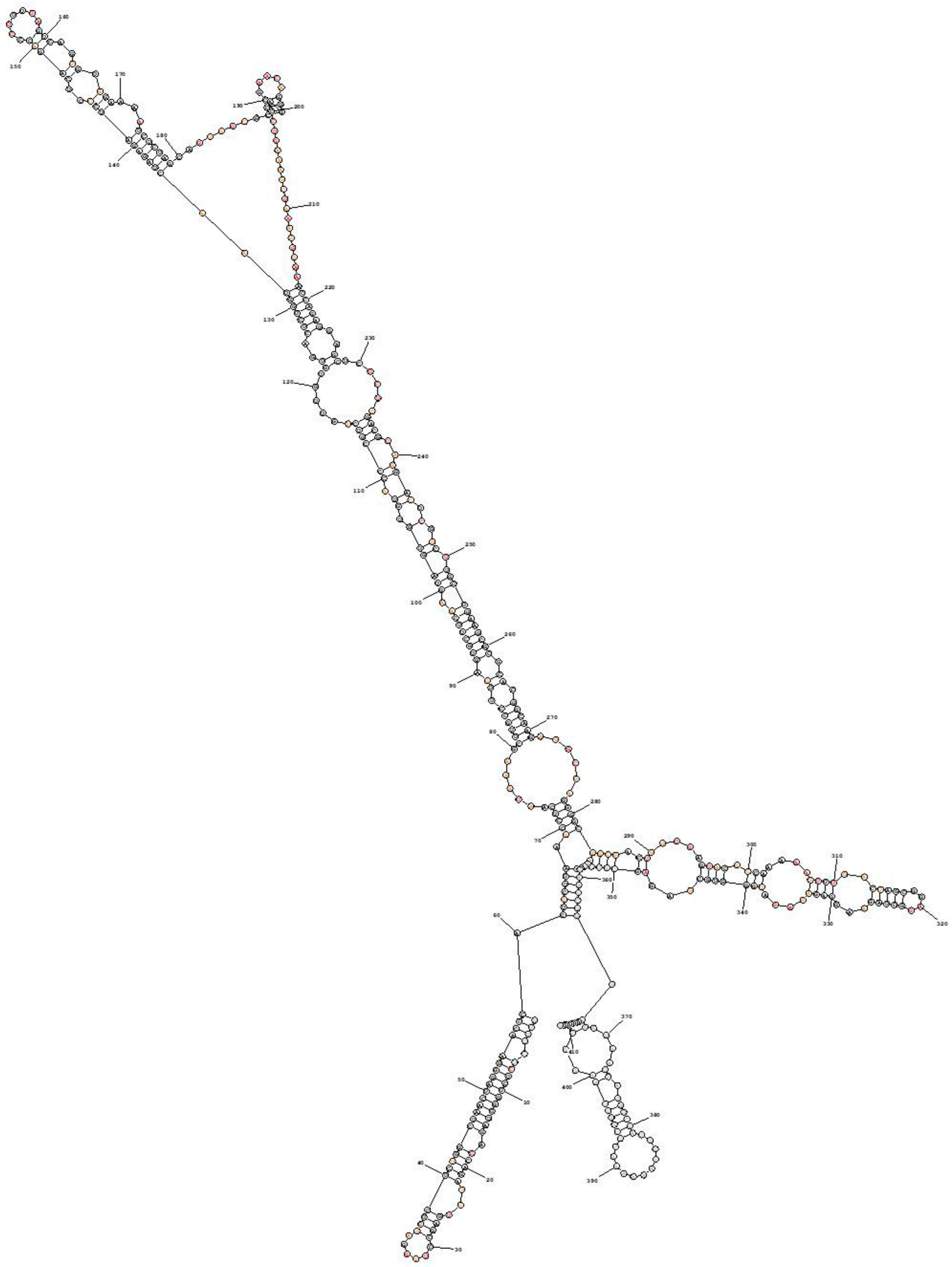

SHAPE >= 0.85

0.85 > SHAPE >= 0.4

0.4 > SHAPE

No Data

ENERGY = −225.4 MonGag414

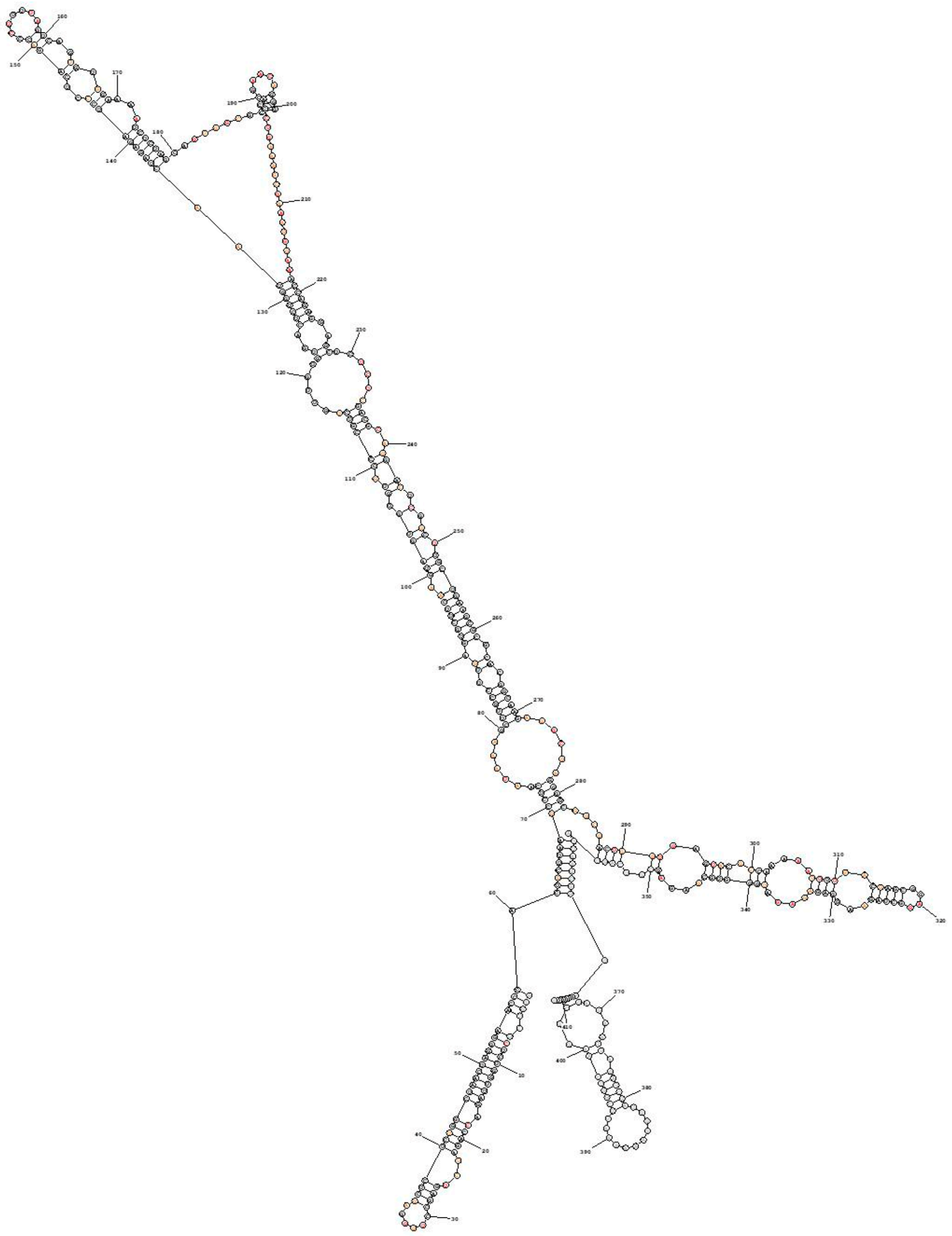

SHAPE >= 0.85
0.85 > SHAPE >= 0.4
0.4 > SHAPE
No Data

ENERGY = -224.9 MonGag414

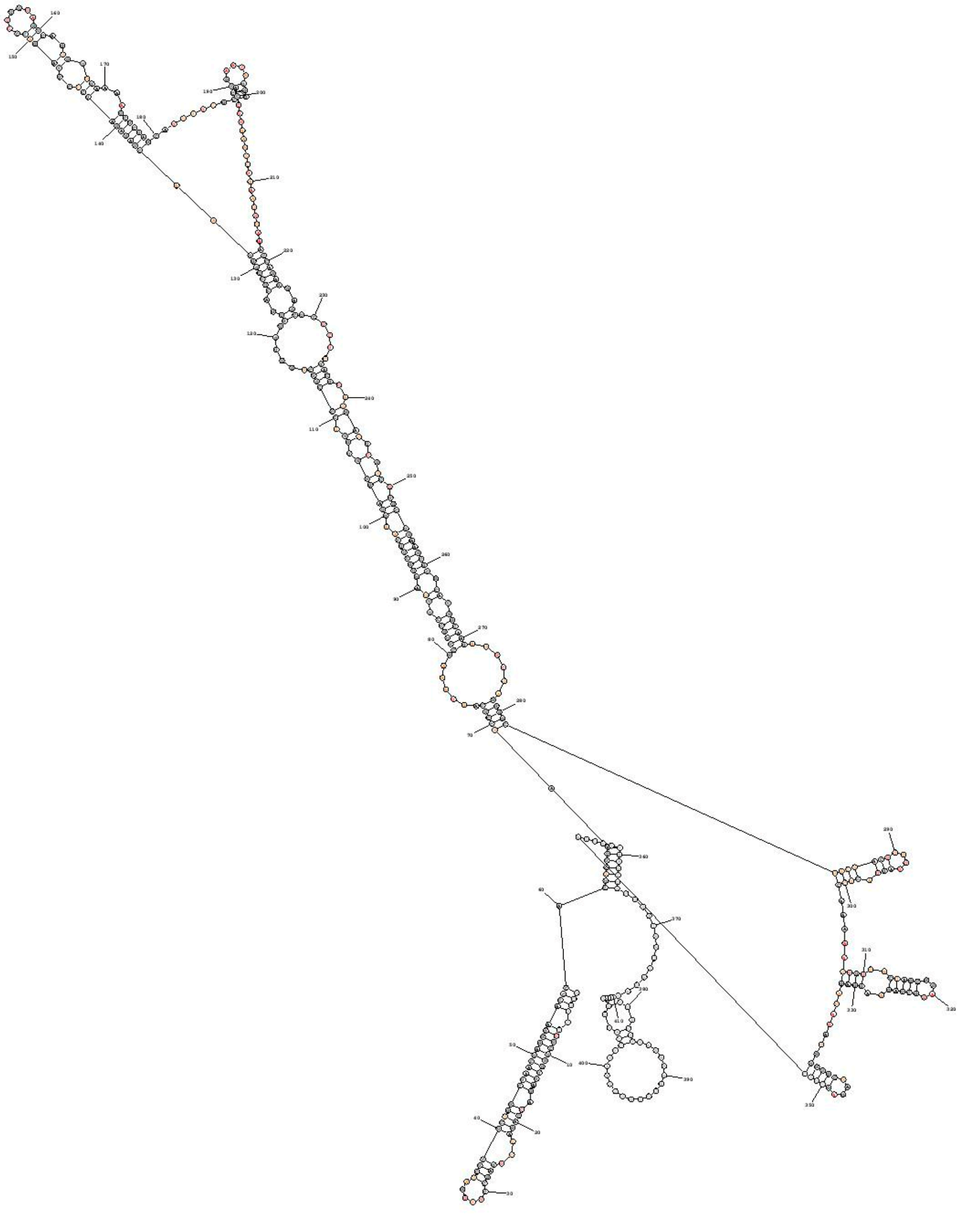

SHAPE >= 0.85
0.85 > SHAPE >= 0.4
0.4 > SHAPE
No Data

ENERGY = −224.8 MonGag414

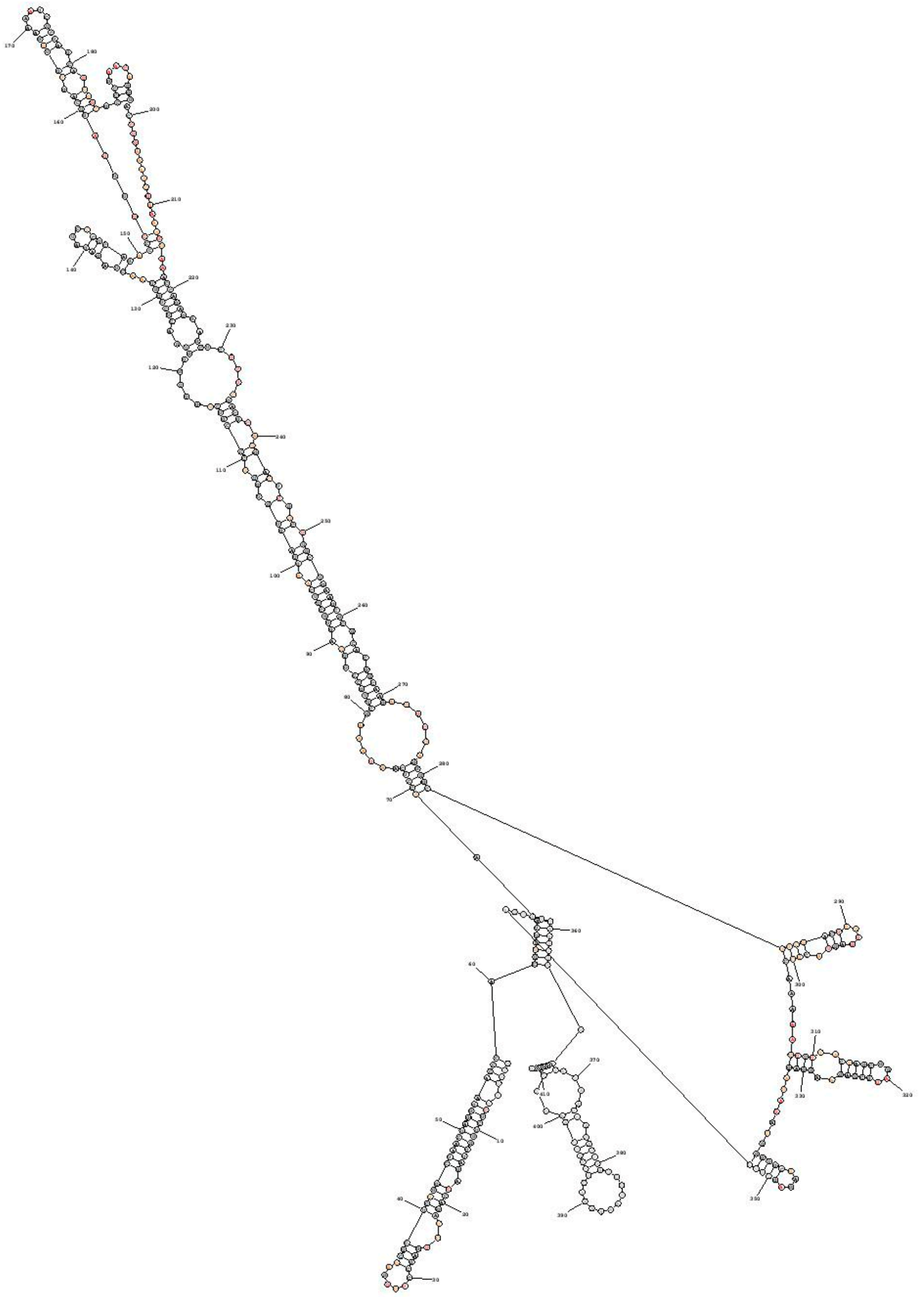

SHAPE >= 0.85
0.85 > SHAPE >= 0.4
0.4 > SHAPE
No Data

ENERGY = −223.9 MonGag414

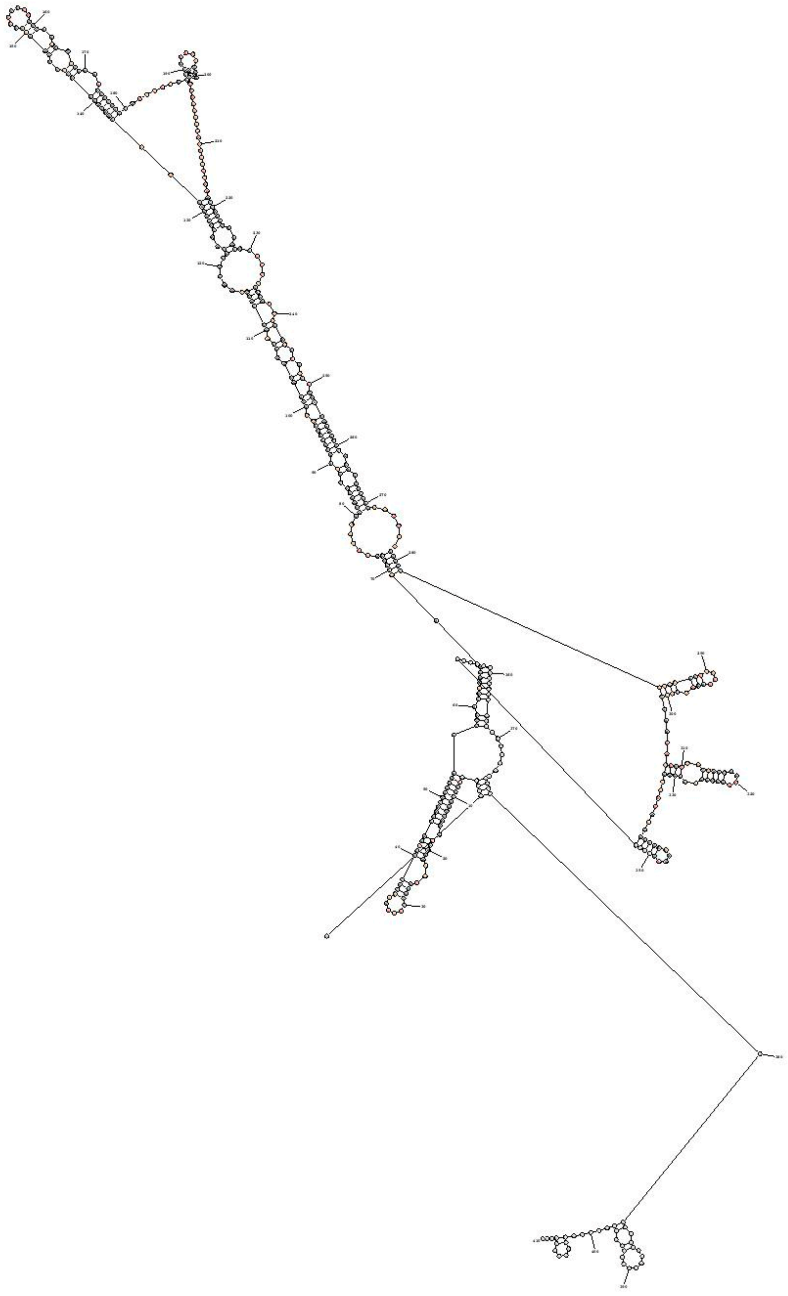

SHAPE >= 0.85
0.85 > SHAPE >= 0.4
0.4 > SHAPE
No Data

ENERGY = -223.8   MonGag414

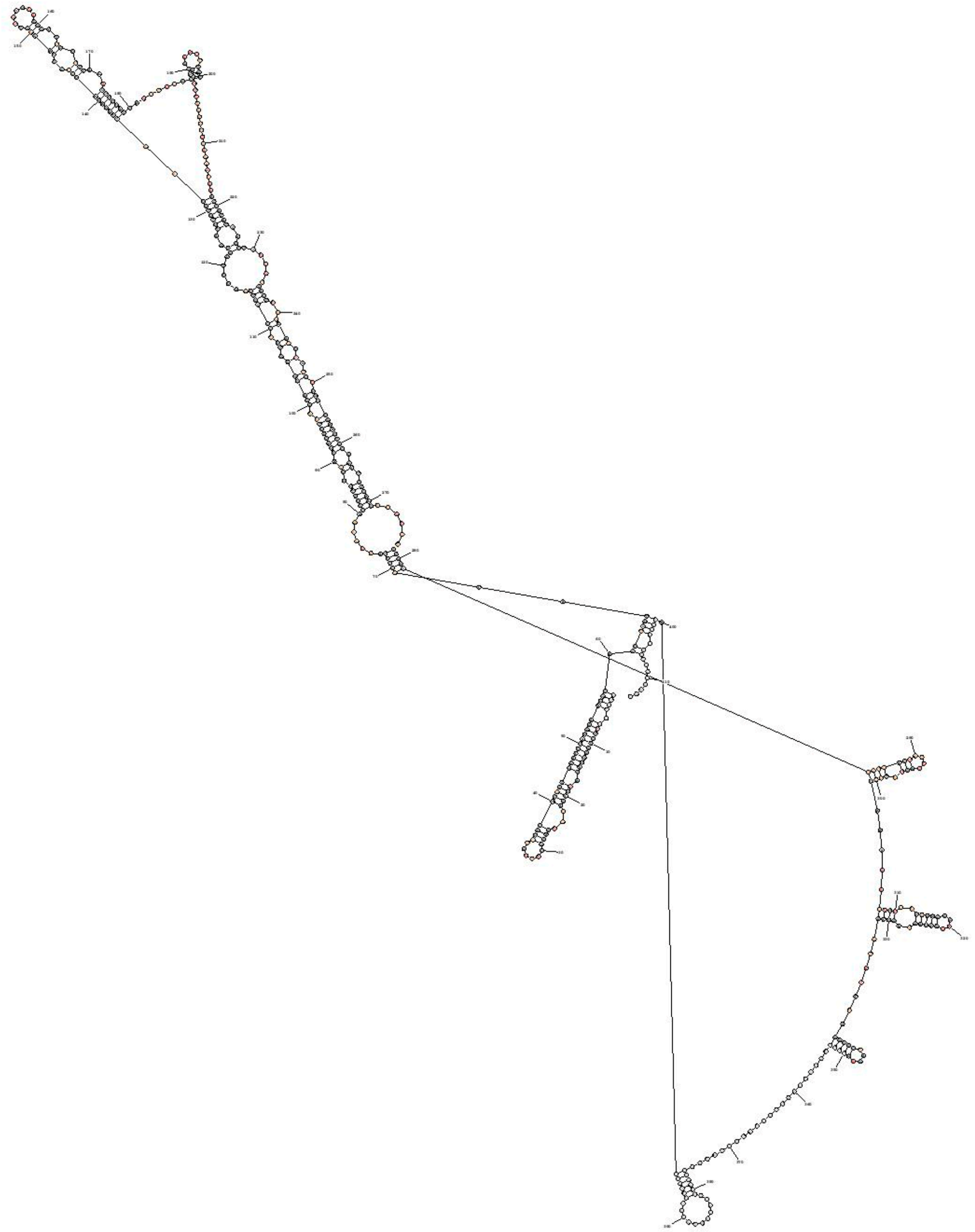

SHAPE >= 0.85
0.85 > SHAPE >= 0.4
0.4 > SHAPE
No Data

ENERGY = -223.4 MonGag414

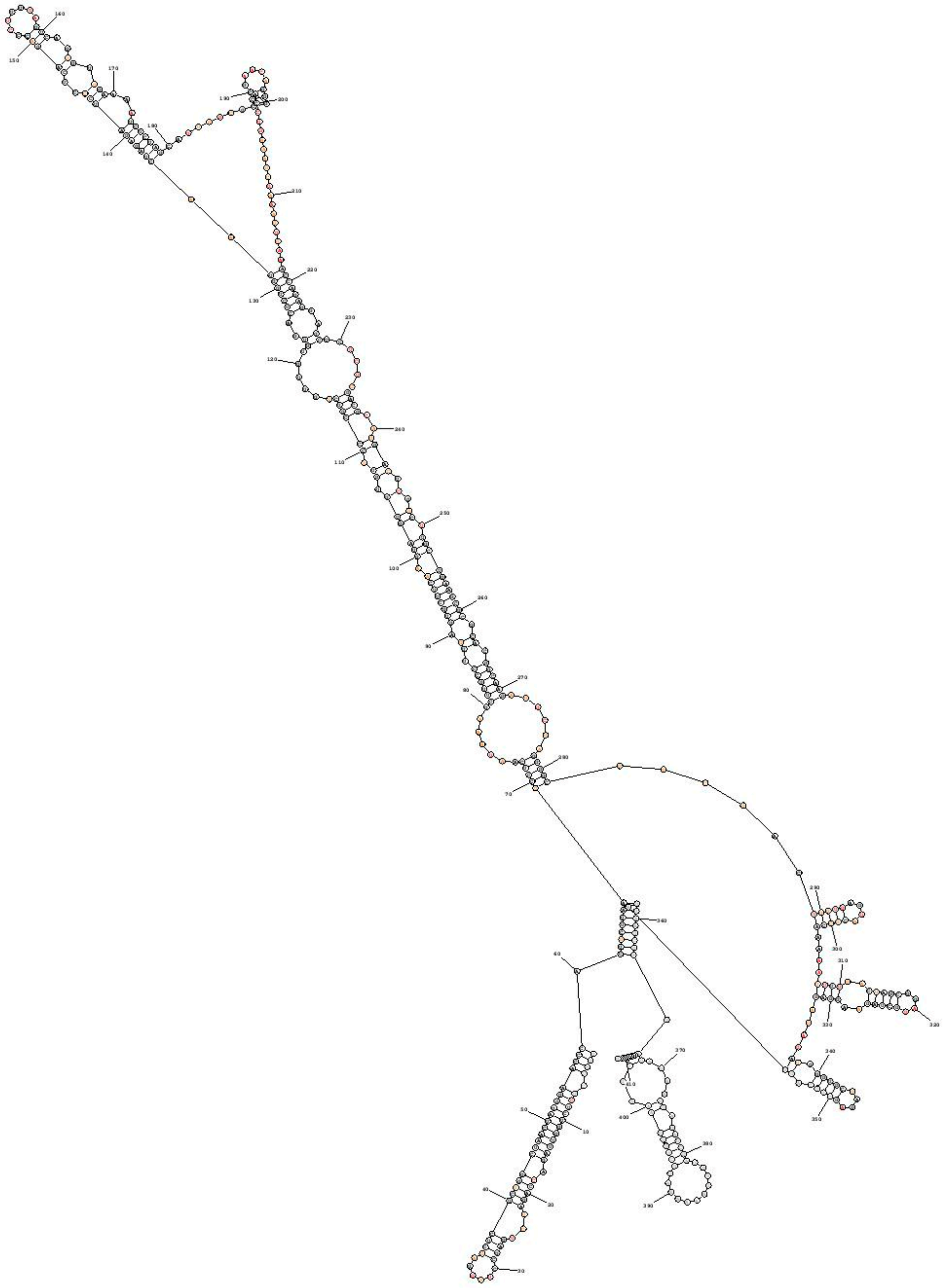

SHAPE >= 0.85
0.85 > SHAPE >= 0.4
0.4 > SHAPE
No Data

ENERGY = −223.3 MonGag414

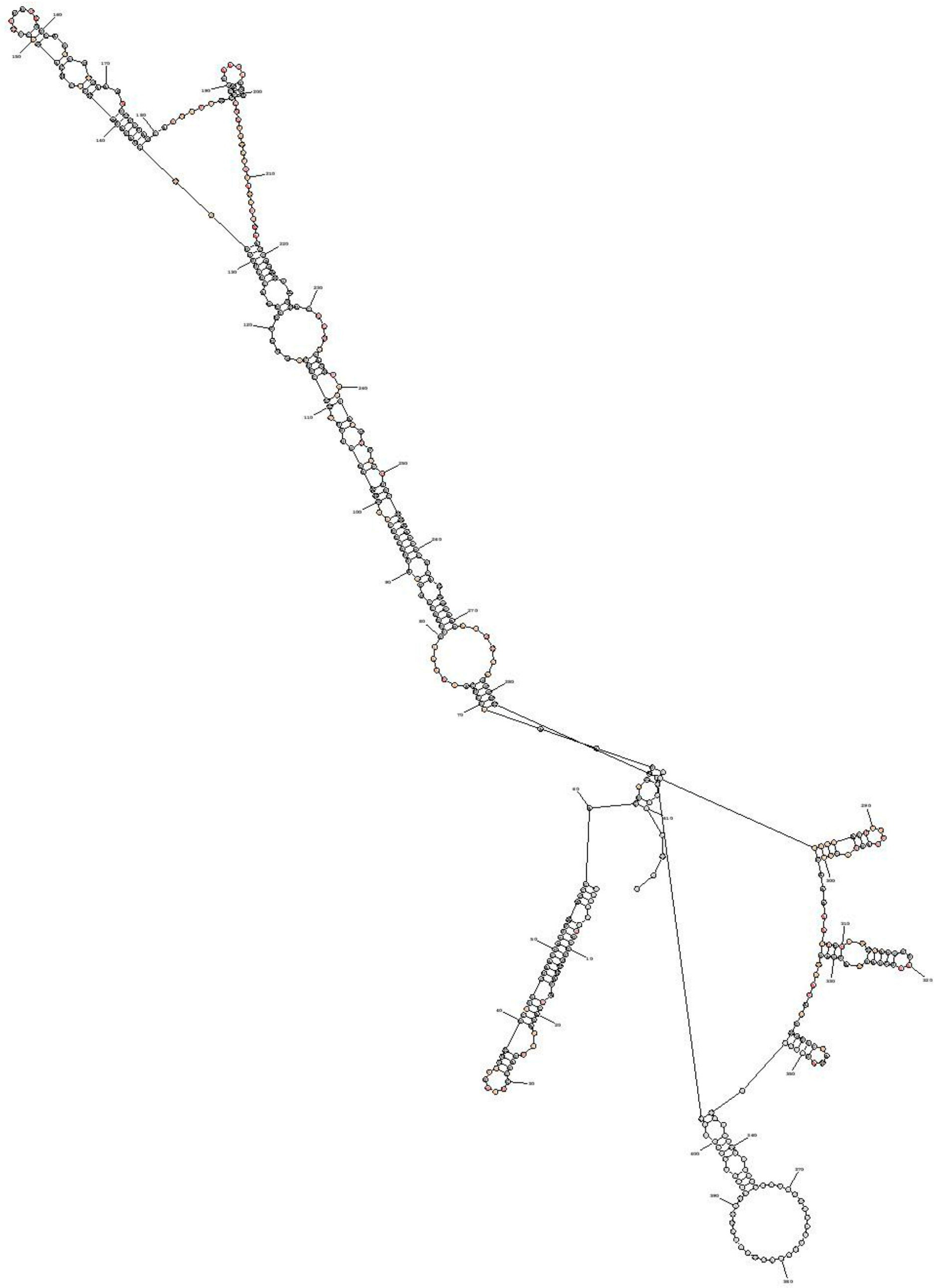

SHAPE >= 0.85
0.85 > SHAPE >= 0.4
0.4 > SHAPE
No Data

ENERGY = −223.2 MonGag414

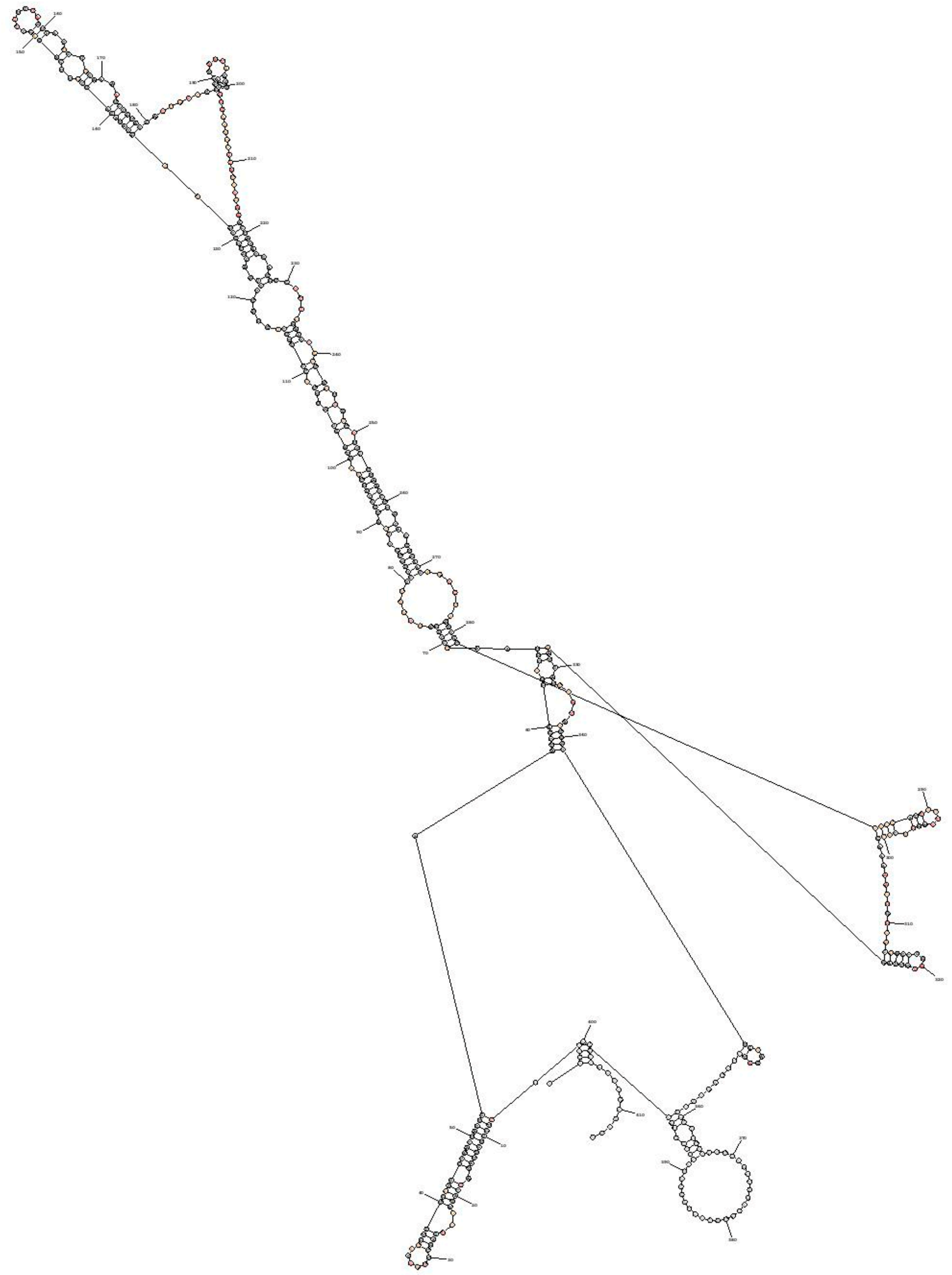

SHAPE >= 0.85
0.85 > SHAPE >= 0.4
0.4 > SHAPE
No Data

ENERGY = −221.6 MonGag414

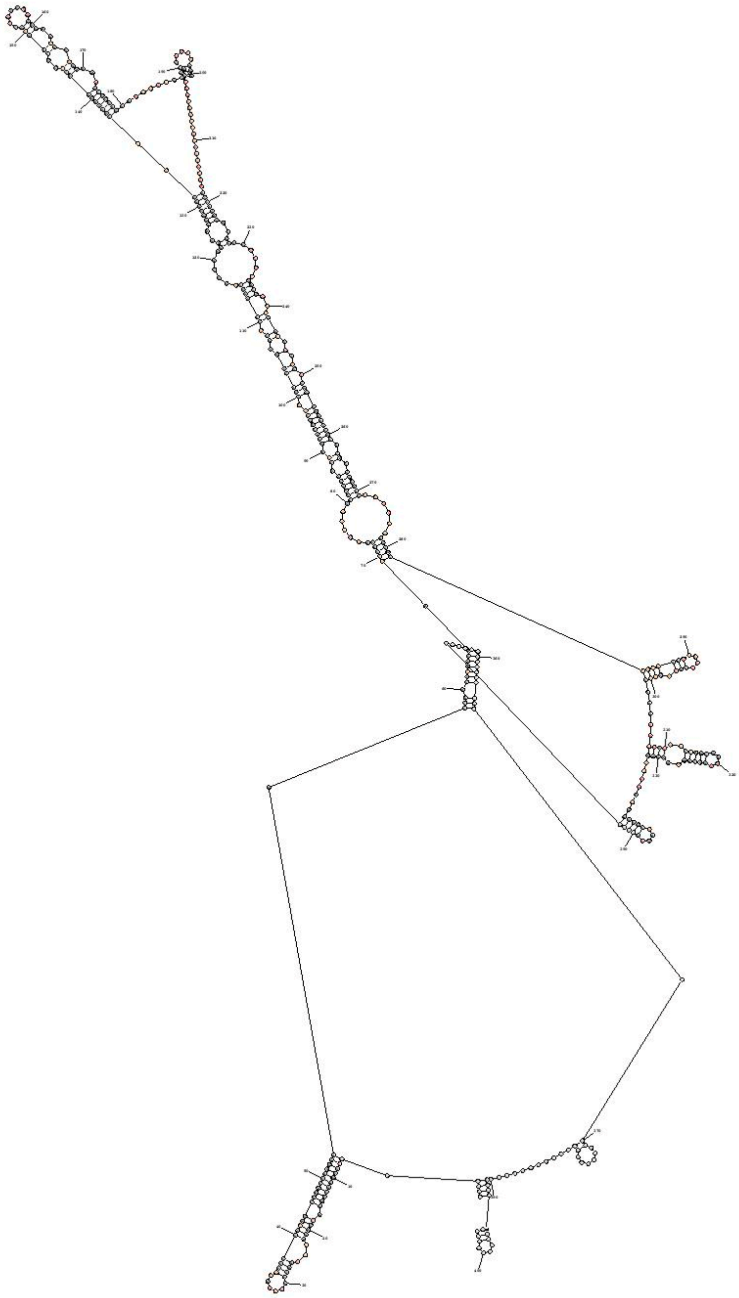

SHAPE >= 0.85
0.85 > SHAPE >= 0.4
0.4 > SHAPE
No Data

ENERGY = −220.7 MonGag414

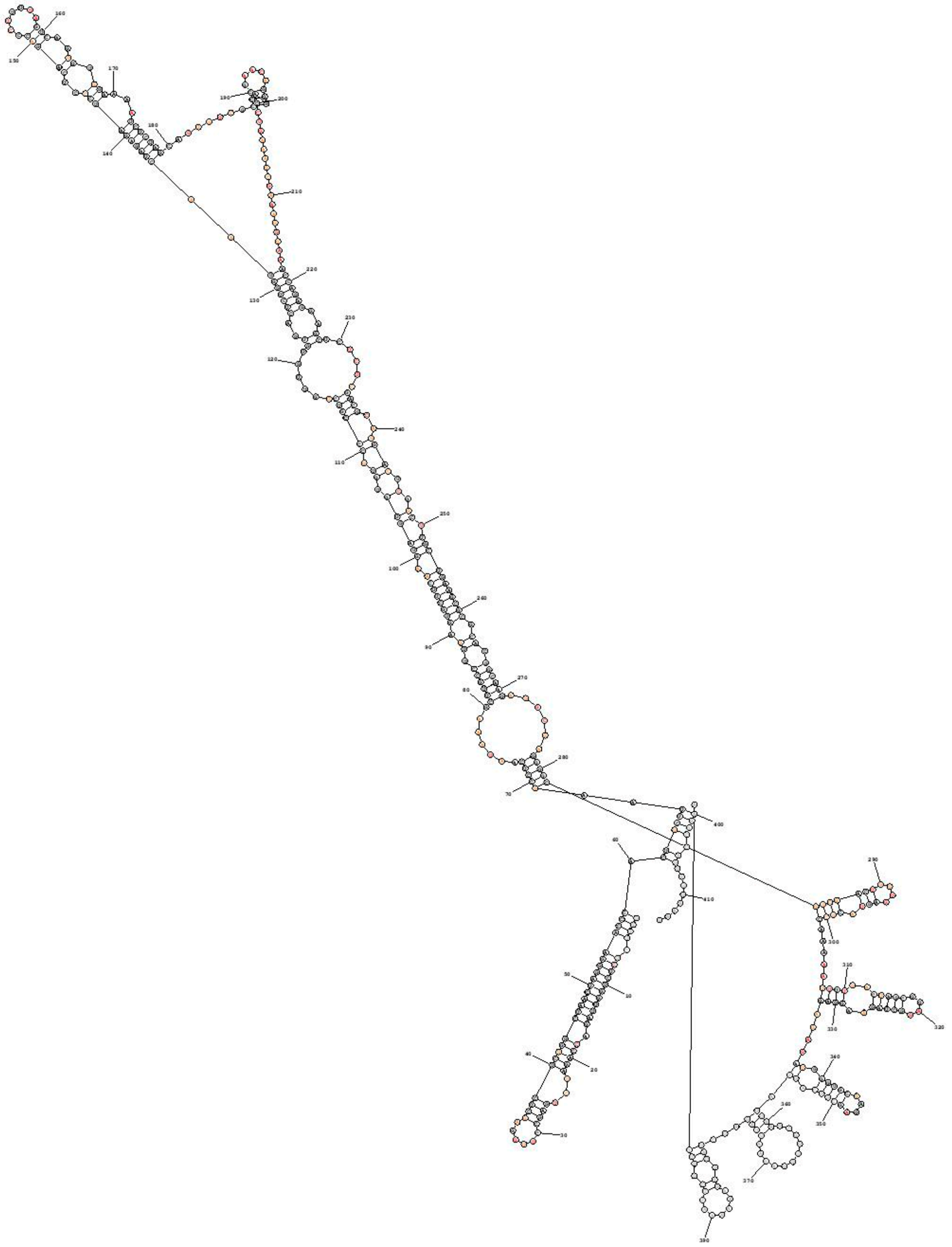

SHAPE >= 0.85
0.85 > SHAPE >= 0.4
0.4 > SHAPE
No Data

ENERGY = −220.7 MonGag414

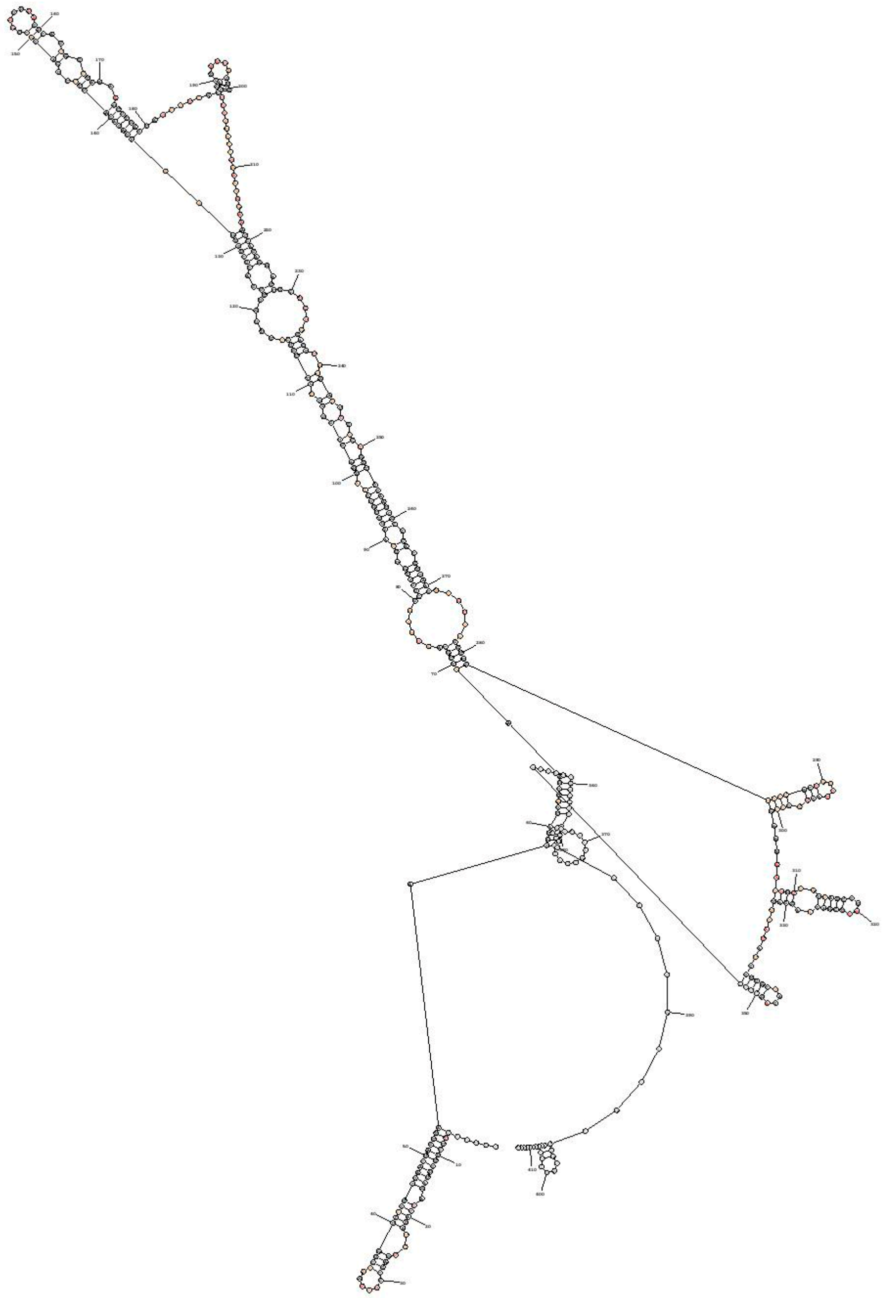

SHAPE >= 0.85
0.85 > SHAPE >= 0.4
0.4 > SHAPE
No Data

ENERGY = -220.5 MonGag414

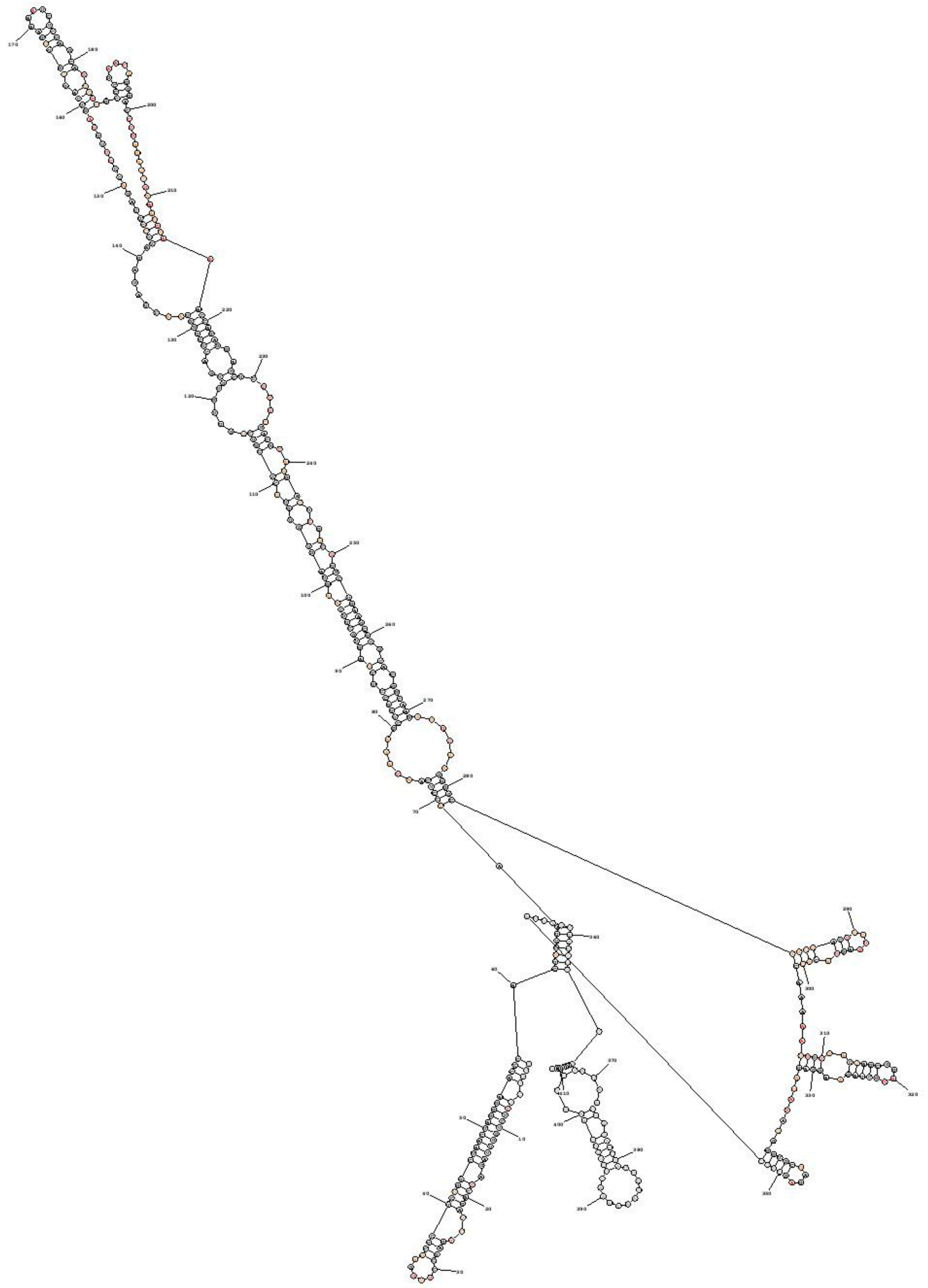

SHAPE >= 0.85
0.85 > SHAPE >= 0.4
0.4 > SHAPE
No Data

ENERGY = -220.0 MonGag414

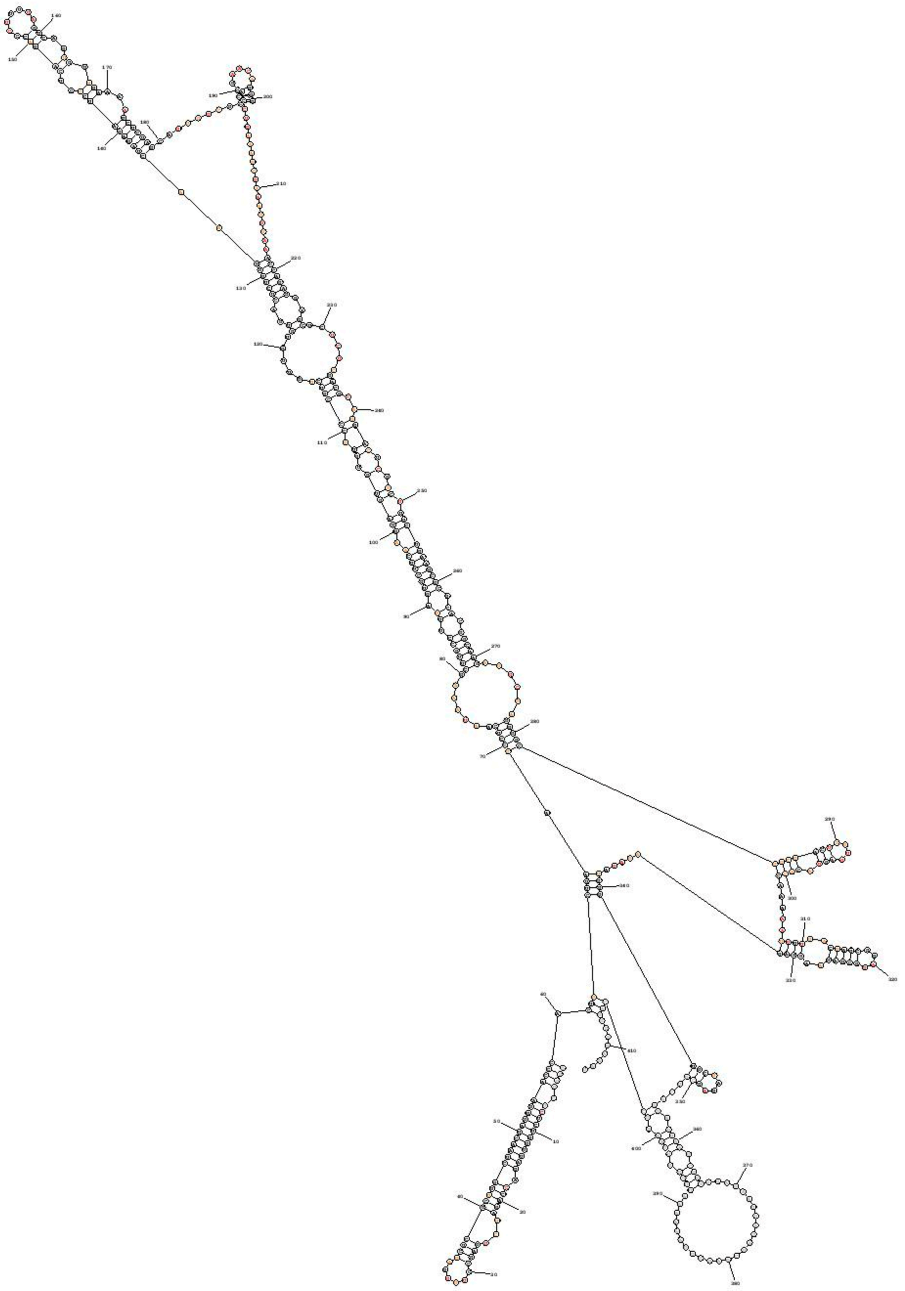

SHAPE >= 0.85
0.85 > SHAPE >= 0.4
0.4 > SHAPE
No Data

ENERGY = −219.7 MonGag414

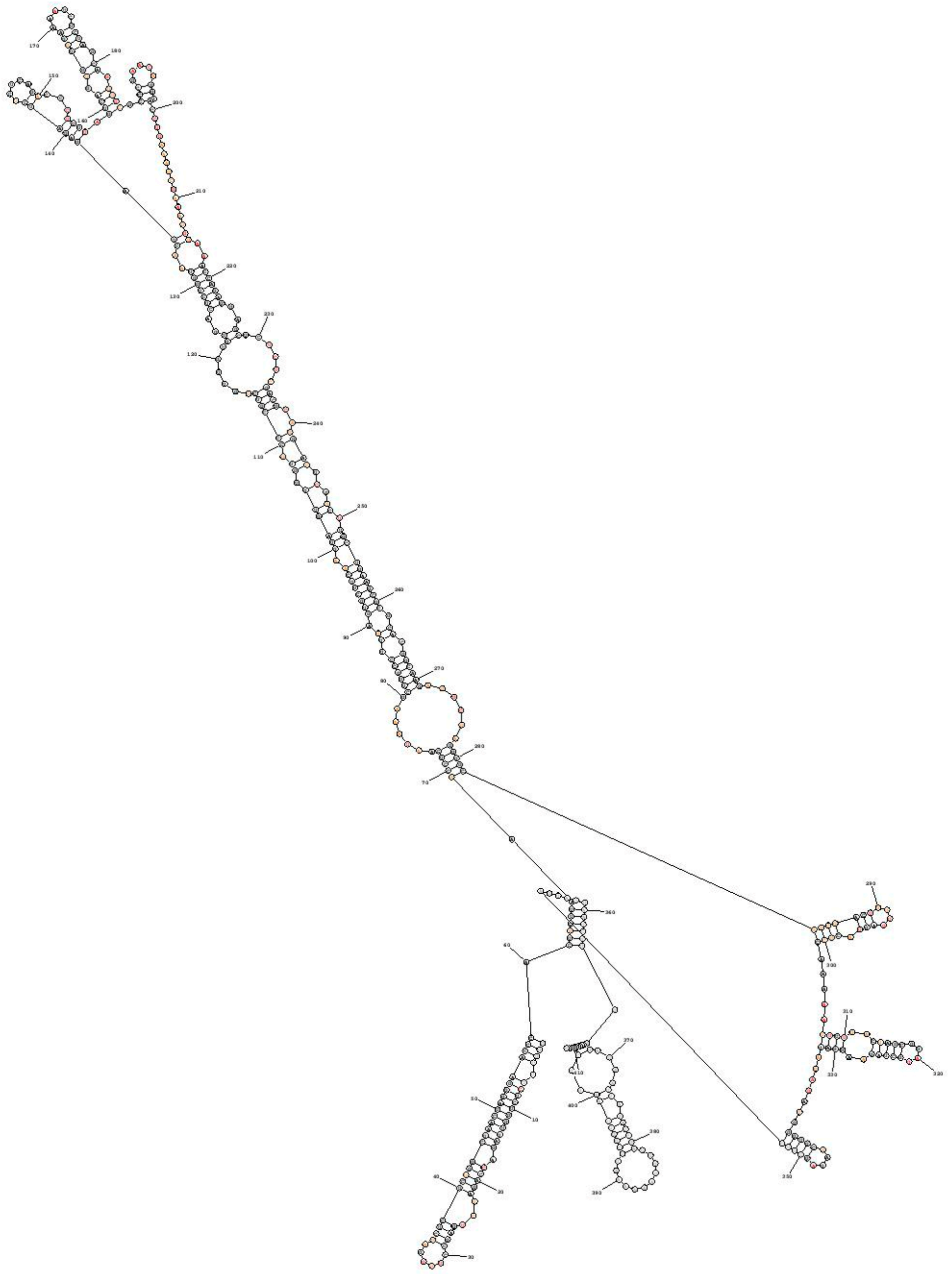

SHAPE >= 0.85
0.85 > SHAPE >= 0.4
0.4 > SHAPE
No Data

ENERGY = -219.5 MonGag414

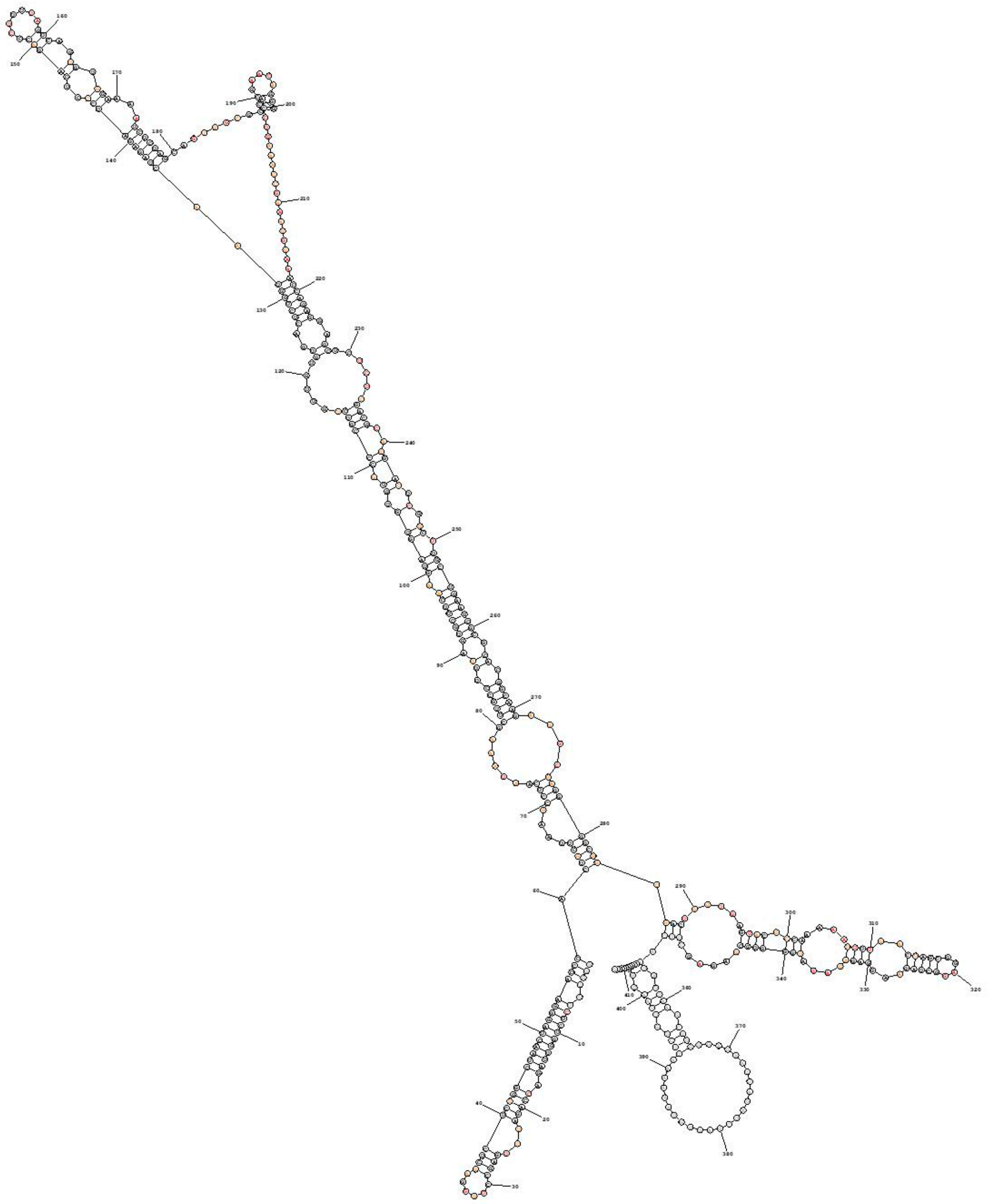

SHAPE >= 0.85
0.85 > SHAPE >= 0.4
0.4 > SHAPE
No Data

ENERGY = −219.4 MonGag414

Supplementary figure 5 - Monomer 414 + NC

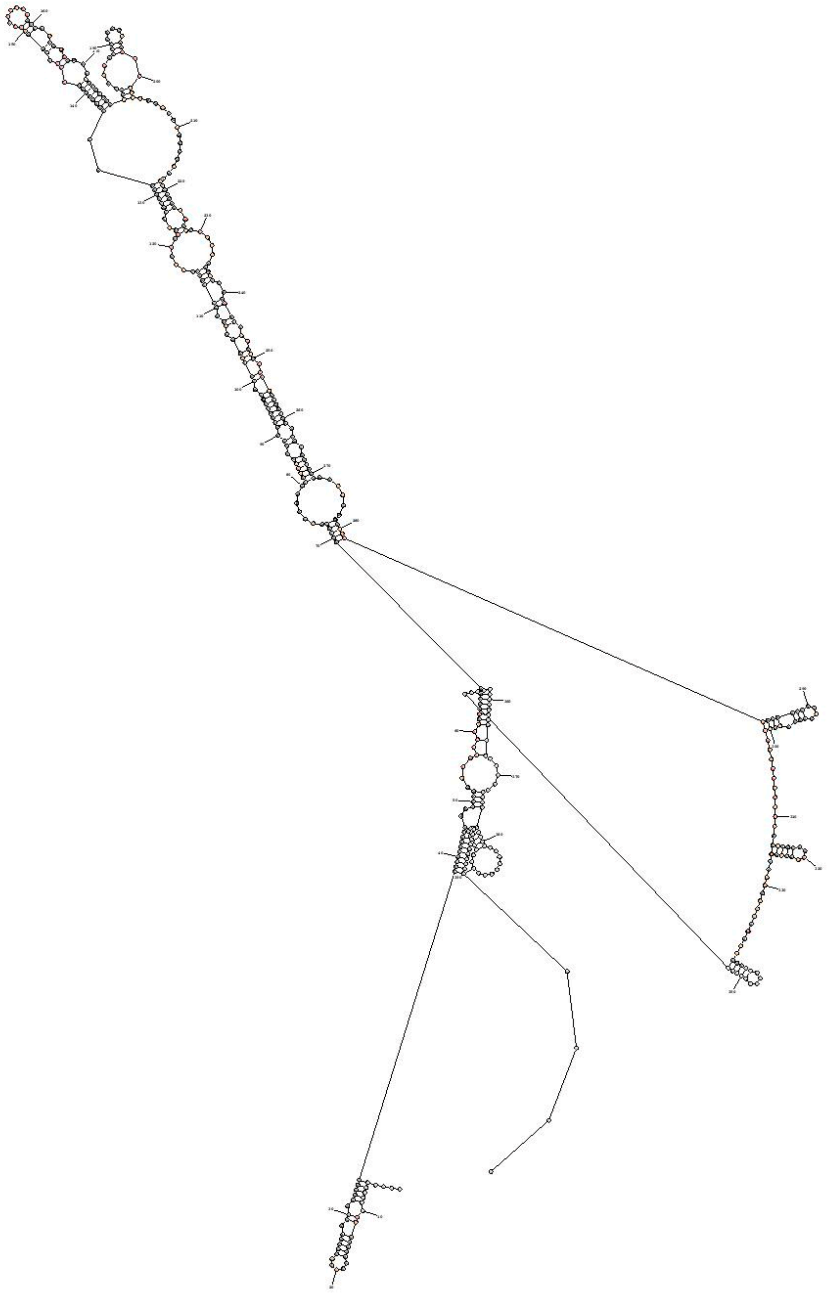

SHAPE >= 0.85

0.85 > SHAPE >= 0.4

0.4 > SHAPE

No Data

ENERGY = -208.3 MonNC414

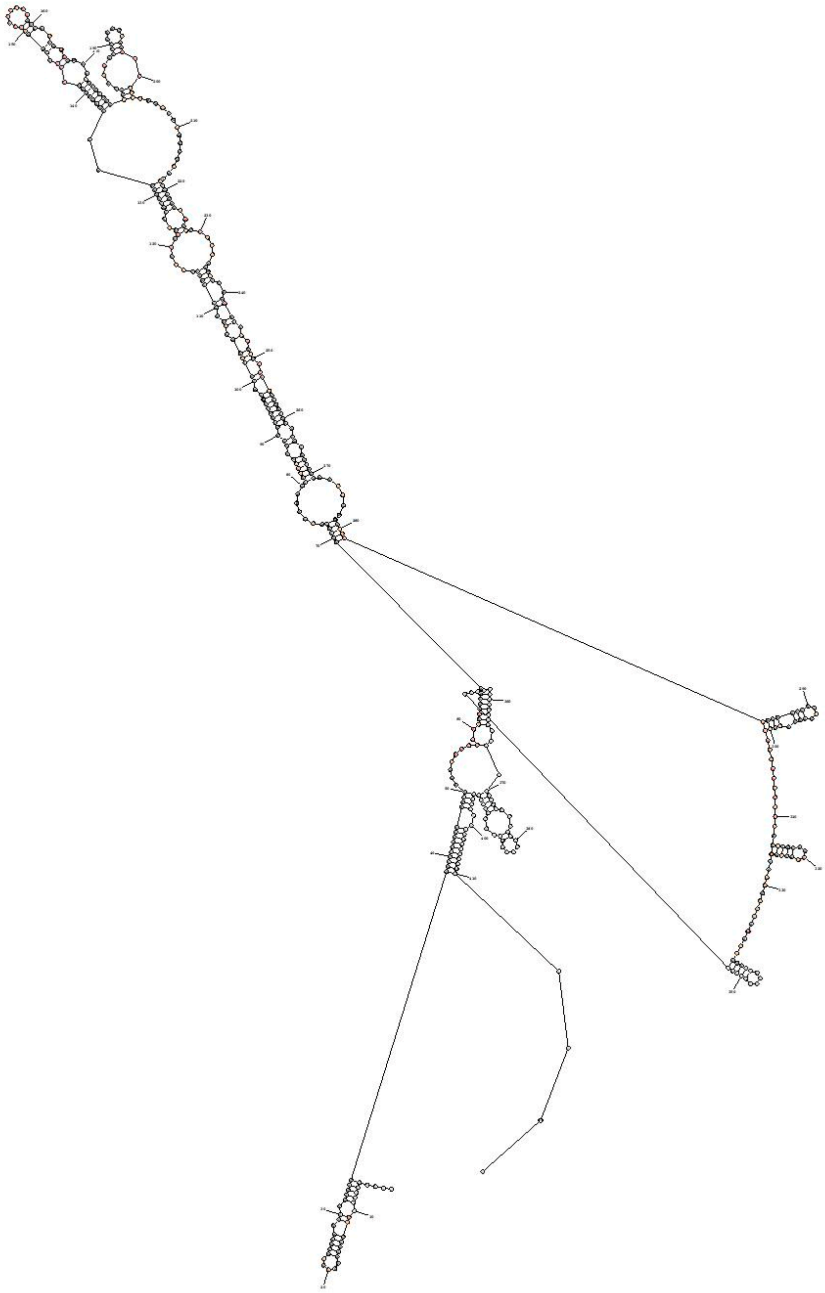

SHAPE >= 0.85
0.85 > SHAPE >= 0.4
0.4 > SHAPE
No Data

ENERGY = -207.1 MonNC414

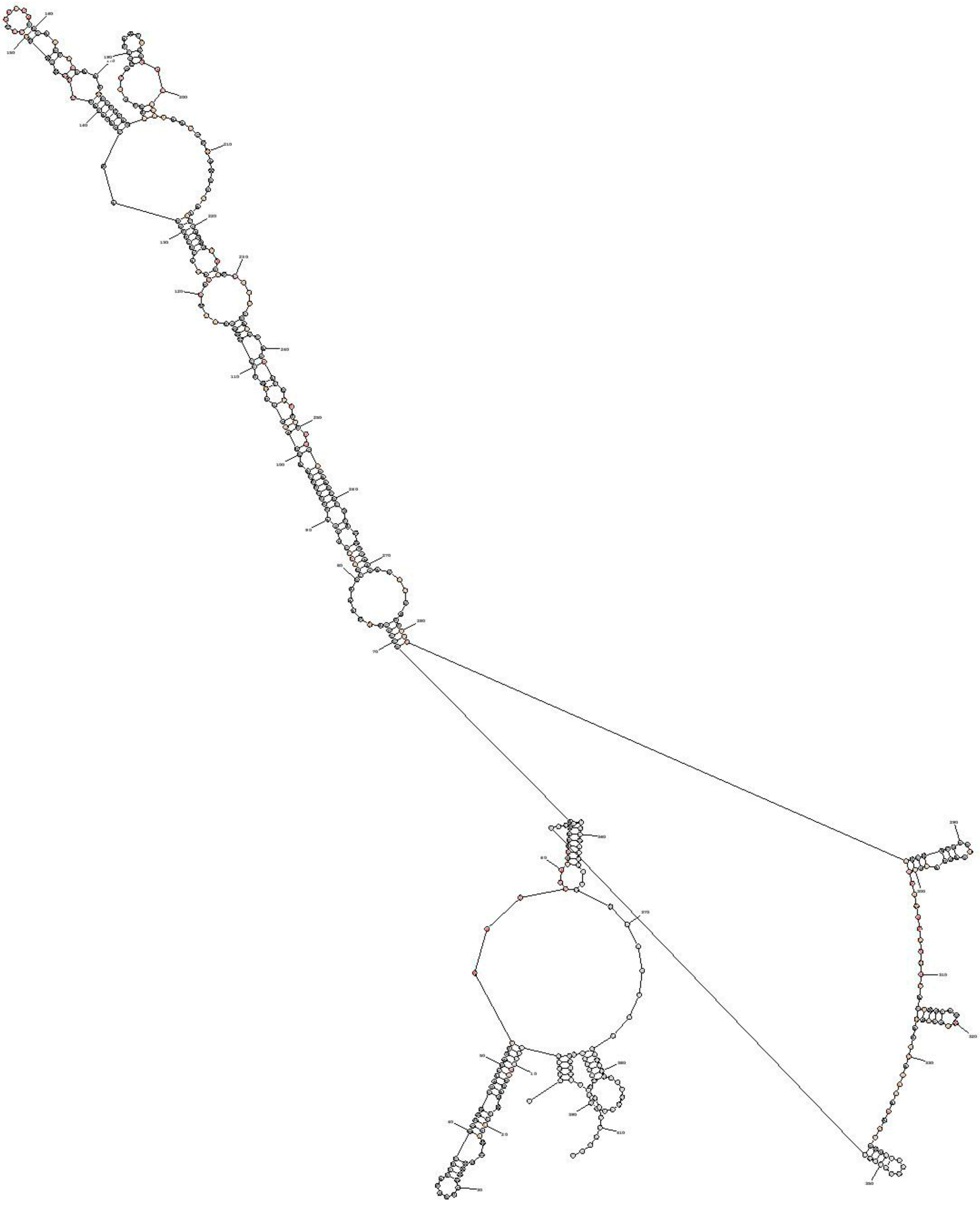

SHAPE >= 0.85

0.85 > SHAPE >= 0.4

0.4 > SHAPE

No Data

ENERGY = −206.5   MonNC414

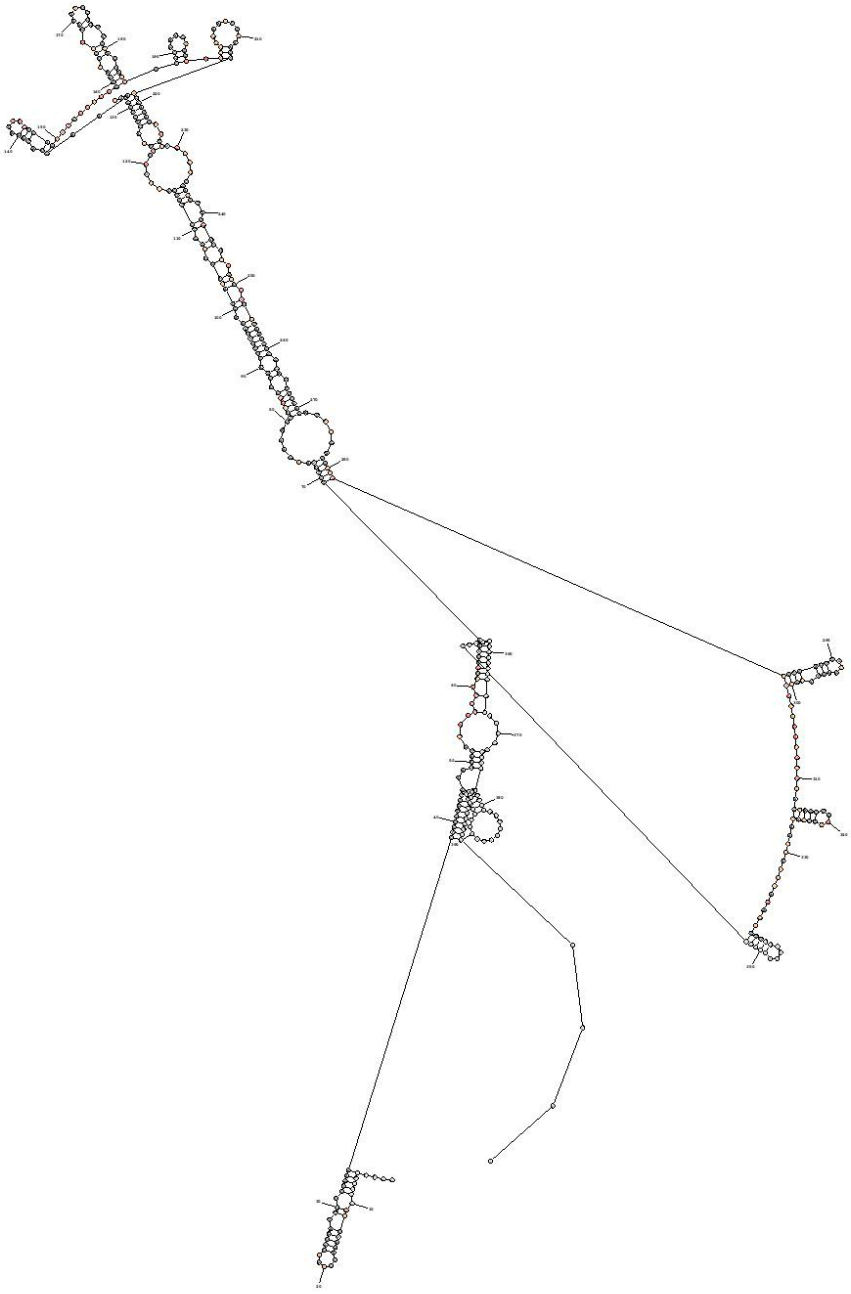

SHAPE >= 0.85
0.85 > SHAPE >= 0.4
0.4 > SHAPE
No Data

ENERGY = −206.2 MonNC414

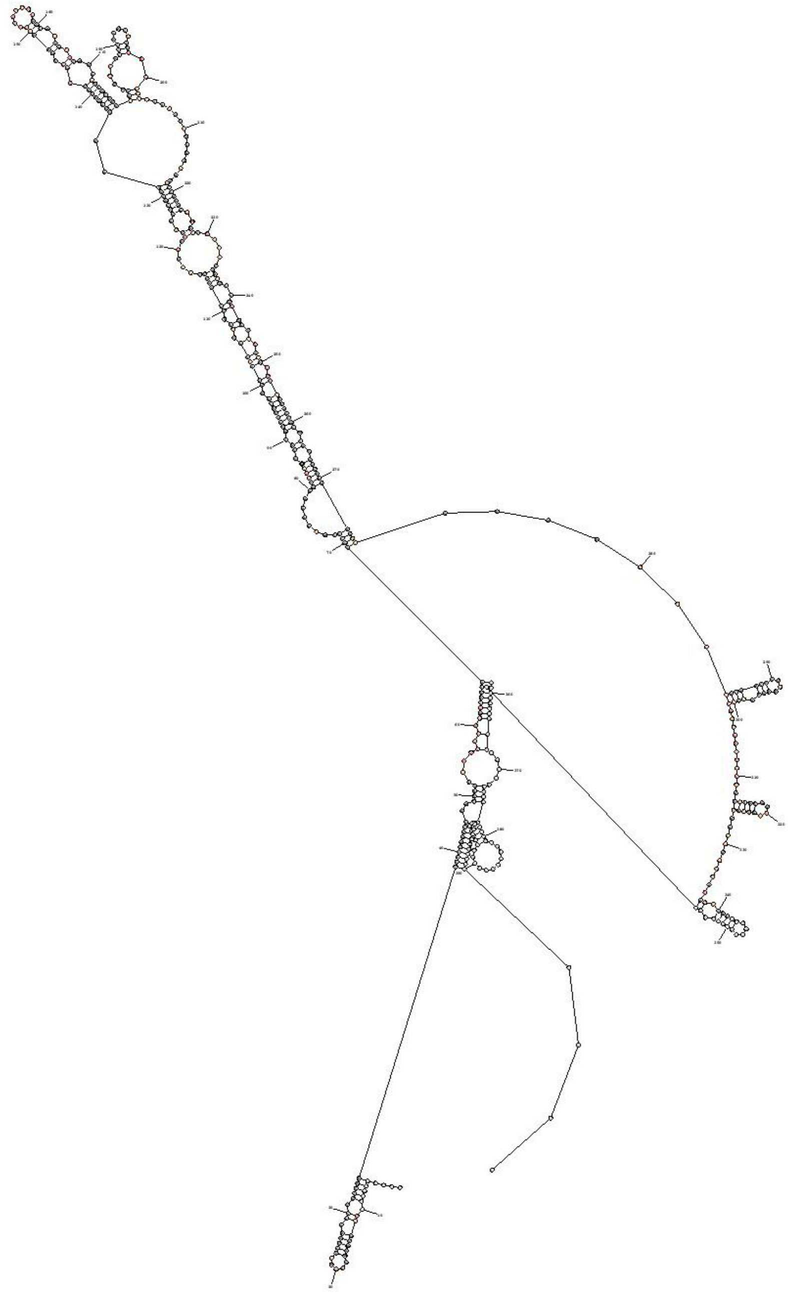

SHAPE >= 0.85
0.85 > SHAPE >= 0.4
0.4 > SHAPE
No Data

ENERGY = −205.6 MonNC414

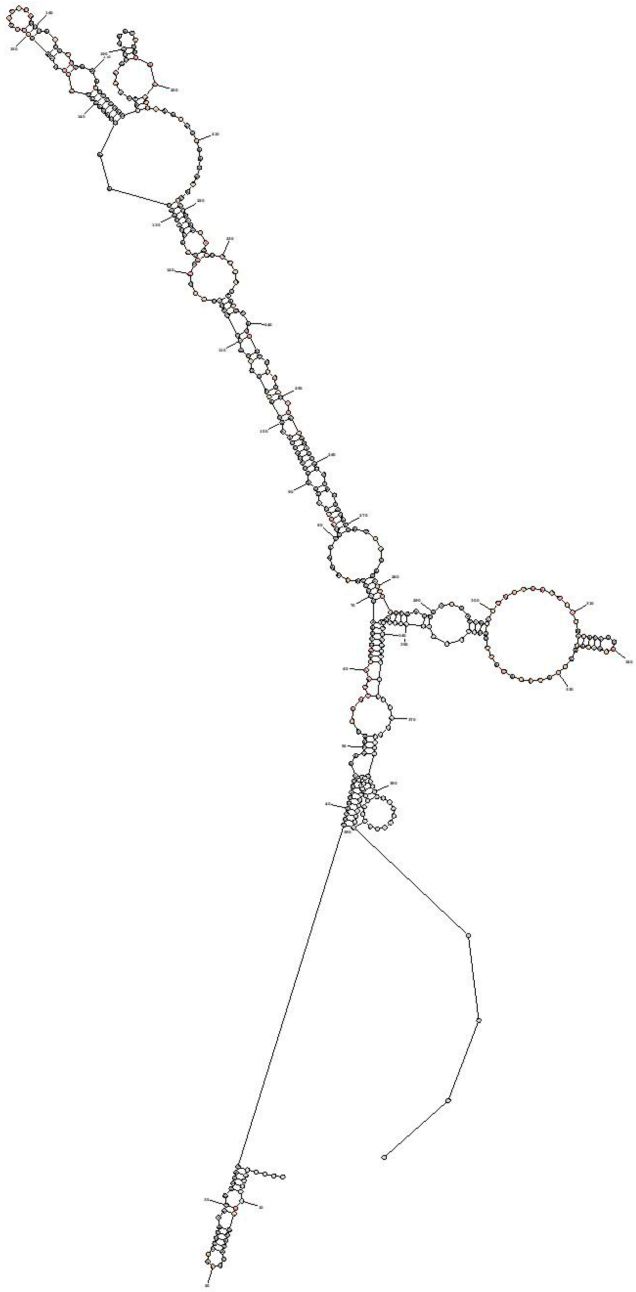

SHAPE >= 0.85
0.85 > SHAPE >= 0.4
0.4 > SHAPE
No Data

ENERGY = -205.4 MonNC414

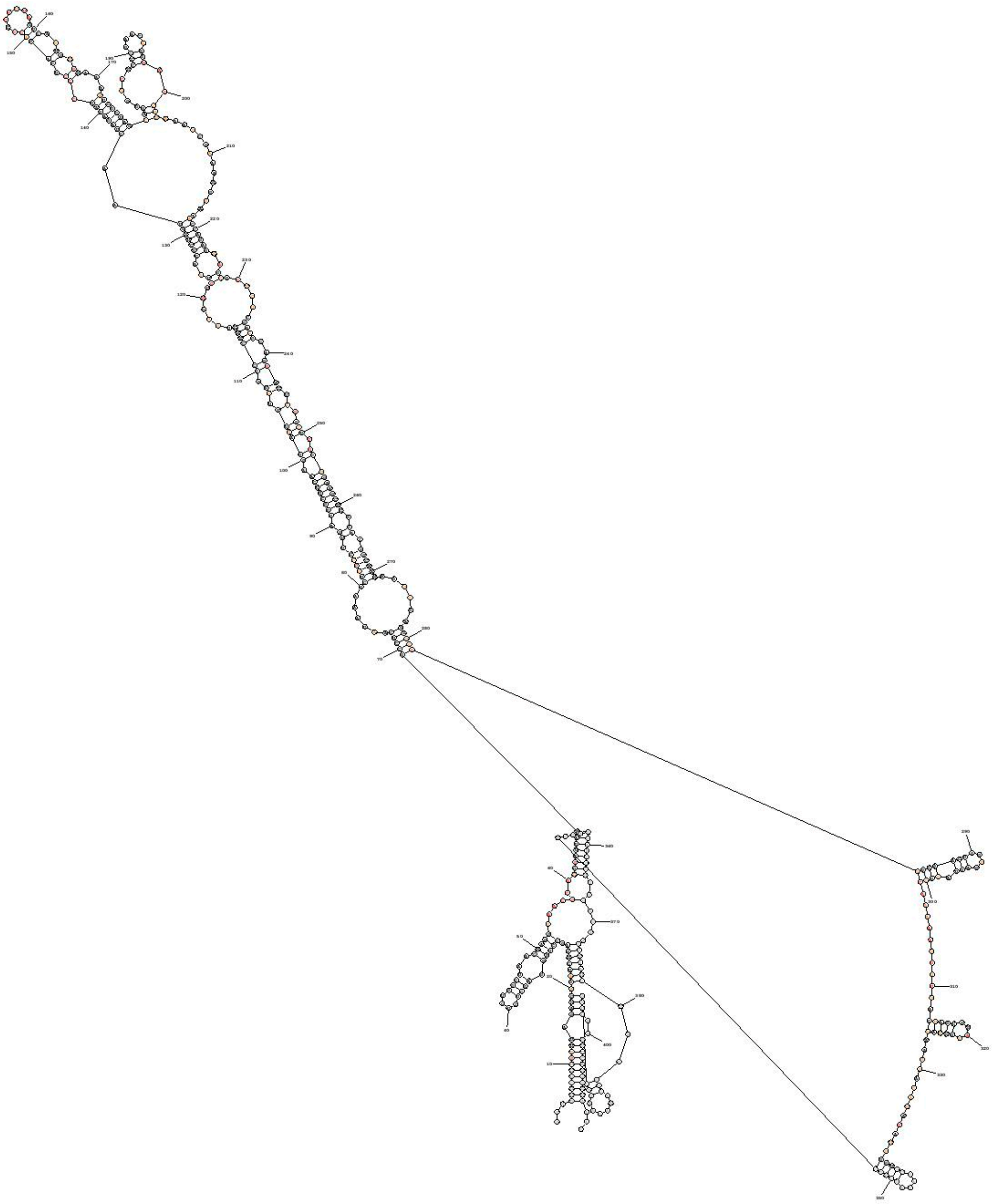

SHAPE >= 0.85

0.85 > SHAPE >= 0.4

0.4 > SHAPE

No Data

ENERGY = −205.3 MonNC414

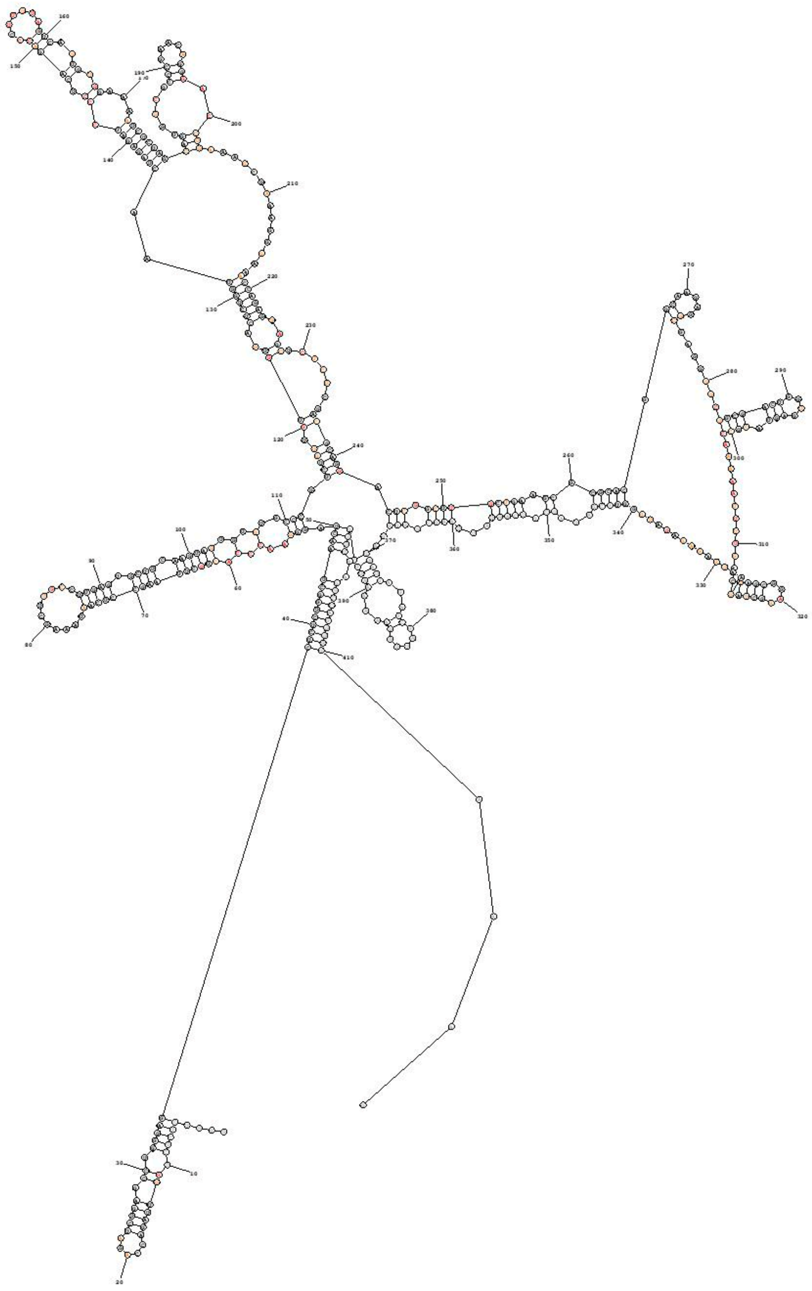

SHAPE >= 0.85
0.85 > SHAPE >= 0.4
0.4 > SHAPE
No Data

ENERGY = -205.2 MonNC414

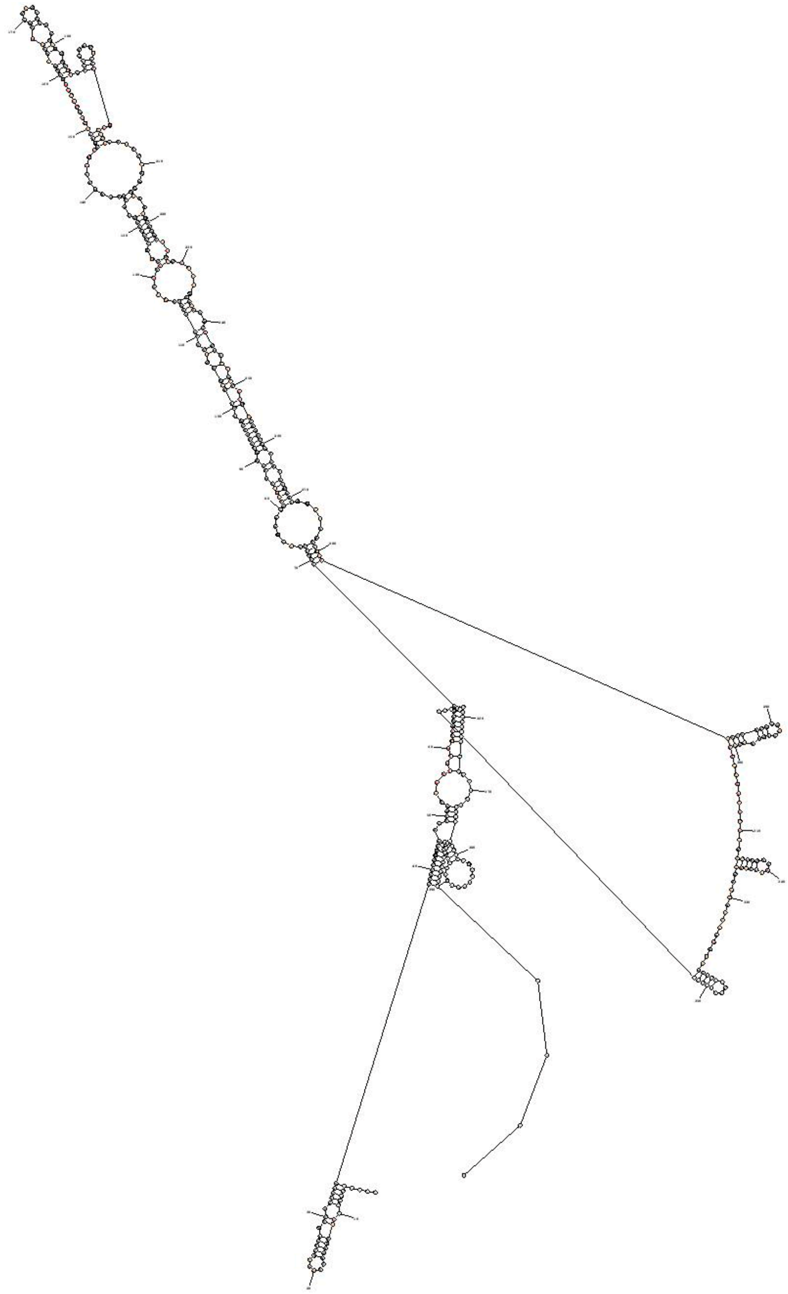

SHAPE >= 0.85
0.85 > SHAPE >= 0.4
0.4 > SHAPE
No Data

ENERGY = −204.3 MonNC414

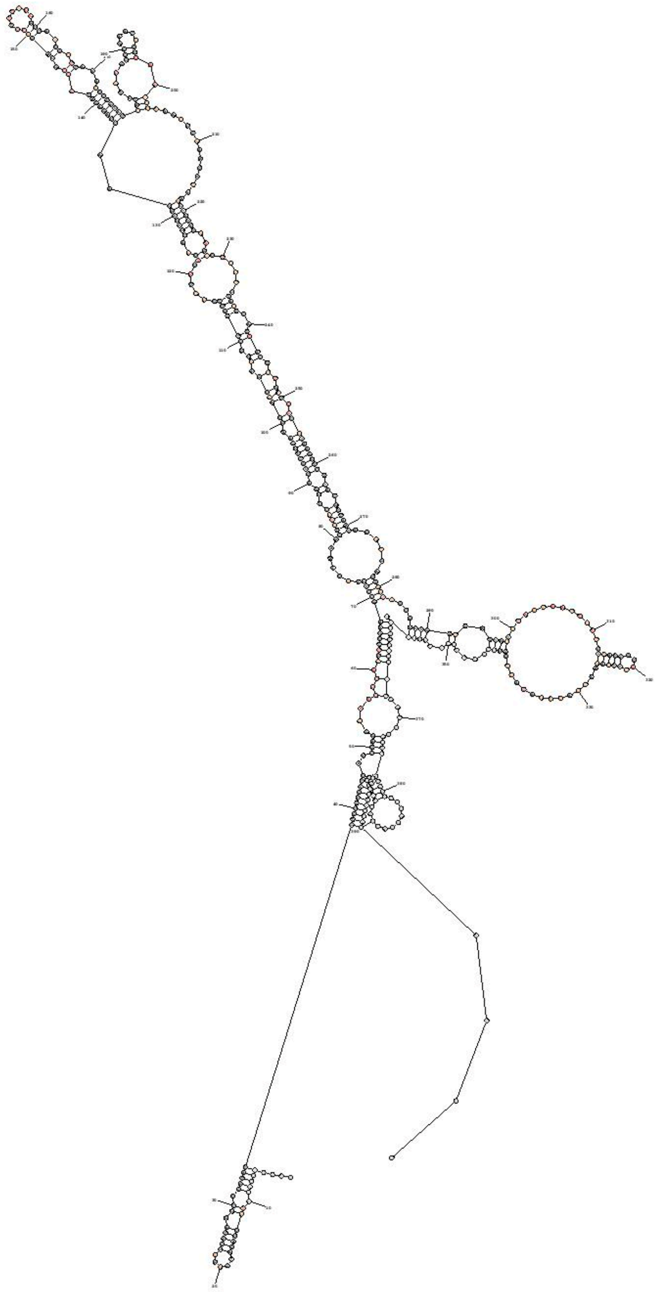

SHAPE >= 0.85
0.85 > SHAPE >= 0.4
0.4 > SHAPE
No Data

ENERGY = -204.2 MonNC414

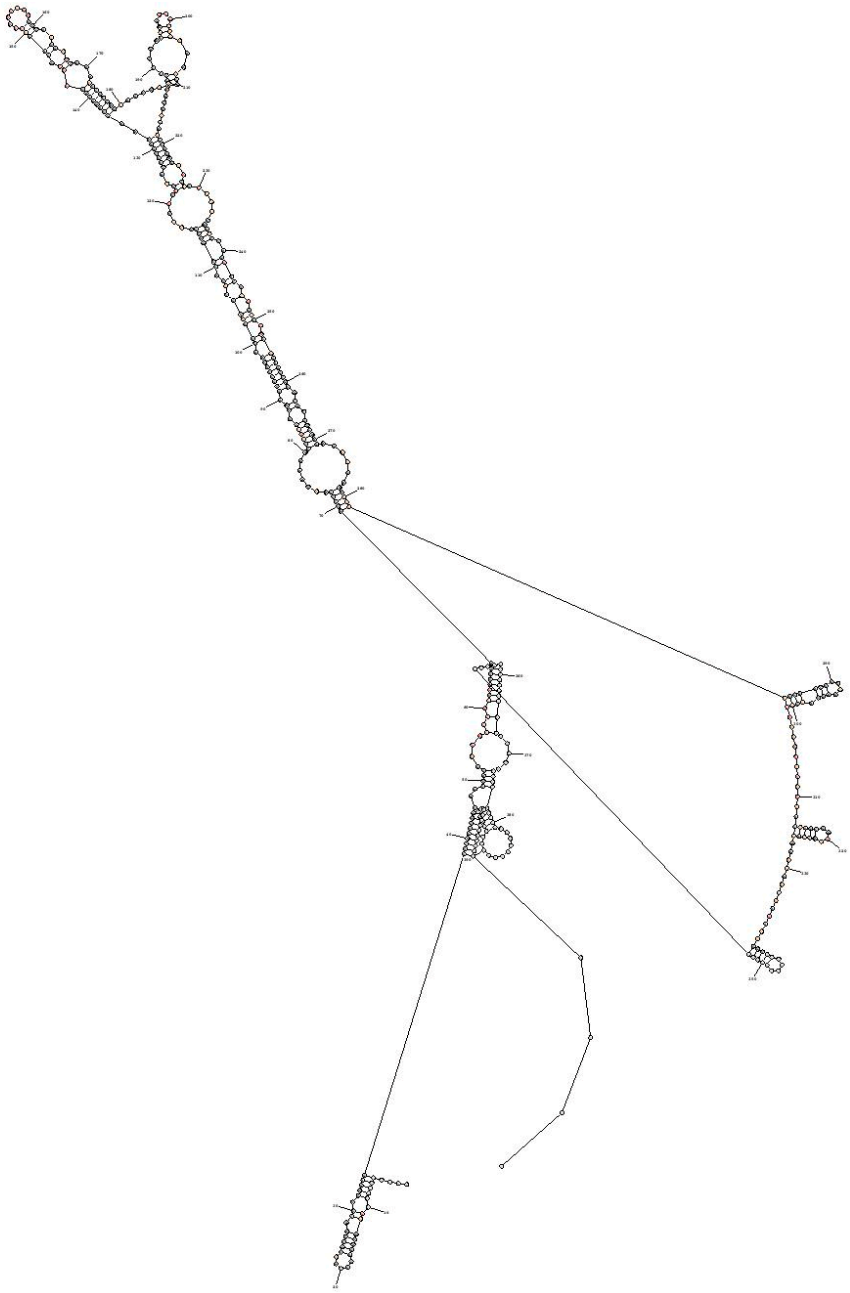

SHAPE >= 0.85
0.85 > SHAPE >= 0.4
0.4 > SHAPE
No Data

ENERGY = -204.1 MonNC414

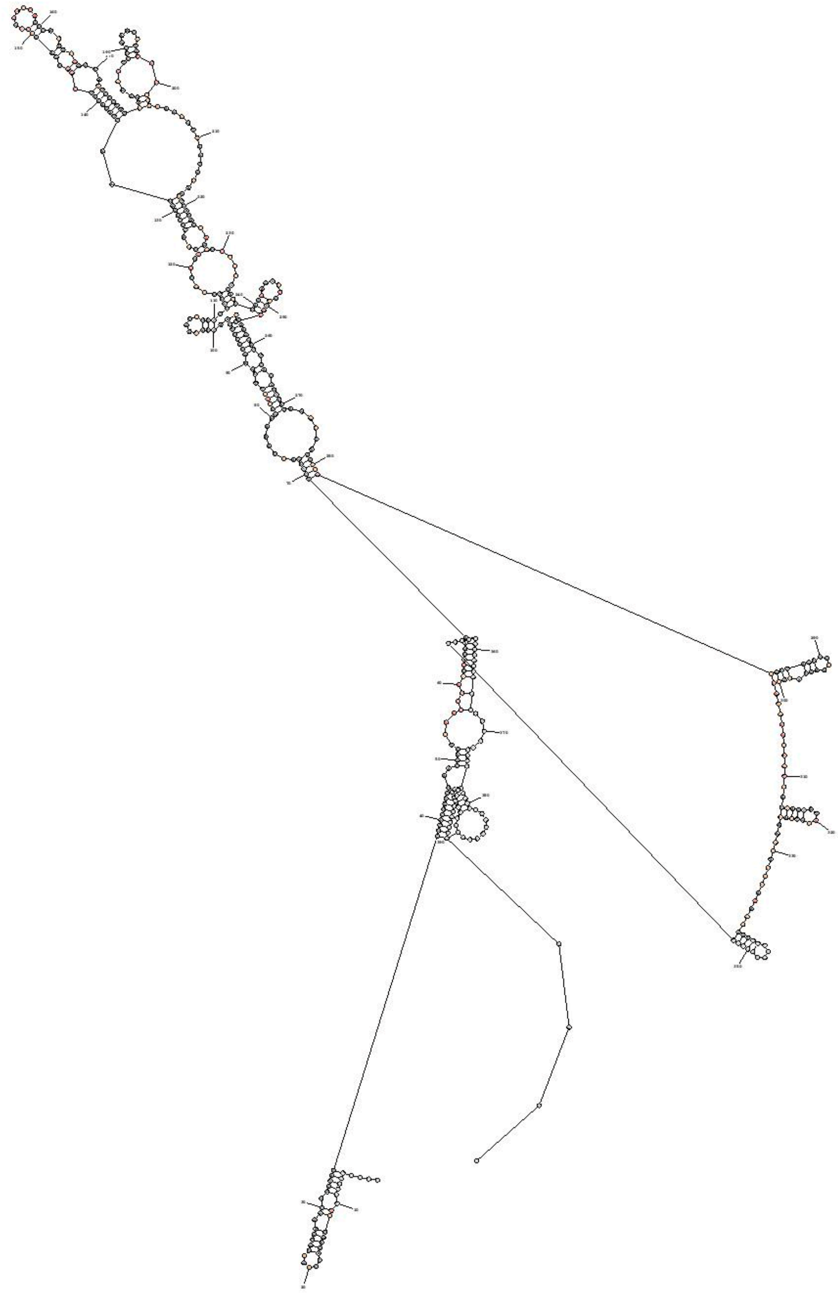

SHAPE >= 0.85

0.85 > SHAPE >= 0.4

0.4 > SHAPE

No Data

ENERGY = -203.5 MonNC414

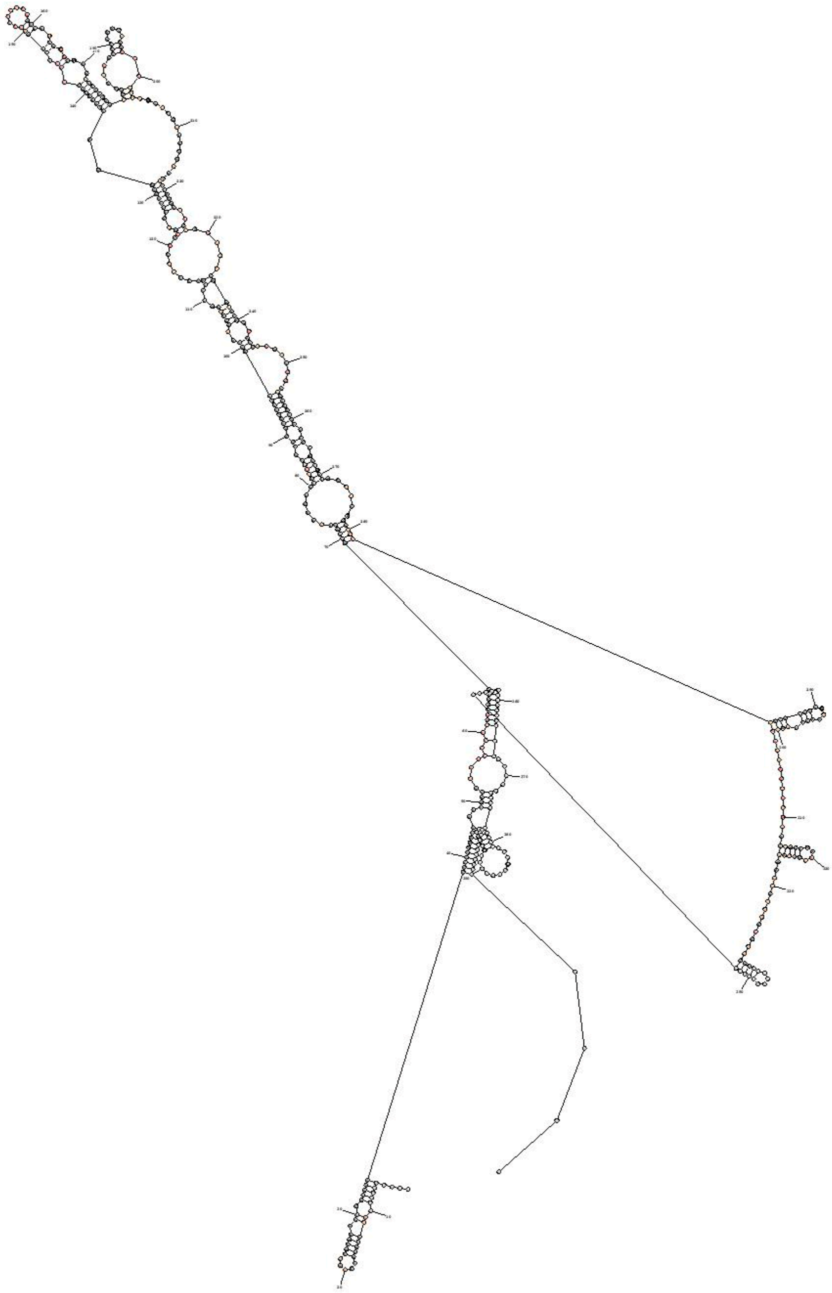

SHAPE >= 0.85
0.85 > SHAPE >= 0.4
0.4 > SHAPE
No Data

ENERGY = -203.1 MonNC414

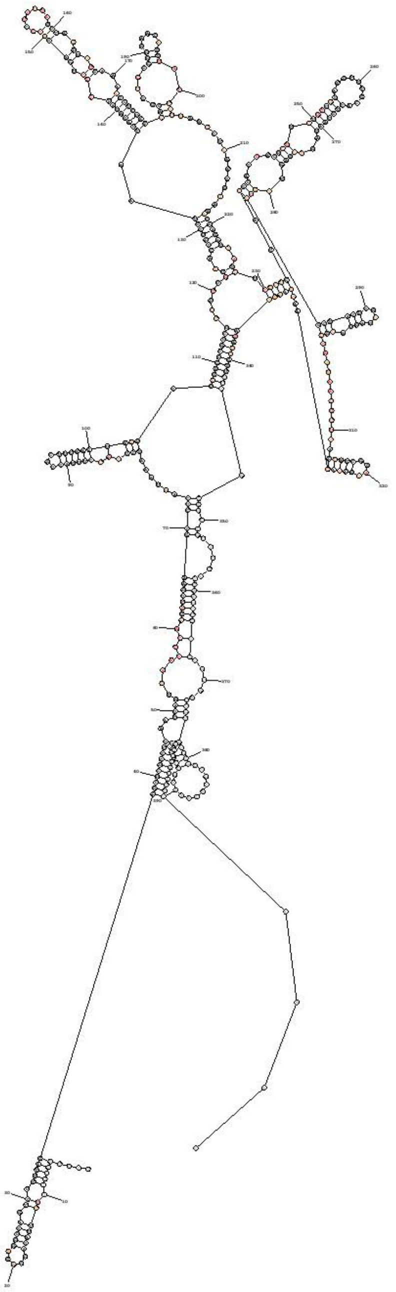

SHAPE >= 0.85
0.85 > SHAPE >= 0.4
0.4 > SHAPE
No Data

ENERGY = −203.0 MonNC414

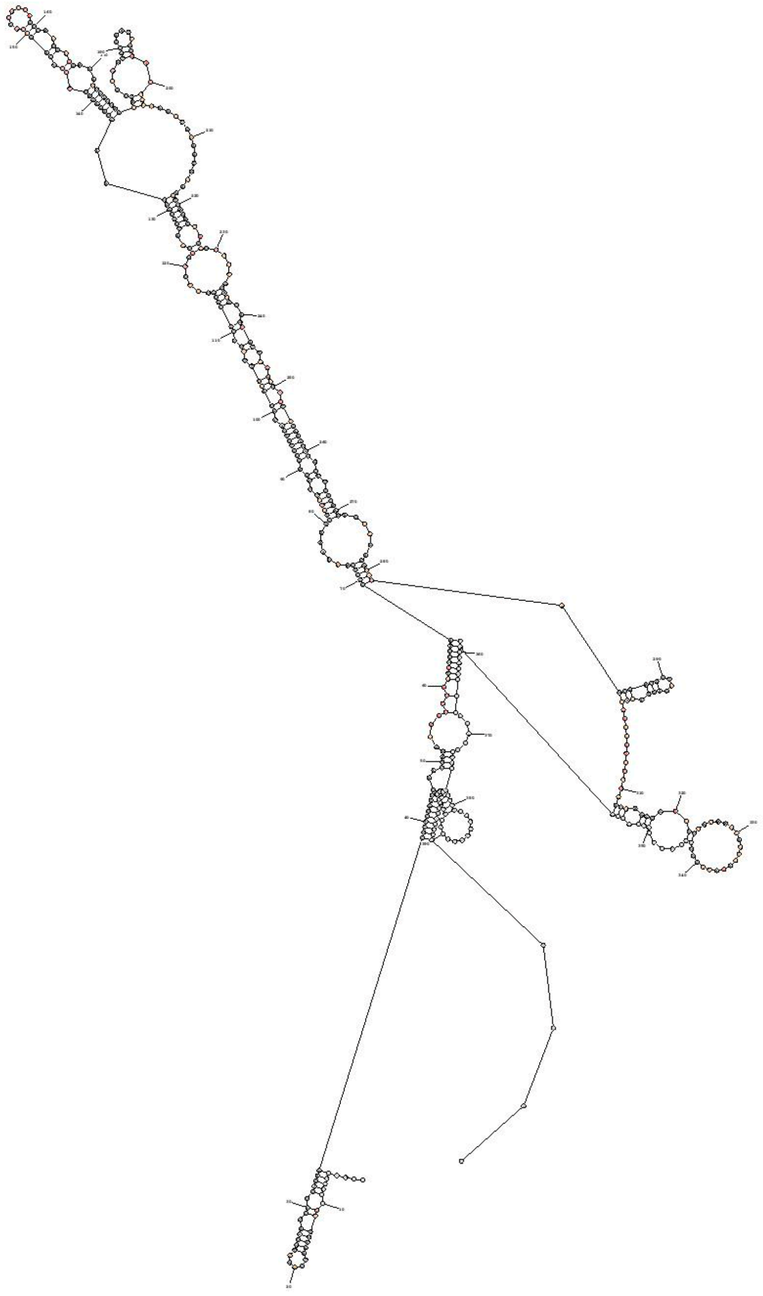

SHAPE >= 0.85
0.85 > SHAPE >= 0.4
0.4 > SHAPE
No Data

ENERGY = -203.0 MonNC414

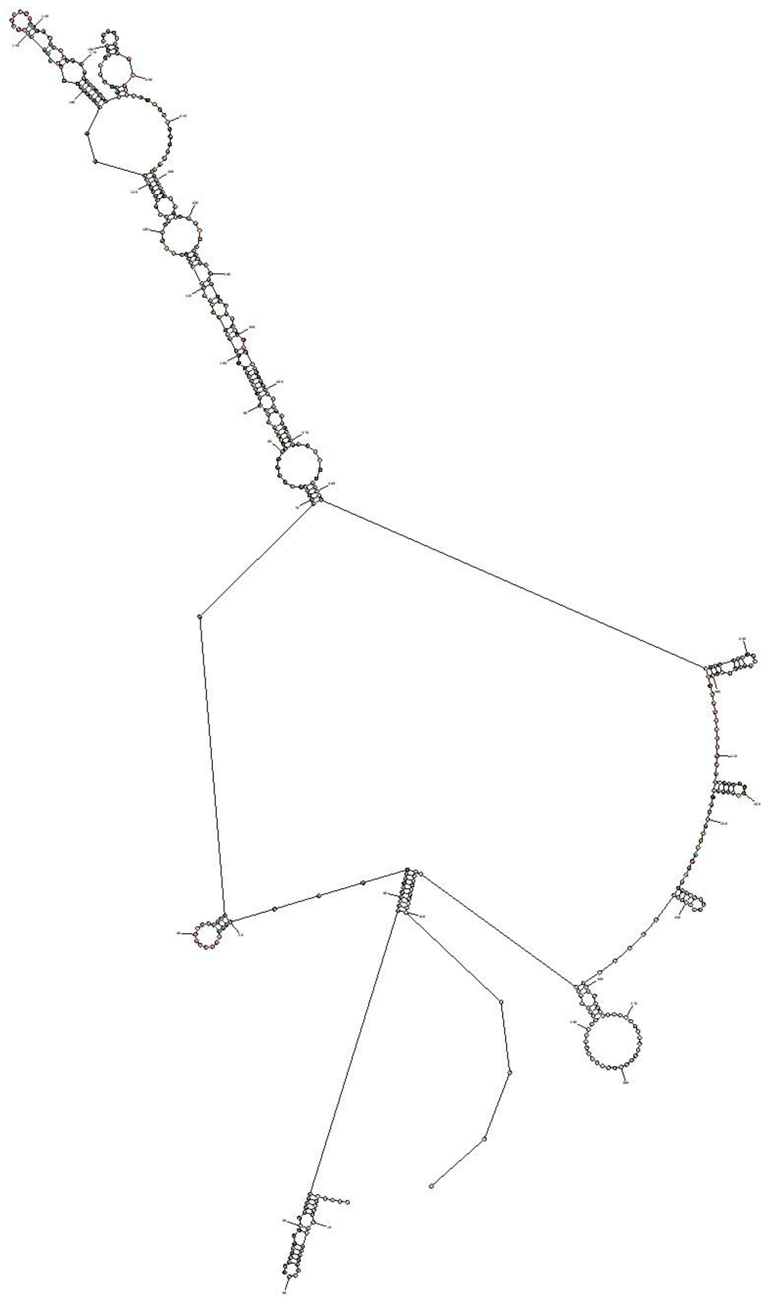

SHAPE >= 0.85
0.85 > SHAPE >= 0.4
0.4 > SHAPE
No Data

ENERGY = -202.5 MonNC414

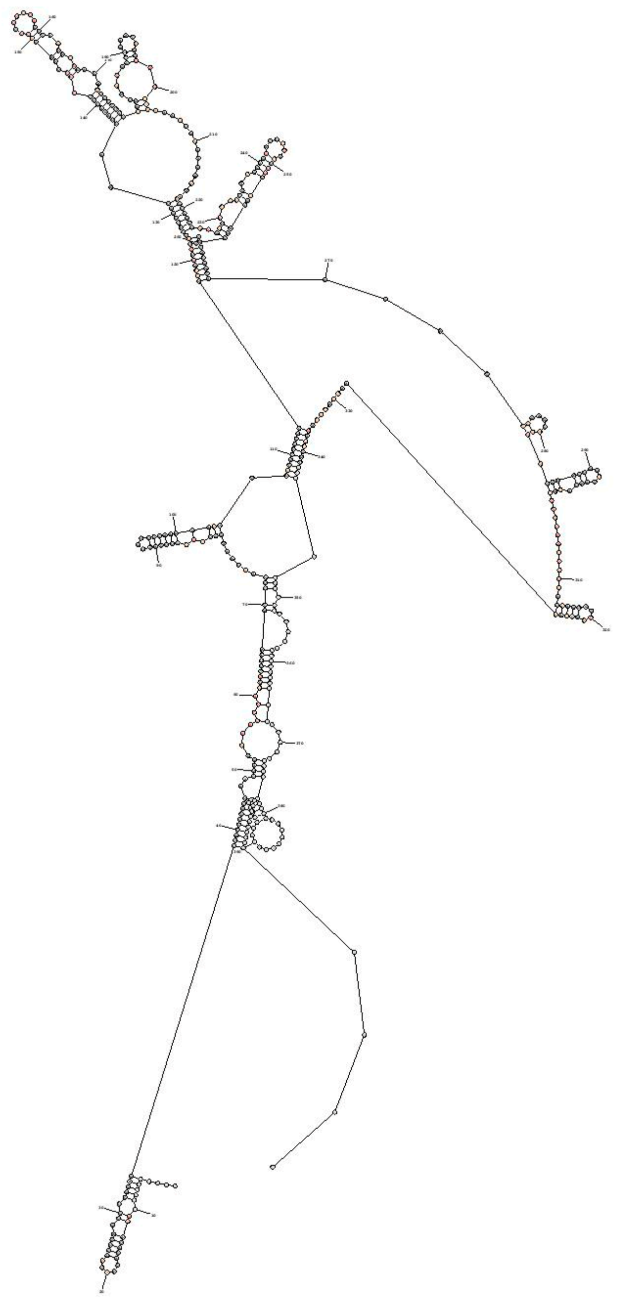

SHAPE >= 0.85
0.85 > SHAPE >= 0.4
0.4 > SHAPE
No Data

ENERGY = −202.4   MonNC414

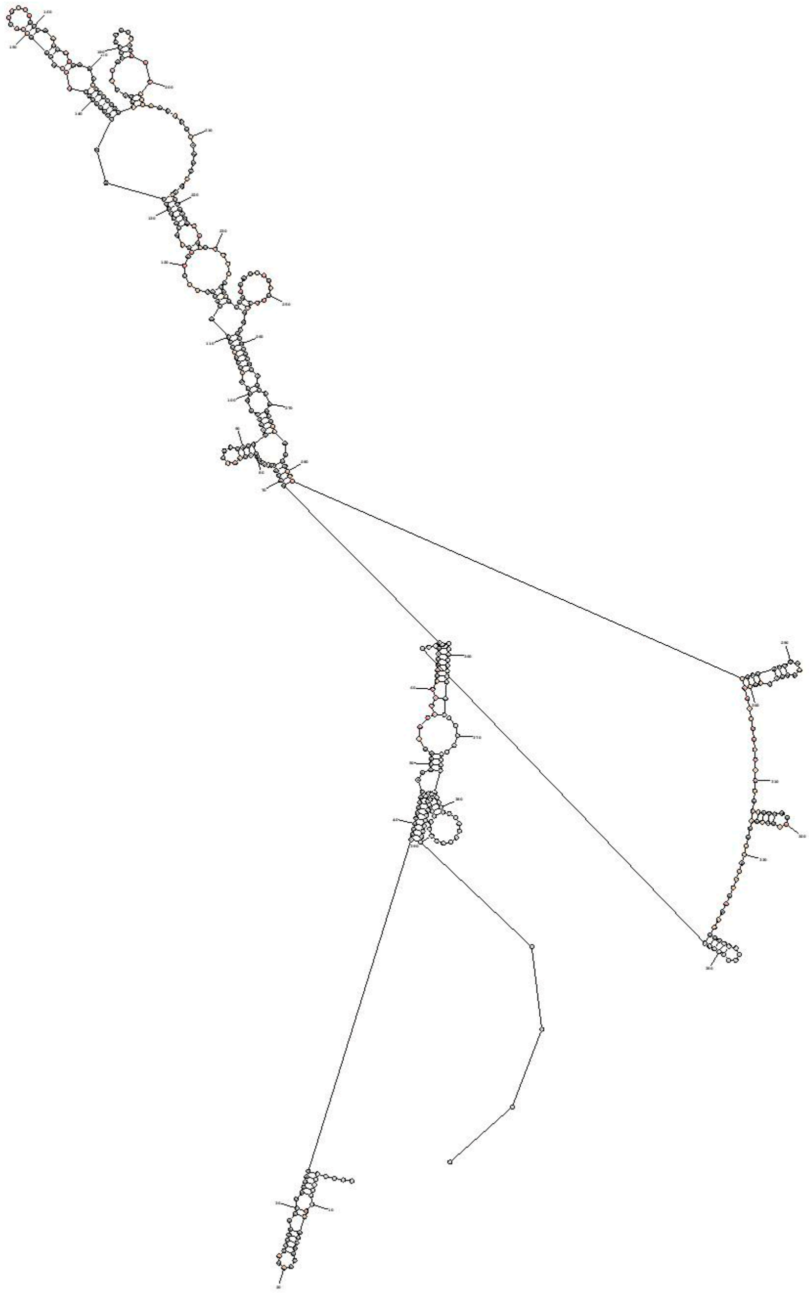

SHAPE >= 0.85
0.85 > SHAPE >= 0.4
0.4 > SHAPE
No Data

ENERGY = -202.3 MonNC414

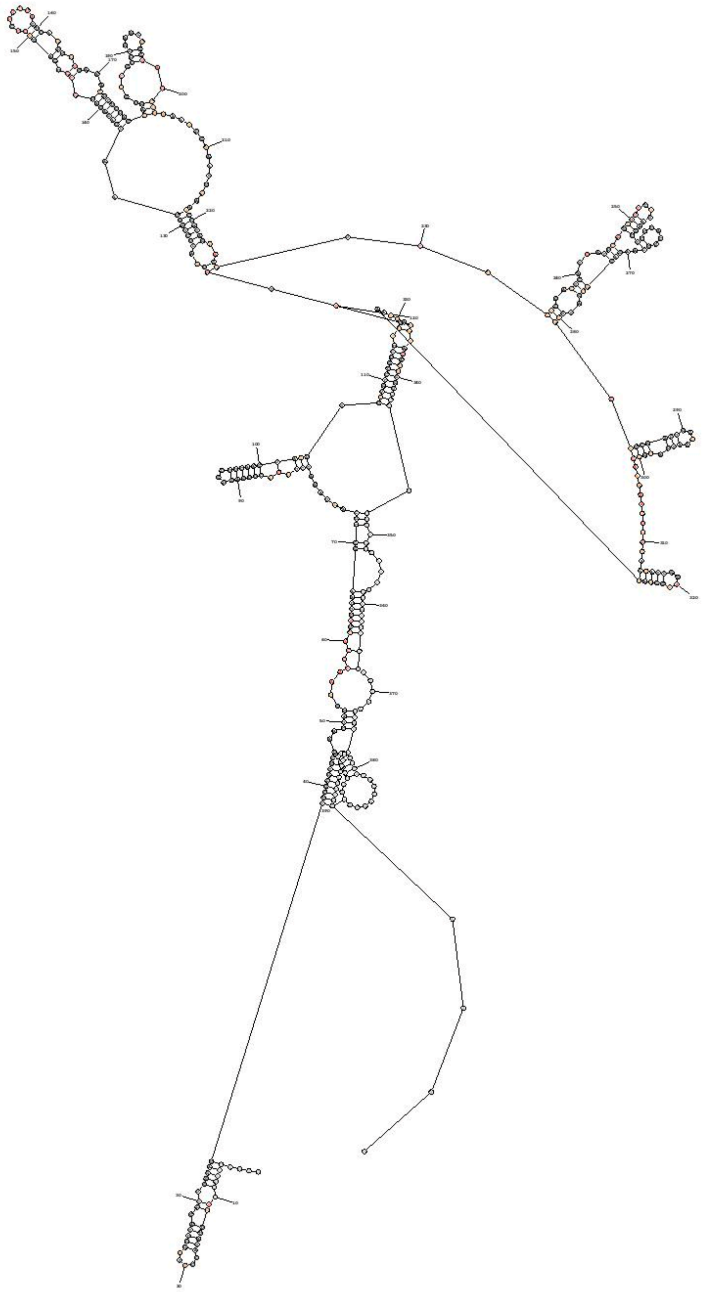

SHAPE >= 0.85
0.85 > SHAPE >= 0.4
0.4 > SHAPE
No Data

ENERGY = -202.0 MonNC414

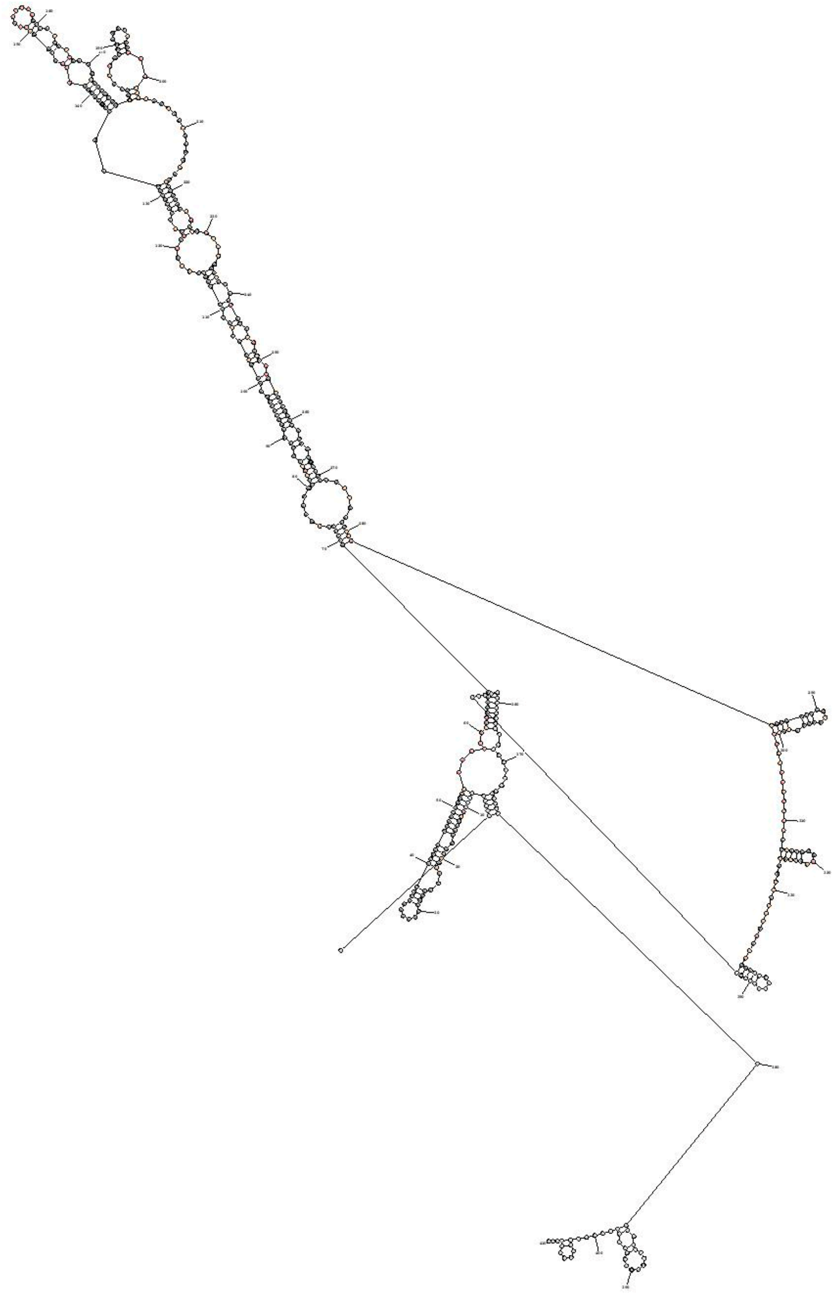

SHAPE >= 0.85

0.85 > SHAPE >= 0.4

0.4 > SHAPE

No Data

ENERGY = -201.9 MonNC414

Supplementary figure 6 - Dimer 414

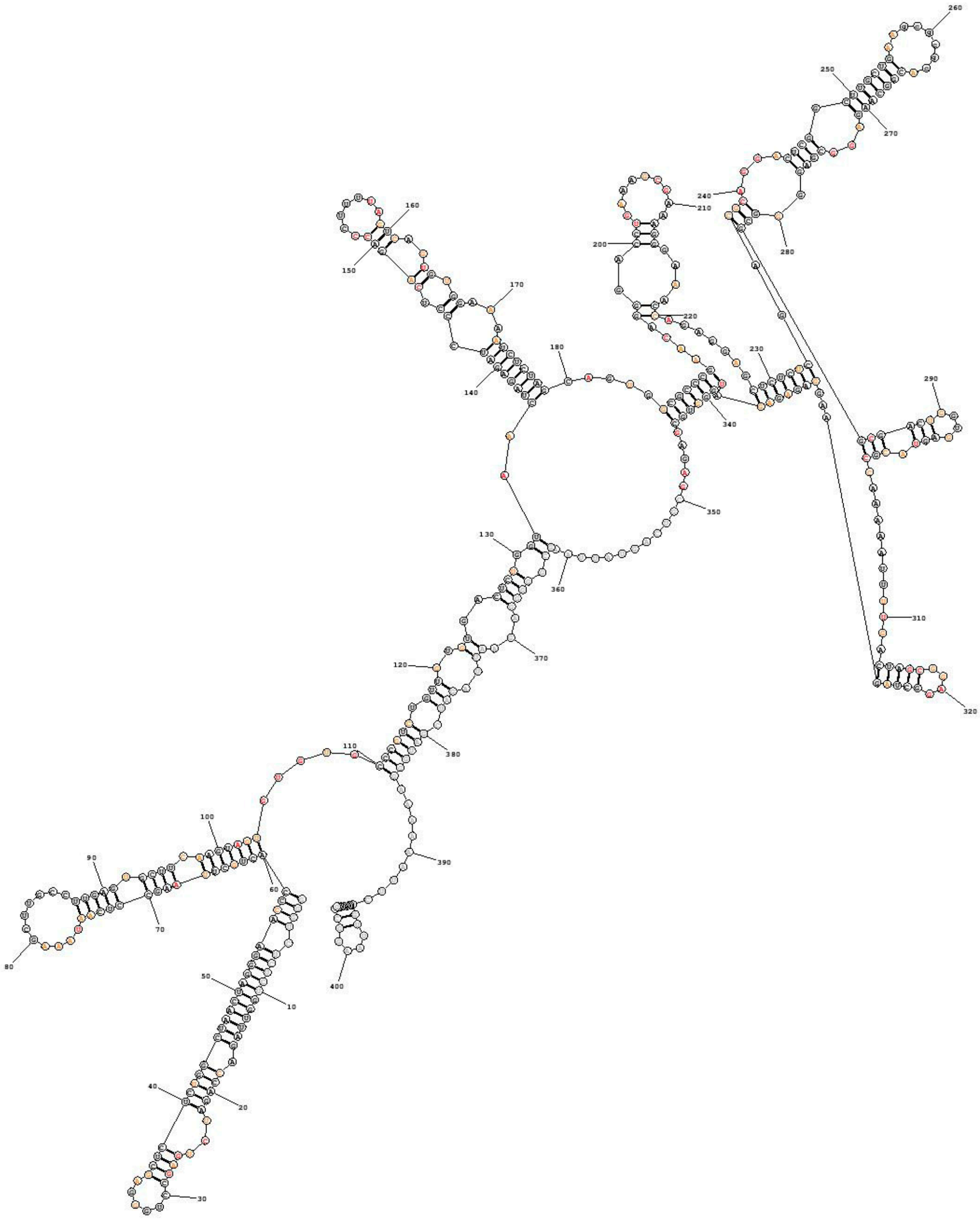

SHAPE >= 0.85

0.85 > SHAPE >= 0.4

0.4 > SHAPE

No Data

ENERGY = -196.0 Dim414

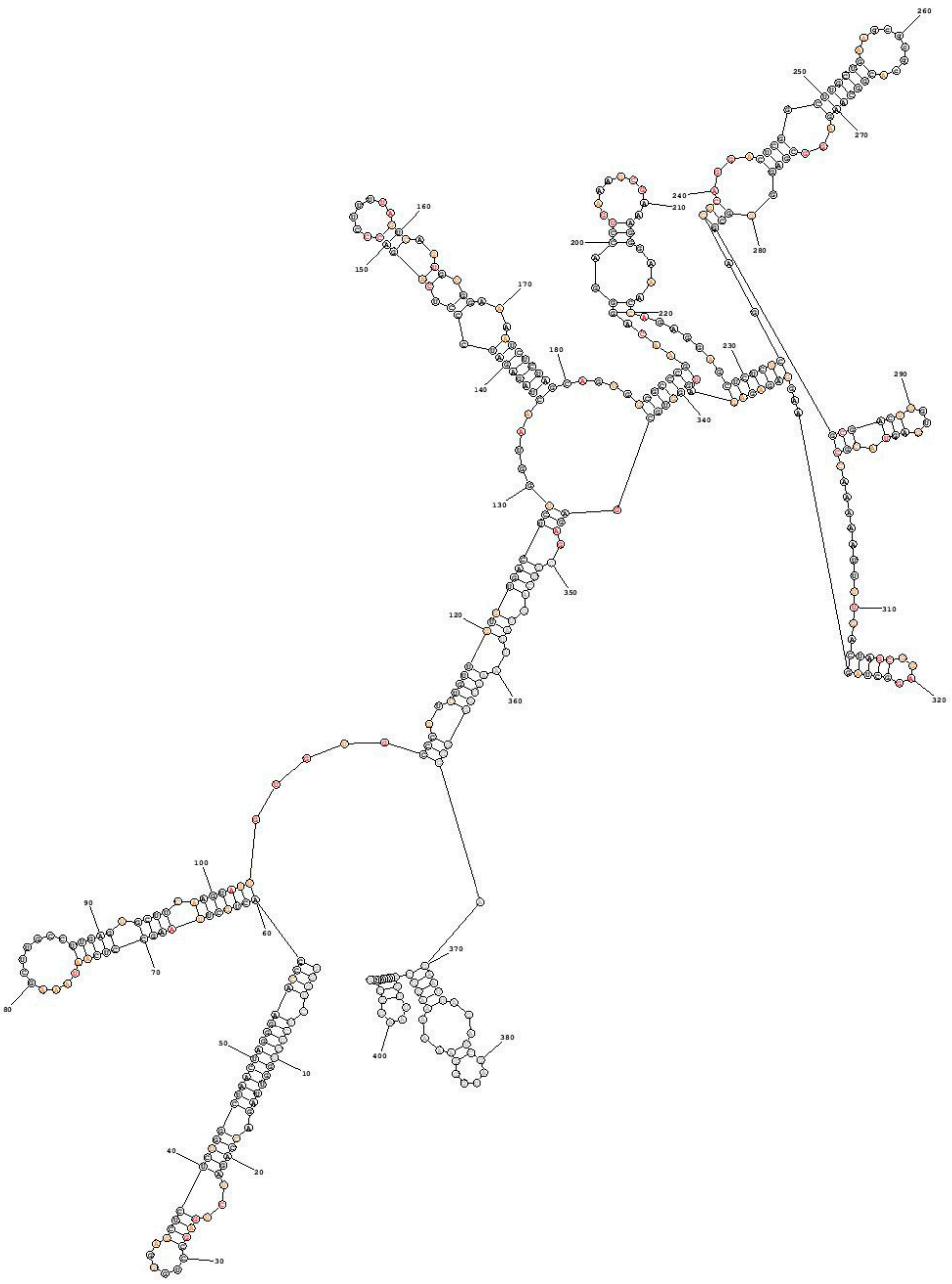

SHAPE >= 0.85
0.85 > SHAPE >= 0.4
0.4 > SHAPE
No Data

ENERGY = −195.6 Dim414

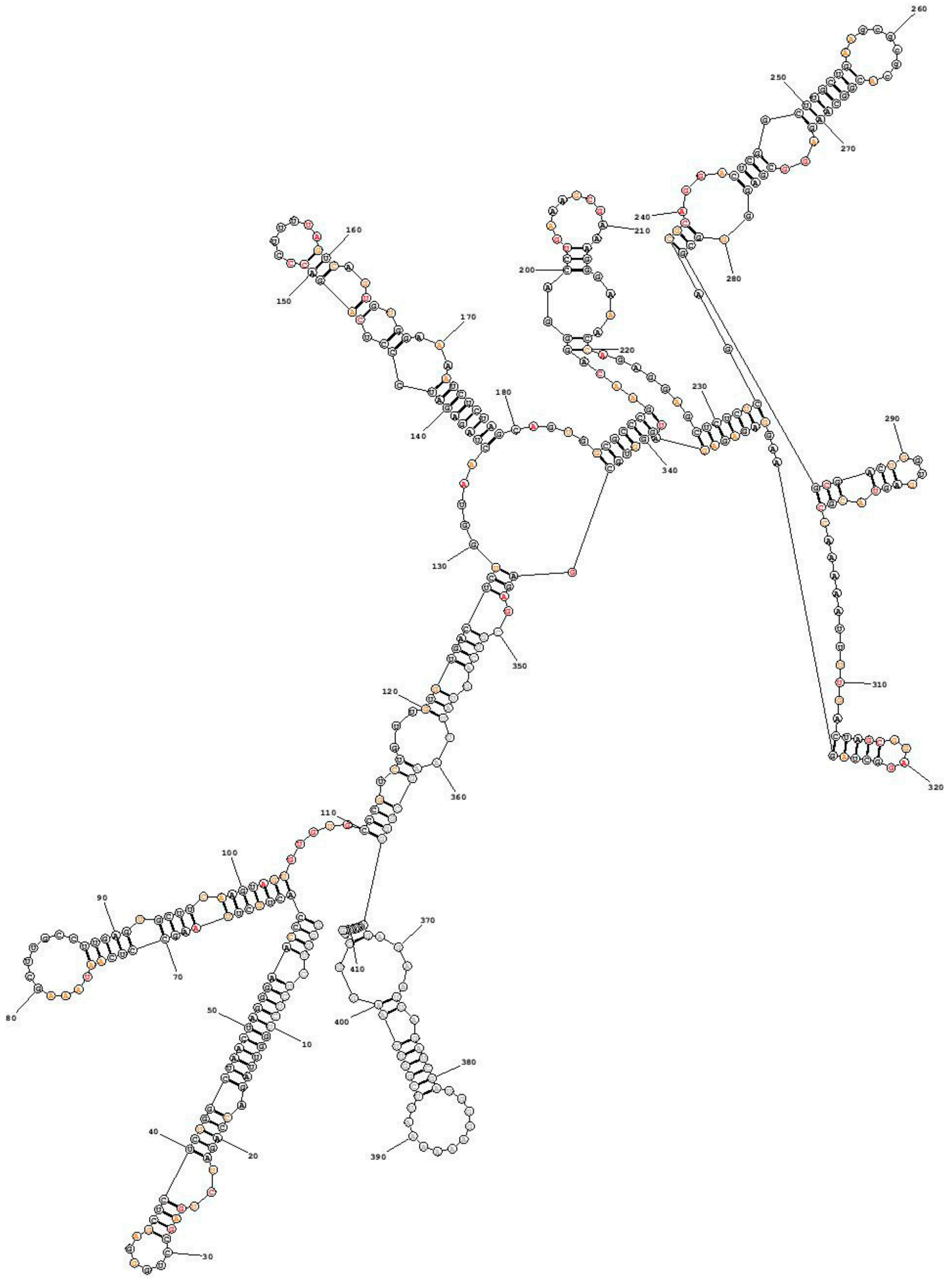

SHAPE >= 0.85

0.85 > SHAPE >= 0.4

0.4 > SHAPE

No Data

ENERGY = -195.5 Dim414

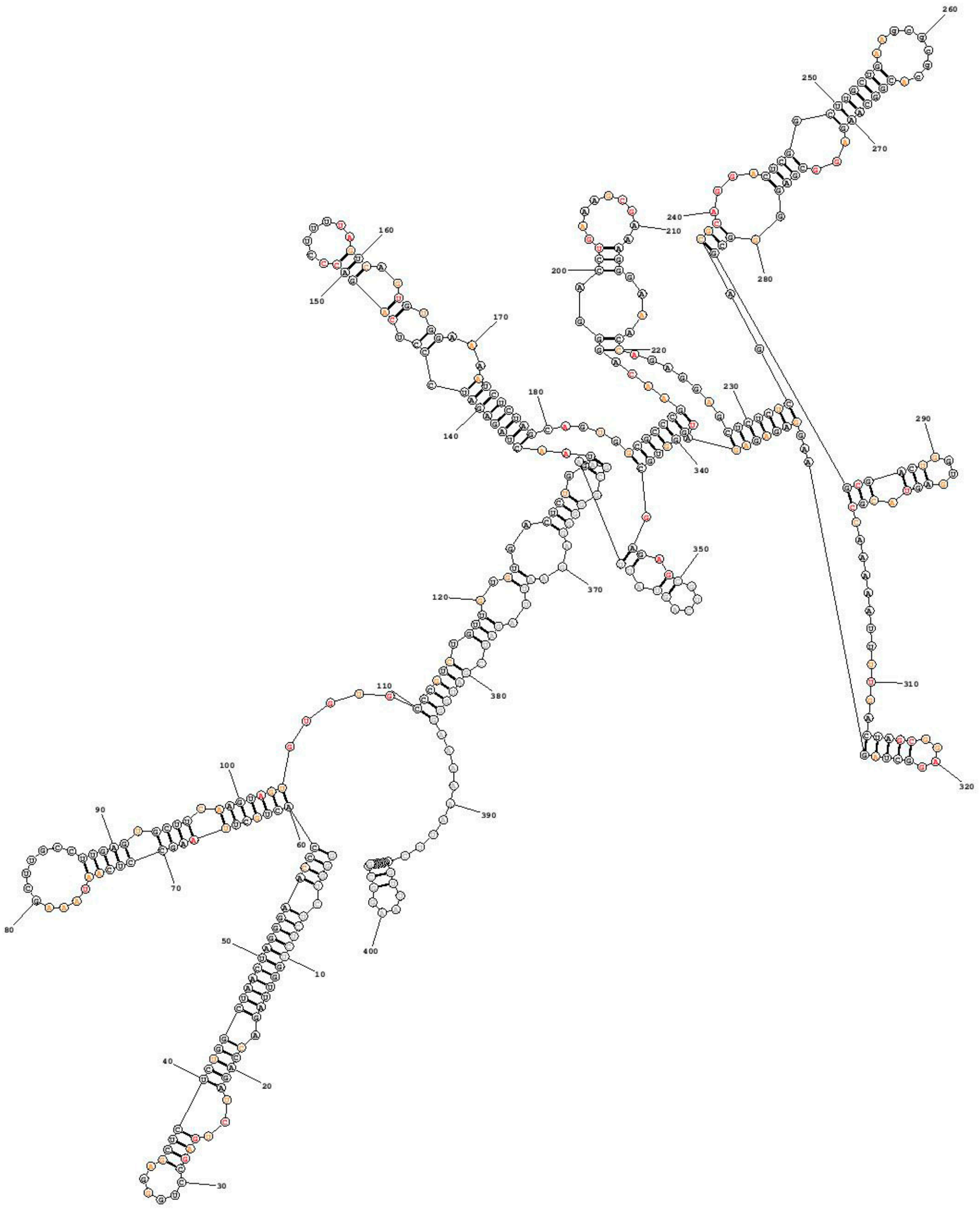

SHAPE >= 0.85

0.85 > SHAPE >= 0.4

0.4 > SHAPE

No Data

ENERGY = −195.4 Dim414

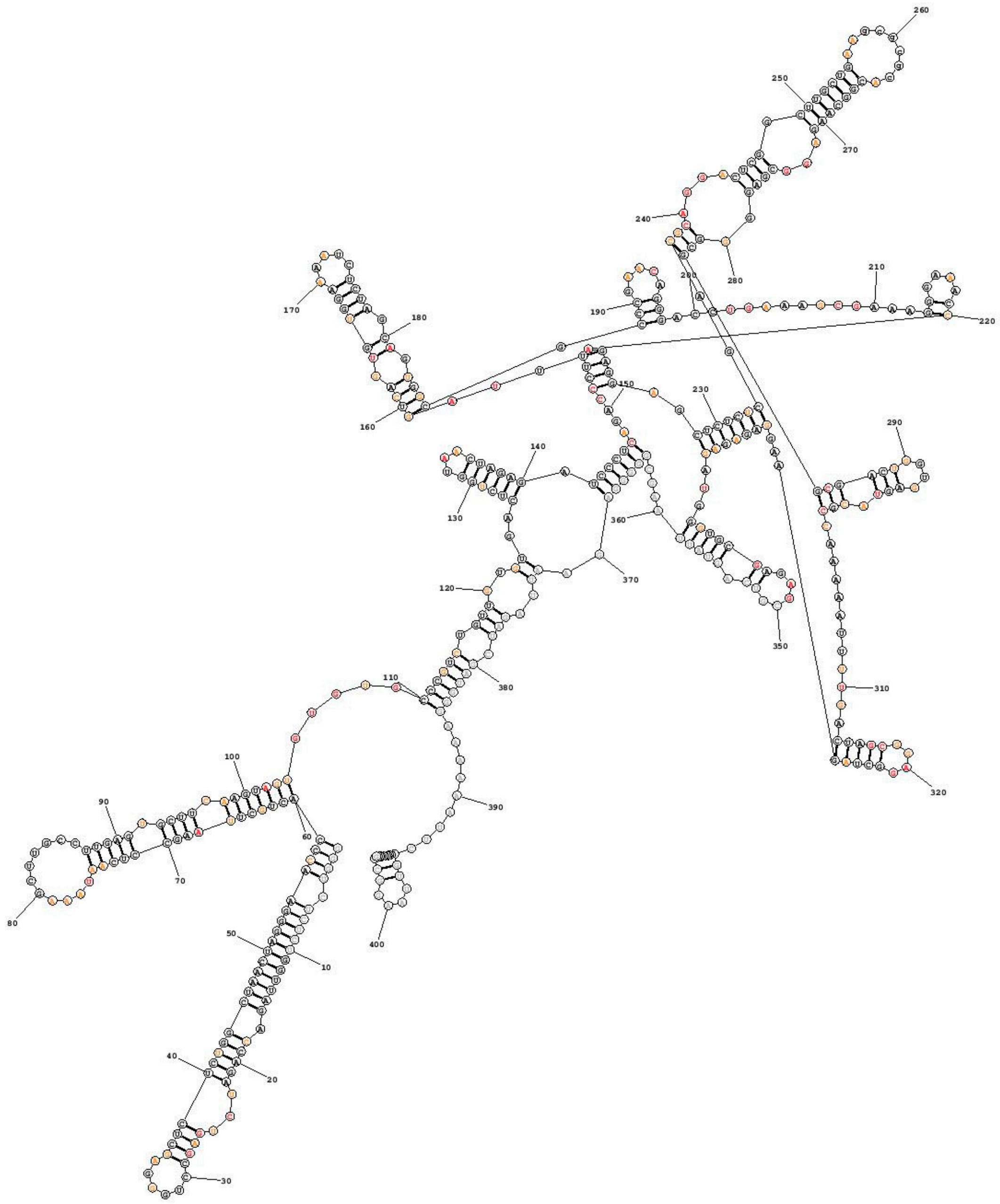

SHAPE >= 0.85
0.85 > SHAPE >= 0.4
0.4 > SHAPE
No Data

ENERGY = -195.3 Dim414

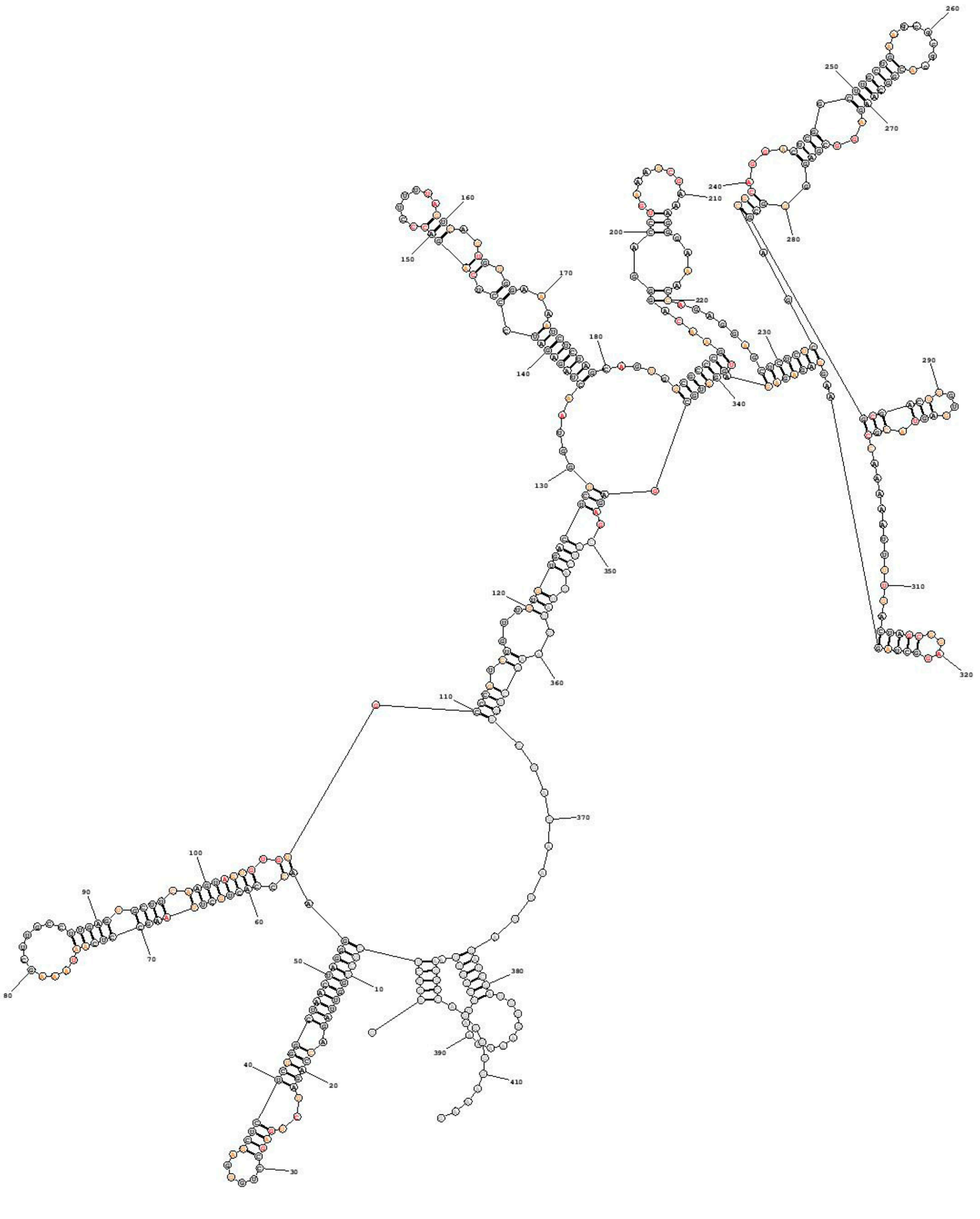

SHAPE >= 0.85
0.85 > SHAPE >= 0.4
0.4 > SHAPE
No Data

ENERGY = −195.3 Dim414

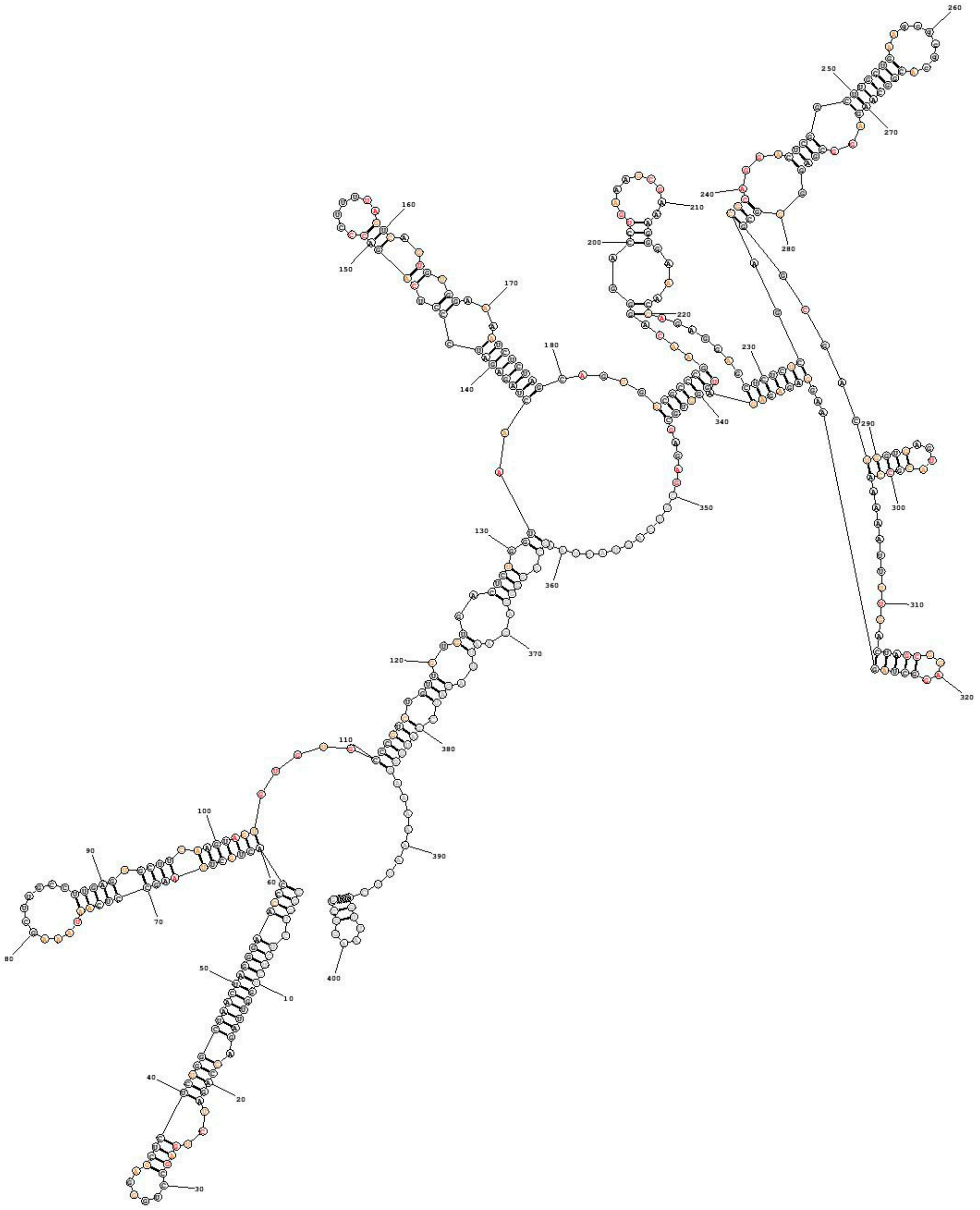

SHAPE >= 0.85
0.85 > SHAPE >= 0.4
0.4 > SHAPE
No Data

ENERGY = -195.1 Dim414

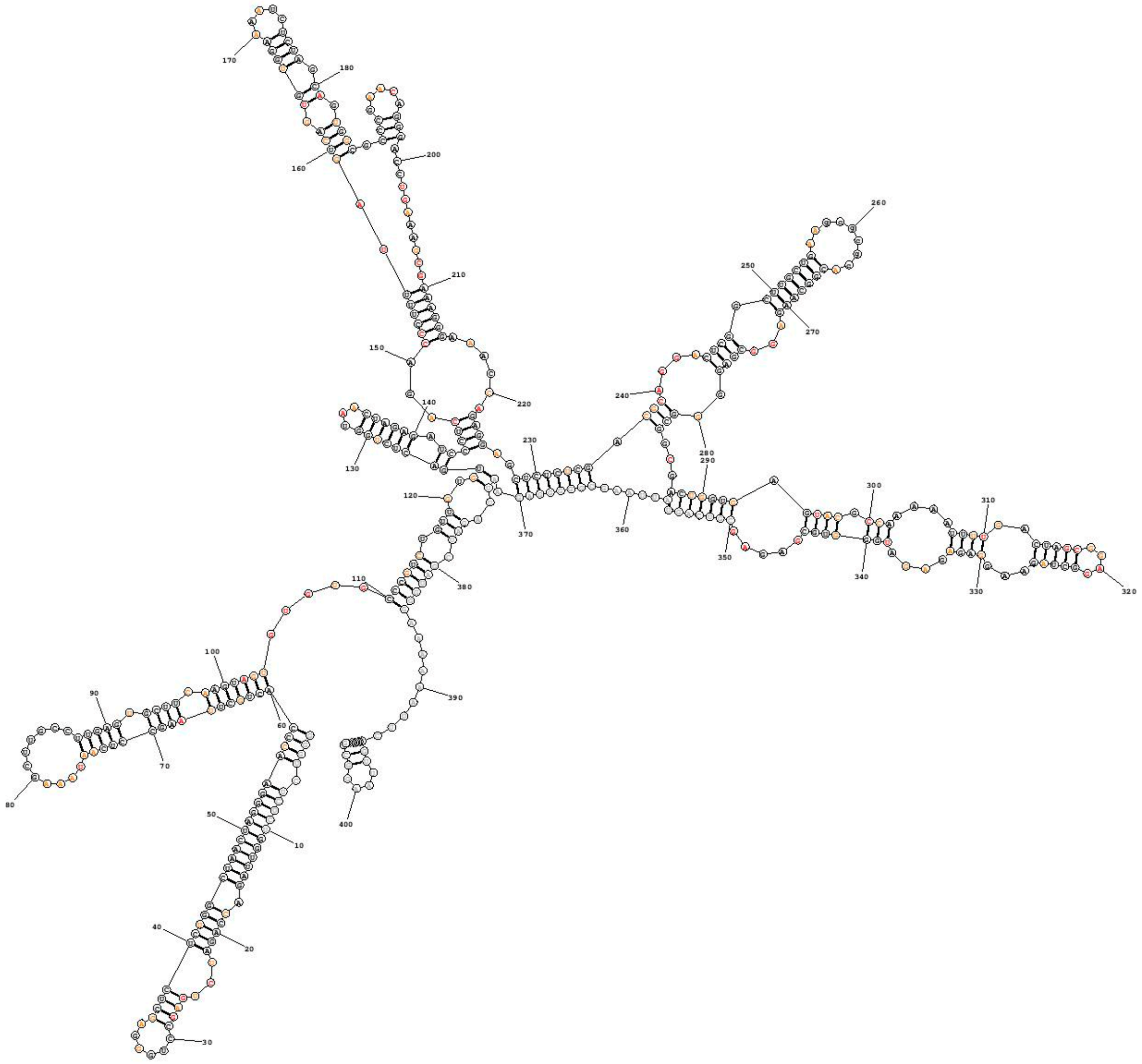

SHAPE >= 0.85
0.85 > SHAPE >= 0.4
0.4 > SHAPE
No Data

ENERGY = -195.0 Dim414

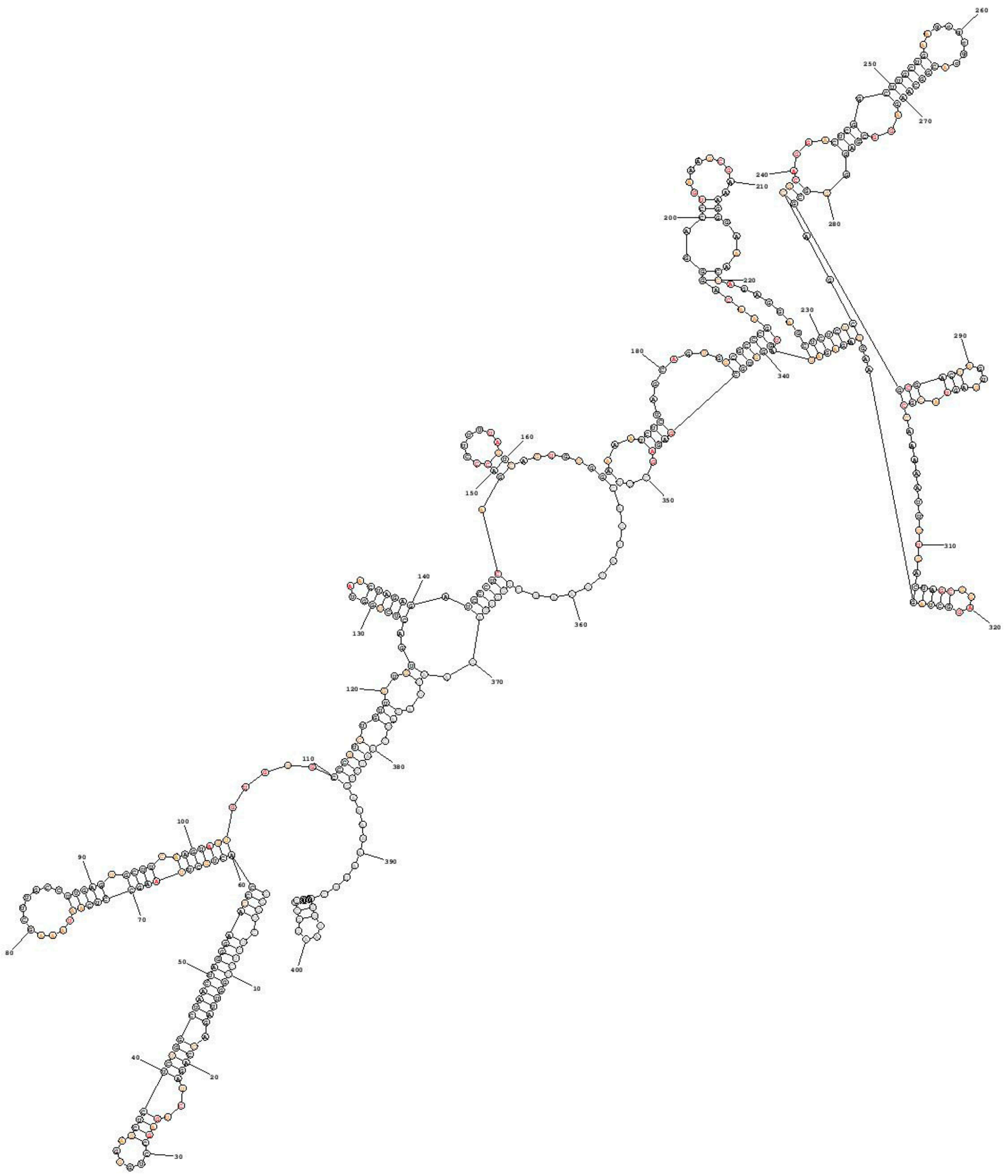

SHAPE >= 0.85
0.85 > SHAPE >= 0.4
0.4 > SHAPE
No Data

ENERGY = −194.4 Dim414

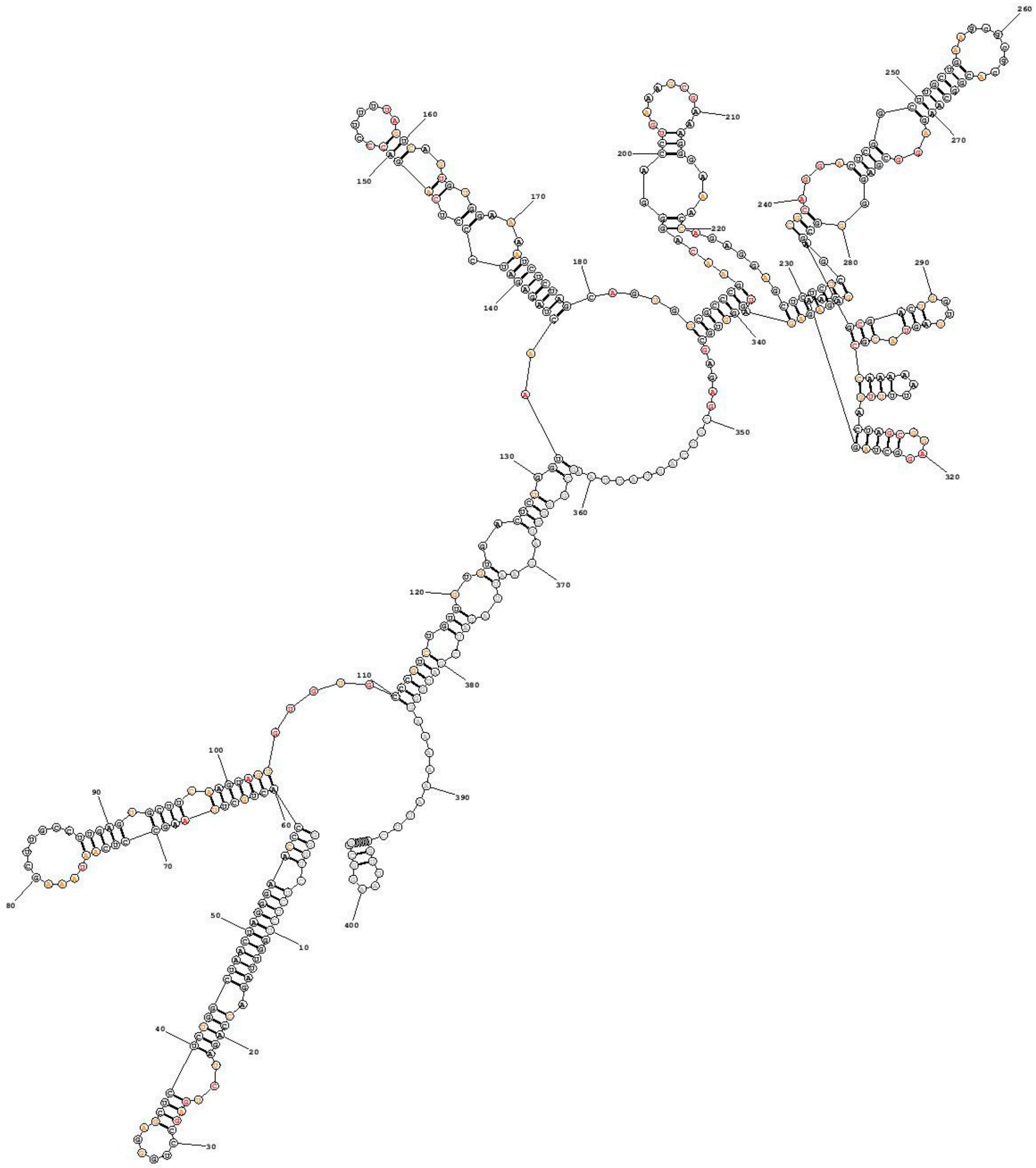

SHAPE >= 0.85
0.85 > SHAPE >= 0.4
0.4 > SHAPE
No Data

ENERGY = -194.4 Dim414

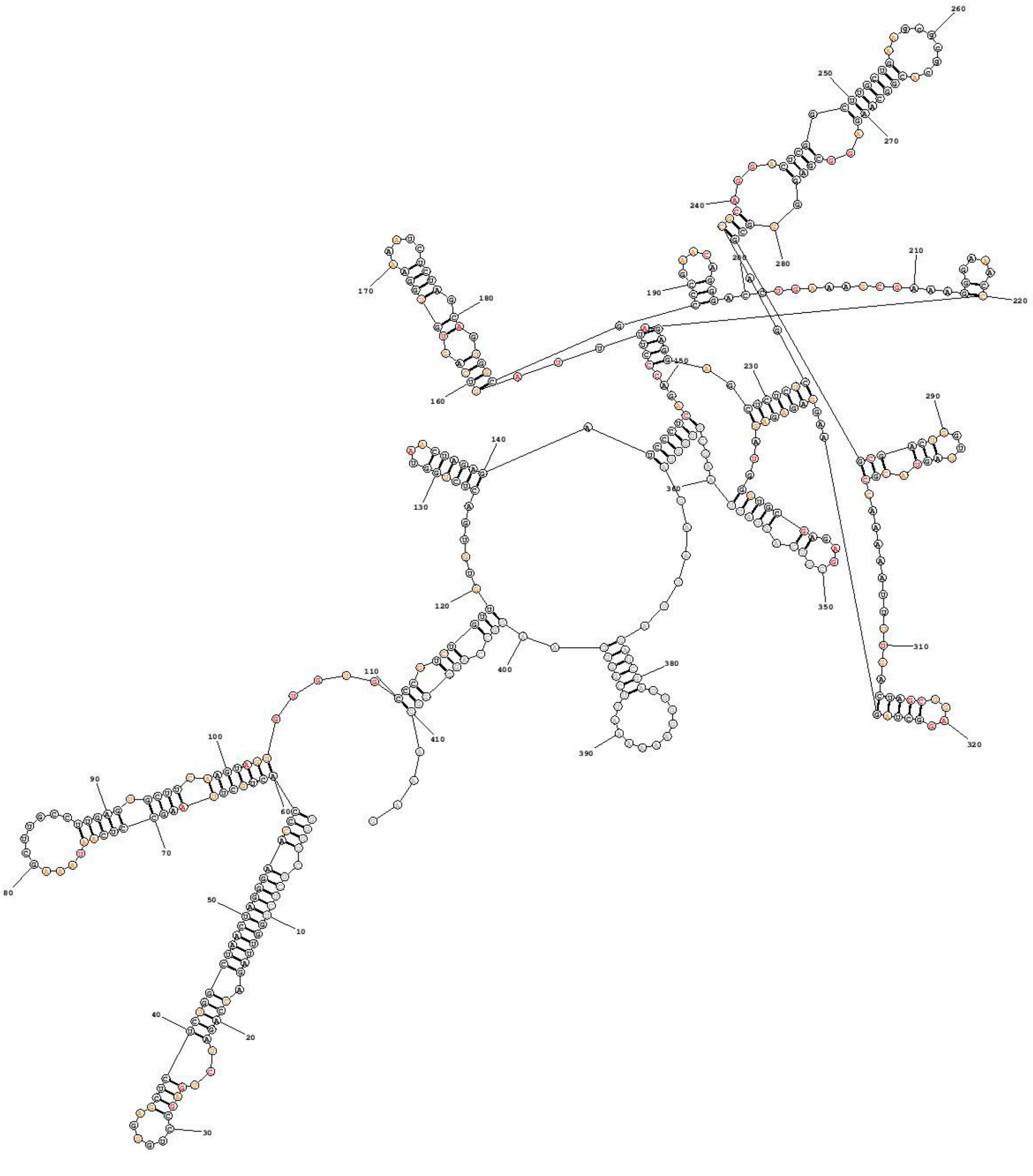

SHAPE >= 0.85

0.85 > SHAPE >= 0.4

0.4 > SHAPE

No Data

ENERGY = −194.3 Dim414

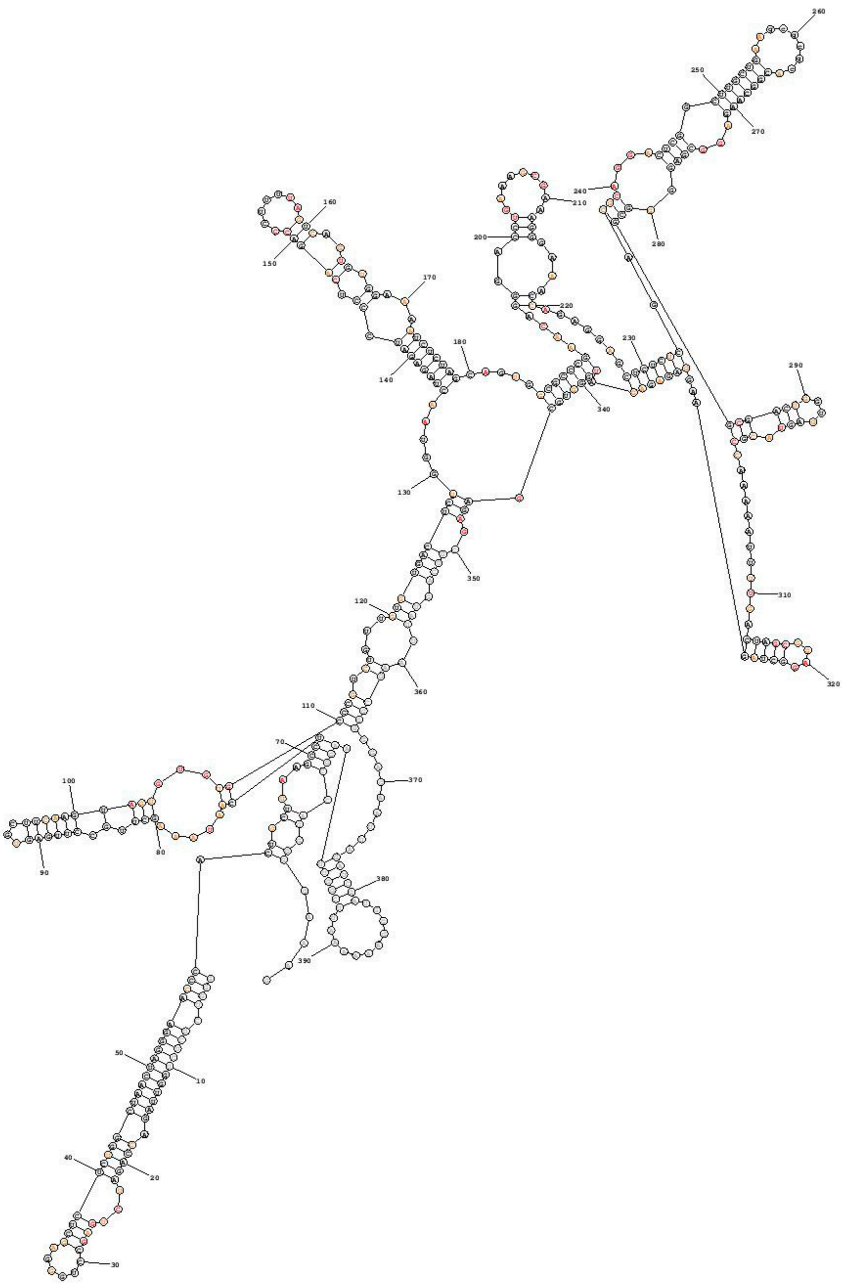

SHAPE >= 0.85

0.85 > SHAPE >= 0.4

0.4 > SHAPE

No Data

ENERGY = -193.9 Dim414

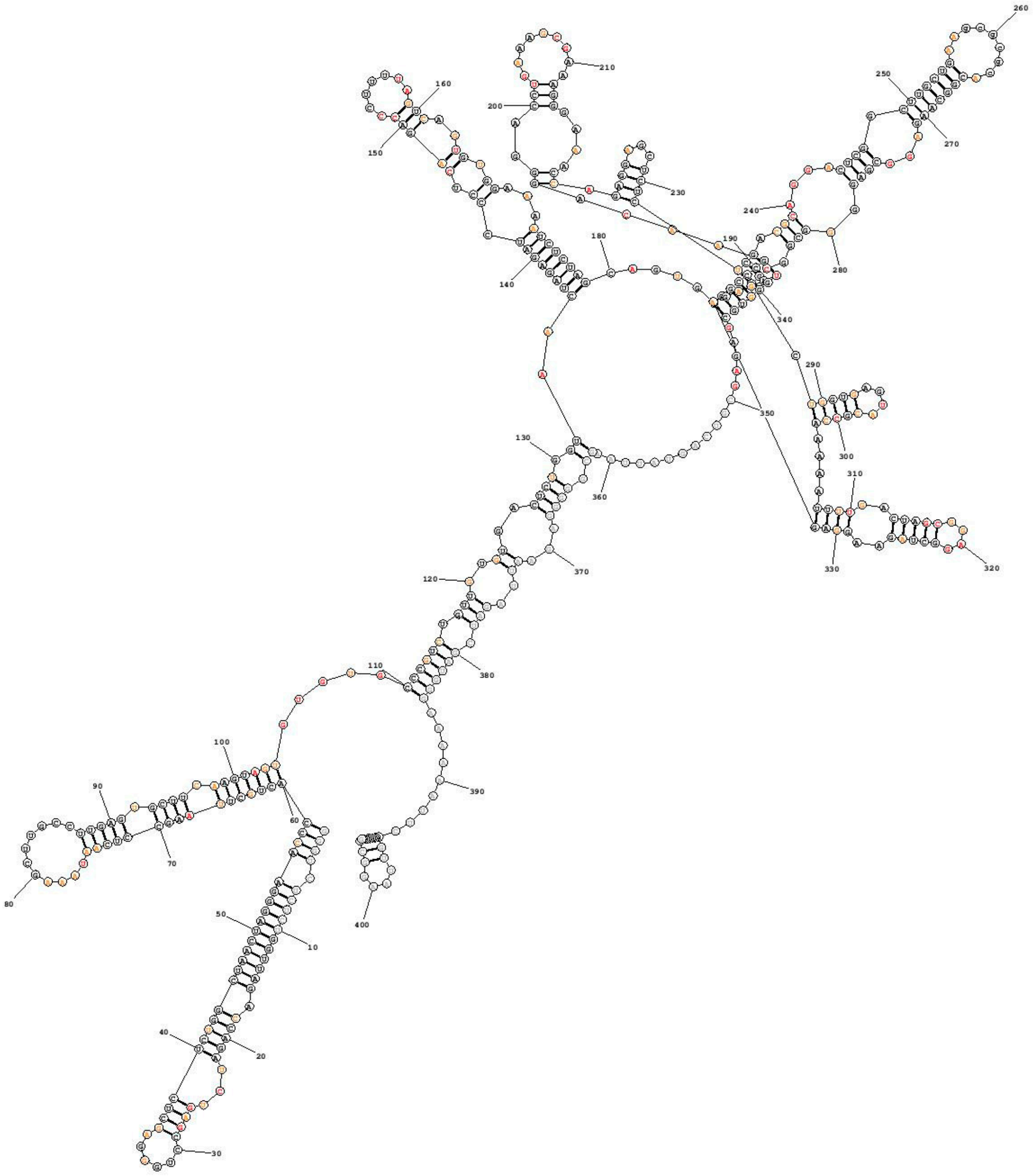

SHAPE >= 0.85

0.85 > SHAPE >= 0.4

0.4 > SHAPE

No Data

ENERGY = -193.9 Dim414

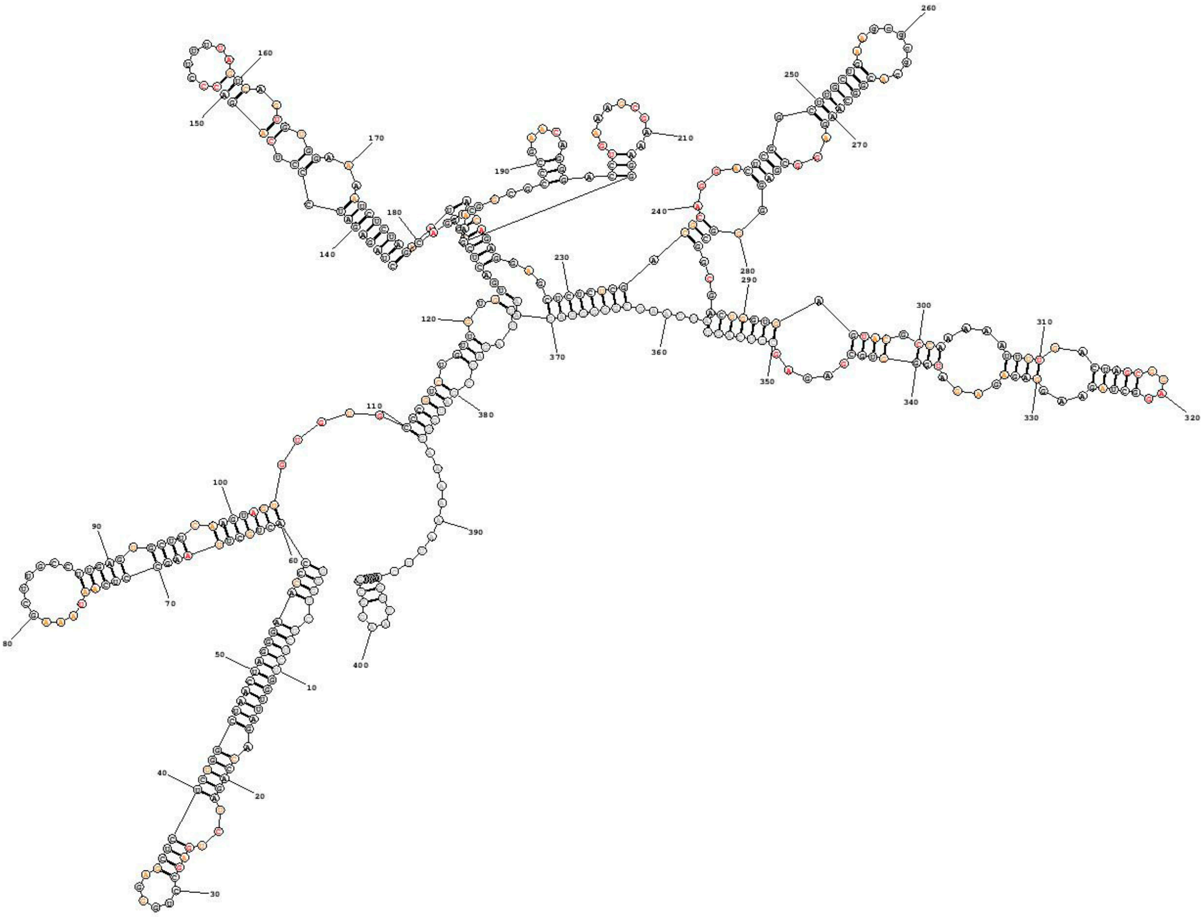

SHAPE >= 0.85

0.85 > SHAPE >= 0.4

0.4 > SHAPE

No Data

ENERGY = −193.7 Dim414

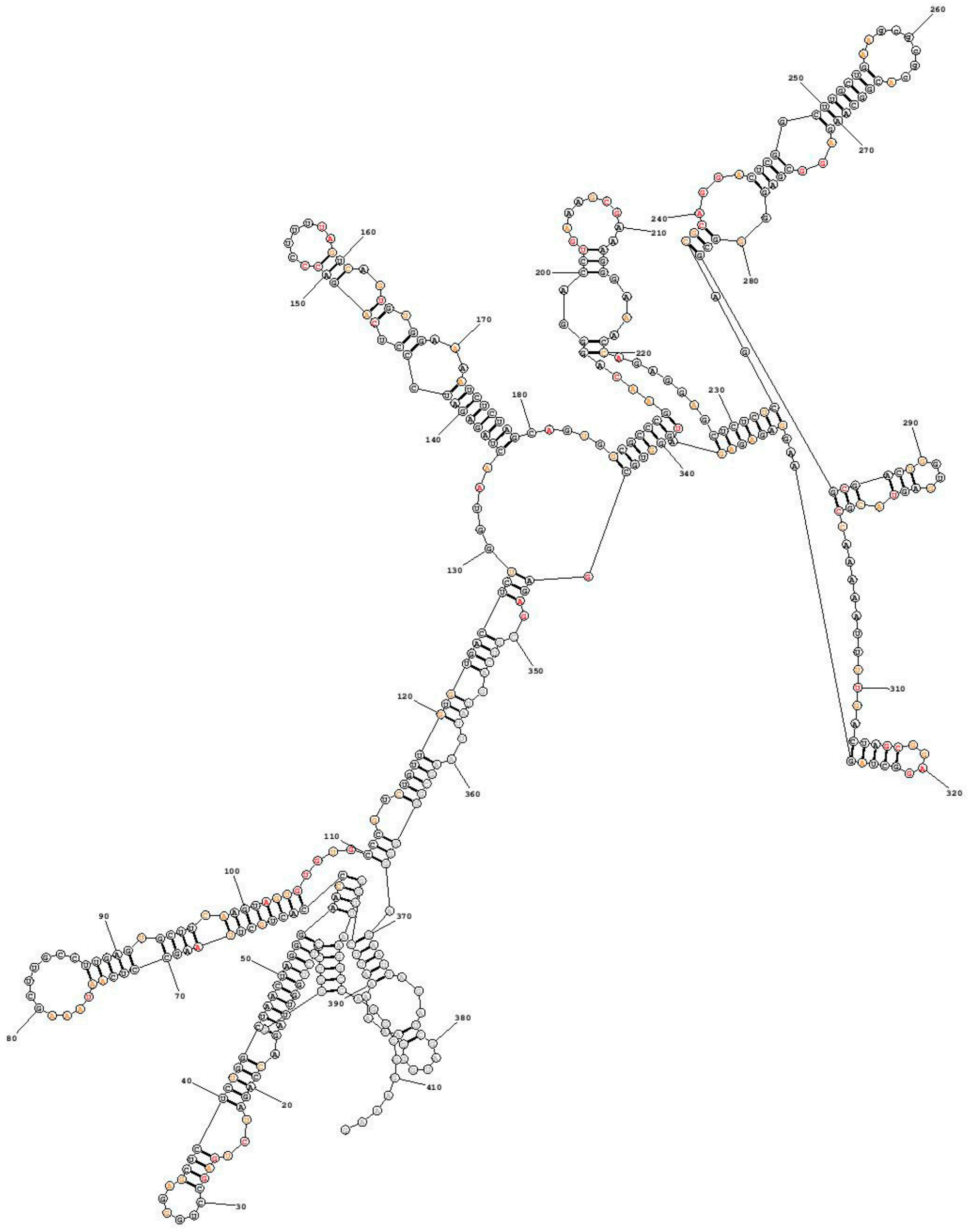

SHAPE >= 0.85

0.85 > SHAPE >= 0.4

0.4 > SHAPE

No Data

ENERGY = -193.6 Dim414

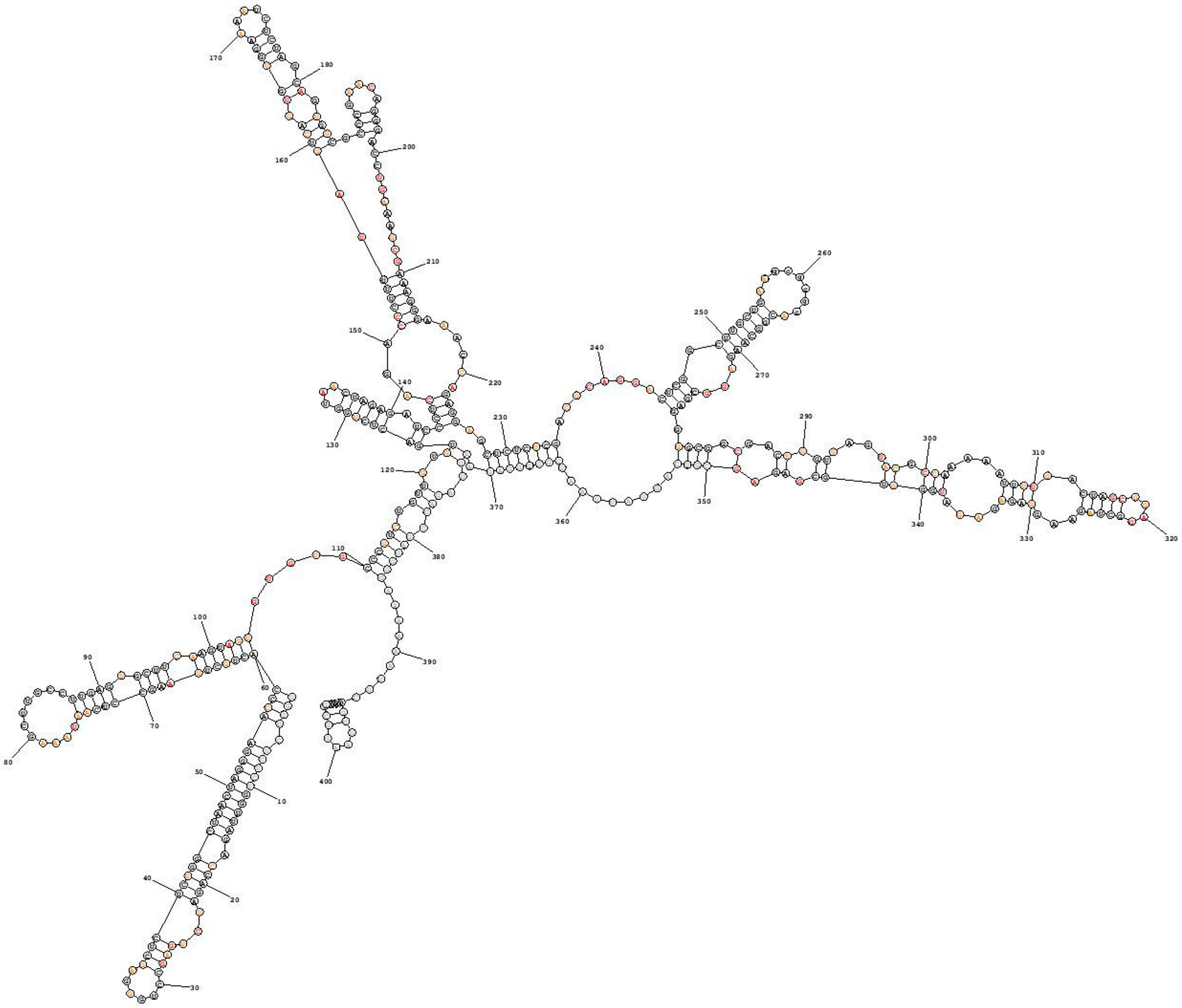

SHAPE >= 0.85
0.85 > SHAPE >= 0.4
0.4 > SHAPE
No Data

ENERGY = -193.4 Dim414

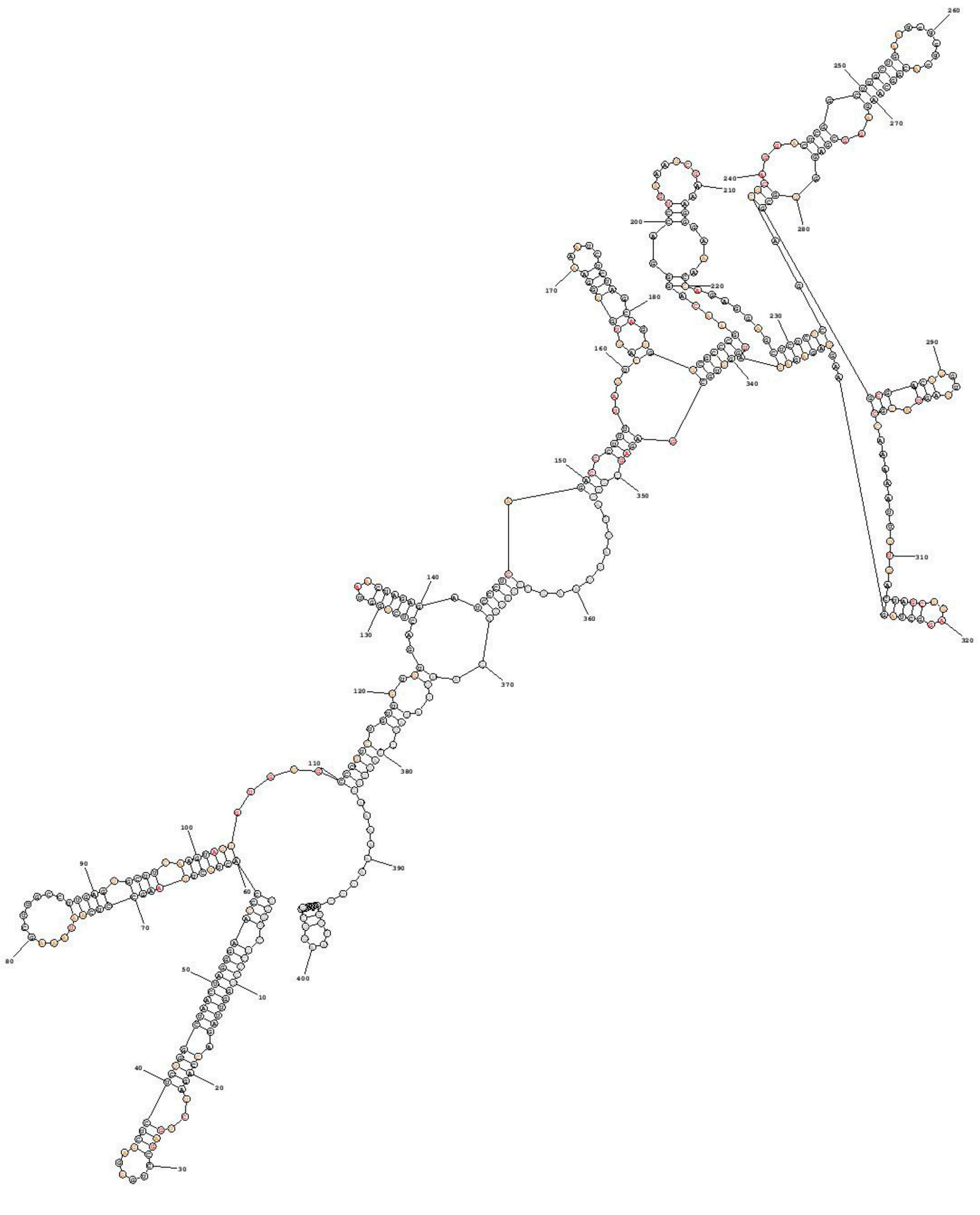

SHAPE >= 0.85

0.85 > SHAPE >= 0.4

0.4 > SHAPE

No Data

ENERGY = −193.0 Dim414

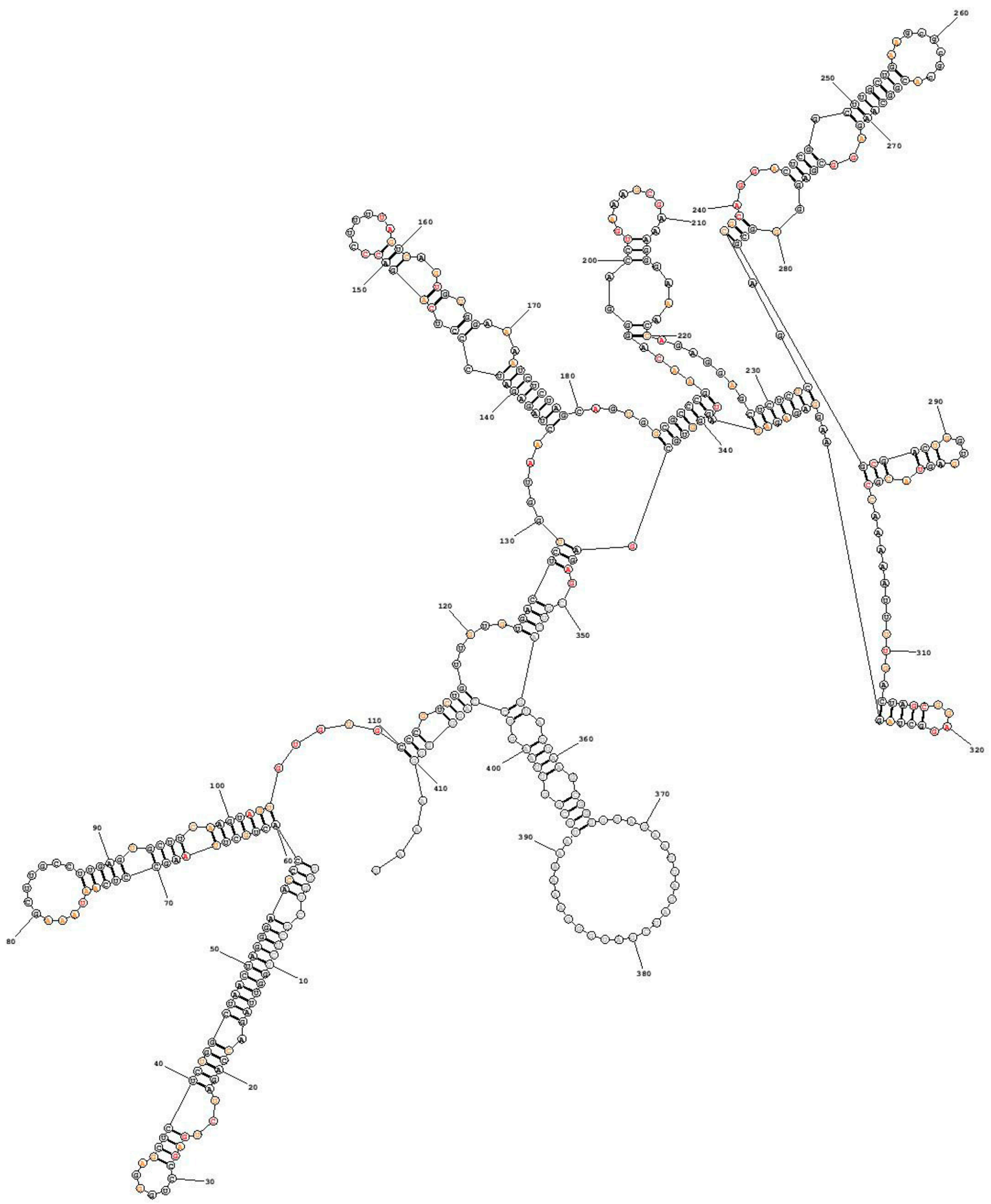

SHAPE >= 0.85

0.85 > SHAPE >= 0.4

0.4 > SHAPE

No Data

ENERGY = −193.0 Dim414

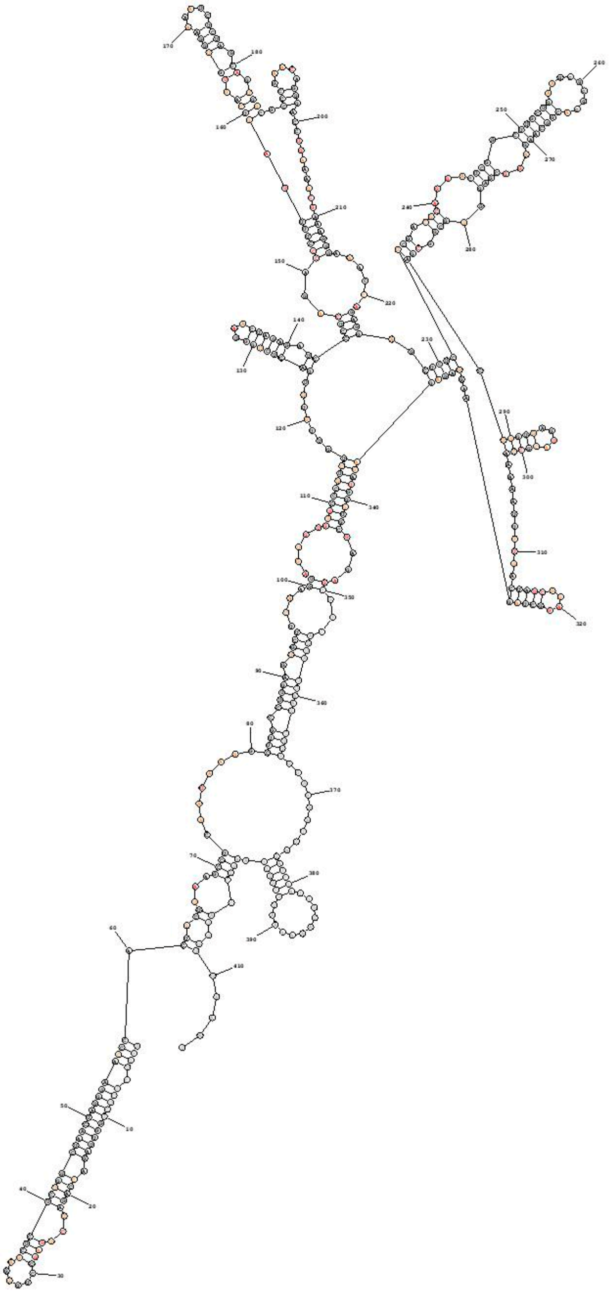

SHAPE >= 0.85
0.85 > SHAPE >= 0.4
0.4 > SHAPE
No Data

ENERGY = -192.9 Dim414

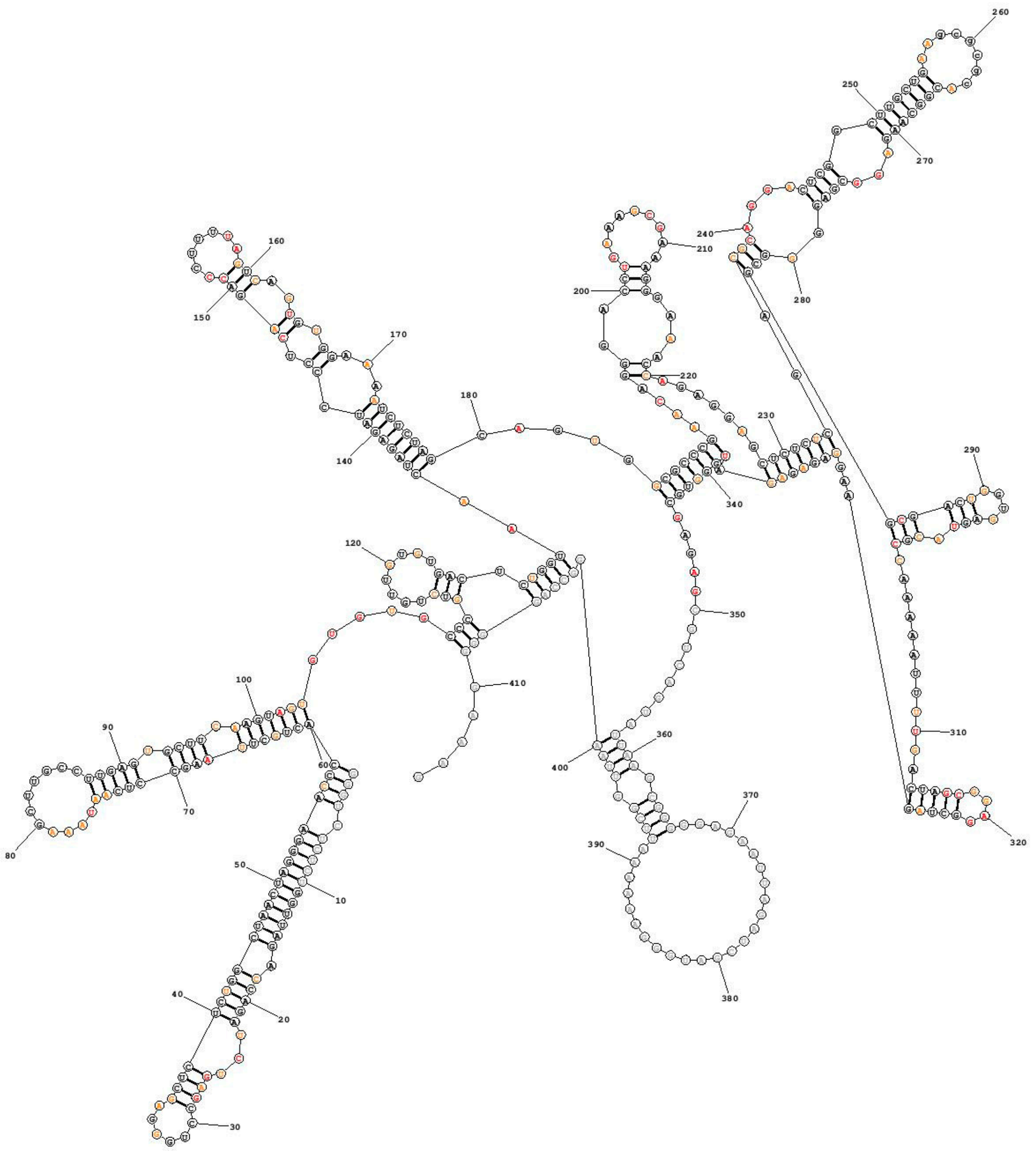

SHAPE >= 0.85

0.85 > SHAPE >= 0.4

0.4 > SHAPE

No Data

ENERGY = -192.8 Dim414

Supplementary figure 7 - Dimer 414 + Gag

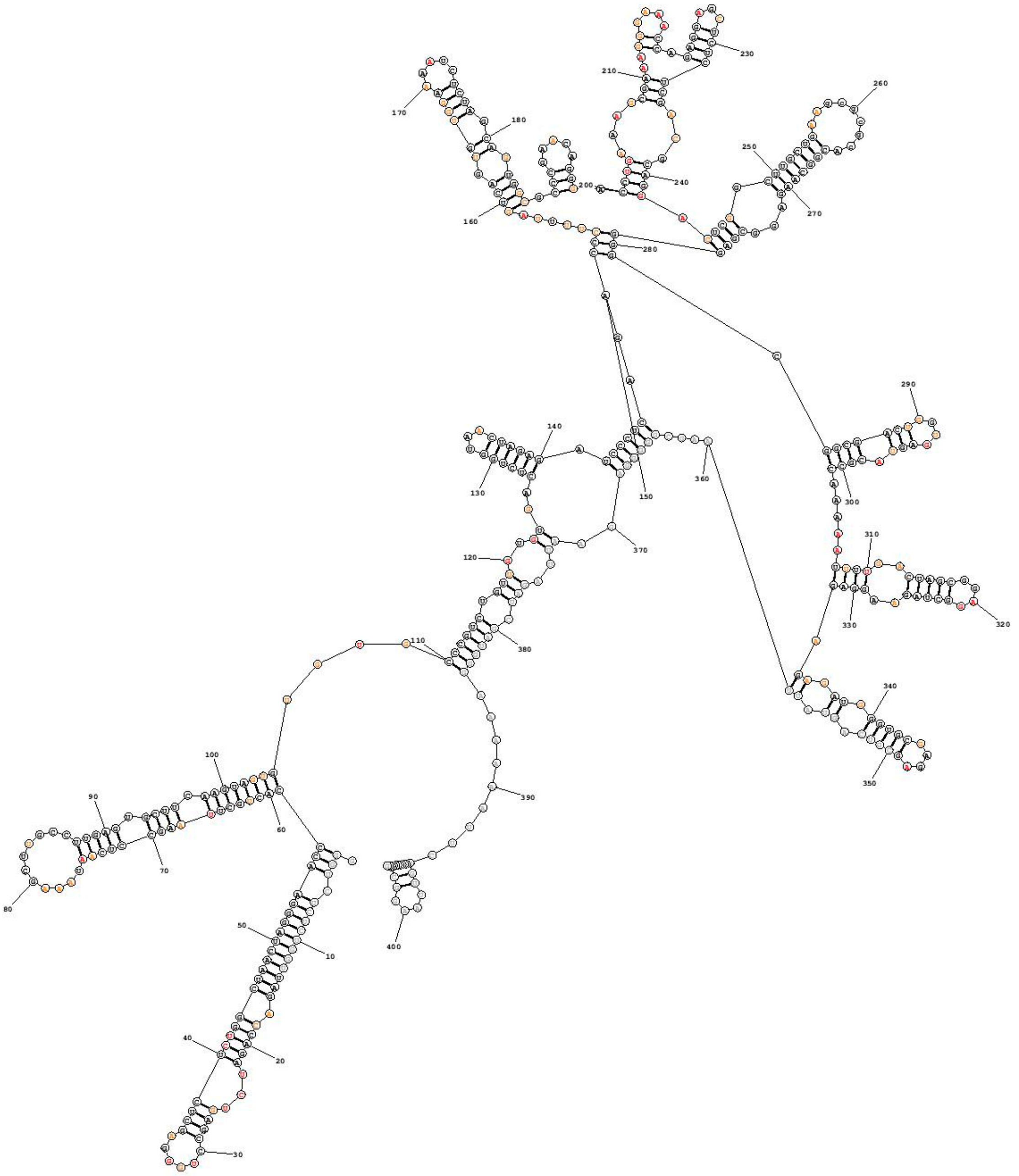

SHAPE >= 0.85

0.85 > SHAPE >= 0.4

0.4 > SHAPE

No Data

ENERGY = -226.5 DimGag414

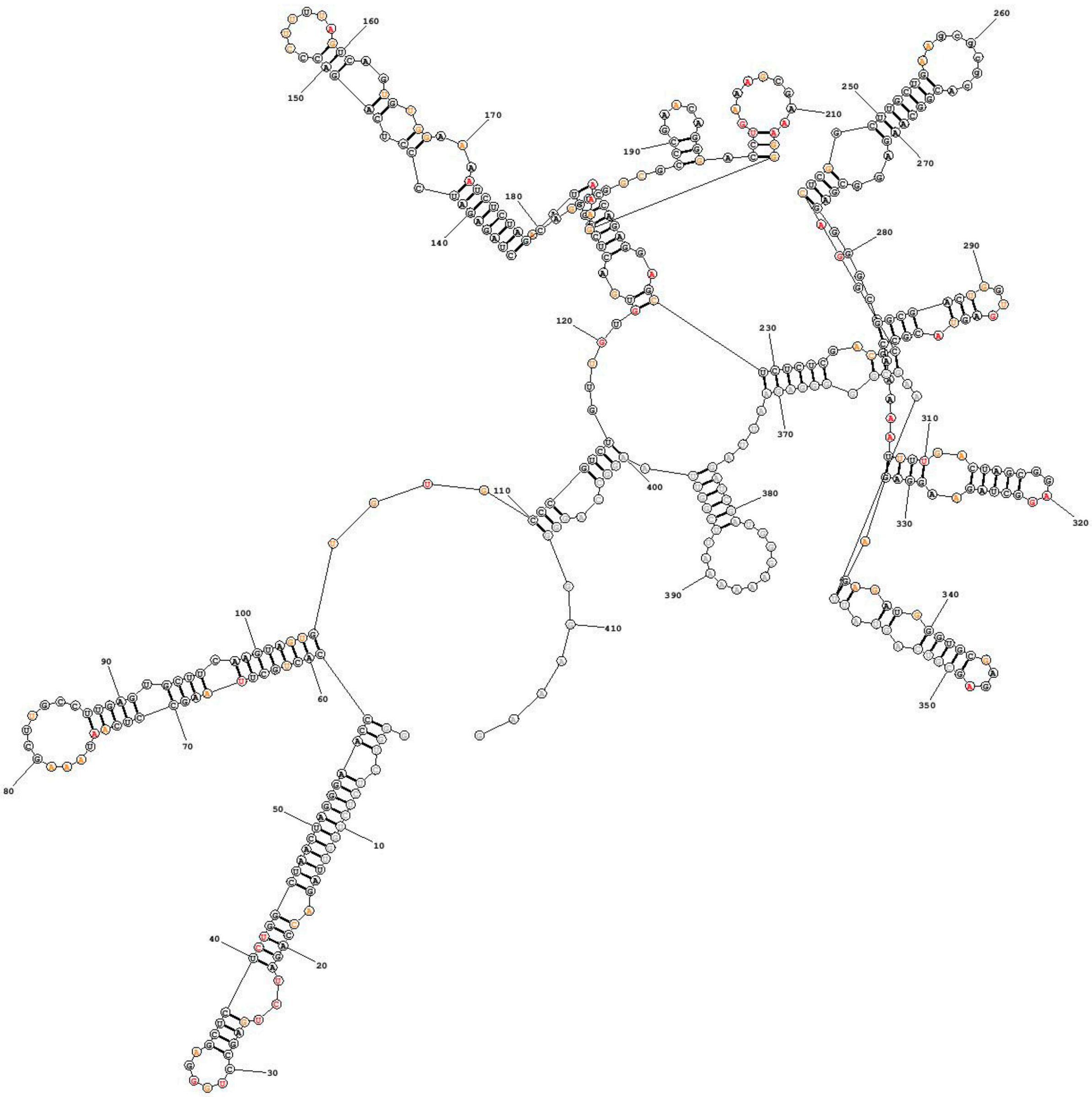

SHAPE >= 0.85

0.85 > SHAPE >= 0.4

0.4 > SHAPE

No Data

ENERGY = −226.4 DimGag414

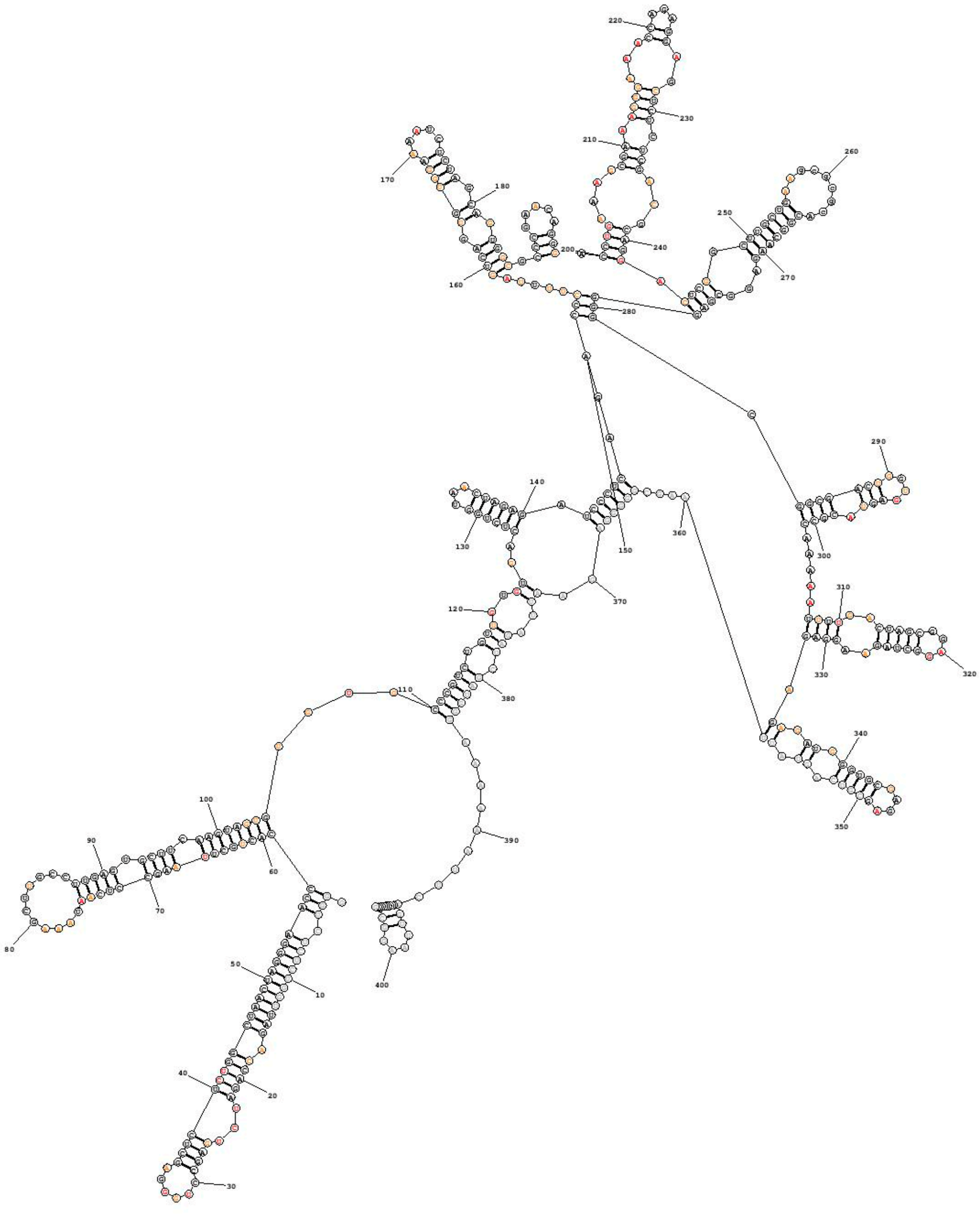

SHAPE >= 0.85
0.85 > SHAPE >= 0.4
0.4 > SHAPE
No Data

ENERGY = -226.1 DimGag414

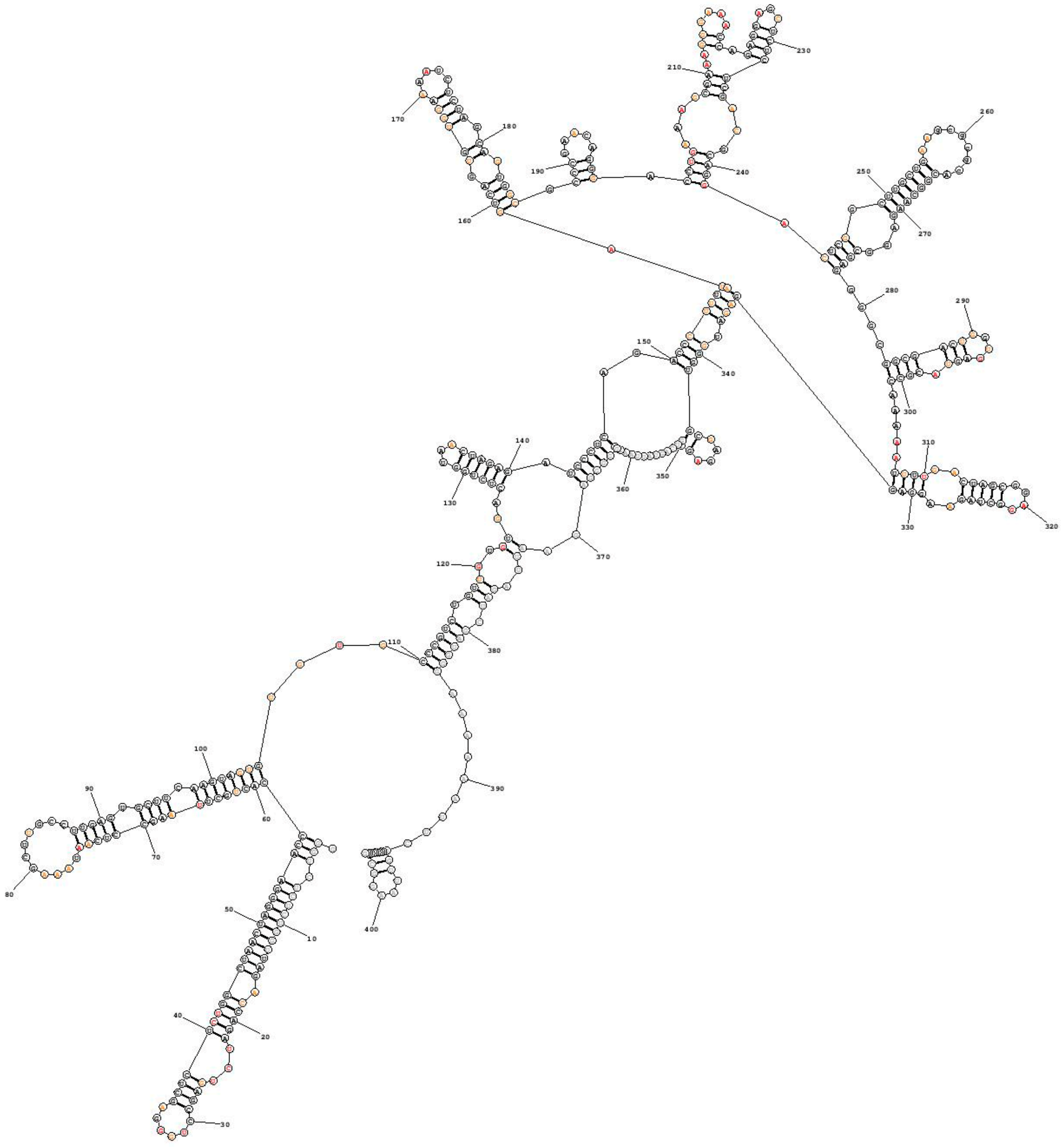

SHAPE >= 0.85

0.85 > SHAPE >= 0.4

0.4 > SHAPE

No Data

ENERGY = −225.6 DimGag414

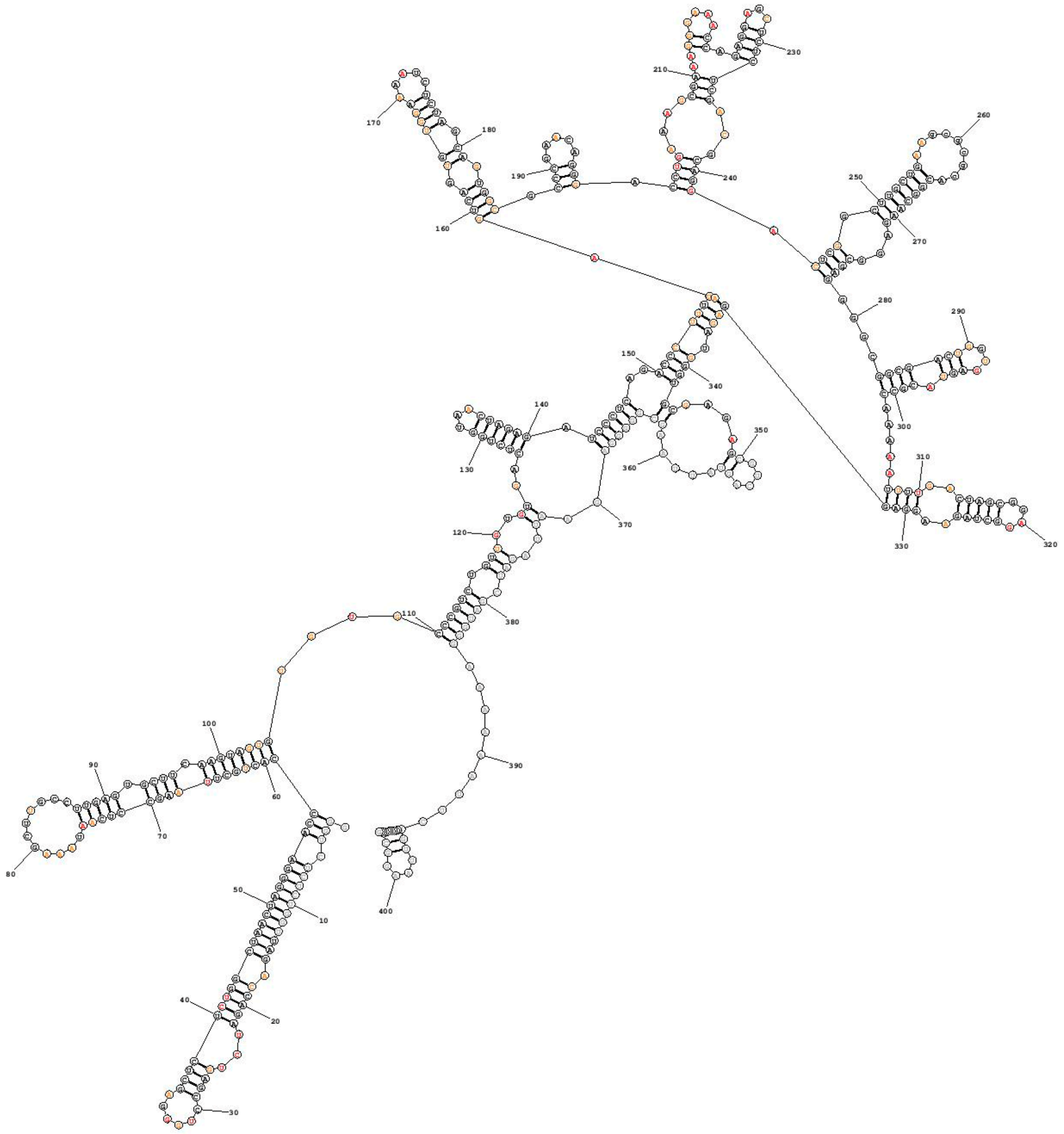

SHAPE >= 0.85
0.85 > SHAPE >= 0.4
0.4 > SHAPE
No Data

ENERGY = -225.4 DimGag414

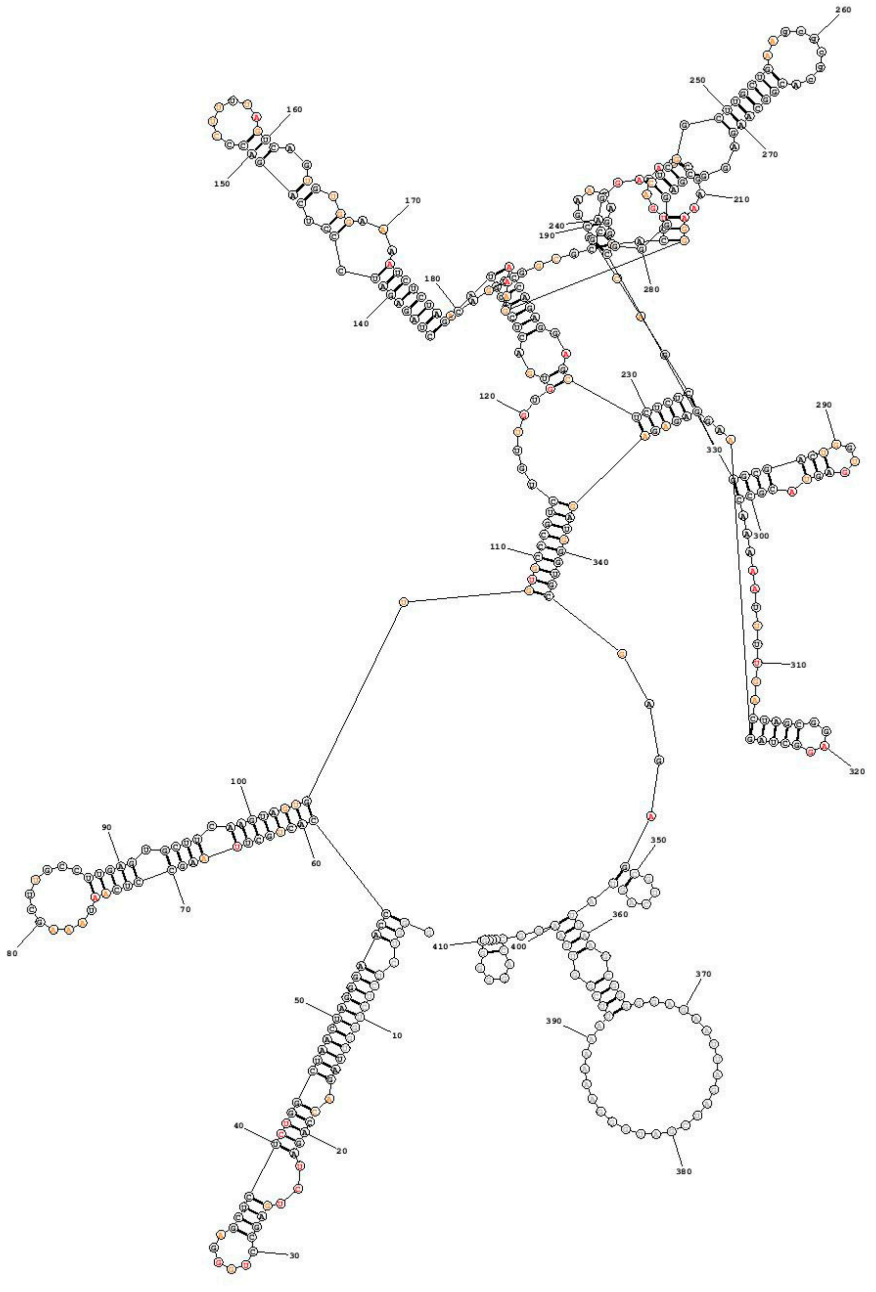

SHAPE >= 0.85
0.85 > SHAPE >= 0.4
0.4 > SHAPE
No Data
ENERGY = −225.4 DimGag414

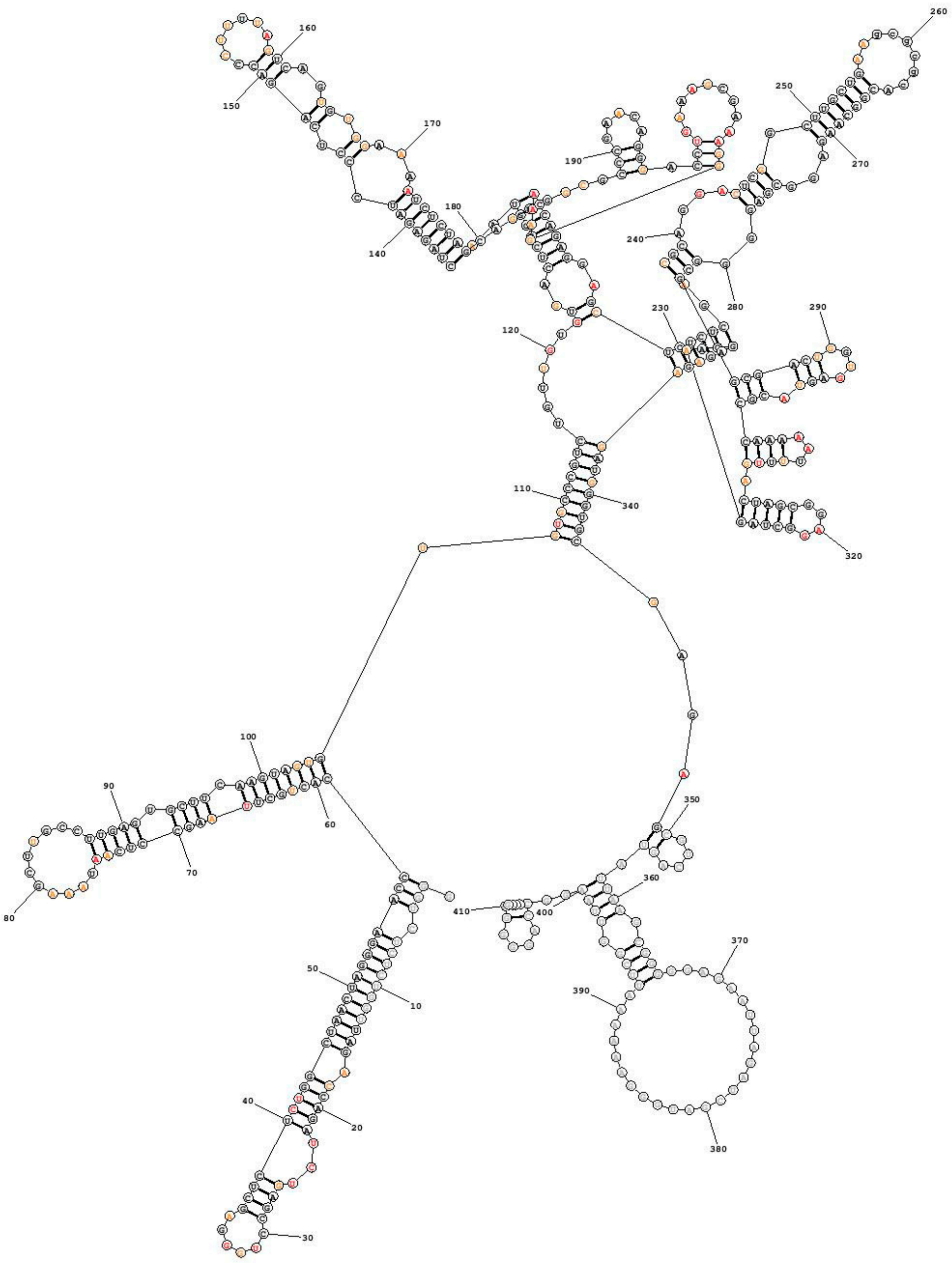

SHAPE >= 0.85

0.85 > SHAPE >= 0.4

0.4 > SHAPE

No Data

ENERGY = -225.4   DimGag414

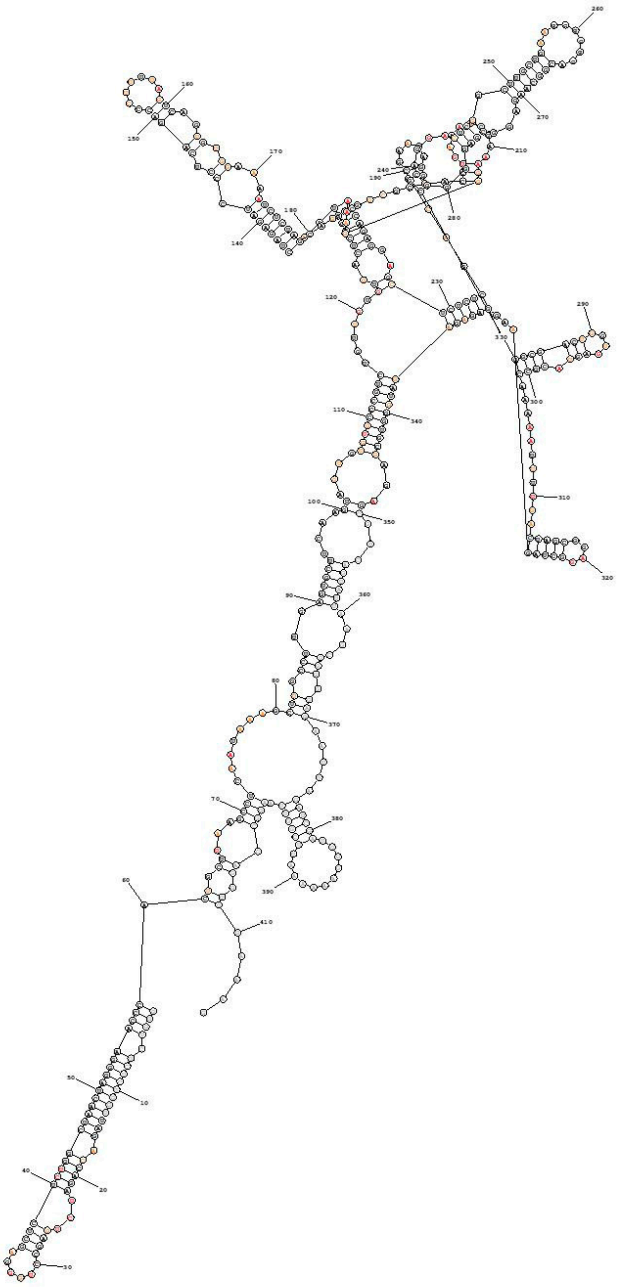

SHAPE >= 0.85
0.85 > SHAPE >= 0.4
0.4 > SHAPE
No Data

ENERGY = -225.0 DimGag414

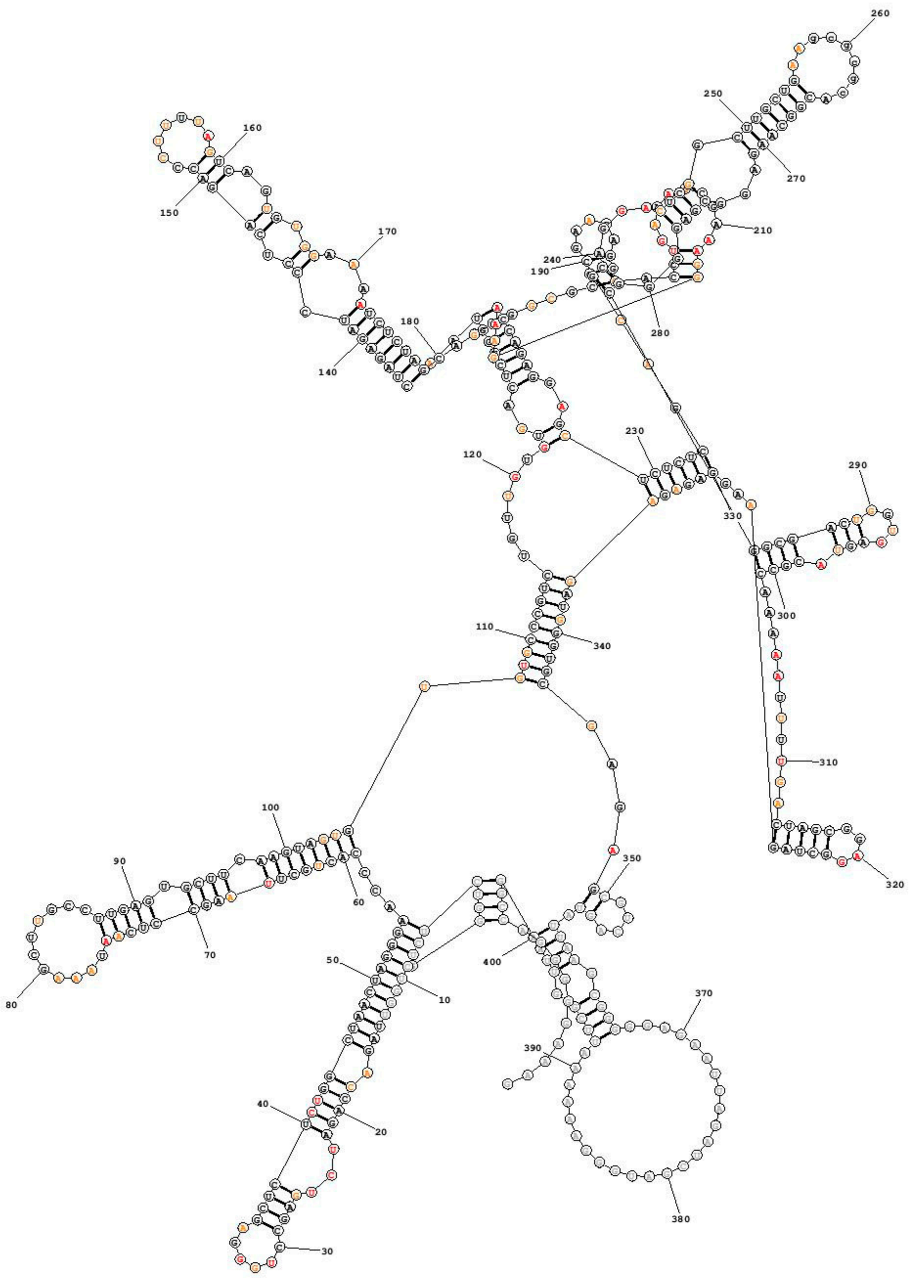

SHAPE >= 0.85
0.85 > SHAPE >= 0.4
0.4 > SHAPE
No Data

ENERGY = −224.8 DimGag414

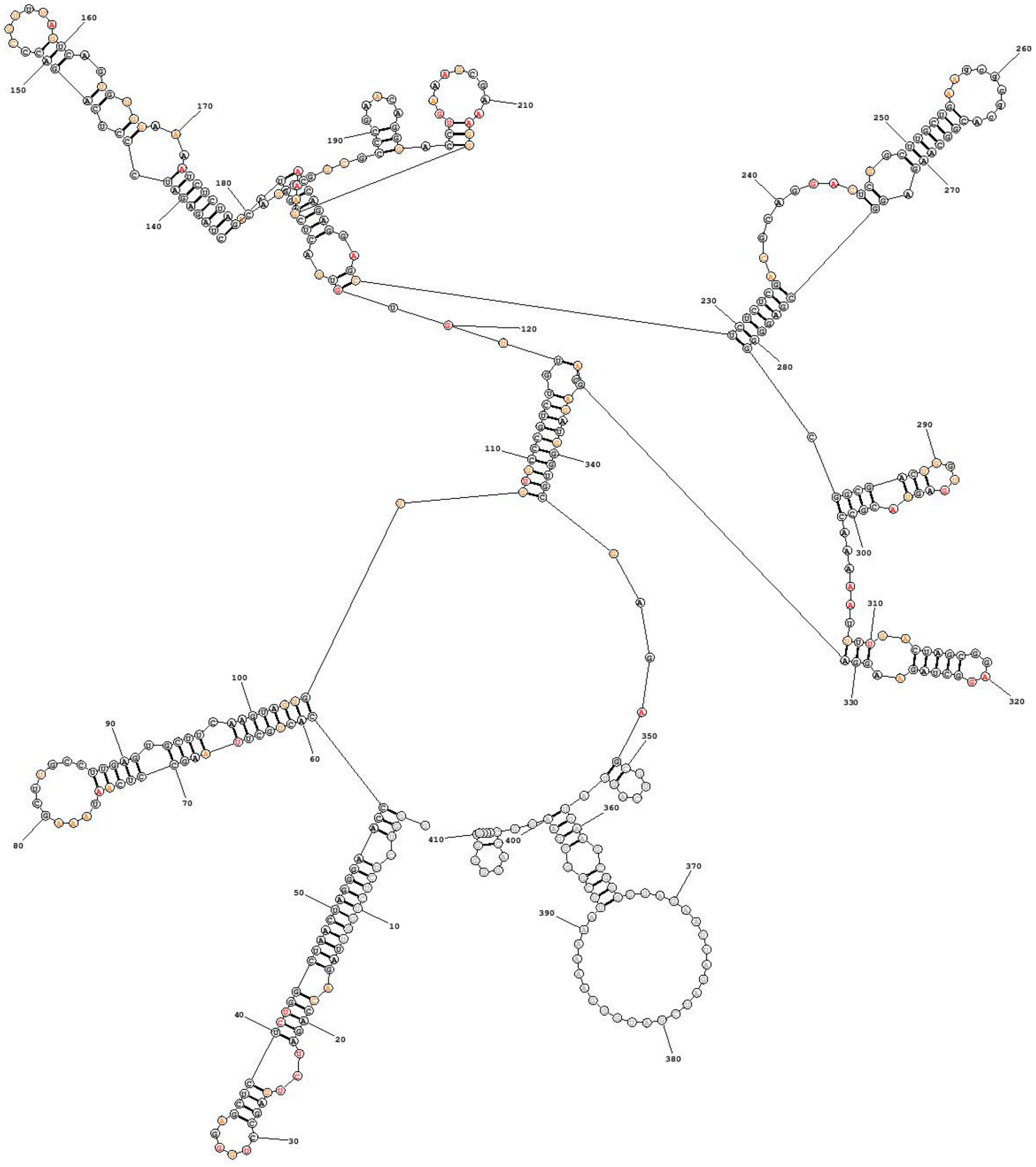

SHAPE >= 0.85
0.85 > SHAPE >= 0.4
0.4 > SHAPE
No Data

ENERGY = −224.7   DimGag414

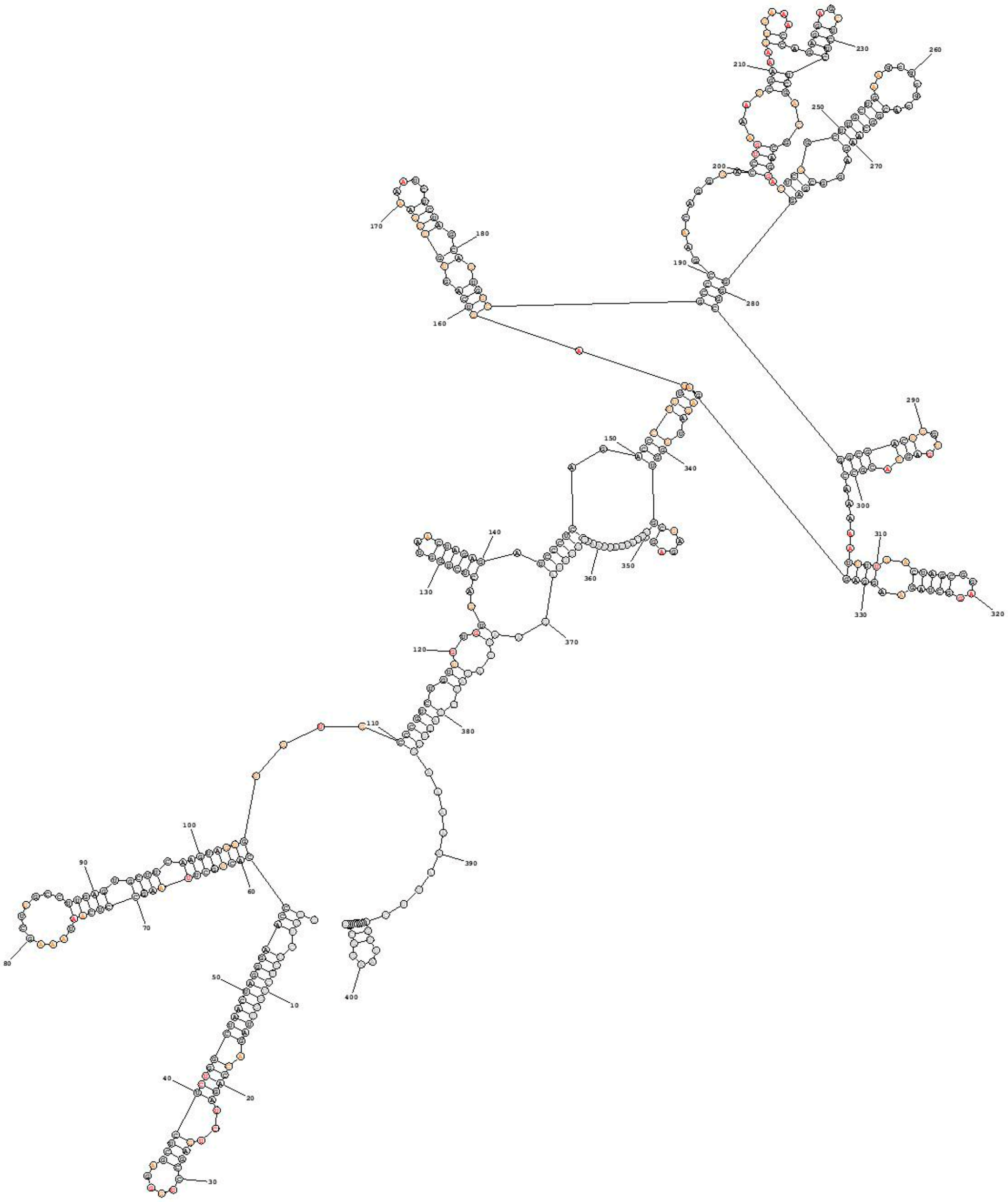

SHAPE >= 0.85

0.85 > SHAPE >= 0.4

0.4 > SHAPE

No Data

ENERGY = −224.6 DimGag414

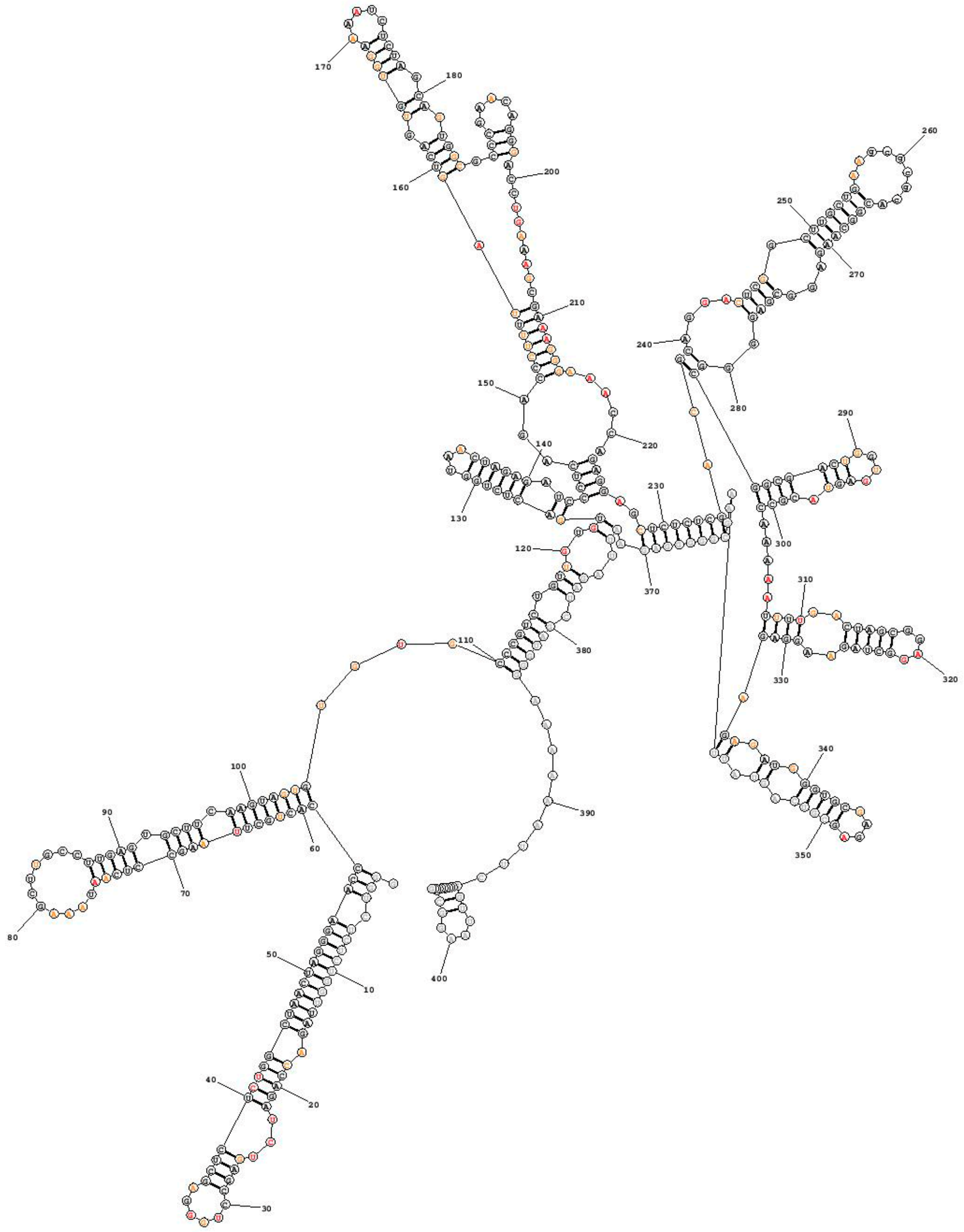

SHAPE >= 0.85

0.85 > SHAPE >= 0.4

0.4 > SHAPE

No Data

ENERGY = −224.5 DimGag414

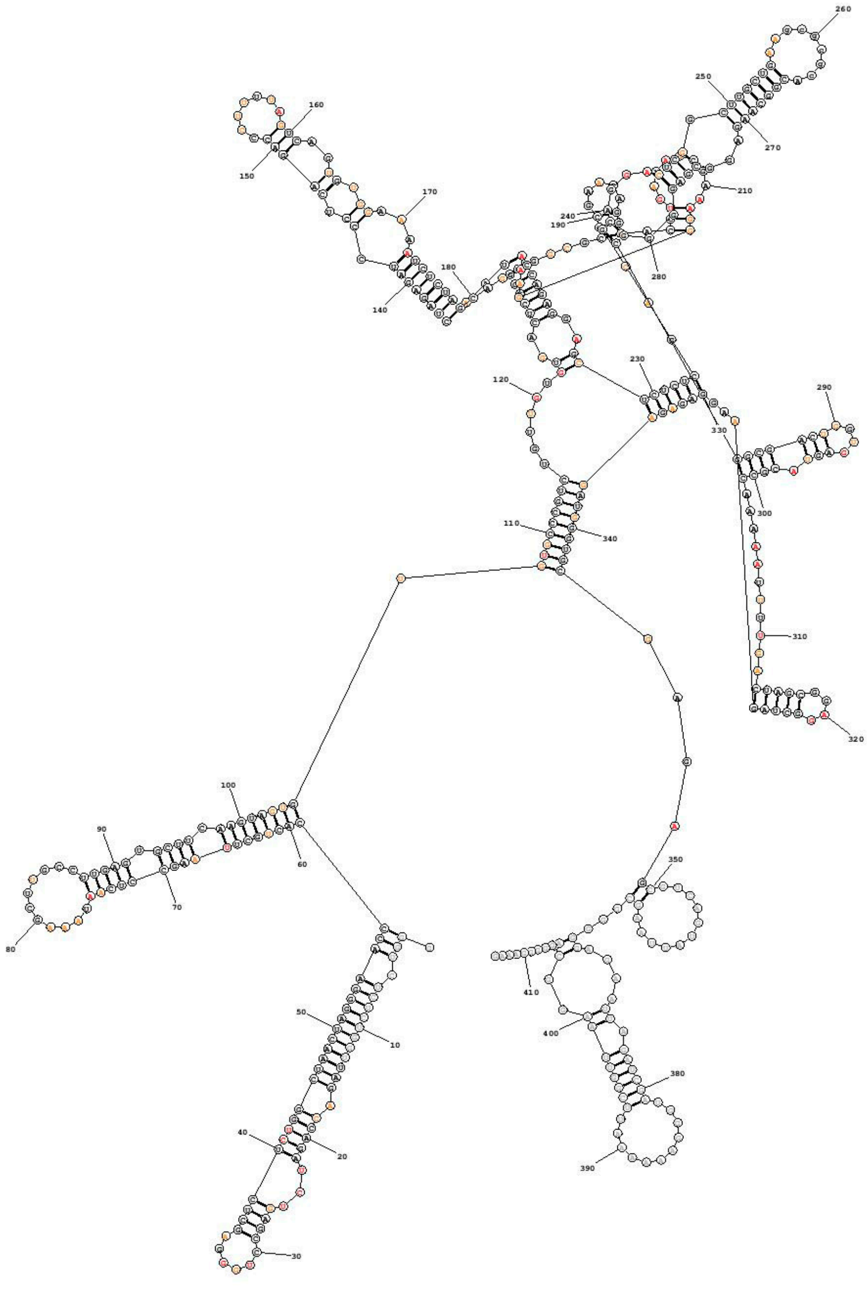

SHAPE >= 0.85

0.85 > SHAPE >= 0.4

0.4 > SHAPE

No Data

ENERGY = −224.4 DimGag414

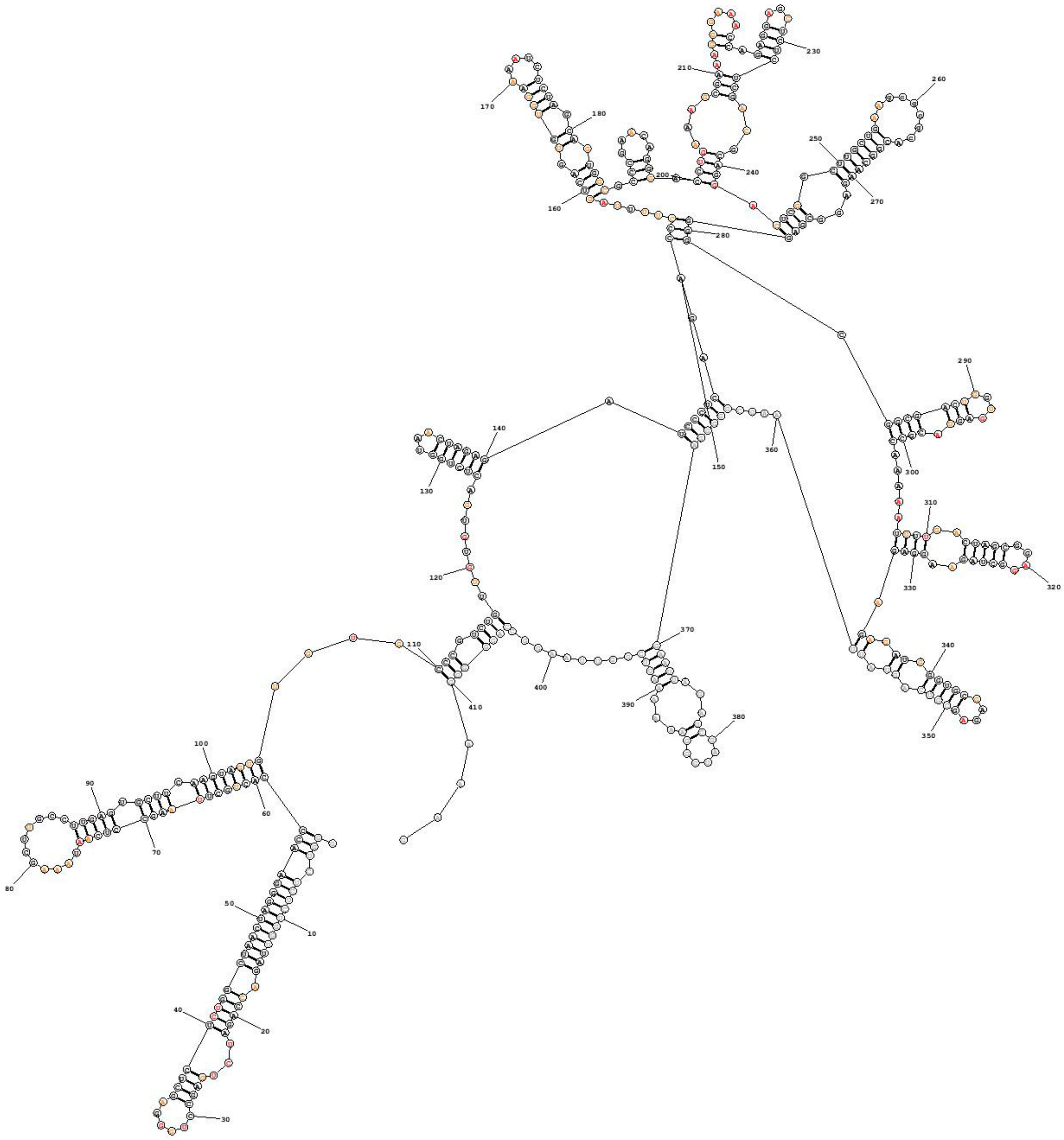

SHAPE >= 0.85
0.85 > SHAPE >= 0.4
0.4 > SHAPE
No Data

ENERGY = -224.3 DimGag414

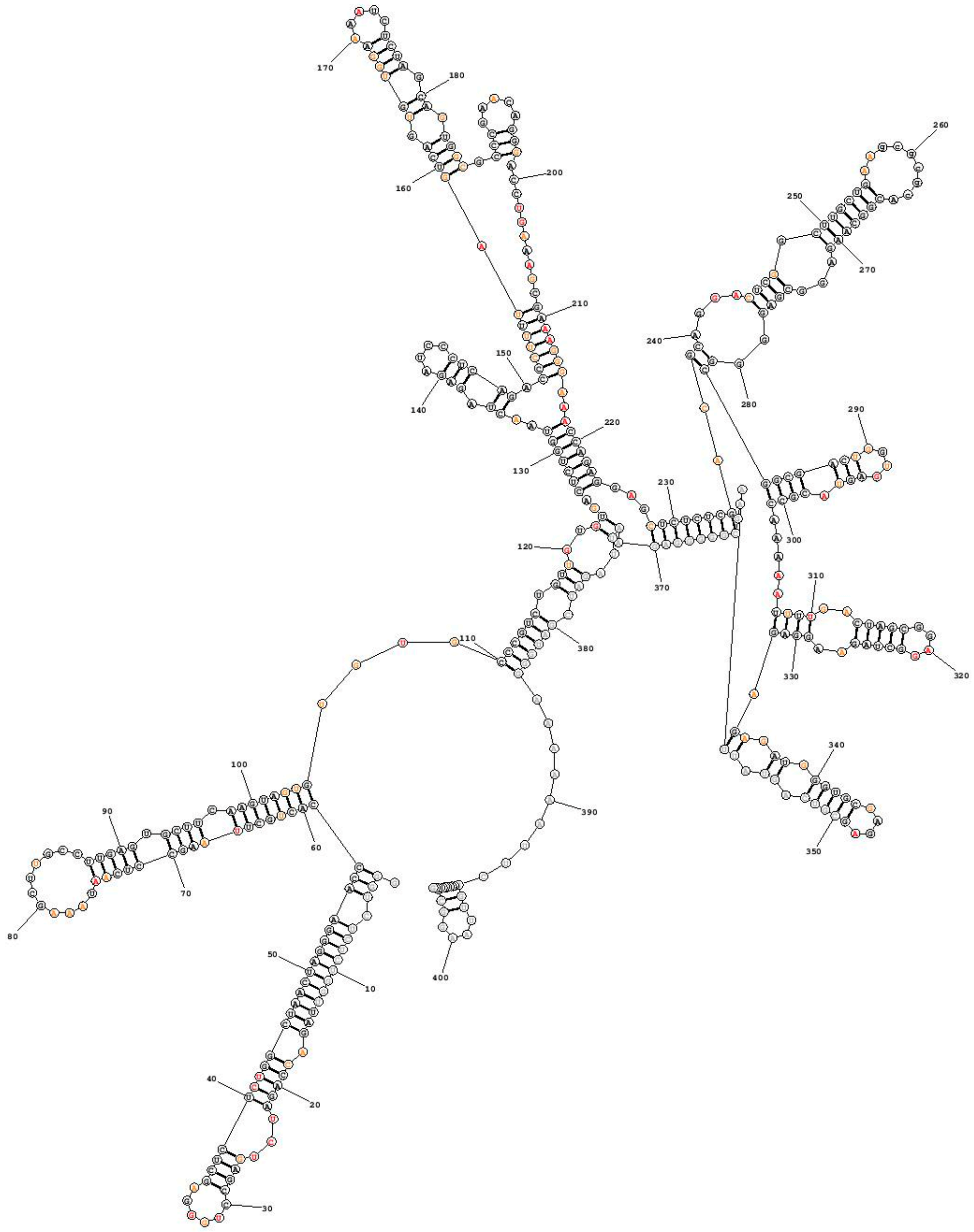

SHAPE >= 0.85
0.85 > SHAPE >= 0.4
0.4 > SHAPE
No Data

ENERGY = −224.2 DimGag414

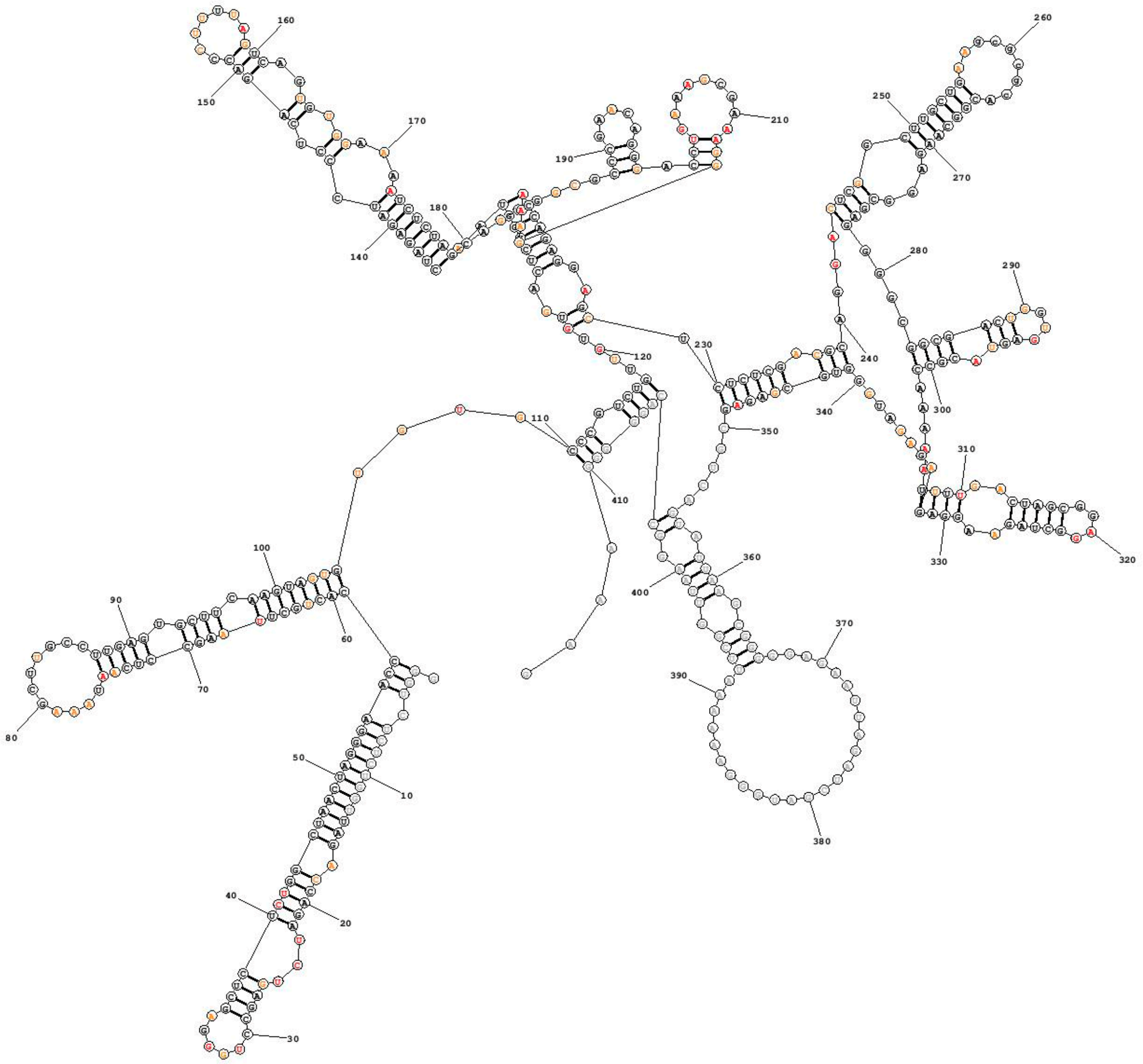

SHAPE >= 0.85
0.85 > SHAPE >= 0.4
0.4 > SHAPE
No Data

ENERGY = -224.1   DimGag414

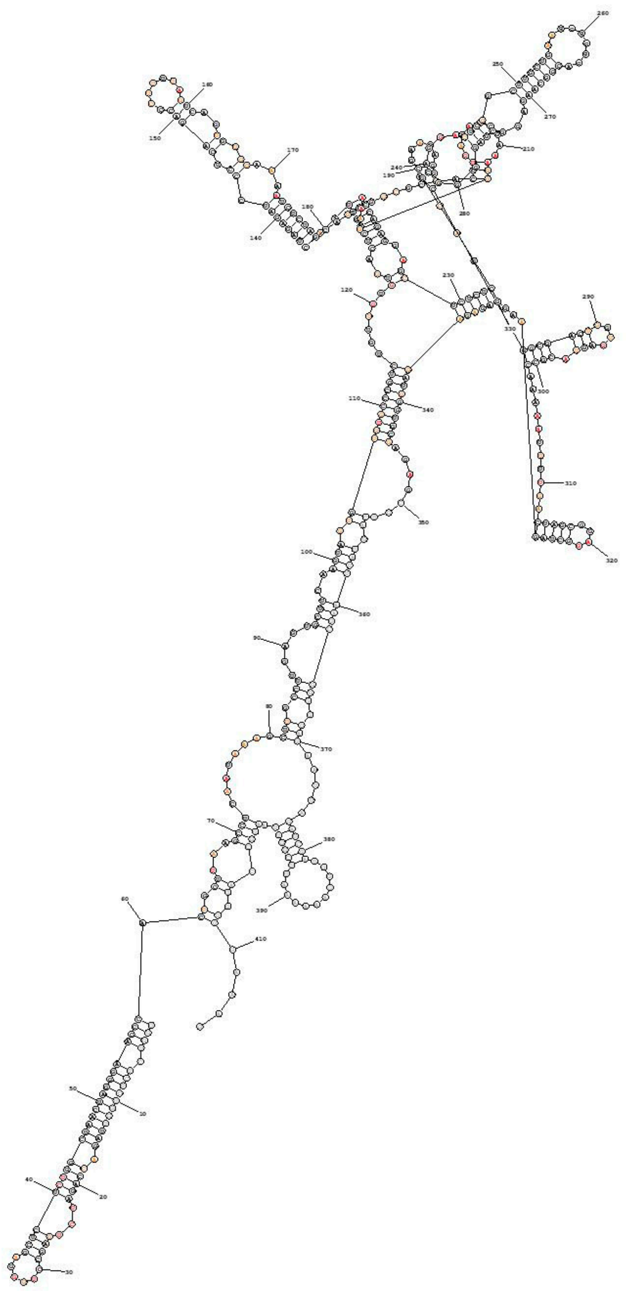

SHAPE >= 0.85
0.85 > SHAPE >= 0.4
0.4 > SHAPE
No Data

ENERGY = −224.0   DimGag414

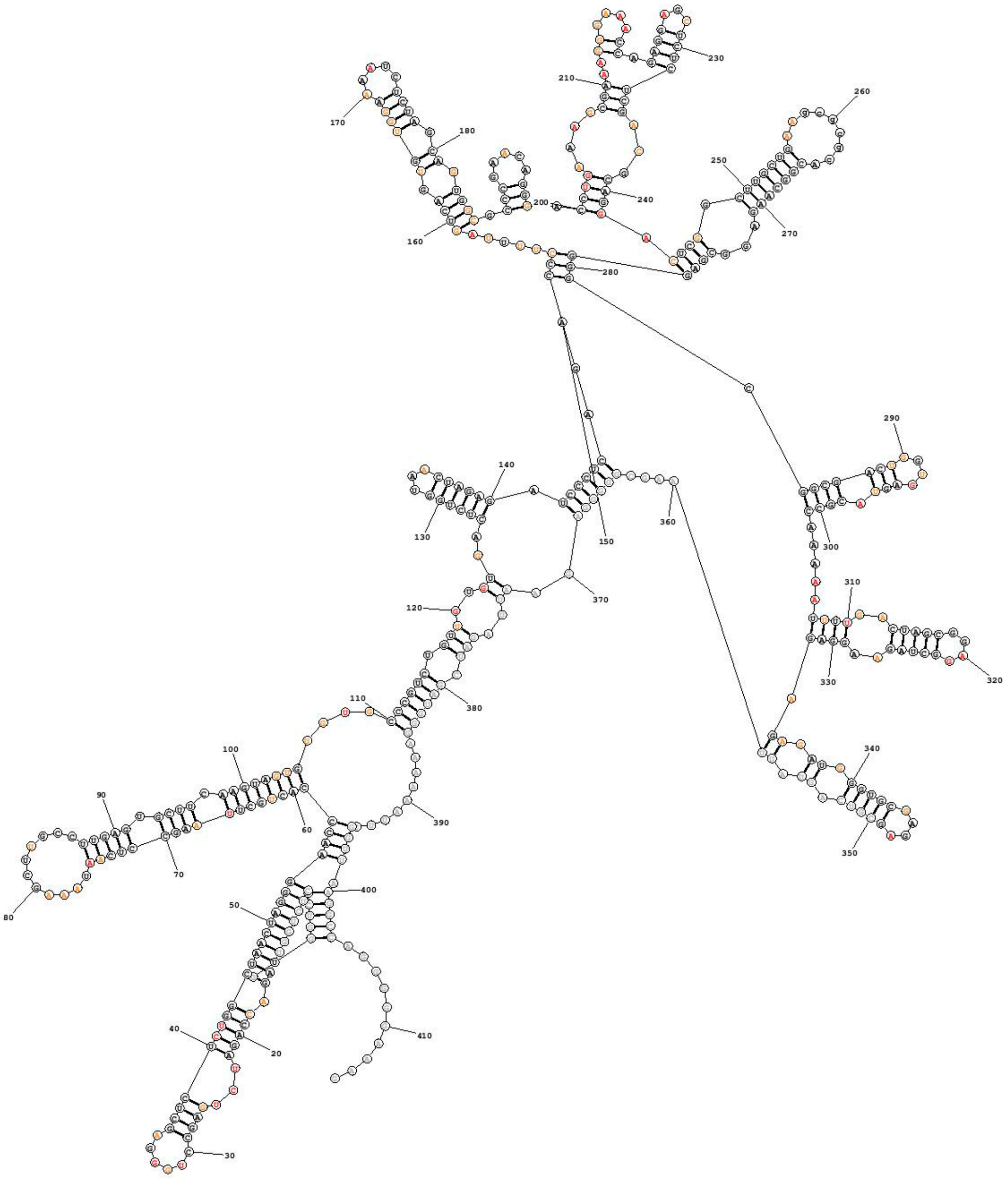

SHAPE >= 0.85

0.85 > SHAPE >= 0.4

0.4 > SHAPE

No Data

ENERGY = −223.9 DimGag414

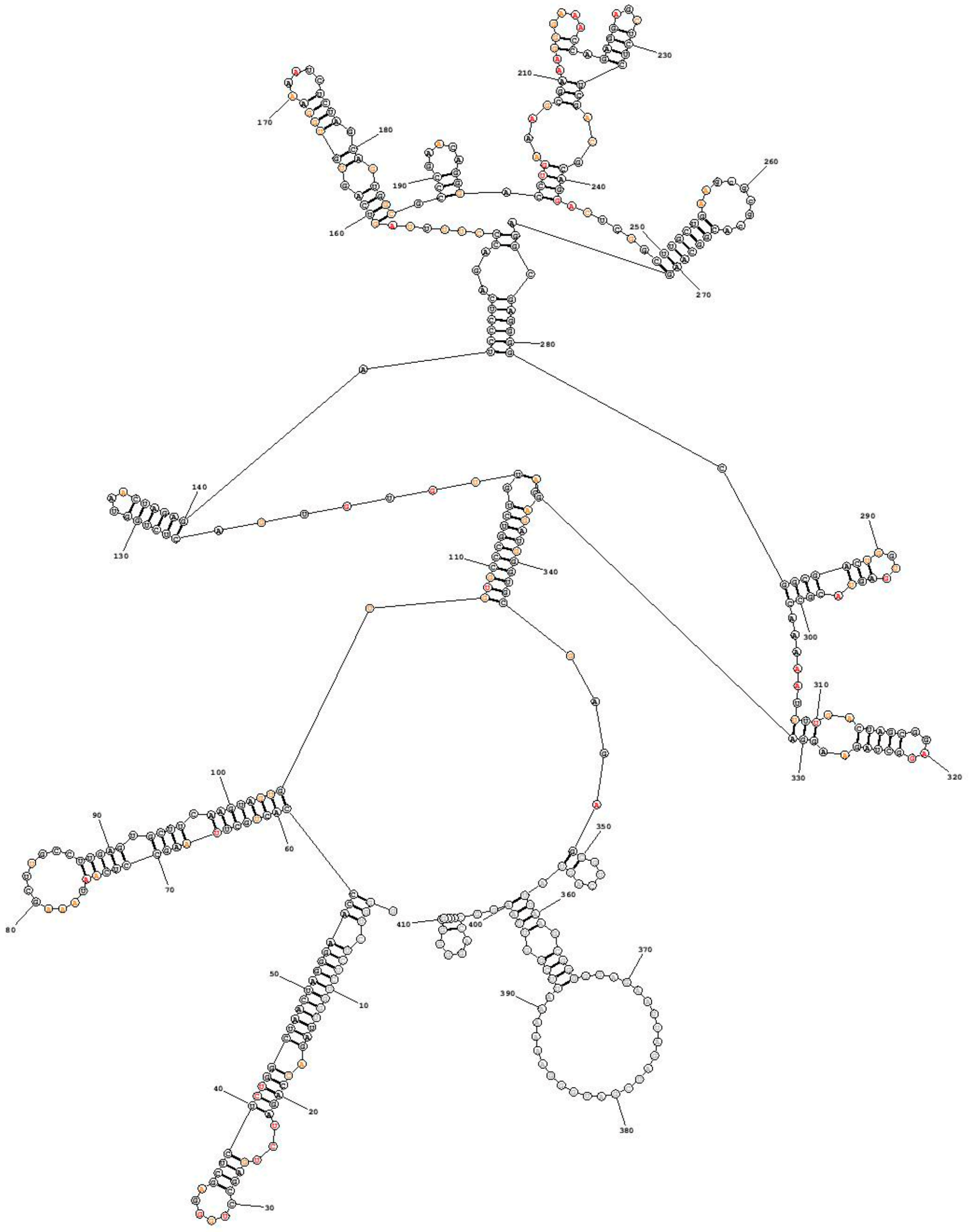

SHAPE >= 0.85

0.85 > SHAPE >= 0.4

0.4 > SHAPE

No Data

ENERGY = −223.5 DimGag414

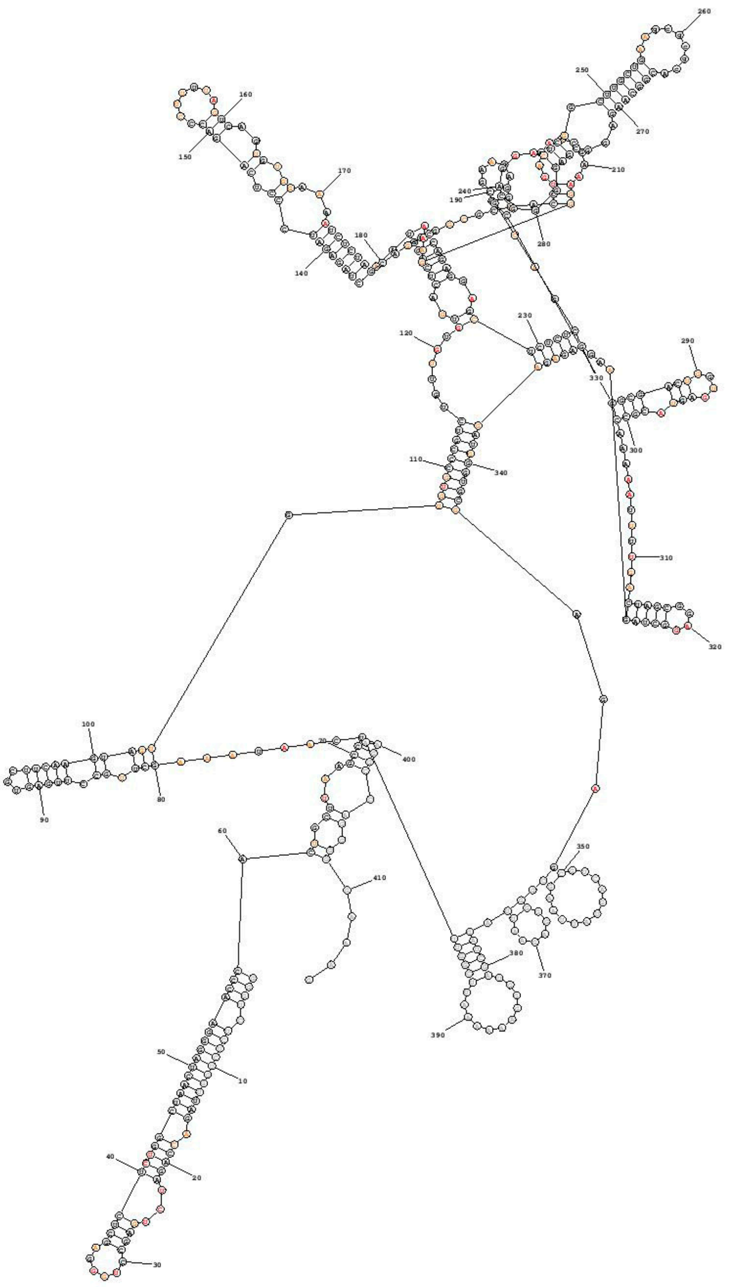

SHAPE >= 0.85

0.85 > SHAPE >= 0.4

0.4 > SHAPE

No Data

ENERGY = −223.5 DimGag414

Supplementary figure 8 - Dimer 414 + NC

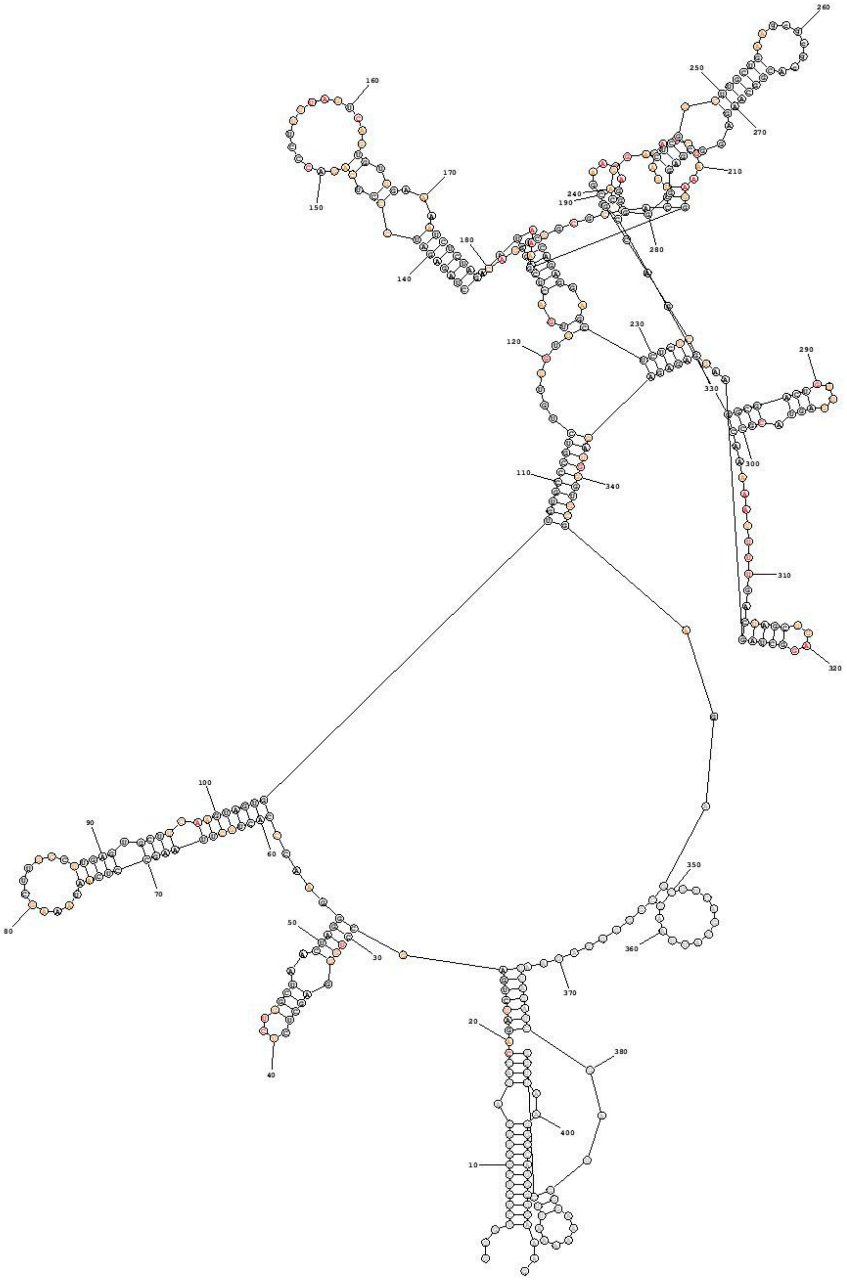

SHAPE >= 0.85

0.85 > SHAPE >= 0.4

0.4 > SHAPE

No Data

ENERGY = -202.6 DimNC414

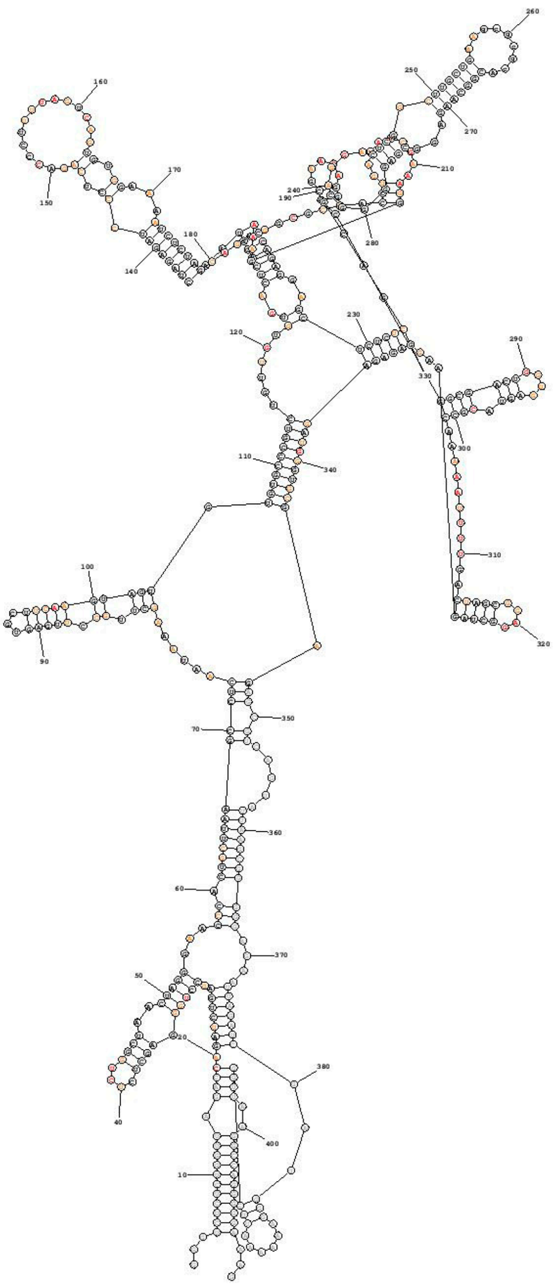

SHAPE >= 0.85

0.85 > SHAPE >= 0.4

0.4 > SHAPE

No Data

ENERGY = -201.1 DimNC414

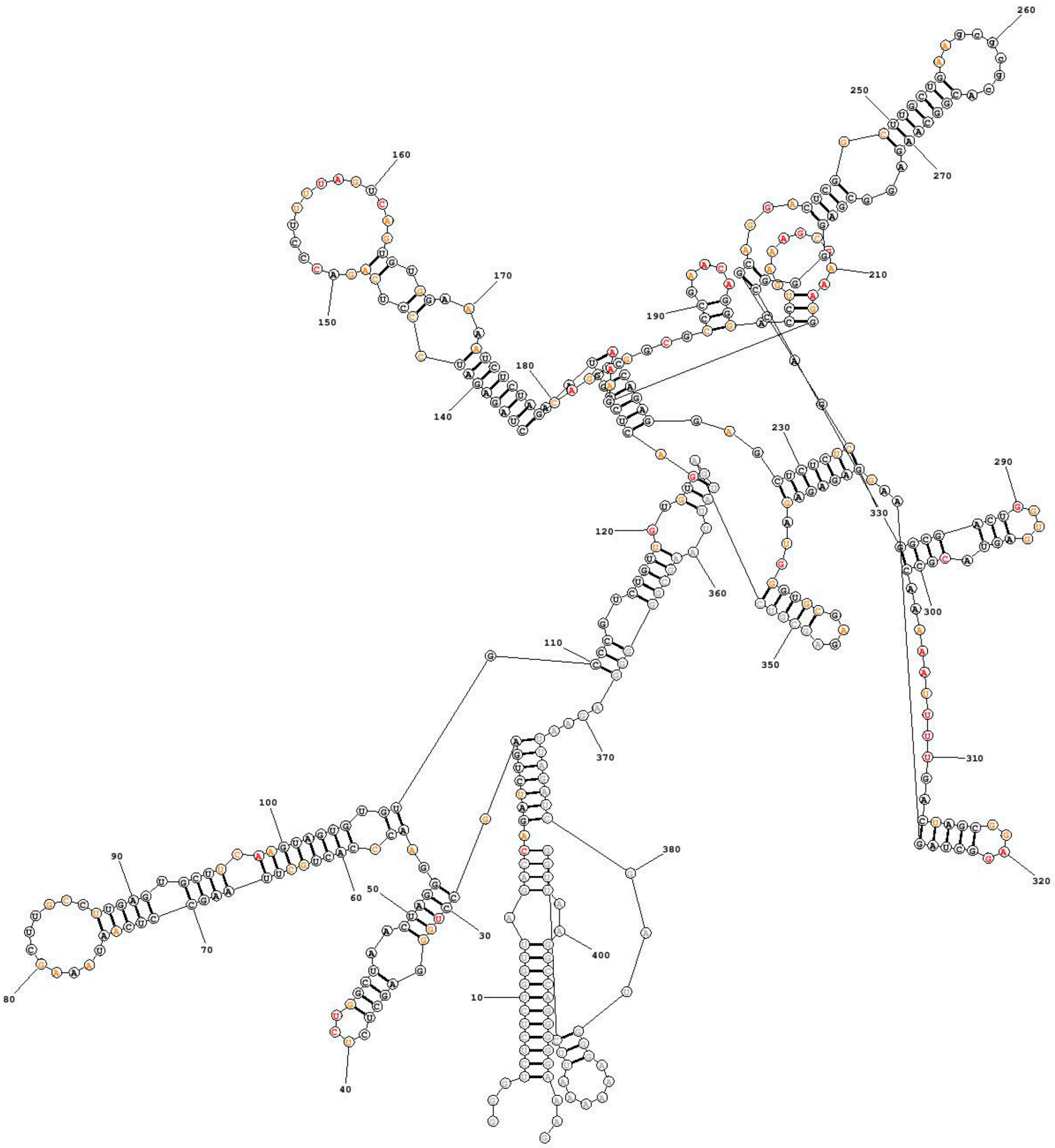

SHAPE >= 0.85

0.85 > SHAPE >= 0.4

0.4 > SHAPE

No Data

ENERGY = −200.6 DimNC414

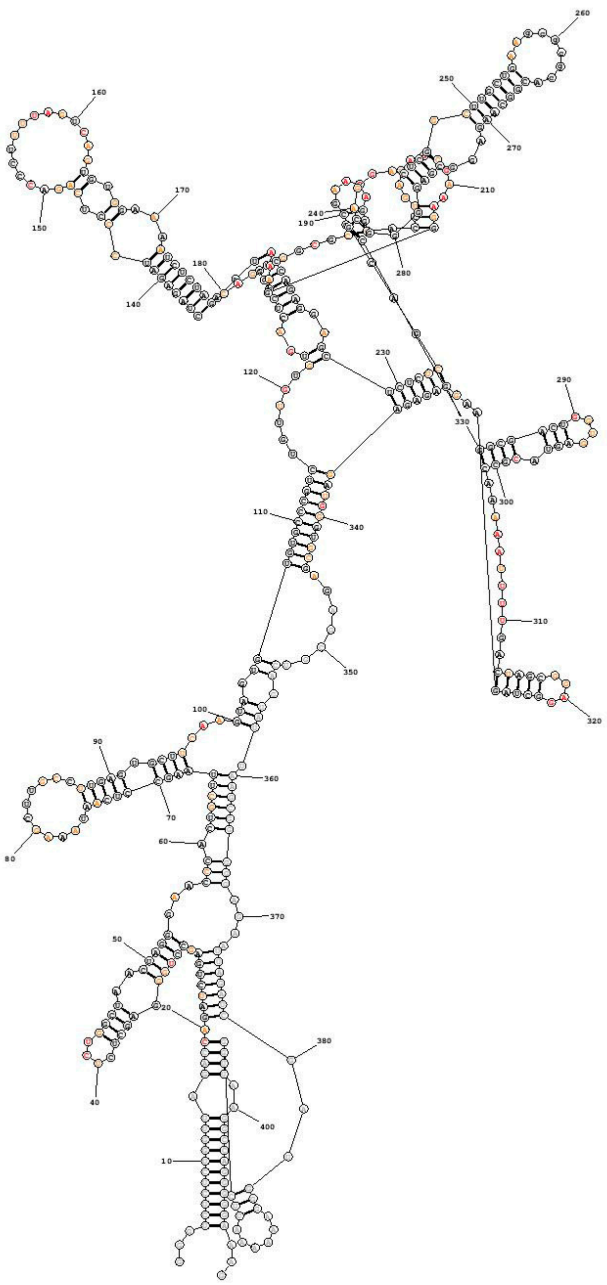

SHAPE >= 0.85

0.85 > SHAPE >= 0.4

0.4 > SHAPE

No Data

ENERGY = -200.5 DimNC414

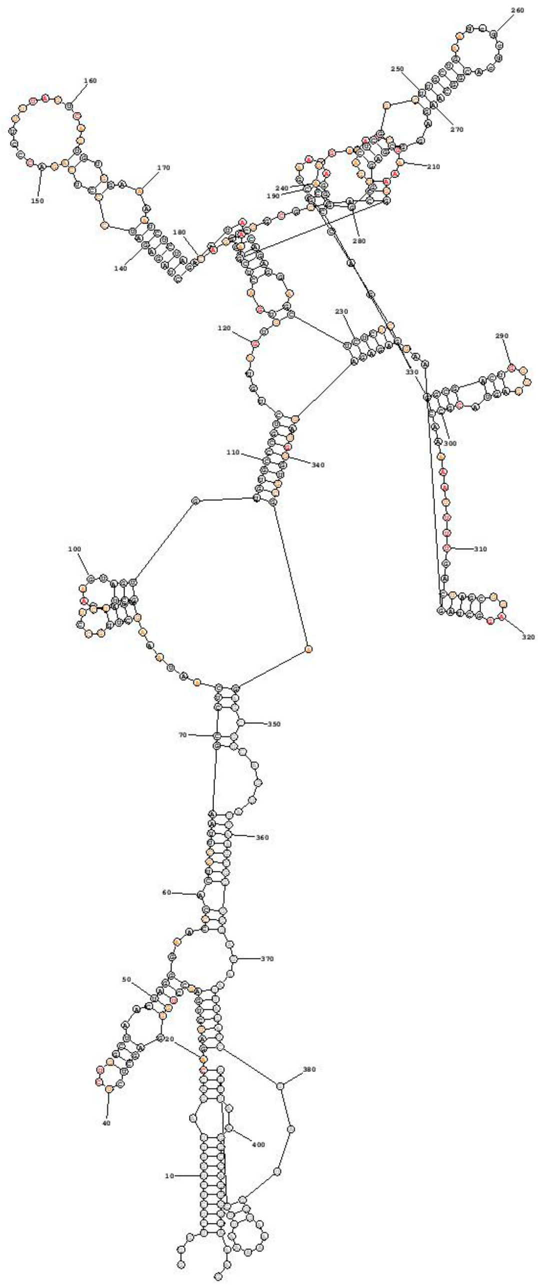

SHAPE >= 0.85

0.85 > SHAPE >= 0.4

0.4 > SHAPE

No Data

ENERGY = −200.1 DimNC414

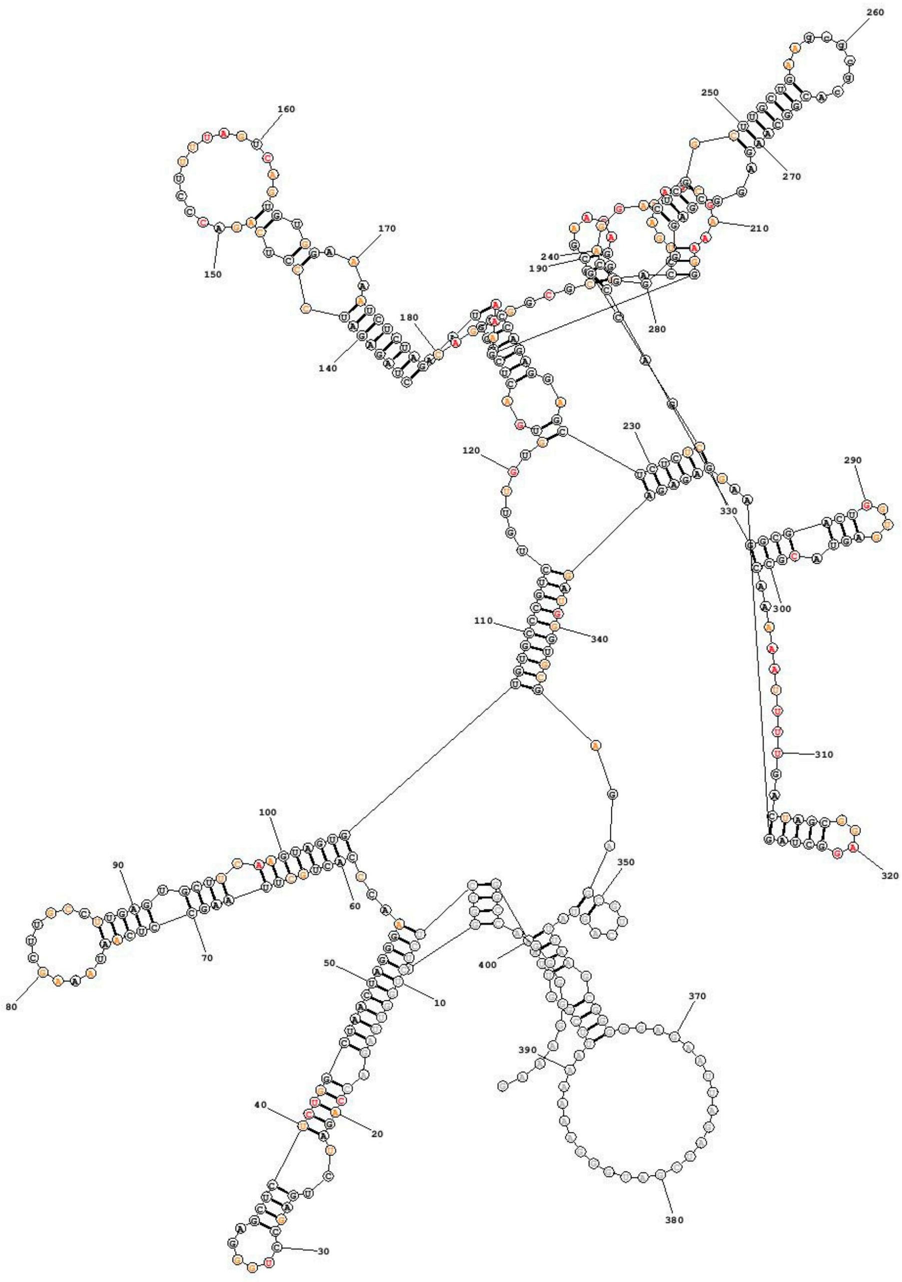

SHAPE >= 0.85

0.85 > SHAPE >= 0.4

0.4 > SHAPE

No Data

ENERGY = -199.5 DimNC414

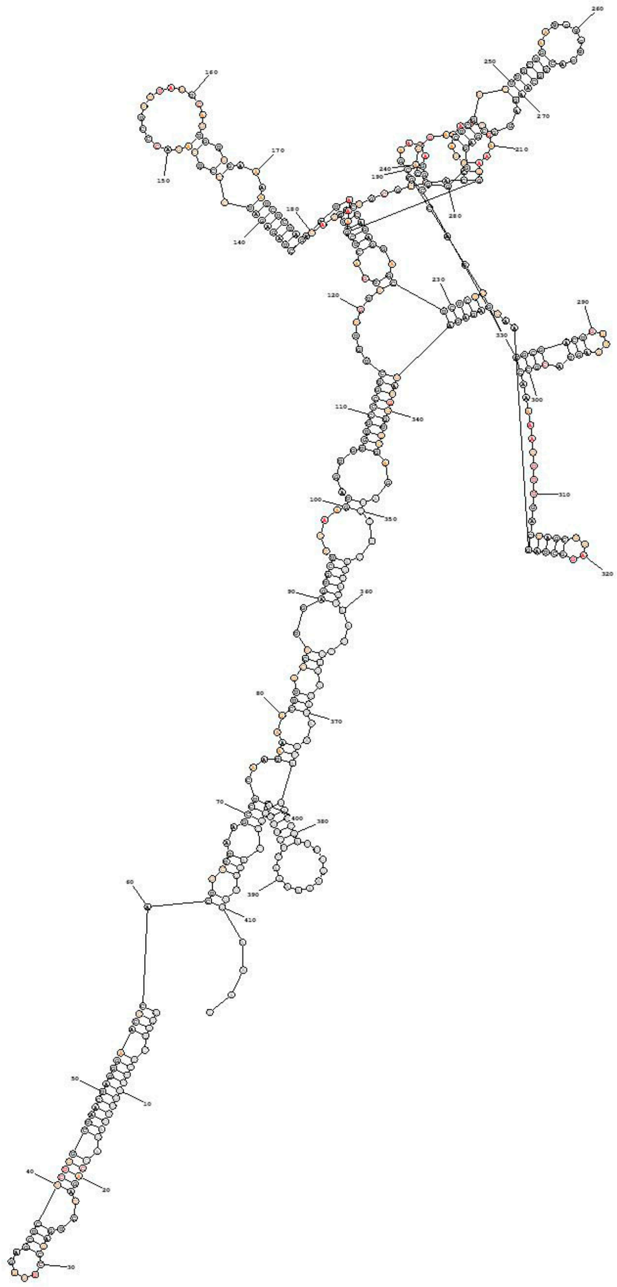

SHAPE >= 0.85

0.85 > SHAPE >= 0.4

0.4 > SHAPE

No Data

ENERGY = -199.3   DimNC414

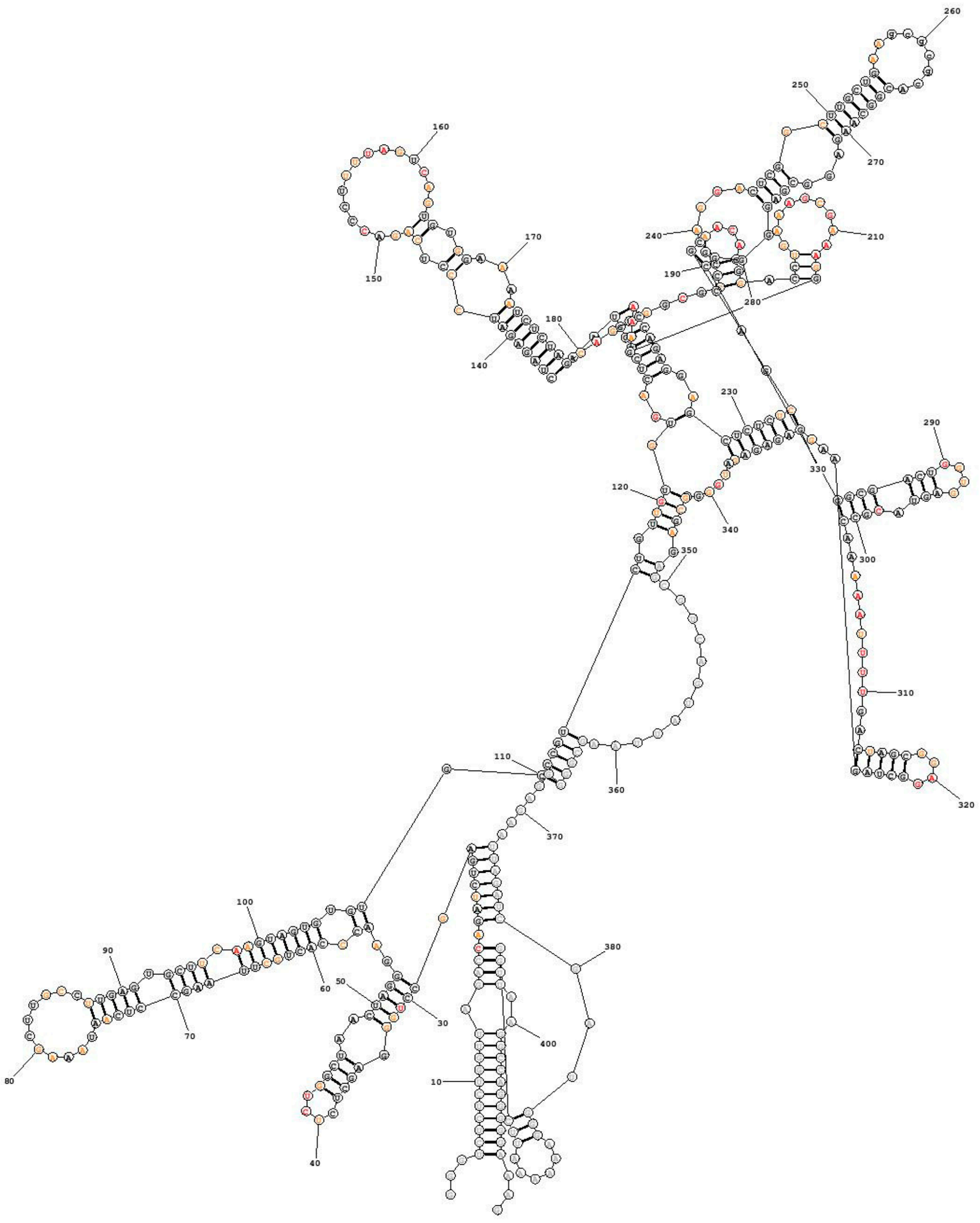

SHAPE >= 0.85
0.85 > SHAPE >= 0.4
0.4 > SHAPE
No Data

ENERGY = -199.2 DimNC414

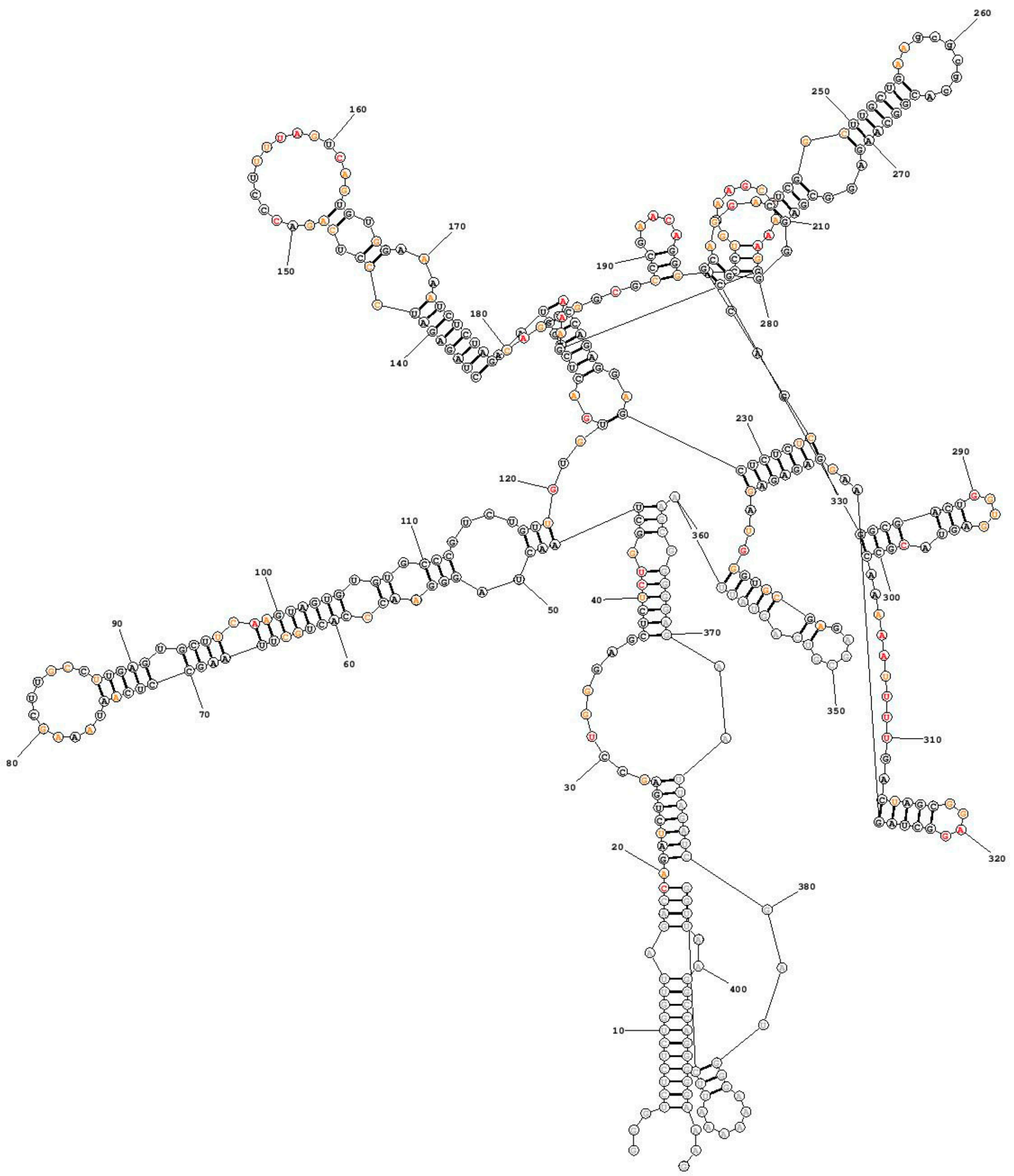

SHAPE >= 0.85
0.85 > SHAPE >= 0.4
0.4 > SHAPE
No Data

ENERGY = -199.1   DimNC414

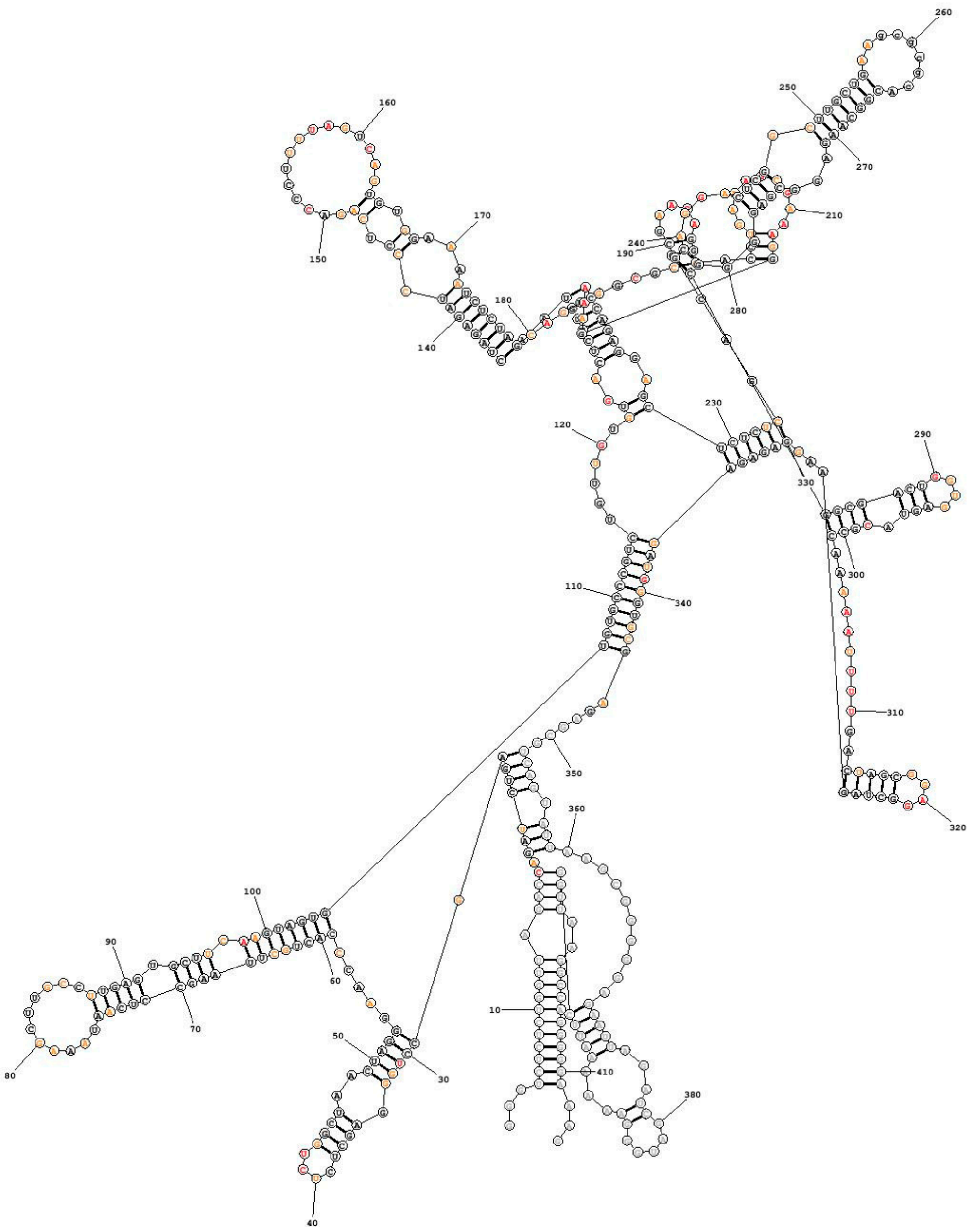

SHAPE >= 0.85
0.85 > SHAPE >= 0.4
0.4 > SHAPE
No Data

ENERGY = −199.1   DimNC414

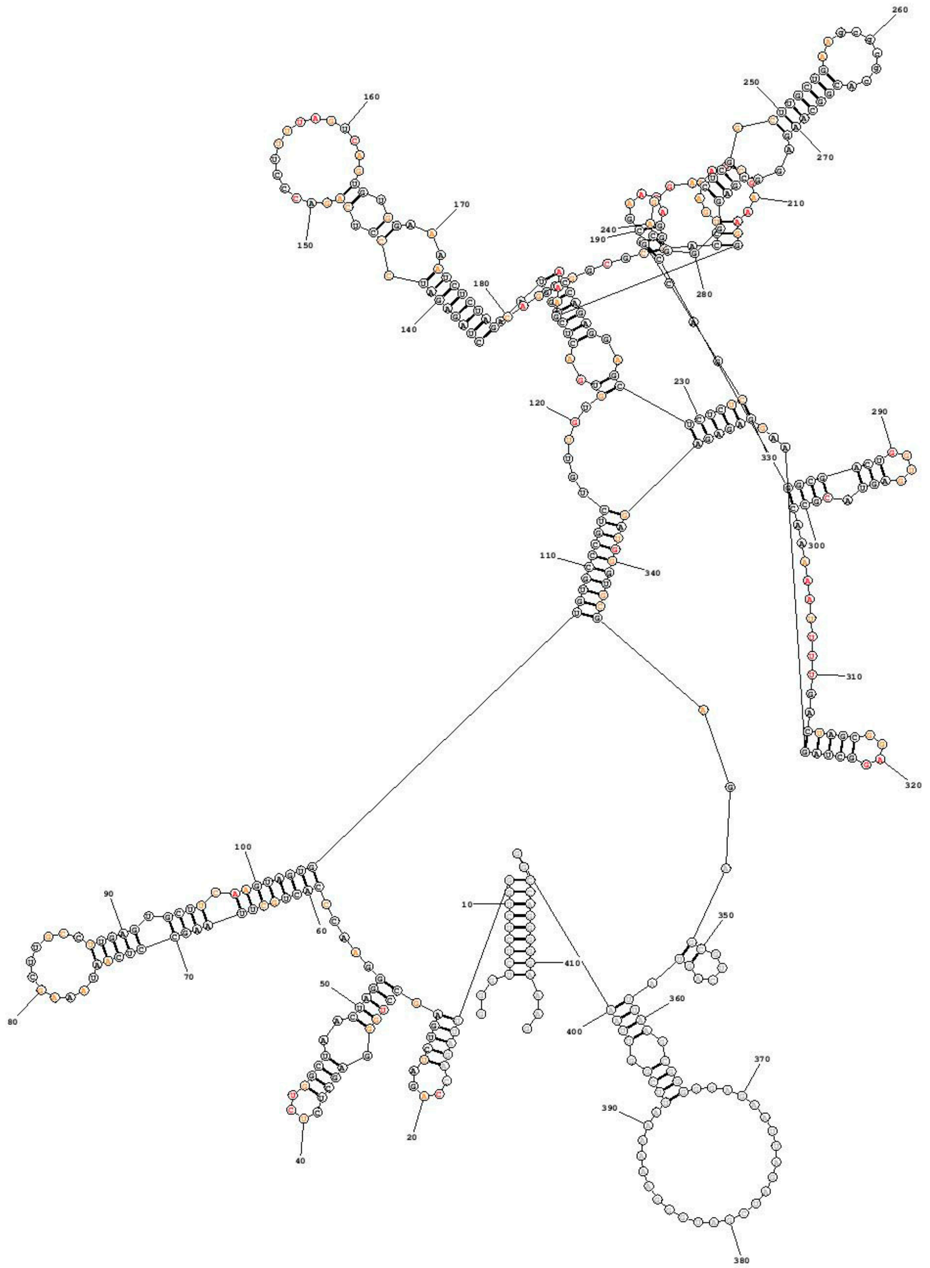

SHAPE >= 0.85

0.85 > SHAPE >= 0.4

0.4 > SHAPE

No Data

ENERGY = −198.9 DimNC414

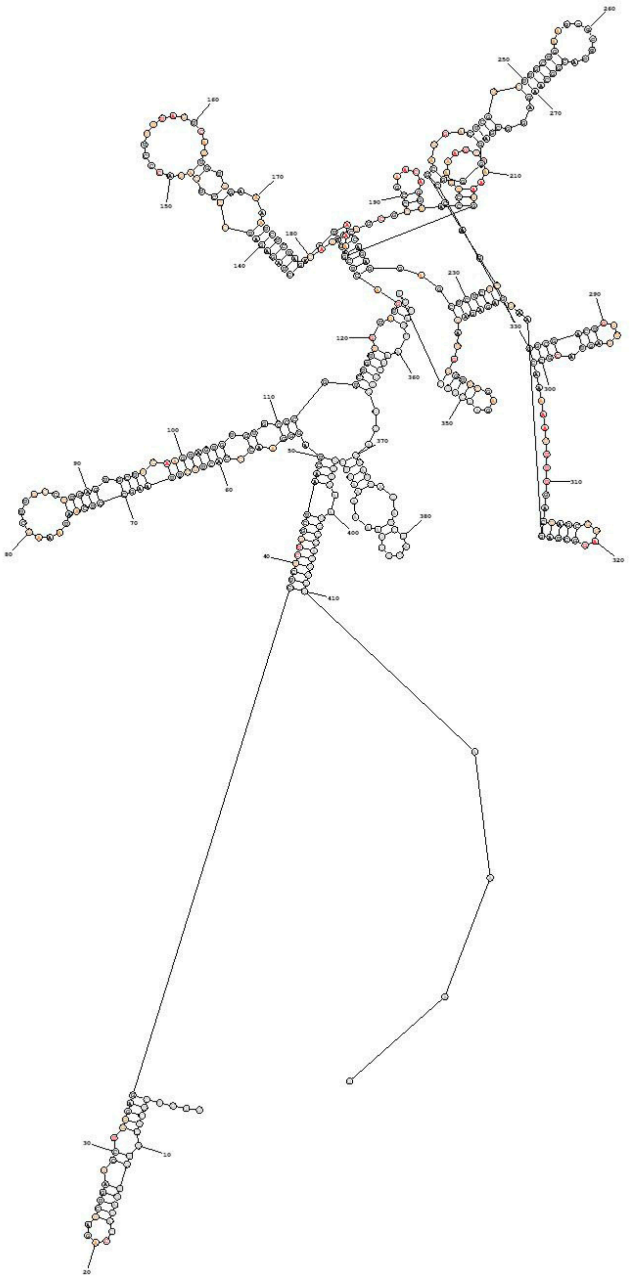

SHAPE >= 0.85
0.85 > SHAPE >= 0.4
0.4 > SHAPE
No Data

ENERGY = -198.8 DimNC414

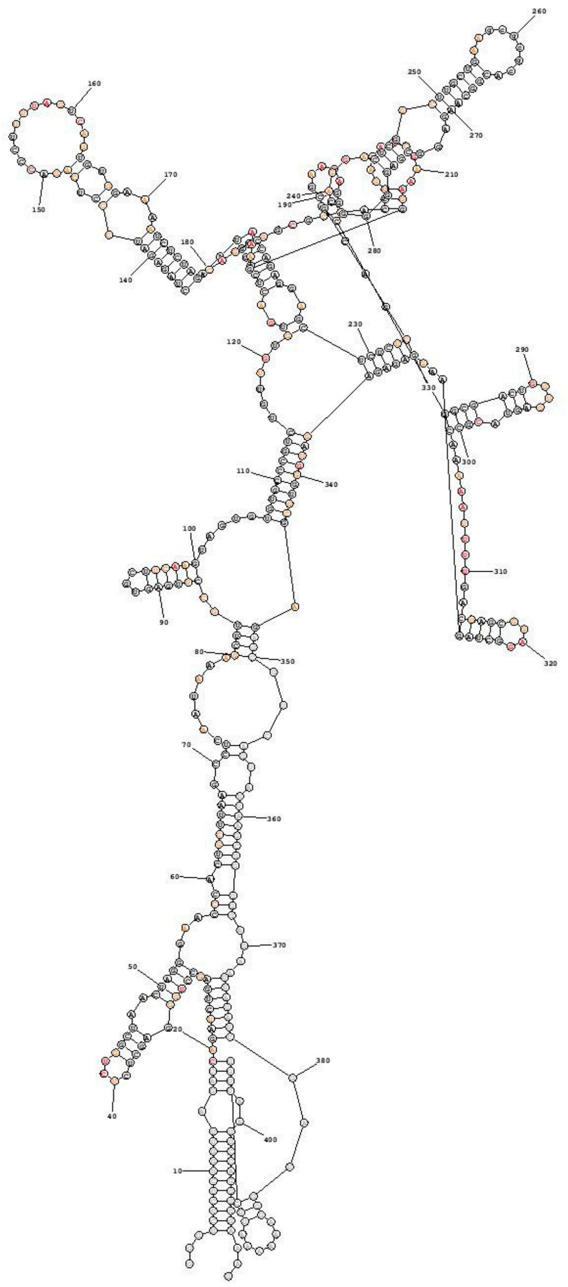

SHAPE >= 0.85
0.85 > SHAPE >= 0.4
0.4 > SHAPE
No Data

ENERGY = −198.4 DimNC414

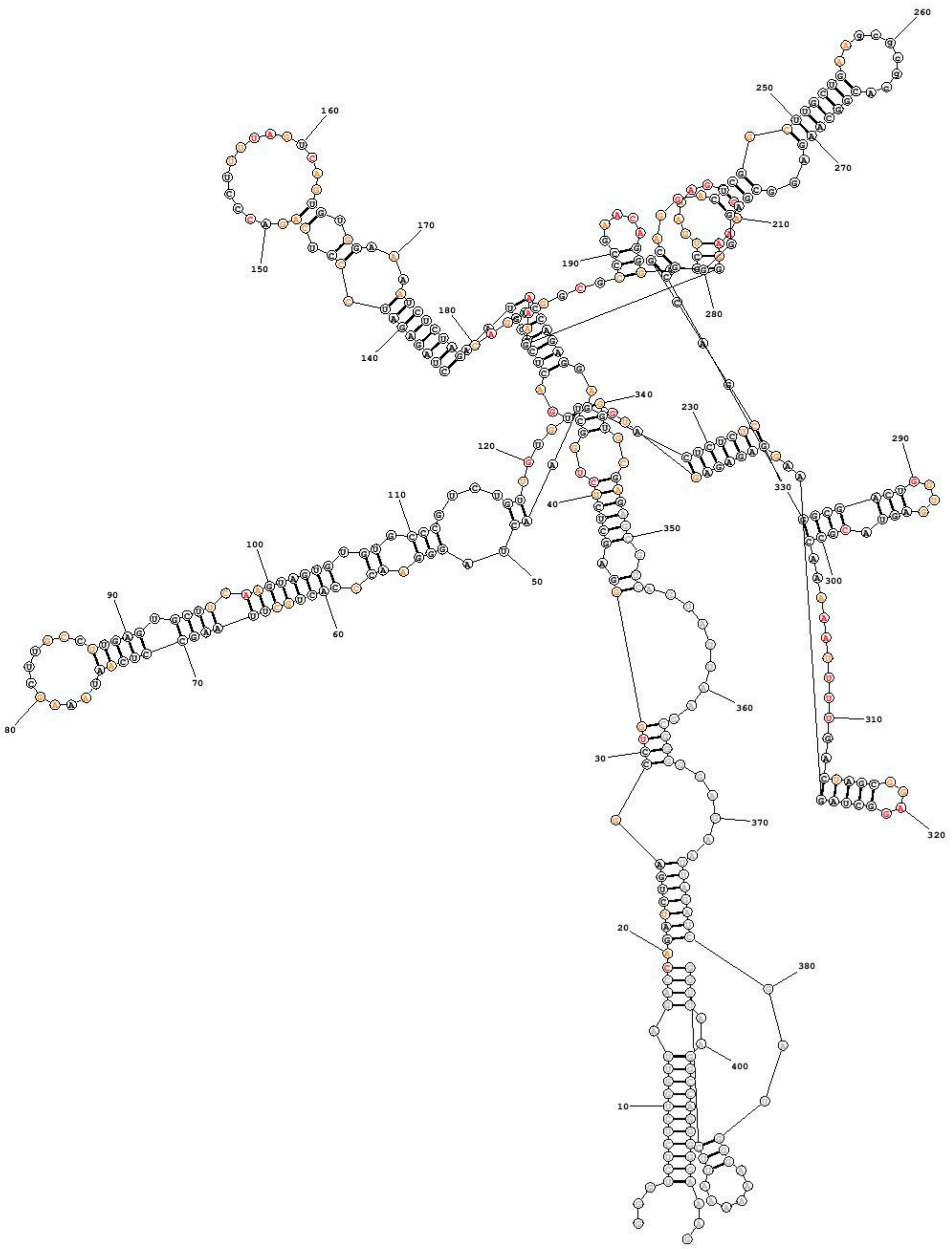

SHAPE >= 0.85
0.85 > SHAPE >= 0.4
0.4 > SHAPE
No Data

ENERGY = −198.3 DimNC414

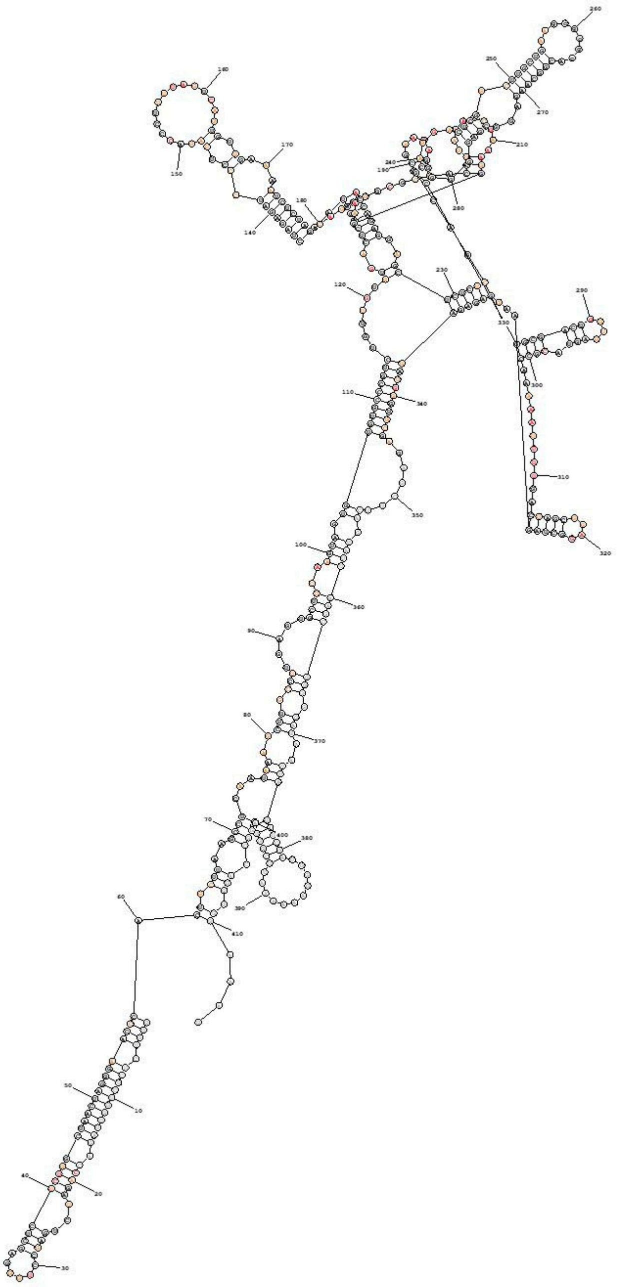

SHAPE >= 0.85

0.85 > SHAPE >= 0.4

0.4 > SHAPE

No Data

ENERGY = -198.1   DimNC414

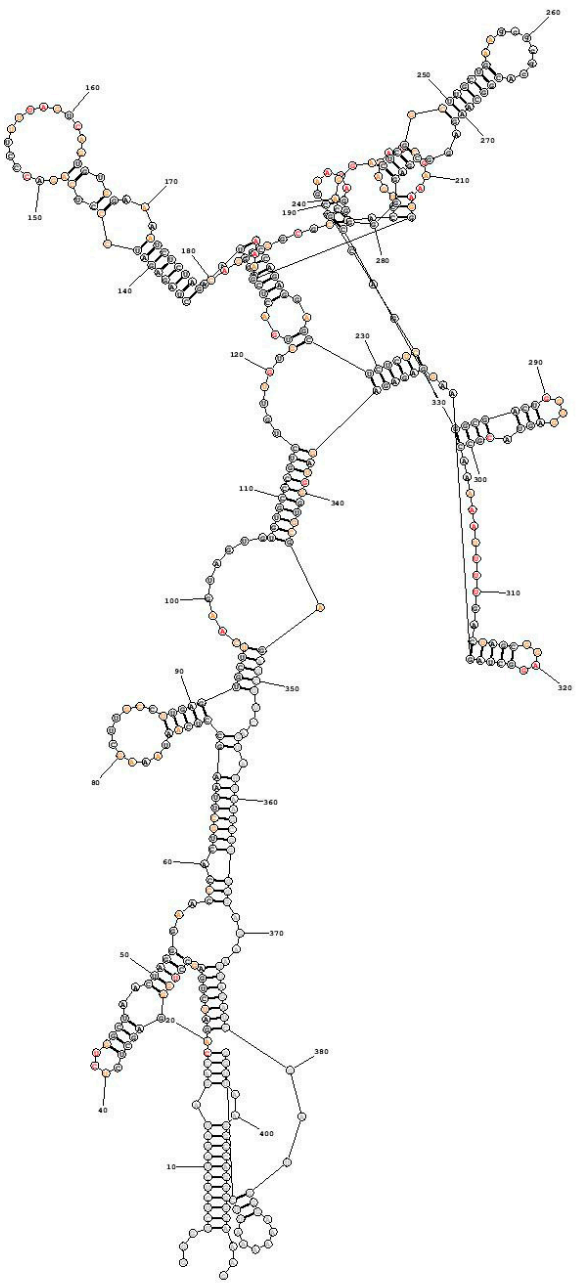

SHAPE >= 0.85
0.85 > SHAPE >= 0.4
0.4 > SHAPE
No Data

ENERGY = −198.0 DimNC414

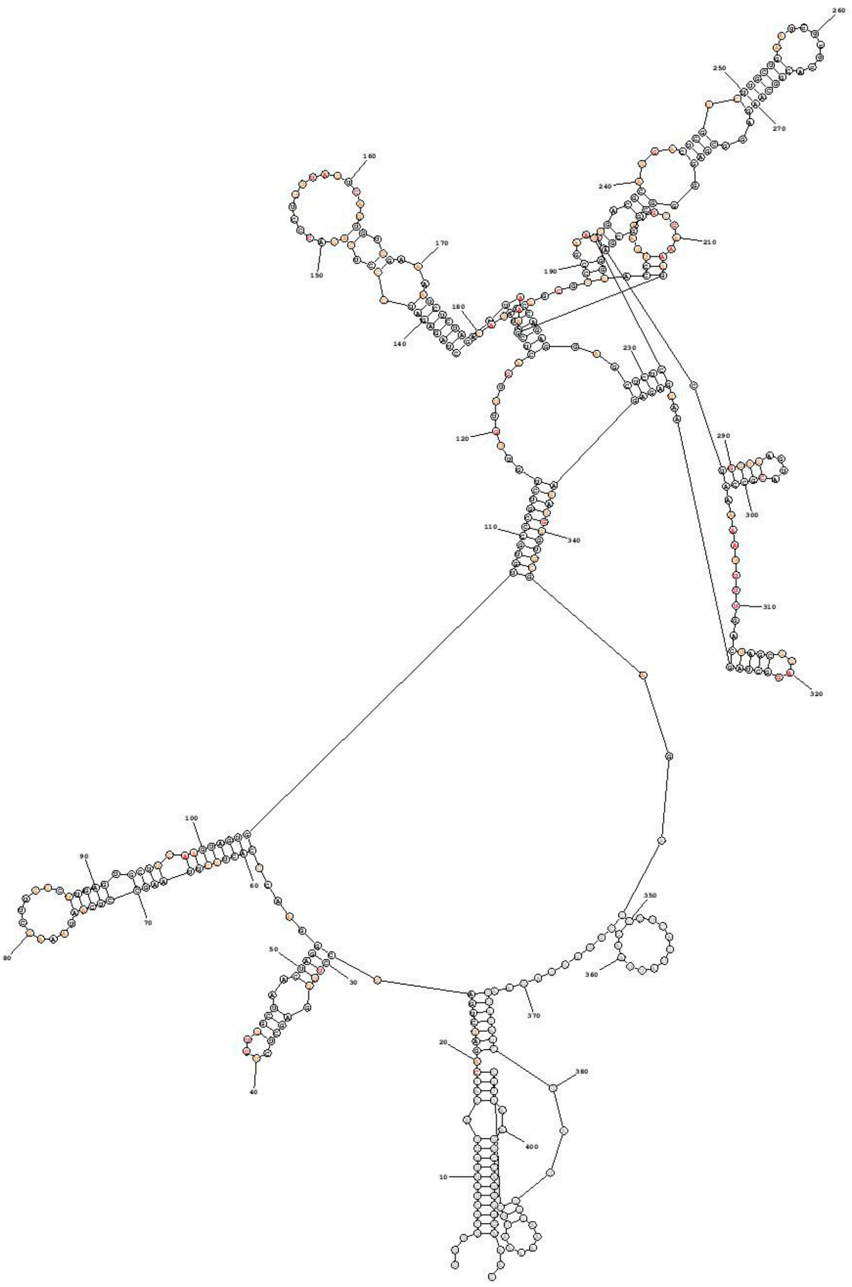

SHAPE >= 0.85
0.85 > SHAPE >= 0.4
0.4 > SHAPE
No Data

ENERGY = -198.0 DimNC414

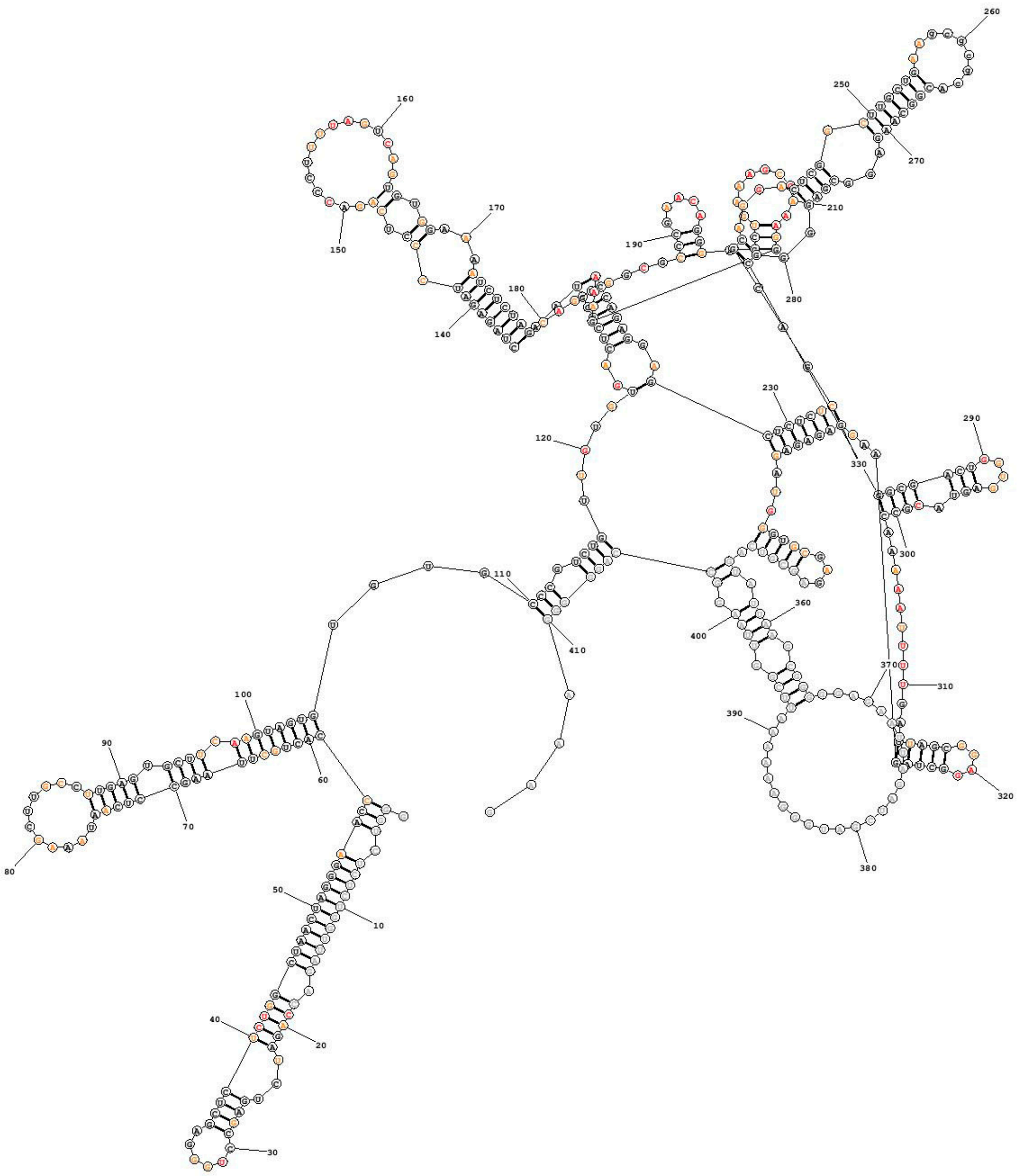

SHAPE >= 0.85

0.85 > SHAPE >= 0.4

0.4 > SHAPE

No Data

ENERGY = -197.6 DimNC414

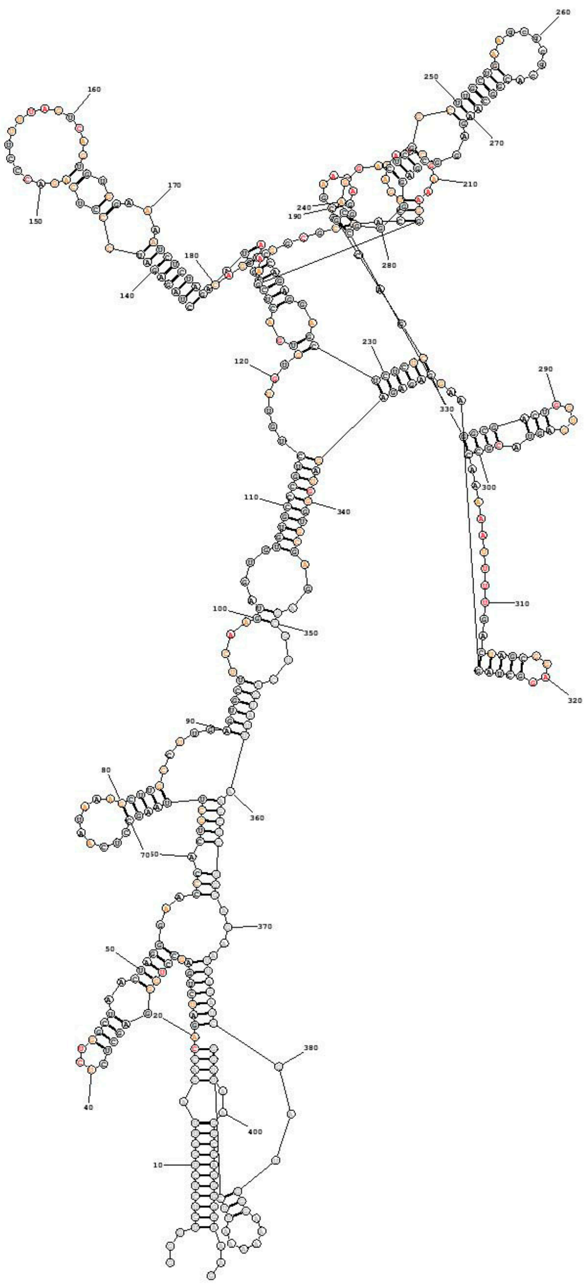

SHAPE >= 0.85

0.85 > SHAPE >= 0.4

0.4 > SHAPE

No Data

ENERGY = -197.3 DimNC414

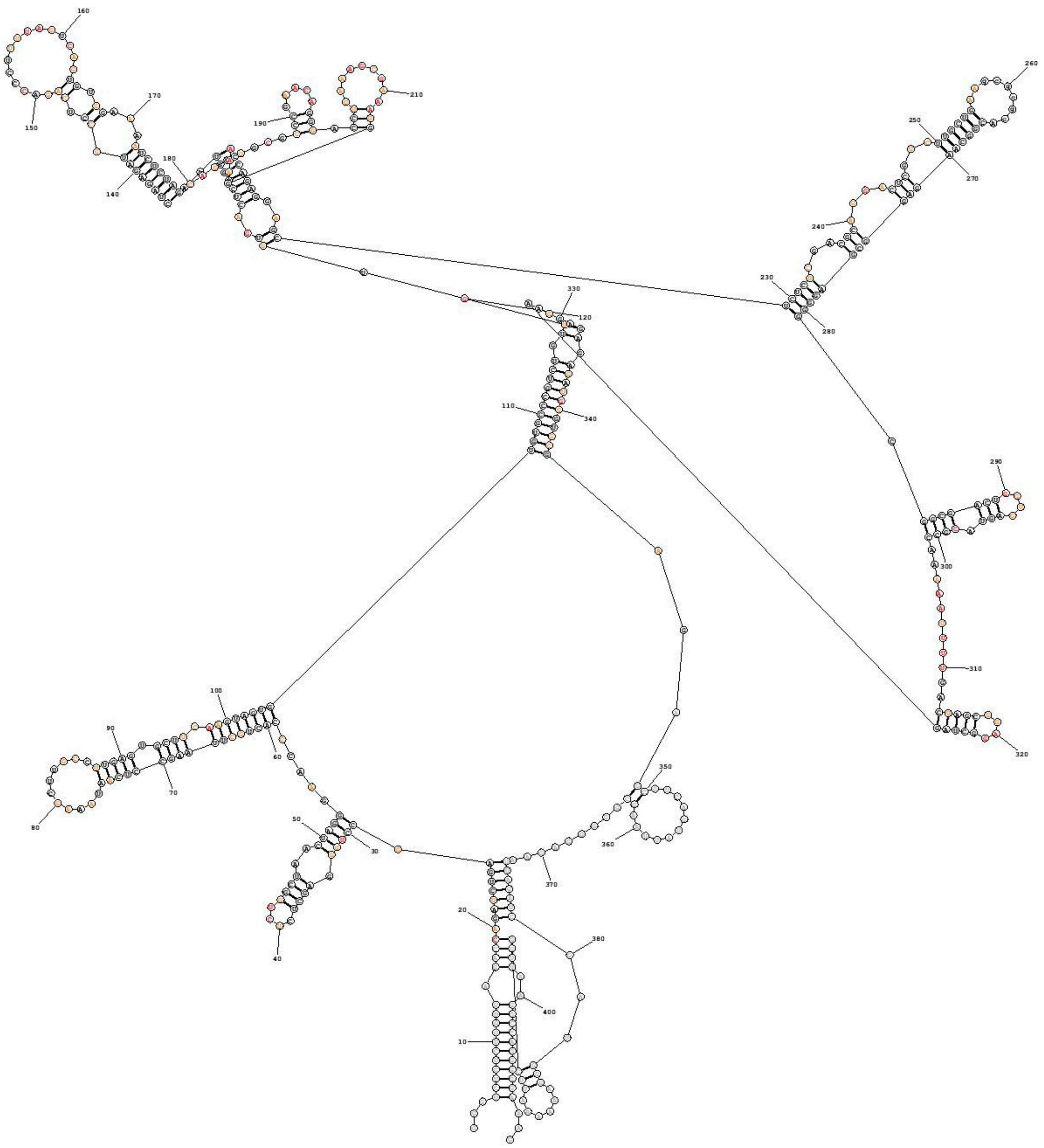

SHAPE >= 0.85
0.85 > SHAPE >= 0.4
0.4 > SHAPE
No Data

ENERGY = -196.9 DimNC414

Supplementary table 1

nt position	Average TAR	STDEV TAR
	0.2665074	0.3661483
2	0.38305327	0.2511443
3	0.37376643	0.2879892
4	0.2504804	0.6653498
5	0.18811451	0.3499239
6	0.15355502	0.2171777
7	-0.0338769	0.4883925
8	-0.2088911	1.2378009
9	-0.1469656	1.1126838
10	-0.0278267	0.5777754
11	0.13689826	0.2775238
12	0.38070582	0.3421622
13	0.08495929	0.0232835
14	0.06061332	0.1643782
15	0.26233257	0.5600154
16	-0.0158949	0.172775
17	-0.2063363	0.6500776
18	0.1808629	0.3344885
19	-0.2263946	0.3442612
20	-0.0626797	0.4389696
21	-0.4299668	1.2885239
22	0.4128341	1.1695634
23	-0.7136459	1.0419538
24	-0.1855131	0.9543451
25	-0.0366177	0.7452045
26	0.11890721	0.3377624
27	0.12308861	0.3969431
28	-0.1274207	0.352617
29	-0.0159404	0.5385892
30	-0.1512345	1.0490851
31	0.36770038	2.3531707
32	-0.1034046	0.0970619
33	0.00214829	1.2602249
34	0.48179247	0.4013812
35	-0.6635995	0.5219972
36	0.28308889	0.2968785
37	-0.1089343	0.2353541
38	0.0025958	0.2585824
39	0.13307275	0.3367265
40	0.01881112	0.3190593
41	0.108156	0.6549583
42	0.2831002	0.4224892
43	0.03002873	0.1609581
44	0.09136774	0.1525556
45	-0.203977	0.5451496
46	-0.2107448	0.2019164
47	-0.0646061	0.9134424
48	0.02129642	0.430392
49	0.10139217	0.3500163

50	0.48766645	0.6132808
51	-0.0840874	0.3246259
52	-0.3815018	1.070679
53	-0.4316778	1.1940773
54	-0.042255	0.8208602
55	-0.0554143	0.0867007
56	-0.2006818	0.3404946
57	0.07836271	0.1327088

	Average	ST DEV
nt position	TAR TAT	TAR TAT
2	-0.341501	2.1556735
3	-0.5118582	1.219165
4	-0.2192913	0.4895126
5	-0.0675181	0.1142591
6	0.24506582	0.7635512
7	0.09344767	0.1082505
8	0.94887086	2.6703417
9	1.12653602	1.27425
10	0.06670213	0.1880391
11	-0.5655252	0.1460915
12	-0.4541309	0.8400084
13	-0.0103076	0.779207
14	0.2428654	0.8540368
15	0.54708811	1.2554516
16	-0.0242978	0.4006823
17	-0.3880923	0.3311671
18	0.07314079	0.2559625
19	0.27420453	0.7363491
20	-0.043345	0.4537053
21	-4.0033462	4.7052252
22	-0.499438	0.5365924
23	-0.9475099	5.1661684
24	-0.0441086	0.1652223
25	0.12765982	1.2453831
26	-0.0751959	0.8261414
27	-0.0377445	0.4335415
28	0.0100594	0.019803
29	0.51024266	0.2694002
30	-0.9350698	3.9337069
31	0.18551024	0.9343271
32	0.57550186	1.8077876
33	-0.4697354	1.8306022
34	-0.3828041	0.2689553
35	-0.1340727	0.3412974
36	0.13484706	0.1001802
37	1.41941712	1.6451059
38	0.30172285	0.4353783
39	-0.2018235	0.6883578
40	-0.0137624	0.3623781
41	-0.1027532	0.1148393
42	0.49513475	1.3061924
43	-0.04601	0.0709409

44	-0.0176365	0.0736213
45	0.31662921	0.6478654
46	-0.141756	0.4270561
47	0.17555167	0.0805944
48	0.35859218	0.6410258
49	-0.3041184	0.3558609
50	-1.6802142	2.1525295
51	0.05679038	0.225161
52	-0.2491283	0.5465371
53	0.32713411	0.3737162
54	0.74236281	0.8143067
55	0.95613735	0.9508794
56	-0.1974891	0.9068471
57	0.99510564	0.8949951

nt position	MONOMER	STDEV
10	0.54826589	0.5011783
11	0.22999821	0.1150694
12	0.04501669	0.0134831
13	0.06692524	0.0044756
14	-0.1363213	0.293368
15	0.03619317	0.1307935
16	0.05743481	0.108898
17	0.39301592	0.7223968
18	-0.0253368	0.113822
19	0.09052461	0.0632818
20	0.11747922	0.1316549
21	0.42004876	0.6807267
22	1.28856133	0.8209222
23	1.05740794	0.6355558
24	1.05120707	0.4895681
25	-1.7268263	5.3573665
26	2.45213454	1.3291514
27	1.78588863	1.1905499
28	1.45396298	1.1181865
29	0.51695837	0.0508863
30	0.30105237	0.3050986
31	0.87759779	0.8655125
32	1.4775496	0.8865496
33	0.23578103	0.0837871
34	0.46970915	0.2829541
35	0.64954816	0.2865549
36	-0.0132903	0.2382527
37	0.05348863	0.0471134
38	0.2632594	0.2456691
39	0.0166689	0.0891778
40	0.21686437	0.1067808
41	0.37470873	3.1776421
42	0.25706062	0.3898065
43	0.4339602	0.0461771
44	0.25941905	0.1504205
45	0.03957939	0.072055
46	0.20300177	0.0328228

47	0.27798333	0.2401543
48	0.18665256	0.189817
49	0.01589314	0.0641626
50	0.26615398	0.2723762
51	0.05302523	0.0164752
52	0.48724774	0.1505422
53	0.04040885	0.139471
54	0.09186612	0.083931
55	0.08034653	0.0669057
56	0.60539527	1.2596453
57	0.14308373	0.2760068
58	0.3063702	0.366633
59	0.26275904	0.2319578
60	0.06651523	0.0888817
61	0.43295705	0.0613845
62	0.47153655	0.5902402
63	0.78190897	0.0362245
64	0.34149032	0.1277212
65	0.67820509	1.0273592
66	-0.5080013	2.4101487
67	0.65440203	0.1703084
68	0.62233591	0.5208027
69	0.39953021	0.1896946
70	0.07625983	0.1263076
71	0.09161125	0.1143863
72	0.08853562	0.2266542
73	-0.1105091	0.6297393
74	0.37881257	0.206766
75	0.99729735	1.8230682
76	0.8008791	0.5162529
77	0.60492945	0.080037
78	0.92832196	0.3602211
79	0.66367306	0.301474
80	0.08237423	0.031101
81	0.07109948	0.1249385
82	0.05657277	0.1187196
83	0.58145881	0.4421954
84	0.30469119	0.103359
85	0.13877875	0.148934
86	0.074133	0.0914709
87	0.01315707	0.0326122
88	0.36913808	0.1979426
89	0.32394097	0.1309464
90	0.51299325	0.014456
91	0.3671735	0.0248071
92	0.14700999	0.0584652
93	0.19967968	0.1567535
94	0.15789849	0.2796108
95	0.32814226	0.3282085
96	0.35660827	0.2799144
97	0.05694559	0.2665564
98	0.17455054	0.0244247

99	0.03275498	0.0924218
100	0.29714948	0.2065653
101	0.1738822	0.1340958
102	0.14215301	0.0269334
103	0.3870908	0.2445045
104	0.30604224	0.2801405
105	0.20204989	0.1257324
106	0.28786202	0.001554
107	0.62460215	0.2171557
108	0.52307626	0.0347703
109	0.27035006	0.2084205
110	0.20857024	0.1076403
111	0.03433936	0.032333
112	0.0624108	0.0726288
113	0.18747066	0.1945779
114	0.27390321	0.1137915
115	0.17530841	0.260255
116	0.32155198	0.29652
117	0.19585662	0.1854483
118	0.26603981	0.1691682
119	0.26291966	0.0469192
120	-0.0155606	0.1612755
121	0.39493979	0.4152205
122	0.05022985	0.1599218
123	0.29399591	0.1523864
124	0.11095413	0.1497256
125	0.22614947	0.1817004
126	0.13430856	0.0773147
127	0.05597059	0.0957042
128	0.56672586	0.3569942
129	0.2239609	0.1025392
130	0.25319245	0.2669218
131	0.13387647	0.0198731
132	0.20537398	0.5022621
133	0.40101366	0.1415459
134	0.0607441	0.026569
135	0.02773147	0.018325
136	0.16877708	0.1714508
137	0.17978914	0.1759918
138	0.33921783	0.3890891
139	0.09323853	0.1563336
140	0.16424722	0.1763269
141	0.08540262	0.0558582
142	0.24790444	0.0604326
143	0.34008493	0.0272232
144	0.31978002	0.1172409
145	0.26568862	0.1046575
146	0.93420576	0.643745
147	0.50083558	0.3599219
148	0.25910454	0.2567879
149	-0.0670053	0.2454268
150	2.85884955	4.5951025

151	1.40191991	0.8548621
152	0.50557173	0.2813086
153	1.08203856	0.7656592
154	0.31760137	0.1823009
155	0.295918	0.2663465
156	0.9054047	0.0931184
157	0.48051284	1.0047978
158	0.3631043	0.3621065
159	0.23073721	0.232144
160	0.37391296	0.4088896
161	0.11826031	0.8872201
162	0.3370754	0.2418902
163	1.05115892	0.1928964
164	0.10317098	0.0956743
165	0.36736583	0.1614212
166	0.48536846	0.0705361
167	0.48374035	0.4842526
168	0.11277342	0.040715
169	0.5003487	0.1717437
170	0.06304816	0.0885089
171	1.17550929	0.487117
172	0.3327098	0.1053531
173	0.36925458	0.1763331
174	0.05336347	0.0165113
175	0.13498061	0.1217963
176	0.23105415	0.2758425
177	0.46726743	0.3691553
178	0.39647421	0.0536501
179	1.3447201	2.2597441
180	-0.0901981	14.315603
181	0.90420628	1.3679504
182	0.79800994	0.3527245
183	0.55455447	0.4513726
184	0.67007747	0.3688484
185	1.02095799	0.5015261
186	0.12585207	0.0360463
187	0.20315819	0.0614172
188	0.49425665	0.1464134
189	0.84506279	0.97311
190	0.1750528	0.3468072
191	0.14129645	0.2106038
192	1.50449269	1.1504748
193	1.70694242	3.7330452
194	-0.0712907	0.1913308
195	0.6505176	0.2379754
196	-0.0072849	0.0125293
197	0.4195145	0.8661255
198	0.34909276	0.2947964
199	0.44226789	0.1933492
200	0.37718909	0.0715639
201	1.13704943	0.0120502
202	0.92434294	0.051101

203	0.87839442	0.3492078
204	0.21523638	0.217817
205	0.49344952	0.2463919
206	0.72394668	0.2149236
207	0.6035173	0.1851386
208	1.47162315	2.5925841
209	0.43153344	0.0391801
210	0.62548787	0.0578869
211	0.59347093	0.1879589
212	0.82047397	0.1453393
213	0.91771111	0.2566116
214	0.63793765	0.1287355
215	0.73806297	0.2585649
216	0.75884189	0.3990471
217	0.74522572	0.3085525
218	0.28582268	0.1670903
219	0.2593542	0.1552926
220	-0.2052838	1.2446059
221	0.30418871	0.1716671
222	0.17147564	0.161882
223	0.29944366	0.2085577
224	0.16977109	0.0400276
225	0.34118192	0.32844
226	0.07186717	0.1025268
227	0.44927412	0.4006707
228	0.0691673	0.0499908
229	0.24743511	0.09942
230	0.18763588	0.1230011
231	0.2260358	0.0912905
232	0.57973702	0.2194489
233	0.23979092	0.0616192
234	0.21500716	0.2381011
235	0.23100508	0.1443601
236	0.17201627	0.2869713
237	0.29954869	0.1384224
238	0.16466403	0.5230471
239	1.36585453	5.2049685
240	0.40009489	0.1344376
241	0.62157961	0.2105437
242	0.19495675	0.0236857
243	0.31352857	0.0340166
244	0.31151616	0.0506037
245	0.33173434	0.1781438
246	0.61654738	0.1065726
247	0.27011908	0.0400851
248	0.15207303	0.1182799
249	0.13900206	0.0981113
250	0.52898704	0.1246084
251	0.52573077	0.1170331
252	0.29683953	0.1797724
253	0.34989333	0.297214
254	0.1697465	0.0934694

255	0.33420967	0.3079027
256	0.20159763	0.1920954
257	0.18002001	0.0413556
258	0.19838145	0.1909577
259	0.50880253	0.3200478
260	0.1039351	0.0946091
261	0.14385354	0.1637085
262	0.17448796	0.0794698
263	0.33982739	0.2783962
264	0.13128837	0.1757539
265	0.31824995	0.193772
266	-0.1879399	0.7052946
267	0.17991014	0.332842
268	0.07805374	0.0911995
269	0.38346642	0.096594
270	0.22305126	0.0626168
271	0.44999903	0.3673027
272	0.71891055	0.349506
273	0.84237852	1.8063208
274	-1.0008528	1.1090477
275	0.43785497	0.392998
276	0.45347423	0.09394
277	0.12261929	0.224153
278	0.23887502	0.150634
279	0.29240354	0.1577285
280	0.29343312	0.1047005
281	0.17941668	0.1774471
282	0.65703166	0.3271571
283	0.85630129	1.7232873
284	-0.6530924	1.0009614
285	0.33141217	0.3596809
286	0.27928378	0.1086454
287	0.25355798	0.1406413
288	0.36371345	0.3339927
289	0.71441252	0.0197207
290	0.56699836	0.2334539
291	0.83895121	0.2926155
292	1.21289194	0.3630051
293	0.17550099	0.1777119
294	0.44587798	0.1772738
295	0.79570796	0.2248014
296	0.5904766	0.1372739
297	0.17767465	0.0686464
298	0.3377365	0.3516191
299	0.17303681	0.5679159
300	0.20444079	0.0609371
301	0.18977482	0.0245699
302	0.1477114	0.1475816
303	-0.1935878	0.6505581
304	0.08181909	0.6313528
305	0.5199394	0.1170551
306	0.02388461	0.275009

307	0.25486919	0.0322611
308	0.10423755	0.1487212
309	0.7707315	0.9160841
310	0.40402621	0.3699181
311	0.53824903	0.0684716
312	0.0379963	0.005008
313	0.31780784	0.038056
314	0.4231403	0.1006969
315	1.96637835	1.7658899
316	0.63834124	0.5347843
317	0.36586724	0.041833
318	0.6809241	0.9441483
319	0.71236325	0.5996414
320	1.01510874	0.0499966
321	0.01856762	0.8410769
322	0.01392494	0.623755
323	0.19026996	0.3731202
324	0.30695984	0.0106918
325	0.08765284	0.2817752
326	0.32565008	0.0120569
327	0.25315504	0.1730451
328	0.34986743	0.2194741
329	0.4783397	0.3750408
330	0.39625523	0.0658654
331	0.09911018	0.214898
332	0.23388083	0.0581032
333	0.45164435	0.3741268
334	0.3059201	0.2562477
335	0.97944727	0.3891737
336	0.06915285	0.1015448
337	0.44567911	0.2522948
338	0.73344748	0.1830358
339	0.04420844	0.2360292
340	0.59656247	0.25383
341	0.92846317	0.5030395
342	0.27227748	0.2639342
343	0.02150945	0.187453
344	1.3084128	1.7095348
345	0.80263704	0.4151739
346	0.7518011	1.6103652
347	3.50735297	4.1345847

	MONOMER	STDEV
nt position	+ NC	
10	1.3162801	0.4718447
11	0.54706335	0.4826417
12	-0.1532149	0.2368116
13	0.00992874	0.0204278
14	-0.0035747	0.1119154
15	0.01840463	0.0364809
16	-0.0501985	0.070751
17	0.08838812	0.0545879
18	0.14826392	0.1812694

19	0.79253599	0.8061514
20	0.09487406	0.0695177
21	0.71382078	0.4644174
22	-0.049772	0.1424311
23	-0.1723365	0.1155967
24	-0.8856828	0.5714153
25	-1.0514078	0.7548351
26	-0.8988707	0.901545
27	-0.7992282	0.9358652
28	-0.2680726	0.2764917
29	-0.0291902	0.0572704
30	0.01247531	0.0604737
31	0.15261319	0.0955025
32	-0.0421379	0.416059
33	-0.3834279	0.1491047
34	-0.5686116	0.2752803
35	-0.5459419	0.3363064
36	-0.4518867	0.4597115
37	-0.0524245	0.0988286
38	0.01566538	0.0402473
39	0.12868114	0.3749456
40	-0.3750854	0.1980339
41	-0.2828434	0.1636946
42	0.01212381	0.2473663
43	-0.0158477	0.0535559
44	-0.0097196	0.0315819
45	0.03052985	0.0225551
46	0.10124446	0.0859798
47	0.06714919	0.0068983
48	0.01019268	0.010633
49	-0.0237303	0.0102208
50	-0.2182859	0.1508145
51	-0.1175077	0.2728859
52	0.28548917	0.1813709
53	0.82009906	0.5896397
54	4.10396461	1.0031056
55	2.44593275	0.2862301
56	18.0619901	1.7432322
57	1.95721359	0.6948864
58	7.20035637	1.2823189
59	9.21403943	1.4846952
60	0.48811183	0.6075651
61	-0.082337	0.2984461
62	1.18064571	0.3503092
63	-0.0475598	0.4486089
64	-0.2572909	0.4603284
65	-0.6128149	0.3503579
66	-1.2526316	0.2900136
67	-0.3330801	0.0861284
68	0.36384655	0.1632587
69	-0.0053592	0.1112602
70	0.07431174	0.036389

71	0.01127333	0.0754425
72	0.15520256	0.2890993
73	-0.4704586	0.170825
74	0.5837941	0.2407617
75	0.24253981	0.0017722
76	-0.1545212	0.1000355
77	0.16031025	0.1063546
78	0.27898114	0.0416686
79	0.16550569	0.128677
80	0.00067448	0.1663538
81	-0.0391439	0.0049295
82	0.53797262	0.3569555
83	6.09049179	1.1207814
84	0.68144923	0.177113
85	0.07404941	0.1158619
86	0.10214632	0.1098294
87	0.00578484	0.2327864
88	0.37302729	0.4715389
89	0.03939827	0.1734206
90	0.01537291	0.0260149
91	0.0585906	0.0312937
92	0.14362212	0.1000388
93	0.04521314	0.0272821
94	0.36637881	0.2783804
95	0.09517923	0.0757915
96	-0.0893398	0.0141205
97	-0.8760776	0.2977111
98	0.03481847	0.0430276
99	0.03610411	0.0010754
100	0.25248731	0.1083561
101	0.05202098	0.0609321
102	0.43537085	0.2570625
103	0.21564167	0.2148129
104	0.29668373	0.1255558
105	0.19840808	0.048306
106	0.40043695	0.0239537
107	0.12974313	0.0783695
108	0.29165413	0.1535573
109	0.13321083	0.0247975
110	0.04240088	0.1823677
111	-0.2775666	0.0723896
112	0.37985711	0.0908993
113	0.01982276	0.0290007
114	0.30416838	0.0391526
115	0.2166427	0.0222474
116	0.63673348	0.2350071
117	0.57438478	0.4598221
118	0.34346016	0.144608
119	0.89152173	0.097777
120	0.26426434	0.1020306
121	0.98432791	0.1060243
122	0.27637682	0.0258988

123	0.61637695	0.1072341
124	0.16940972	0.1059234
125	0.11889846	0.0616568
126	0.02367914	0.0063834
127	0.05190576	0.0156058
128	0.12480208	0.0474999
129	0.08871436	0.0142311
130	0.03611673	0.0131158
131	0.03619205	0.0094528
132	0.219374	0.0689884
133	0.21358132	0.0188459
134	0.15515132	0.0266781
135	0.01369778	0.0264248
136	0.26140586	0.2933783
137	0.0282115	0.0557424
138	0.03335222	0.0491132
139	0.02218212	0.0135357
140	0.04831722	0.0165286
141	0.26349765	0.2161033
142	4.39488301	0.8960606
143	0.92103037	0.4271166
144	1.04394211	0.1021127
145	0.05725786	0.138818
146	0.06358578	0.0927573
147	-0.0042861	0.5150279
148	-0.029356	0.0877631
149	0.62602941	0.1234289
150	7.10006808	1.588477
151	4.57365874	1.0314479
152	0.28326189	0.2506008
153	4.65017684	0.9828555
154	1.96026111	0.6479274
155	0.49649701	0.2414208
156	2.82157856	0.2051331
157	0.90538137	0.8335107
158	-0.0105077	0.2366652
159	-0.0580322	0.1466839
160	0.07313384	0.0399063
161	-0.1596259	0.2051585
162	0.42621578	0.2777697
163	0.21587953	0.1007307
164	0.17377376	0.0352234
165	0.53727662	0.2654426
166	1.41105904	0.1202406
167	0.27551373	0.0878902
168	0.11798255	0.0430414
169	0.28548787	0.1043983
170	0.09975404	0.0624332
171	0.4221824	0.0368583
172	0.10189394	0.0078258
173	0.12926218	0.005344
174	0.18989535	0.0252946

175	0.14568259	0.0575129
176	0.11751151	0.0409289
177	0.19350165	0.0096294
178	0.05832576	0.0412368
179	0.41769969	0.0351527
180	-1.6313769	0.5115339
181	-0.0253733	0.1073963
182	0.26787439	0.2515935
183	0.25815885	0.1343076
184	0.48653065	0.1444369
185	0.89288555	0.3462754
186	0.21949225	0.1065151
187	0.1521251	0.0327848
188	0.2725428	0.0839917
189	0.31345078	0.0440747
190	0.12447281	0.05159
191	0.12201138	0.0520627
192	0.18328712	0.0191075
193	0.09202677	0.0442391
194	0.73670528	0.0469956
195	0.15596825	0.1077087
196	0.21557229	0.1130272
197	1.33071097	0.3308027
198	2.46037579	0.3663069
199	1.48189179	0.1588202
200	0.80342812	0.0654496
201	0.53440062	0.4153188
202	0.51440744	0.5212766
203	0.4879912	0.0574989
204	0.06191205	0.1260007
205	0.19856574	0.0296673
206	0.60604958	0.1268552
207	0.28298021	0.1968528
208	-0.2203923	0.1892872
209	0.49871604	0.1149462
210	0.23313645	0.1004143
211	0.17841553	0.0298908
212	0.32825961	0.0326744
213	0.28372111	0.3936026
214	0.64498635	0.0861717
215	0.26524485	0.4374861
216	0.25987179	0.1511531
217	0.4692591	0.1095936
218	0.15348924	0.125676
219	-0.0024607	0.0054182
220	-0.0625112	0.0340463
221	0.10529137	0.0562459
222	0.13357244	0.0307012
223	0.1002011	0.0399048
224	0.52858953	0.422207
225	1.0593074	0.6401974
226	0.19500528	0.1189507

227	0.61693023	0.1968022
228	0.18426271	0.079118
229	1.6451018	0.7495287
230	0.46373164	0.5581887
231	0.62957545	0.3649482
232	0.75676572	0.4194693
233	0.2305562	0.6667512
234	0.34153371	0.2240008
235	0.31927813	0.0465839
236	0.42819144	0.0422381
237	0.09959194	0.0433136
238	-0.0354926	0.0414496
239	-1.8974685	0.7161797
240	-0.0176654	0.0343545
241	1.03983741	0.7135317
242	0.37317495	0.1598387
243	0.22116402	0.148359
244	0.20568818	0.0143198
245	0.55986924	0.1031712
246	1.18157538	0.0225134
247	0.20078991	0.132971
248	0.44529648	0.2139394
249	0.21655518	0.0731756
250	1.48004786	0.1091922
251	1.32420248	0.4058173
252	-0.1164174	0.2591429
253	0.73421179	0.3530627
254	0.04737863	0.1604088
255	0.11983671	0.1990251
256	0.04203104	0.040784
257	0.14041597	0.0283978
258	0.11461827	0.0953746
259	0.20198524	0.2003696
260	0.12905075	0.0233918
261	0.23919721	0.1157406
262	0.35605186	0.0811832
263	0.1323316	0.0350591
264	0.25862055	0.230421
265	0.10594065	0.0287122
266	0.23216723	0.074159
267	0.07148957	0.0538598
268	0.0516485	0.0134338
269	0.11832794	0.1148963
270	0.24992502	0.120052
271	0.34252635	0.1259953
272	0.28004731	0.3911973
273	0.53333511	0.3939172
274	0.45723386	1.4787899
275	0.25807593	0.3331573
276	0.1928986	0.2475752
277	0.3207861	0.1433536
278	0.32298413	0.0254502

279	0.54304082	0.0707303
280	0.67569793	0.118631
281	1.29470003	0.11012
282	0.7150979	0.2090904
283	0.31974253	0.2257545
284	-0.0679249	0.1455229
285	0.09353133	0.0244743
286	0.13901335	0.0010224
287	0.11859967	0.1113723
288	0.16399572	0.1932869
289	0.31710112	0.1634015
290	0.09364465	0.0818205
291	0.55074133	0.1575031
292	0.35658298	0.3322378
293	0.07970379	0.0375936
294	0.17967498	0.1245548
295	-0.0660106	0.0252049
296	0.12721225	0.3568942
297	0.59487111	0.1473643
298	0.26746303	0.0431636
299	0.73792276	0.2011782
300	2.82787092	0.8038633
301	0.9185953	0.1704199
302	0.56253115	0.120103
303	0.5140518	0.6078411
304	1.38579728	1.2048338
305	2.17877134	1.7138143
306	0.69281962	0.1655271
307	1.87847794	0.9173289
308	0.73675802	0.2777321
309	0.89094118	0.2121726
310	0.54284694	0.1552076
311	0.23493186	0.1278745
312	-0.022395	0.0065835
313	0.43328004	0.2127105
314	0.36618795	0.0863051
315	0.14080197	0.0382395
316	0.04502028	0.0986493
317	0.18158475	0.0394102
318	0.16801461	0.063085
319	0.93319756	0.0815321
320	0.63416531	0.496419
321	0.13608315	0.0341204
322	0.25540561	0.0688337
323	0.80720572	0.2400142
324	0.2138452	0.0346486
325	0.73353772	0.119803
326	0.2860646	0.1011221
327	0.1093506	0.1374217
328	0.78102796	0.1368108
329	0.69927368	0.3256712
330	0.3115515	0.0366393

331	0.47415104	0.2218851
332	0.48227099	0.0991783
333	0.80123538	0.3269047
334	0.39962679	0.1236485
335	1.06302283	0.5096654
336	0.25865555	0.0524528
337	0.74750195	0.1356716
338	0.47730951	0.3898377
339	0.29253914	0.0870071
340	0.13494898	0.471807
	MONOMER	STDEV
nt position	+ GAG	
16	0.30003725	0.6109645
17	1.09872468	0.2692355
18	0.06911088	0.0610552
19	0.05846196	0.124712
20	0.14946229	0.2232164
21	-0.1919506	0.3234349
22	0.45822899	0.0550894
23	0.66851244	1.4073743
24	1.78194244	1.0545088
25	-0.7790846	3.6094924
26	-2.461583	3.6523706
27	-3.7353428	6.2423141
28	-1.0906761	2.0461237
29	0.17051447	0.1595108
30	3.22568555	4.2885843
31	0.67776774	0.3475162
32	0.97279045	0.3185884
33	0.20393577	0.1397965
34	0.41619635	0.226696
35	0.55242783	0.1071687
36	0.19414764	0.370151
37	-0.0329746	0.0383109
38	0.27564471	0.3343988
39	0.25351145	0.3183471
40	0.29869672	0.4733924
41	0.83018936	0.2087653
42	0.09797115	0.0489268
43	-0.3372542	0.0990624
44	-0.0444324	0.0058328
45	0.03809226	0.0533874
46	0.08474325	0.1999454
47	0.0726737	0.0967977
48	0.07791273	0.1527632
49	0.14008998	0.1398376
50	0.25609041	0.5111343
51	0.04255282	0.0385601
52	0.0778147	0.1204655
53	-0.034682	0.1794665
54	0.04636853	0.1094215
55	-0.051465	0.0676108

56	0.00818805	0.044601
57	0.05209531	0.0889762
58	0.06686618	0.0688674
59	0.2468594	0.152708
60	0.11069443	0.1371283
61	0.15546073	0.1264541
62	0.77513483	0.9888476
63	0.09507412	0.2617639
64	0.20344868	0.4452087
65	0.23562142	0.0238078
66	0.24428308	0.645887
67	0.39793387	0.0681887
68	0.71443022	0.0741826
69	0.07883059	0.2052827
70	0.07304497	0.0765401
71	0.17143867	0.1332646
72	0.16173692	0.2918166
73	0.05766376	0.4725241
74	0.59248206	0.5080654
75	3.89180558	3.0323578
76	0.59409965	0.2394105
77	0.40984898	0.1330055
78	0.48695235	0.1488043
79	0.35428015	0.1488137
80	0.05482731	0.0940416
81	0.03849101	0.0605393
82	0.08233596	0.13188
83	0.04376768	0.2262677
84	0.01176938	0.1257347
85	0.05591468	0.1130966
86	0.12251232	0.1253632
87	0.13115145	0.1904642
88	0.4401	0.7452506
89	0.10488567	0.0825402
90	0.16277878	0.2459083
91	0.11817502	0.2409914
92	-0.0040809	0.0846729
93	0.02467715	0.0563176
94	0.13450598	0.2650791
95	0.14065201	0.2044025
96	0.17194349	0.1778782
97	0.49247706	0.8426544
98	0.5973574	0.2530766
99	0.11880105	0.0604822
100	0.31840424	0.2386894
101	0.32873269	0.2216423
102	0.18631552	0.0688613
103	0.32372427	0.1298409
104	0.22489165	0.1015164
105	0.24656177	0.0876159
106	0.08968601	0.3241181
107	0.33988666	0.2246775

108	0.65947824	0.145506
109	0.14241519	0.193527
110	0.17493276	0.3725578
111	0.19858769	0.3002567
112	0.28652111	0.3763773
113	0.09228688	0.1272316
114	0.15488017	0.1149413
115	0.52161992	0.4239695
116	0.36150827	0.5292621
117	0.10214832	0.0826709
118	0.22340821	0.0153389
119	0.39584139	0.3163624
120	0.13550706	0.1936139
121	0.24552529	0.1056674
122	-0.0311203	0.0505027
123	0.11810924	0.033909
124	0.00151139	0.0228503
125	0.05841167	0.1172176
126	0.0569361	0.1207095
127	0.0292574	0.0437929
128	0.0874908	0.1086088
129	0.11540365	0.1439083
130	-0.0683852	0.1240045
131	0.06490654	0.0365558
132	0.7797116	0.1116629
133	0.55726168	0.0388679
134	0.25257138	0.085597
135	0.28534933	0.1549466
136	0.36315698	0.2430252
137	0.13920362	0.0378542
138	0.07696989	0.1097873
139	-0.0313947	0.0482257
140	0.06413553	0.1208893
141	0.03924209	0.038037
142	0.0974084	0.0943356
143	0.43400694	0.5080331
144	0.28644507	0.2449972
145	0.14659013	0.1900354
146	0.29963176	0.2937639
147	0.31352785	0.4015545
148	0.17560011	0.2252879
149	0.45054866	0.4849872
150	-0.5093226	0.9017596
151	-0.0845081	0.9008391
152	1.48576318	3.0202711
153	1.13495416	1.98441
154	0.23289139	0.8299108
155	0.01491802	0.0708327
156	1.25465749	0.9765847
157	2.4705334	2.6781281
158	0.38984266	0.1679246
159	0.21004656	0.1042574

160	0.30186686	0.453069
161	0.18688139	0.2610078
162	0.26111114	0.0889003
163	0.4415007	0.2067577
164	0.10466179	0.0733821
165	0.35688509	0.0845236
166	0.40420468	0.1143009
167	0.2050591	0.0773538
168	0.11200313	0.1188468
169	0.37708651	0.0790324
170	0.10987459	0.1115194
171	1.12650391	0.2389256
172	0.34686989	0.1707054
173	0.31823062	0.0754397
174	0.16774682	0.1843396
175	0.27454741	0.2539773
176	0.18928092	0.1504522
177	0.24202258	0.2807634
178	0.18561804	0.044851
179	0.3146314	0.4419186
180	0.13044406	2.2964773
181	0.97918028	0.2991375
182	0.70492131	0.1611599
183	0.82393567	0.1811702
184	1.00928872	0.3378954
185	0.68910507	0.5916575
186	0.1170261	0.1765468
187	0.08715147	0.0597113
188	0.073717	0.0441287
189	0.06466544	0.1569615
190	0.14433702	0.0979898
191	0.94671164	0.5535594
192	1.1377084	1.8663737
193	3.14642248	4.5747819
194	0.54319354	0.4682094
195	0.10116703	0.3383431
196	0.05188357	0.1205932
197	0.16410394	0.1991787
198	0.23268725	0.1569899
199	0.1811204	0.4670778
200	1.06538895	0.9038355
201	1.8323701	0.5914511
202	1.28281804	0.7446119
203	0.49525602	0.4806288
204	0.47014701	0.2350649
205	0.63806644	0.1738035
206	0.57180636	0.1615892
207	0.60866141	0.1099905
208	0.90724568	1.1049235
209	0.7373914	0.456678
210	0.9951111	0.0756533
211	0.62669532	0.3719783

212	0.66364298	0.1278811
213	0.88424607	0.4648498
214	0.59008374	0.259367
215	0.95779995	0.1358361
216	1.16180173	0.2518796
217	0.19779559	0.1596632
218	0.09703149	0.1912639
219	0.30974971	0.7121353
220	0.12740255	0.041275
221	0.01644714	0.0411612
222	0.02931763	0.029821
223	0.06884545	0.0935508
224	0.14715451	0.0651571
225	0.01320837	0.0859778
226	0.2477589	0.0844822
227	-0.0019252	0.0156692
228	0.37683388	0.6738398
229	0.21364815	0.8748006
230	1.03586659	1.6738221
231	3.22921984	4.8632856
232	1.20632207	3.4318809
233	0.51792297	0.69287
234	0.18669529	0.0664621
235	0.19674609	0.0389632
236	0.19669611	0.0310156
237	0.19584306	0.1187193
238	3.17998473	1.2795929
239	0.82991768	0.4251506
240	0.45030064	0.04424
241	0.24856479	0.098372
242	0.33433241	0.0427975
243	0.50390731	0.1376773
244	0.21930168	0.169518
245	1.61784586	0.1494076
246	0.16263362	0.1691901
247	0.56233579	0.107414
248	0.13008887	0.0598987
249	0.85625659	0.4400876
250	0.34162952	0.6028229
251	0.34972961	0.3369961
252	0.24817646	0.1577833
253	0.32819254	0.2694455
254	0.08753166	0.0676717
255	0.17183426	0.0307582
256	0.10315826	0.0451273
257	0.17703936	0.0158297
258	0.00662429	0.0056865
259	0.08346339	0.0872013
260	0.0573824	0.0207588
261	0.10021208	0.061103
262	0.09841303	0.2363327
263	0.05971369	0.0317293

264	0.1151726	0.0609743
265	0.10901105	0.0422441
266	0.09599792	0.0965208
267	0.28029038	0.3581124
268	0.05896562	0.0586502
269	0.20573642	0.0932768
270	0.13144878	0.0270803
271	0.42822997	0.0511675
272	0.67284823	0.2589097
273	2.10908862	2.3077699
274	1.31581154	1.6124141
275	0.54730296	0.2095054
276	0.43355621	0.063457
277	0.20585525	0.1123829
278	0.20054111	0.0031754
279	0.15020219	0.0533412
280	0.16319898	0.0463905
281	0.08060114	0.1304872
282	0.65541319	0.517533
283	0.64800845	1.3271914
284	0.75858687	1.2200952
285	0.54147365	0.3842421
286	0.37744751	0.1285484
287	0.31003446	0.1182557
288	1.01760963	0.4275998
289	0.60476366	0.2451578
290	0.44354403	0.0658887
291	1.44192924	0.5235602
292	1.28766661	0.0907058
293	0.15607347	0.1785243
294	0.35191326	0.2060952
295	2.11957106	1.3825611
296	0.51715223	0.1163064
297	0.15138163	0.0794797
298	0.79200012	0.3546189
299	0.41960281	0.3705993
300	0.2662093	0.1225078
301	0.19518278	0.0455469
302	0.14772502	0.0452986
303	0.3585549	0.0336627
304	1.7363739	0.735857
305	1.47877186	0.6922749
306	0.64487648	0.3118258
307	1.04817941	0.4386736
308	0.38705736	0.2177476
309	1.68046627	0.3632711
310	0.49466017	0.1591376
311	0.65993551	0.179313
312	0.07762737	0.0743036
313	0.48856564	0.1331622
314	-0.0207209	0.1342057
315	-0.067072	0.1004296

316	-0.0236625	0.0236451
317	0.1754409	0.1505863
318	0.33063025	0.1493152
319	1.1746533	0.3962799
320	1.11598432	0.4108204
321	-0.0128811	0.1407572
322	-0.108265	0.0560096
323	0.08303597	0.0376684
324	0.10166513	0.0369445
325	-0.017038	0.112857
326	0.5993971	0.3404752
327	0.29936162	0.0812261
328	0.18878635	0.0465595
329	0.12468816	0.1883862
330	0.07253394	0.1853059
331	-0.064395	0.1538848
332	0.79708846	0.7368213
333	0.61020009	0.4683576
334	1.27933728	1.1405134
335	1.39109825	1.0054719
336	0.34666599	0.5450952
337	0.61495751	0.1497505
338	0.15800765	0.5938958
339	0.30102156	0.2528227
340	0.01831439	0.1319896
341	0.02977996	0.085967
342	-0.0883491	0.0827363
343	0.06481212	0.1905726
344	0.41155339	0.2811199
345	-0.028346	0.1253698
346	0.39648349	0.2592906
347	1.36333379	0.6342337
348	-0.3452748	0.2963584
349	0.96692322	0.9709317
350	-0.1515561	0.0881114
351	0.22995255	0.6872252
352	0.56182897	1.3092321
353	0.09893379	0.2419559
354	0.62721049	0.1523108
355	4.74158816	5.3591668
356	0.18871798	0.5976791

nt position	DIMER	STDEV
10	-1.5091861	3.3353857
11	0.04695004	0.4388164
12	-0.1006607	0.1202374
13	0.14955714	0.2478588
14	0.21129856	0.0997011
15	0.22167714	0.8373627
16	0.26114476	0.1242793
17	0.43660259	0.1575201
18	0.03341339	0.1247217
19	0.27532484	0.1329647

20	0.04277549	0.0346823
21	0.13308471	0.1230173
22	0.55151478	0.2260389
23	0.89464866	0.1825081
24	0.76350901	0.543063
25	0.86925626	0.6385925
26	0.58939422	0.665692
27	0.95261417	0.9102378
28	0.3755073	0.0092698
29	0.16243758	0.1565657
30	0.14686635	0.1842703
31	0.24033916	0.0918247
32	0.69151356	0.239424
33	0.256187	0.2546462
34	0.50999449	0.3595083
35	0.71969514	0.3263161
36	0.19857082	0.1752578
37	0.02090933	0.028837
38	0.04378226	0.1030686
39	0.07956071	0.1362419
40	0.07455647	0.4594298
41	0.49657634	0.3577418
42	0.24374749	0.3855984
43	0.23171072	0.1901585
44	0.22353193	0.1816152
45	0.08677787	0.109796
46	0.13324041	0.1290029
47	0.23478203	0.1102285
48	-0.0730878	0.2746756
49	0.12725712	0.0767378
50	0.26003818	0.1748542
51	0.02565376	0.0922924
52	0.09379279	0.3474319
53	0.13431179	0.0945733
54	0.26918463	0.1418899
55	-0.0220042	0.3136088
56	0.40105471	0.2320807
57	0.14876844	0.0319548
58	0.26221538	0.1036001
59	0.12299779	0.0253873
60	0.07348131	0.0556217
61	0.29973474	0.170064
62	0.65741292	0.5611282
63	0.16863216	0.1291806
64	0.24951762	0.1009091
65	0.69885447	0.5119069
66	0.91838401	0.6770307
67	0.20481743	0.2342176
68	0.18890608	0.1729729
69	0.07710878	0.0391775
70	0.04558226	0.0261182
71	0.08343518	0.0839405

72	0.326694	0.031374
73	0.41454704	0.254297
74	0.48231849	0.1254395
75	2.08238978	0.2781769
76	0.64060809	0.6232221
77	0.60727222	0.4247846
78	0.58507073	0.0914463
79	0.29136777	0.0582645
80	0.05544616	0.0230095
81	0.10326854	0.0589224
82	0.2557721	0.1237524
83	0.36018181	0.0479021
84	0.19131665	0.0834022
85	0.16849821	0.0647214
86	0.07737473	0.149149
87	0.01933444	0.2274967
88	0.33873505	0.2338424
89	0.23594262	0.124371
90	0.35176272	0.275305
91	0.48635458	0.2671107
92	0.3590896	0.1305908
93	0.09899351	0.0904342
94	0.21578764	0.2189642
95	0.15733723	0.1182579
96	0.42552871	0.1291884
97	0.52683706	0.2726404
98	0.3587609	0.0661494
99	0.05728567	0.0150658
100	0.23293746	0.1500883
101	0.95068986	0.8423009
102	0.57707339	0.4214777
103	0.81582625	0.474855
104	0.9249478	0.6528466
105	1.27694407	0.2303053
106	1.26986555	1.1342232
107	0.63231212	0.475964
108	0.88941564	0.5582105
109	0.11833979	0.0759482
110	0.20556368	0.1039258
111	0.26844133	0.0290544
112	0.45503957	0.3541947
113	0.20019788	0.077692
114	0.53470966	0.5706162
115	0.36051911	0.4522488
116	0.21259865	0.0960343
117	0.15017036	0.0656437
118	0.15194749	0.0641577
119	0.43939788	0.4556566
120	0.047733	0.0219166
121	0.40675536	0.4316153
122	-0.0245767	0.0804338
123	0.21831276	0.2893509

124	0.21934123	0.1196603
125	0.17240254	0.1341805
126	0.08700477	0.0523836
127	0.10920247	0.0432243
128	0.42076799	0.3132864
129	0.36683944	0.07772
130	0.25012358	0.400917
131	0.27565038	0.0173405
132	1.18954572	0.8387034
133	0.59475215	0.3909992
134	0.11021826	0.0386435
135	0.05995733	0.0198039
136	0.13093306	0.0739472
137	0.16214885	0.2154785
138	0.09945758	0.0633008
139	0.10340185	0.1530089
140	0.06396656	0.0367403
141	0.11144223	0.08703
142	0.17720948	0.0836552
143	0.16603527	0.1594382
144	0.06379649	0.138107
145	-0.0571778	0.13839
146	0.9241429	0.4800952
147	0.58597362	0.4801534
148	0.17392727	0.1205626
149	0.38366867	0.6733133
150	2.34109049	5.2859995
151	1.74866121	3.028982
152	0.0589269	0.3183825
153	0.26724256	0.0256039
154	0.21923233	0.141453
155	0.3075617	0.0713617
156	1.98347761	1.0697426
157	1.25658616	0.8344813
158	0.52415459	0.1847633
159	0.16545785	0.1077786
160	0.68951517	0.4071989
161	0.33105586	0.3339962
162	0.41625098	0.3103094
163	1.08933421	0.5523831
164	-0.063112	0.34322
165	0.5594881	0.1684072
166	0.39312838	0.4932162
167	0.3081261	0.2725204
168	0.2980558	0.0957525
169	0.41956482	0.3994301
170	0.30923191	0.4455253
171	0.72631204	0.4310639
172	0.25694542	0.044228
173	0.14947447	0.111355
174	0.08444406	0.0460289
175	0.01381145	0.1282304

176	0.19402369	0.4518977
177	0.27102525	0.3097745
178	0.1122356	0.1660612
179	0.24649194	0.3254126
180	1.70564452	3.0365833
181	0.37914816	0.2712343
182	0.44481532	0.1792575
183	0.316695	0.176618
184	0.50827019	0.361395
185	0.38196215	0.2473628
186	0.00578116	0.1636049
187	-0.0694365	0.1981046
188	-0.0703531	0.194694
189	-0.1408385	0.4478867
190	-0.0922778	0.3695278
191	0.53707487	0.2816262
192	0.81146187	0.3175094
193	0.9421259	0.5504053
194	0.17103513	0.4261784
195	-0.0812984	0.4319157
196	-0.2140594	0.3978533
197	-0.0402357	0.3641209
198	0.01962426	0.1031565
199	0.08565825	0.3280594
200	0.30958256	0.1726305
201	1.80971764	1.3732762
202	1.18615594	0.5102764
203	0.43132977	0.2182779
204	0.21308441	0.1372202
205	0.26128035	0.3315133
206	0.75120482	0.4771411
207	1.40185144	1.1548557
208	1.44832655	2.1244354
209	0.24819789	0.1180415
210	0.37615458	0.1484363
211	0.2921156	0.1377764
212	0.30585727	0.1382034
213	0.23119249	0.2386875
214	0.208635	0.0331869
215	0.375316	0.152826
216	0.40807123	0.1547436
217	0.23635203	0.1623333
218	0.2741659	0.1500304
219	0.66549614	0.4440102
220	1.85591276	2.0005922
221	0.31540738	0.2083004
222	0.13042628	0.0536316
223	0.19178216	0.1275491
224	0.35861965	0.388823
225	0.41097639	0.4677021
226	0.29290292	0.0456726
227	0.15340287	0.2697025

228	-0.0253795	0.1217207
229	0.16638109	0.4311349
230	0.20418413	0.1208946
231	0.24996069	0.2998824
232	0.58724105	0.6022389
233	0.05825873	0.0397779
234	0.31772634	0.2567084
235	0.13827105	0.0974805
236	0.43620534	0.2041079
237	0.40415419	0.3588308
238	1.87425449	2.2691897
239	1.66559965	3.0778669
240	2.0710225	2.416491
241	2.79872031	1.5257885
242	0.57421834	0.4944599
243	0.1973487	0.2782353
244	0.12677688	0.1218189
245	0.01280975	0.1880676
246	0.19416206	0.4150958
247	0.15697234	0.1282107
248	0.07789097	0.0998456
249	0.03653428	0.0702223
250	0.06236687	0.1289449
251	0.0069573	0.0566963
252	0.10575484	0.1590947
253	0.07828602	0.2869353
254	0.1995039	0.1337459
255	0.55921861	0.1547165
256	0.52852374	0.1313131
257	0.13106942	0.2305769
258	0.02791479	0.0870129
259	-0.0613283	0.0522682
260	0.05936634	0.0951326
261	0.06780703	0.0683655
262	0.07232476	0.0352324
263	0.45851823	0.6552997
264	0.26620691	0.3295446
265	0.16740956	0.1863003
266	0.1223415	0.0380511
267	0.13553846	0.0943841
268	0.03803151	0.0309706
269	0.16640221	0.0919846
270	0.0818928	0.1529569
271	0.44651664	0.1362968
272	0.90489402	0.120794
273	1.04136399	1.1811853
274	-0.021546	0.1760735
275	-0.0320247	0.2217937
276	0.10036175	0.0744392
277	0.00808985	0.141678
278	0.36617715	0.1775576
279	0.46254686	0.2005224

280	0.25706136	0.0485392
281	-0.0318855	0.3360828
282	0.06479	0.237193
283	0.30777588	0.4860114
284	1.16424821	0.2163258
285	0.05765941	0.4303091
286	0.06164223	0.1380661
287	0.09768129	0.3165212
288	0.45145535	0.3705347
289	0.44518383	0.3939603
290	0.34486232	0.3069456
291	-0.7480312	1.5659798
292	0.71611033	0.306899
293	0.04357282	0.0069665
294	0.31914735	0.1506162
295	1.20628419	1.8309819
296	0.62587315	0.1160218
297	0.48105787	0.4138681
298	0.25378324	0.3839238
299	1.01000553	1.8785158
300	0.51478602	0.3450092
301	0.18216696	0.1596552
302	0.14628881	0.0360188
303	-0.2377358	0.7059468
304	-0.8727969	1.3979613
305	-0.3378886	0.8987634
306	-0.0395535	0.2802691
307	0.29928439	0.2349642
308	0.55900844	0.0923326
309	2.76320548	2.2178919
310	0.75602162	0.3585871
311	0.38909338	0.1873621
312	-0.021043	0.0677465
313	0.1542273	0.3322769
314	0.13558874	0.0473193
315	1.07800377	0.9535393
316	2.86424667	2.3152673
317	0.51592211	1.1164936
318	0.80315172	0.4729163
319	1.11589428	1.3153209
320	2.16427464	1.9671914
321	0.29196977	0.6035719
322	-0.1446764	0.1783793
323	0.18040497	0.2950101
324	0.40586695	0.1079961
325	0.19488905	0.4046984
326	0.37190686	0.4449171
327	0.17594795	0.1179038
328	0.28970451	0.2034436
329	0.60892998	0.5228294
330	0.10180687	0.4811982
331	-0.0064364	0.1145031

332	0.77797982	0.7208118
333	0.31927018	0.2645798
334	0.61779243	0.4385336
335	0.57945282	0.334522
336	0.38639162	0.515107
337	1.30515443	0.7279677
338	0.16741099	0.5380135
339	-0.1335193	0.1005449
340	0.55512167	0.1459973
341	0.22872701	0.1861909
342	0.15581646	0.3221439
343	0.12788735	0.9678474
344	1.19271888	0.4888172
345	0.02323654	0.0520451
346	-0.023859	0.1607692
347	1.5881485	0.1474113
	DIMER	STDEV
nt position	+ NC	
19	0.53222418	0.7969628
20	0.099634	0.0608395
21	0.21755411	0.2936644
22	0.43203829	0.4675295
23	-0.3045862	0.6386803
24	-0.0704901	0.2592849
25	0.06872596	0.3162451
26	0.2241172	0.2268401
27	0.79087421	1.1722241
28	0.23963087	0.1910228
29	0.03165096	0.1101708
30	0.94738852	1.3294555
31	0.51754203	0.2559548
32	0.63316637	0.5068248
33	0.25252006	0.1579788
34	0.27603141	0.4439666
35	0.34179718	0.2278037
36	0.26943541	0.4822305
37	0.19463565	0.2780405
38	0.39469207	0.5075119
39	0.80752477	1.1438896
40	1.10396917	1.0407618
41	1.31814905	2.2848109
42	0.63959328	0.451536
43	0.16475045	0.2171109
44	-0.0114773	0.113234
45	0.18382135	0.3025631
46	0.12894132	0.0827975
47	0.10278475	0.0900214
48	0.04388305	0.0378447
49	0.12326399	0.2197151
50	0.30475855	0.2930724
51	0.10592726	0.076649
52	0.28501241	0.2290727

53	0.12494402	0.1587218
54	0.55683597	0.3643154
55	0.39320397	0.3791436
56	0.3712055	0.2135198
57	0.44145733	0.2563402
58	0.33879939	0.0425117
59	0.36723244	0.167969
60	0.27051793	0.1741053
61	0.20151864	0.0541151
62	0.40754241	0.4490504
63	0.41640365	0.1703654
64	0.21314394	0.180082
65	0.00819526	0.3485343
66	-0.323704	1.1396871
67	0.20299655	0.1432304
68	0.37946273	0.3218418
69	0.21960467	0.1831759
70	0.2130497	0.1971307
71	0.36432802	0.3810817
72	0.24107592	0.2386883
73	0.46310111	0.1993699
74	0.39529322	0.1761362
75	0.01432809	2.1635002
76	0.47275369	0.0250064
77	0.32950762	0.2765488
78	0.59250271	0.3180236
79	0.75028943	0.0881043
80	0.15190014	0.0232035
81	0.13199042	0.0862552
82	0.38975344	0.200516
83	0.66965543	0.097995
84	0.47384782	0.251909
85	0.31314649	0.2842244
86	0.46301975	0.098269
87	0.13045697	0.2137713
88	0.23185005	0.0885908
89	0.33351546	0.103749
90	0.15849097	0.1510764
91	0.16424471	0.1005177
92	0.36406683	0.0933847
93	0.38055623	0.2066148
94	0.29796057	0.1708319
95	0.4182997	0.2700838
96	0.46743039	0.0193911
97	1.31473428	0.1493918
98	0.76516838	0.036499
99	0.33799129	0.2383643
100	0.23477293	0.3049819
101	0.01049812	1.2596389
102	0.37582268	0.0615963
103	-0.0104484	0.5435406
104	0.00016804	0.3995859

105	-0.0700505	0.486491
106	0.10844324	0.1033902
107	0.19160798	0.053805
108	0.12136191	0.2981494
109	0.16769435	0.1930783
110	0.22245441	0.1065266
111	0.23039325	0.0096713
112	0.11913021	0.1188241
113	0.22654946	0.1780677
114	0.24616751	0.0614858
115	0.2497829	0.1598417
116	0.22995508	0.0814831
117	0.22497557	0.0859436
118	0.4617186	0.2375688
119	1.24213892	0.516917
120	0.28870667	0.133641
121	0.67662784	0.1132727
122	0.12023758	0.1022863
123	1.38387953	1.1672156
124	0.43447031	0.0153874
125	0.25017888	0.1277739
126	0.1582313	0.0056484
127	0.05074442	0.0293969
128	0.17229909	0.1675992
129	0.13969489	0.1297849
130	-0.0246162	0.1370171
131	-0.0094675	0.3165281
132	-0.6744186	1.8587315
133	0.16161136	0.0945738
134	0.14650282	0.023419
135	0.1068241	0.1426541
136	0.28365728	0.1963218
137	0.27432576	0.2470871
138	0.08294927	0.0446432
139	0.2577487	0.2312798
140	0.22132971	0.1816952
141	0.3678185	0.2740798
142	0.82820999	0.3246746
143	0.56151548	0.1602821
144	0.34031898	0.4323525
145	0.26273473	0.4488262
146	0.40075197	0.4210824
147	0.54058246	0.1518425
148	0.49549438	0.2927425
149	0.38487991	1.0138676
150	1.40049096	3.1795572
151	0.26212046	0.9498026
152	0.27406523	0.686187
153	-0.0823478	0.2104904
154	0.59150291	0.9090577
155	0.4253095	0.3850454
156	2.02214887	0.7616326

157	3.13605777	2.1044893
158	0.82912465	0.061085
159	0.39786943	0.1195546
160	1.86071325	1.5726387
161	0.58273786	0.2267103
162	0.48989201	0.0177136
163	0.03489293	0.4951164
164	0.10526848	0.1336528
165	0.36712194	0.053074
166	0.52199843	0.2348101
167	0.10652403	0.1684056
168	0.258557	0.0866276
169	0.40108025	0.3594006
170	0.20656535	0.1075172
171	0.5211491	0.1627259
172	0.32589799	0.0340798
173	0.08261615	0.1184989
174	0.05209383	0.1223939
175	0.01815027	0.0645859
176	0.13099172	0.1105288
177	-0.0802179	0.2265082
178	0.07945288	0.0862335
179	0.42404278	0.3965792
180	1.84977039	1.3084615
181	0.598704	0.7115863
182	0.06878913	0.5592671
183	0.48046168	0.5664634
184	0.38800964	0.4177774
185	1.81450701	1.0339297
186	0.39398748	0.1961601
187	0.41295717	0.3015789
188	0.37679597	0.4279216
189	0.27287605	0.4706614
190	0.16007882	0.1748562
191	0.40346424	0.4952382
192	1.36409707	1.3176137
193	4.13034983	7.10915
194	0.94654201	0.9936897
195	0.36040645	0.2316762
196	0.23295701	0.1126254
197	0.56507781	0.6915022
198	0.27866894	0.4284031
199	0.33947723	0.420707
200	0.1596821	0.2894718
201	0.46161949	1.0734851
202	0.60956113	0.882744
203	0.73174196	0.611458
204	0.47434605	0.2976918
205	0.99067895	0.2113131
206	0.91848201	0.4715417
207	0.66474285	0.1043252
208	1.42994362	1.1276922

209	0.61733048	0.3107279
210	0.96442901	0.5562596
211	0.99313783	0.6296004
212	0.64395325	0.5393828
213	0.38215844	0.4708467
214	0.3487228	0.3277124
215	0.61421691	0.3407221
216	1.04425768	0.7940207
217	0.85966208	1.0461256
218	0.00919722	0.2083047
219	0.1228968	0.4723025
220	-0.0902271	0.2711979
221	0.00460327	0.2877027
222	0.16097009	0.0912377
223	0.12034824	0.1191703
224	0.27154843	0.2102535
225	0.43867435	0.4528977
226	0.39196357	0.0611701
227	0.36984049	0.2610091
228	0.00509824	0.0675331
229	0.22127249	0.4780385
230	0.06781299	0.1282967
231	-0.0591876	0.0796624
232	0.50699911	0.6662701
233	0.6096382	0.6565547
234	0.317998	0.0432153
235	0.18313211	0.0296235
236	0.14155846	0.2416806
237	-0.0406336	0.3922027
238	-0.1697636	0.4088602
239	0.80754993	1.8847142
240	0.82302969	0.4259609
241	1.13707595	0.9493334
242	0.56147953	0.4383689
243	0.28102595	0.0998435
244	0.11402989	0.1041739
245	0.22214126	0.2288203
246	0.2848067	0.2731831
247	0.41733255	0.4143833
248	0.58839971	0.3877954
249	0.30412792	0.3697984
250	0.26103201	0.1789874
251	0.08850792	0.0882236
252	0.01402638	0.015503
253	-0.0041297	0.0925224
254	0.16268957	0.3539922
255	0.42869884	0.3228535
256	0.65498379	0.0172467
257	0.26413075	0.3124491
258	0.11728438	0.0793059
259	0.02870015	0.1586412
260	0.10069301	0.3971942

261	0.24687059	0.211974
262	0.09778944	0.070948
263	0.23808854	0.1246088
264	0.37191942	0.1682366
265	0.35171162	0.3809118
266	-0.0881034	0.2963386
267	-0.3959242	0.9598
268	0.00708936	0.0491394
269	0.03612128	0.1428564
270	0.05620625	0.1163095
271	0.12673826	0.1545017
272	0.11567434	0.4619504
273	0.05016087	1.1145951
274	-0.216932	0.860586
275	0.04382487	0.1527112
276	0.03251951	0.2283366
277	0.20689515	0.2200356
278	0.27030452	0.220377
279	0.26474312	0.5653209
280	-0.3839532	0.5894762
281	0.21661855	0.4922044
282	0.22278998	0.2658553
283	0.14487169	0.1454089
284	0.09484446	0.1056205
285	-0.0414281	0.2500477
286	0.02569724	0.1179803
287	0.00345937	0.2867766
288	0.39809653	0.8051441
289	1.15383859	0.8874056
290	0.45807373	1.0509771
291	0.80874725	0.7913057
292	0.63753469	0.1671675
293	0.08547196	0.0666766
294	0.3849454	0.1020511
295	0.09107083	1.0445531
296	0.11707881	1.797893
297	1.1740011	1.5006371
298	0.32389853	0.2911773
299	-0.0425002	0.1621852
300	-0.2328683	0.4684541
301	0.19648026	0.3565636
302	0.22479273	0.1962852
303	0.57011787	0.0872963
304	1.61515924	0.7422977
305	2.15753936	0.830039
306	0.50755337	0.9588117
307	1.09936314	0.7728668
308	1.30297608	1.0184124
309	3.19191192	2.7927514
310	0.28698828	0.7744921
311	0.24360635	0.0424004
312	0.05409706	0.0538896

313	0.51224891	0.6417811
314	0.06504459	0.3279238
315	0.09044658	0.2097432
316	-0.1624623	0.3692755
317	0.74883394	0.1407889
318	0.45664574	0.2781991
319	1.958515	1.739111
320	1.44899442	1.8423125
321	0.03269062	0.28227
322	-0.0041818	0.1112522
323	0.12282724	0.4647093
324	0.06684702	0.2081289
325	0.02593317	0.3199322
326	0.03245395	0.5440787
327	0.19195328	0.1447751
328	0.44253622	0.4710946
329	-0.1567404	0.6627959
330	0.03731087	0.104245
331	-0.2158209	0.1692989
332	0.16322336	0.1018609
333	0.10367667	0.0858936
334	0.18293948	0.1354731
335	0.4956701	0.2894705
336	0.25046106	0.0571368
337	0.84477457	0.5790063
338	0.93146756	0.2888088
339	0.52411173	0.3432191
340	0.34173794	0.1963007
341	0.19017711	0.5230095
342	0.44928846	0.0579031
343	0.6786825	0.3656529
344	-0.3493193	0.1786182
345	0.50547236	0.5207062
	DIMER	STDEV
nt position	+ GAG	
13	0.27920937	0.1966122
14	0.04818577	0.0617967
15	0.02847024	0.0498594
16	0.51721113	0.2663134
17	0.5185746	0.5832225
18	0.17467792	0.118569
19	0.13637279	0.1416303
20	-0.0327669	0.0983138
21	0.31794744	0.4086698
22	0.93351923	0.5446066
23	1.94965315	0.9422823
24	1.14268639	0.7409589
25	0.57693138	0.8007204
26	0.27610682	0.3754391
27	0.03939548	0.1388566
28	0.00379796	0.05897
29	0.01207814	0.2309817

30	2.17704414	2.5938808
31	0.47981677	0.1324861
32	1.13603626	1.4026504
33	0.22641137	0.1074812
34	0.81309588	0.3861418
35	0.36988038	0.4350912
36	0.04461588	0.0508727
37	0.04863628	0.0497179
38	0.05122395	0.0460863
39	0.18910528	0.113313
40	1.44655573	1.2616709
41	1.2655657	1.1897349
42	0.27888038	0.3260208
43	0.21331289	0.1421862
44	0.09910876	0.1202702
45	0.05650747	0.1257909
46	0.21653166	0.0184712
47	0.09851951	0.0860852
48	0.05653873	0.078752
49	0.14628554	0.2055746
50	0.00365704	0.1603307
51	0.18028721	0.1287277
52	0.08413294	0.195211
53	0.15323494	0.1337563
54	0.11176844	0.115385
55	0.18747807	0.0966289
56	0.08472977	0.1141015
57	0.17093224	0.1106915
58	0.21401597	0.1163133
59	0.0848967	0.0506733
60	0.1126852	0.1136731
61	0.4303268	0.4892182
62	0.28930275	0.3904027
63	0.21049026	0.0060939
64	0.24539263	0.4054386
65	1.37643027	1.8071381
66	0.46914196	0.1441794
67	0.28287236	0.2231019
68	0.11181115	0.0760138
69	0.11658391	0.0245971
70	0.19066125	0.2079678
71	0.21750028	0.1959213
72	0.3491573	0.3749229
73	0.49496253	0.3487633
74	1.9829924	1.8759248
75	0.23988947	0.4643551
76	0.61378369	0.2626705
77	0.72560208	0.2280504
78	0.57952526	0.2075165
79	0.26433082	0.1669043
80	0.09952345	0.0982521
81	0.15169959	0.1203317

82	0.43160715	0.1660446
83	0.39545915	0.1594207
84	0.20785647	0.1838207
85	0.25523719	0.1866481
86	0.16235459	0.1427171
87	0.21660012	0.2068674
88	0.21450879	0.146758
89	0.11239314	0.1000646
90	0.15958814	0.1579907
91	0.39058708	0.2130902
92	0.26256798	0.1989964
93	0.0802941	0.0176862
94	0.10411103	0.0831766
95	0.04984175	0.0651408
96	0.24517111	0.2430486
97	0.27700228	0.1893105
98	0.19312188	0.197286
99	0.16220367	0.1050768
100	0.07265125	0.1830916
101	-0.0189815	0.5213937
102	0.50499575	0.1292717
103	0.5695726	0.4237288
104	0.350685	0.3703486
105	0.6377732	0.4159131
106	0.8278198	0.5488513
107	0.87739467	0.0403979
108	0.820284	0.2233449
109	0.2102302	0.0776659
110	0.154335	0.1303979
111	0.144268	0.0801516
112	0.1326572	0.1052201
113	0.1069518	0.1164055
114	0.2586092	0.1509903
115	0.193288	0.093089
116	0.23141933	0.0353994
117	0.2909654	0.0883904
118	0.4348988	0.0883587
119	1.5381865	0.2361768
120	0.1776458	0.0787057
121	0.8866415	0.0815796
122	-0.0167565	0.0203982
123	0.525539	0.2316345
124	0.35297525	0.0687685
125	0.2212018	0.063844
126	0.0868954	0.0522791
127	0.0309878	0.0253802
128	0.01471725	0.0372068
129	0.019414	0.0473349
130	0.09317725	0.0991251
131	-0.028522	0.0758765
132	-0.0013045	0.6290666
133	0.8369764	0.187624

134	0.3241434	0.1099017
135	0.0901204	0.0264235
136	0.071121	0.0498817
137	0.0318652	0.0365335
138	0.069275	0.0804446
139	-0.0093462	0.0249164
140	0.0432474	0.0741569
141	0.03795175	0.0321932
142	0.026877	0.0335353
143	0.030602	0.0152132
144	0.0445566	0.1869933
145	0.0119745	0.0199033
146	0.0785094	0.1368562
147	0.07979075	0.1685728
148	0.0197164	0.3085259
149	0.219335	0.6211989
150	-1.649506	4.6573096
151	-0.2208836	1.4202044
152	0.48608067	0.0905003
153	0.59060967	0.0716565
154	0.4083018	0.2265834
155	0.0832906	0.0290926
156	0.8222528	0.6601592
157	1.671303	0.7243296
158	0.502794	0.0191727
159	0.2947534	0.1033136
160	0.0071798	0.6926449
161	-0.1435948	0.2955636
162	0.36307	0.094065
163	0.4238086	0.21751
164	0.0812435	0.0416745
165	0.567176	0.1235886
166	0.4785635	0.243932
167	0.5729002	0.5629899
168	0.1700696	0.1127952
169	0.76496275	0.3012039
170	0.0687796	0.0578924
171	1.242885	0.118093
172	0.292566	0.0256202
173	0.201603	0.0741535
174	0.0230714	0.0375947
175	0.0543408	0.0319303
176	0.0669526	0.0869497
177	0.0853992	0.3373165
178	0.053488	0.0855615
179	0.10695375	0.2021711
180	-1.20095	0.5936287
181	0.72126	0.7049286
182	0.105435	0.0808806
183	0.30958093	0.2298463
184	0.5763278	0.0884028
185	0.84654324	0.3741771

186	0.24792019	0.3069894
187	0.19065225	0.150708
188	0.13427044	0.1826097
189	0.1445242	0.3457478
190	0.1509858	0.2459752
191	0.3623792	0.1946346
192	0.73947582	0.2259146
193	-0.0133996	2.0746889
194	0.01462573	0.3375266
195	0.28375631	0.1676381
196	0.05405675	0.034578
197	0.5796113	0.2331396
198	0.1788654	0.3145838
199	0.27117667	0.0183812
200	0.1018646	0.1620585
201	0.88639434	0.8164379
202	0.94473176	0.653719
203	0.83365027	0.6175787
204	0.3014188	0.1055779
205	0.8970198	0.3924018
206	0.69089905	0.6764644
207	0.31281671	0.438138
208	-0.5120876	1.7364337
209	0.19843611	0.3281451
210	1.0260392	0.8119164
211	1.2189308	0.7902188
212	0.55151476	0.2547781
213	0.77082531	0.7956022
214	0.43858694	0.2419982
215	0.42797054	0.3649098
216	0.90660726	0.5108425
217	1.19248997	0.6390556
218	0.3009235	0.2118325
219	0.13563828	0.0969778
220	-0.1612218	0.8611268
221	0.16805561	0.0823852
222	0.119077	0.0475947
223	0.28687733	0.0064891
224	0.15522425	0.047344
225	0.88292198	0.2622174
226	0.21874913	0.1151278
227	0.41562375	0.0647895
228	0.0229516	0.0562507
229	0.12093533	0.0221542
230	0.023939	0.0163635
231	0.1012655	0.0915016
232	0.09232607	0.1008636
233	0.03033752	0.1169687
234	0.20809775	0.0632309
235	0.42832054	0.2246215
236	0.50265527	0.487581
237	0.33018871	0.1366192

238	-0.1622834	0.2500907
239	-1.181731	3.8924776
240	0.35550265	0.3311994
241	2.46719528	1.6614566
242	1.05018914	0.5049728
243	0.4669674	0.1874097
244	0.23666659	0.2354847
245	0.0326368	0.0343071
246	0.45091441	0.4090765
247	0.07241225	0.0374166
248	0.25672409	0.2493956
249	0.02289835	0.0172497
250	0.2888732	0.1773421
251	0.1260554	0.0389845
252	0.022286	0.0213631
253	0.04957643	0.110891
254	0.12171375	0.0817869
255	0.645839	0.260408
256	0.7274818	0.1736791
257	0.169798	0.0627554
258	0.0229535	0.0248911
259	0.01378125	0.0308523
260	0.174738	0.1414041
261	0.10259574	0.085534
262	-0.0759744	0.2943837
263	0.03937778	0.0548755
264	0.0751136	0.085315
265	0.070587	0.1050221
266	-0.0637867	0.0415553
267	-0.599157	0.4124092
268	-0.0011043	0.0291628
269	0.0739858	0.0659272
270	0.0247195	0.0138245
271	0.20785	0.1709468
272	0.3689089	0.1497574
273	0.33540423	0.578846
274	-0.4751884	0.4199997
275	0.102484	0.072857
276	0.03378809	0.378804
277	0.1533836	0.1633791
278	0.102359	0.2377077
279	0.25197866	0.0326197
280	0.13507943	0.2680571
281	0.26435331	0.1943795
282	0.05403599	0.28712
283	-0.1094488	0.2653851
284	-0.1229577	0.3924965
285	0.174649	0.1772448
286	0.1729572	0.126237
287	0.01722211	0.0926468
288	0.42003265	0.2412708
289	0.64406894	0.4324075

290	0.39324253	0.3969969
291	0.8267218	0.9785693
292	1.425379	0.6107092
293	0.1738206	0.1591653
294	0.1928295	0.0434647
295	0.80027901	1.219631
296	1.0350088	0.5581173
297	0.350417	0.1015572
298	0.2355021	0.398768
299	0.28358434	0.5635031
300	0.1646855	0.2423154
301	0.32057267	0.0361159
302	0.13433123	0.1121614
303	0.35952687	0.2548188
304	1.32682929	1.2071837
305	1.5763555	0.4551983
306	0.252878	0.1052885
307	0.529901	0.243523
308	0.0162846	0.3545566
309	1.00081165	1.1269767
310	0.8272058	0.3139811
311	0.5955336	0.3684239
312	0.003187	0.0280147
313	-0.2269469	0.3917759
314	-0.0464586	0.1306972
315	-0.1343562	0.3438002
316	0.11618907	0.2286434
317	0.06938733	0.1347642
318	0.17715635	0.1695669
319	1.219697	0.3459055
320	1.0541592	0.6247138
321	0.00283002	0.0369723
322	0.03673813	0.0318425
323	0.12080181	0.0263169
324	0.08408315	0.0576797
325	0.11163698	0.0057704
326	0.5954475	0.8122442
327	0.39705568	0.1527298
328	0.13648903	0.1316066
329	0.14343016	0.0955126
330	0.10240569	0.12949
331	0.13115237	0.1051391
332	0.44903019	0.2011397
333	0.37168023	0.2317546
334	0.79979083	0.239266
335	0.70418845	0.5113102
336	0.01595701	0.0423793
337	0.24832443	0.3195986
338	0.70614663	0.2585577
339	0.13043392	0.1833394
340	0.23100267	0.052069
341	0.12749495	0.0760722

342	-0.0985004	0.1146532
343	0.10799365	0.0276211
344	0.60247423	0.1106517
345	0.18292148	0.0298376
346	0.34742383	0.16139
347	1.11752066	0.5731723
348	-0.0255623	0.0701962

nt number	crosslink AP	crosslink Gag	t test Gag vs AP	crosslink NC	ttest NC vs AP
21	-7.1670294	-1.62089369	0.000235944		
22	-5.7864717	-0.15827313	0.006015984		
23	-7.8940574	0.43670795	0.004107723		
24	-11.538143	1.221639329	0.045666204		
25	-9.8448569	1.967455255	0.000526538	32.6998848	0.00758743
26	-6.472692	2.499901559	0.060105217	16.8883241	0.00150528
27	-4.3701665	1.328823823	0.140987995	7.38042291	0.00022569
28	-0.9966045	1.760760371	0.044444037	11.8546987	3.1521E-08
29	-0.1513601	1.672530904	0.054708589	0.99829988	0.04126549
30	0.3937017	1.485272653	0.195423828	1.46466385	0.56681803
31	-0.0012783	1.338799266	0.709140439	-0.2331804	0.54875548
32	-0.0244755	1.50006599	0.135069715	1.18296832	0.06673156
33	0.1859181	1.414610164	0.955938965	3.86818964	0.00187351
34	-0.2539904	1.400601183	0.070596979	1.0159993	0.05361173
35	-0.3201684	1.169705756	0.058425464	0.94410719	0.06472341
36	0.19634558	0.80908915	0.000515289	0.20585668	0.21072688
37	-0.07974	0.374132146	0.931720361	-0.5035812	0.10476229
38	-0.2451726	0.885842459	0.30538391	0.43096766	0.02026286
39	-0.2886421	0.432741926	0.068626253	-0.7264679	0.0360045
40	-0.6106317	0.360141718	0.13788204	-0.4062835	0.80178417
41	-0.4132883	0.34414077	0.831785884	0.25714885	0.2433503
42	-0.3231594	0.320635918	0.855911569	-0.0448839	0.49950882
43	-0.1041837	0.301286292	0.574818645	0.43288316	0.08611583
44	-0.0404294	0.334317728	0.151619043	0.99976661	0.01799729
45	0.18549448	0.217111717	0.424061741	0.45846989	0.1406784
46	-0.0391457	0.17773396	0.554842388	0.58367347	0.87279372
47	-0.2913015	0.133962704	0.015243232	0.06013754	0.20879222
48	0.12495651	0.131149919	0.395326198	0.98607007	0.05474802
49	0.17523529	0.167577734	0.925495924	0.7729323	0.44032888
50	0.57794555	0.13332531	0.034606776	0.34262993	0.95111905
51	0.34703641	0.16421251	0.185769816	0.02977958	0.02365492
52	0.42559292	0.270259672	0.037590823	1.91195471	0.00100908
53	0.49438269	0.303823233	0.017637492	-0.5586544	0.00112845
54	-0.1774473	0.285444897	0.539396437	1.64857538	0.01316357
55	-1.2666852	0.196043475	0.197257888	0.024188	0.78082204
56	-1.3193727	0.317924212	0.310631416	0.10140238	0.0142006
57	-0.4237631	0.270701187	0.918305727	-0.4418562	0.17720203
58	-0.8564647	0.221441906	0.58185319	-0.258436	0.7831602
59	-0.6723771	0.12227155	0.759495097	0.29923819	0.0149142

60	-0.0985154	0.055115247	0.293541493	0.54735781	0.09109819
61	0.11875491	0.10302036	0.910948042	1.42440976	0.00153677
62	-0.2715693	0.174085827	0.183423632	2.90597462	0.00593268
63	0.14573513	0.076013612	0.065427402	1.039619	0.00885481
64	1.26403337	0.059207778	0.041756606	4.58241823	1.8562E-05
65	3.62094932	0.172918565	0.217668271	2.75827482	0.2545435
66	3.30462986	0.191040373	0.01144043	5.27382198	0.92560337
67	0.08261637	0.039734072	0.109952151	-5.0150763	0.00047031
68	0.38718272	0.01387947	0.297807491	1.41580441	0.00314837
69	0.56087849	0.038423738	0.058713045	0.46131808	0.08896091
70	-0.1189999	0.033300322	0.308275847	0.17385888	0.27880081
71	0.40439686	0.030421739	0.062837962	0.31786037	0.67569335
72	2.64022227	0.012678495	0.015727281	1.10739086	0.27818052
73	1.69416512	-0.21496307	0.034605241	-1.240284	0.03666952
74	-0.3427716	-0.23279792	0.103072349	0.10580372	0.01047377
75	1.24884066	-0.13678283	0.367097395	2.65746027	0.13120457
76	0.64722475	-0.18121084	0.313531935	1.307163	0.92011347
77	-1.1819081	-0.10989891	0.054244689	2.05409408	0.00455731
78	-1.0329794	-0.03164285	0.007198929	2.82430476	0.00022969
79	0.29236832	0.017865322	0.254182233	2.40515487	0.00587018
80	0.52804024	0.02447224	0.134650246	3.90977629	0.00060378
81	0.0770122	0.090380175	0.284570602	0.92717864	0.14519167
82	-0.8786367	0.164158096	0.949464975	1.4008154	0.08547701
83	-0.8700373	0.270705481	0.281151184	0.65062786	0.4252096
84	0.01134725	0.403144051	0.87760645	0.75759094	0.41804177
85	-0.6108143	0.456034604	0.939036204	0.2157715	0.79369512
86	-0.9373137	0.340117121	0.52315434	1.42550402	0.00931441
87	-1.2860708	0.288945454	0.653447595	0.86156233	0.17372473
88	-1.1298364	0.233827226	0.408105513	1.27533469	0.15362151
89	-0.2201079	0.157434742	0.605824182	1.65236326	0.00051892
90	-0.0161406	0.105575799	0.248941906	0.89716094	0.00213206
91	0.07209586	0.102993709	0.917088988	1.14247944	0.04878708
92	0.35118231	0.136217892	0.890030804	0.1952845	0.88133079
93	0.31327859	0.125447546	0.485430486	0.73019446	0.44376951
94	-0.2318346	0.022424714	0.151392978	0.97071729	0.00385144
95	0.03140178	-0.06333555	0.418196132	1.29171614	0.01172143
96	3.73041111	-0.03725303	0.179142769	-0.2350553	0.0635032
97	3.86079976	-0.10530145	0.099784522	0.95050614	0.19678594
98	0.55615202	-0.10976296	0.013371311	4.40540941	0.00071691
99	0.77121152	-0.12122044	0.052564975	0.41652766	0.88762706
100	4.91910567	-0.08559716	0.038305993	-1.9607339	0.00038598

101	4.49667155	-0.08770919	0.09342295	-10.7351	0.0054287
102	0.21410326	-0.06664645	0.939573189	0.03457672	0.2421834
103	0.38353472	-0.08366757	0.803099319	0.67478853	0.15819246
104	0.2195831	0.00134627	0.167972029	0.9996922	0.36564336
105	-0.2544248	0.05214011	0.120880564	1.32060126	0.03362303
106	-0.0877812	0.180300591	0.359530971	1.38582645	0.12617058
107	-0.2561151	0.215714296	0.008572114	-1.7376537	0.006021
108	-0.3803557	0.340749894	0.357181858	2.4971103	0.00809555
109	0.17738029	0.348166475	0.88685743	0.14271969	0.31723597
110	0.7154271	0.308121439	0.086495801	-0.1051189	0.21375041
111	0.5467201	0.282962773	0.250585703	0.84304265	0.76642002
112	0.15048892	0.27555957	0.117552386	2.29284206	0.00084128
113	-0.1001947	0.231255308	0.696669193	0.59920466	0.09198464
114	0.00675535	0.195120028	0.634291004	0.1788222	0.26686935
115	0.26661413	0.07781746	0.122733613	0.63328403	0.19034607
116	0.10342596	0.002918623	0.053093268	1.11619269	0.07321863
117	0.04435842	-0.08954931	0.31999627	1.32717629	0.01223668
118	-0.2882408	-0.13828023	0.673540989	1.70281802	0.02373412
119	-0.7254965	-0.18231505	0.946105008	3.52525674	0.0421394
120	-0.3713073	-0.21661242	0.558787064	-0.5939143	0.88947278
121	-0.2310621	-0.11022695	0.496394672	3.73009181	0.00405248
122	-0.2845392	-0.13261479	0.792067524	0.85253318	0.01306929
123	-0.0525761	-0.08694741	0.255369475	0.83003277	0.03601092
124	0.2106401	-0.0702572	0.885916143	2.96416481	6.6956E-05
125	0.47871858	0.012121292	0.517946048	1.31797778	0.00110968
126	0.34622127	0.071814647	0.953629217	0.94708189	0.01337565
127	0.24987878	0.113348203	0.210080836	0.44670287	0.20655138
128	0.36325869	0.19642291	0.471433634	1.2931654	0.0871903
129	0.17329232	0.197654145	0.405902696	1.16575386	0.06296567
130	0.43729577	0.265584073	0.041283165	1.98335542	0.00010225
131	2.77837792	0.361693984	0.596026576	-0.3898285	0.02738536
132	2.70380317	0.389714315	0.160107437	-1.577447	0.03398288
133	0.77223969	0.42537224	0.043168063	2.5420877	0.00227263
134	0.70473047	0.442600504	0.078076107	1.84966573	0.00884188
135	0.70623425	0.435978966	0.309777702	-0.9851565	0.01035008
136	0.29982118	0.330057881	0.682589933	-4.8958939	0.01457183
137	-0.2295892	0.386346611	0.114398361	0.82523896	0.00542008
138	-0.5968403	0.418003169	0.09414604	0.77793876	0.00566157
139	-0.4404824	0.347925204	0.17460059	-0.1013669	0.14801678
140	0.12605789	0.336167342	0.475354186	0.18008112	0.2530447
141	-0.1716508	0.307695814	0.314756695	0.77989012	0.02592679

142	-0.3524928	0.277552725	0.185631277	1.83530387	0.00370481
143	-0.1944452	0.243857839	0.402535323	1.04536059	0.07605226
144	-0.0373451	0.285491309	0.375990948	-0.7811047	0.24027455
145	-0.0736998	0.25418371	0.867876718	0.65251359	0.09051101
146	2.33912547	0.255322894	0.267903558	-2.3164585	0.00688008
147	3.86002418	0.275793246	0.059019926	-1.8386252	0.01898982
148	-4.903693	0.368700371	0.321609014	-16.433816	2.8479E-07
149	-2.3232867	0.456600224	9.73445E-06	0.44837431	0.00028071
150	0.53431856	2.298130885	0.577981253	-42.346246	0.01634576
151	-5.5417939	2.610657902	0.54309122	-1.2768837	0.9828115
152	-2.7233784	2.496658264	0.654235947	6.69350997	0.04851011
153	-0.9079027	2.439337538	0.709723383	5.14385673	0.4342047
154	0.42556394	2.519124288	0.960047039	5.32334867	0.3596001
155	-0.0968441	2.56045937	0.60287392	-2.2715734	0.16953689
156	-0.463481	2.571832514	0.858824953	2.29287073	0.93370942
157	0.41302711	2.61094802	0.781106614	-0.1717435	0.71863741
158	-0.059544	2.676698271	0.66192462	1.91728883	0.09215803
159	0.68924548	2.472265784	0.430474819	0.61309682	0.07183323
160	4.12053583	2.275066424	0.199176914	-1.9273045	0.07047453
161	3.54990464	0.345752561	0.279533222	-1.2380847	0.28264408
162	0.4062019	0.061222703	0.779237989	2.03419639	0.00110149
163	0.27986662	0.170347567	0.923403308	1.5834717	0.00105096
164	-0.1528111	0.219846551	0.983369995	0.47537242	0.26703407
165	-0.7777013	0.091737098	0.631186646	2.40980248	0.00332471
166	-0.8400792	-0.00531621	0.519235289	4.66384625	0.00121777
167	-0.3692911	0.033715726	0.068810052	5.32104524	0.00035371
168	-0.2179319	0.073237403	0.133673388	-0.6426198	0.15429306
169	-0.6370051	0.03587637	0.79865532	3.31892814	0.02586334
170	-0.6432487	0.039985746	0.07668539	-0.7797381	0.87706609
171	-0.2702837	0.105050508	0.982842493	0.98585614	0.04540936
172	0.160499	0.2066729	0.594647106	0.59040295	0.10178459
173	0.32974478	0.182332405	0.154529499	0.85436374	0.75623856
174	0.04016689	0.124729346	0.223791495	0.53397017	0.91531145
175	0.1086955	0.120639049	0.636897054	1.23112965	0.42740507
176	2.38157427	0.097054874	0.167772849	-0.0344439	0.34846953
177	2.47249126	0.04452876	0.212826131	-2.1602182	0.13031287
178	5.93382896	0.019606611	0.687908383	-0.9825267	0.00467399
179	36.7307697	-0.28317599	0.000398274	-1.708996	0.0011693
180	33.0813883	-1.02557884	0.02992255	83.5124475	0.17424977
181	2.93798748	-1.2114032	0.016969945	12.016873	0.01044904
182	0.80942295	-1.21640067	0.020588908	2.74328169	0.01098459

183	-0.5368895	-1.19289997	0.473322493	0.77384679	0.22002501
184	-0.5232294	-1.22013262	0.180736434	1.44713142	0.00330534
185	-0.2963929	-1.16458518	0.423271813	4.23009262	0.00070418
186	-0.6607777	-1.14672283	0.012170313	1.16644	0.00897697
187	-0.4714922	-1.08462763	0.077563903	-2.0366581	0.0473112
188	0.18964956	-0.96160555	0.486650686	-1.9592814	0.12614452
189	0.02646973	-0.91493903	0.848830723	3.37261707	0.39840163
190	-0.2048932	-0.72781161	0.907224531	2.89166686	0.00243358
191	0.09262723	0.001399706	0.701156325	1.45045787	0.05030018
192	2.1094262	0.173924207	0.018328679	0.45651752	0.06298593
193	2.88174528	0.102665541	0.074969	3.6692535	0.65742777
194	0.6112296	-0.01098822	0.237502871	2.11272719	0.33795606
195	-0.1895752	-0.01211779	0.428652455	-0.1498585	0.39426273
196	0.11498273	-0.05207412	0.755735586	0.28115789	0.34031037
197	-0.4192623	-0.14935359	0.234620002	4.35114778	0.03585626
198	-0.5295645	-0.25578654	0.455946449	0.68554117	0.0069079
199	-0.2106308	-0.36200302	0.186138397	0.92809367	0.17627611
200	0.23868177	-0.46403387	0.842210579	1.51120411	0.01405546
201	1.184277	-0.31525348	0.908324516	4.01423531	0.10072036
202	0.55491772	-0.2091255	0.626523634	4.28647056	0.40993027
203	-0.1613696	-0.17749845	0.823403014	1.54682973	0.09857243
204	0.10687034	-0.0925292	0.64136502	0.35042251	0.73292678
205	0.00085996	-0.01697903	0.619192401	1.29762103	0.07674124
206	0.52206222	0.012238399	0.817503938	0.25502307	0.60471566
207	3.54367815	0.063727658	0.067170774	2.14413132	0.01161664
208	3.11626081	0.152168674	0.211763941	4.92079775	0.75200429
209	0.15441398	0.233731311	0.883338803	0.7276524	0.02019575
210	-0.1864893	0.256114082	0.541778171	1.55871899	0.00880736
211	-0.2527563	0.267546209	0.385565984	0.29114223	0.03371092
212	-0.0528511	0.167849504	0.418698074	-0.1245721	0.65274333
213	-0.2760131	0.080945187	0.759555306	1.75973907	0.00366333
214	-0.7037317	0.166856518	0.008498423	2.71464738	0.02004396
215	-0.6368933	0.170059247	0.068038728	0.35337199	0.02709396
216	-0.4451437	0.155902251	0.379296657	0.05296763	0.11884668
217	-0.3931866	0.171639476	0.073644501	0.31321505	0.08337169
218	0.68315114	0.166626383	0.022642186	0.55498636	0.00754574
219	8.29019011	0.160186626	0.088941148	0.09358319	0.07340181
220	7.81728237	0.118839599	0.147107917	-1.8106328	0.11219373
221	0.52060119	0.143444894	0.084422835	1.44329182	0.06017137
222	0.03744168	0.204296863	0.598994787	1.16241213	0.04145651
223	0.307695	0.182886264	0.693748082	0.18717383	0.35864615

224	0.16339088	0.201771862	0.335727596	2.87132805	0.01487324
225	-0.0978161	0.16906123	0.052612999	0.55072345	0.05432837
226	0.05031873	0.121186667	0.060099602	-0.1002777	0.30483115
227	-0.1159206	0.142262354	0.454780613	0.25245092	0.25922094
228	-0.1261522	0.100421064	0.364865748	1.11348273	0.00020159
229	-0.1466518	0.056967775	0.824550332	0.42816676	0.10516545
230	0.028177	0.032228988	0.593287024	0.06669325	0.98446301
231	0.63096247	0.076755963	0.990805068	0.61031909	0.51542521
232	0.23612913	0.114772802	0.188291389	3.19087353	0.00282087
233	-0.1480292	0.051108337	0.21738769	-0.3110343	0.37394838
234	-0.2285068	0.063851744	0.778717151	0.36553478	0.82373527
235	-0.3663978	0.064217786	0.009719602	0.39252127	0.01406653
236	0.86886296	0.048209509	0.535242468	1.63006589	0.01468418
237	3.55985216	0.160999643	0.252920438	3.06239253	0.00741826
238	44.4992168	0.141327797	0.006780678	6.35221597	0.28920873
239	41.7074315	0.096375891	0.048959088	79.5205913	0.75230308
240	-0.3469763	-0.03459636	0.102692692	3.67568914	0.00012183
241	-0.1034373	-0.1151524	0.313630709	2.90554023	0.00031594
242	-0.6726811	-0.01510359	0.032918961	6.60331499	6.812E-06
243	-1.739841	-0.19065992	0.321930739	-0.9321302	0.00252703
244	-1.3779928	-0.32494389	0.808878187	-0.1342049	5.2749E-05
245	0.16168933	-0.41423176	0.611073571	-0.7967684	0.51616158
246	-0.4235137	-0.52932273	0.088709652	0.69138422	0.86274437
247	-1.0958796	-0.65771708	0.271456951	-0.4974989	0.05761601
248	-0.1679531	-0.7268847	0.841874413	0.65953098	0.04862281
249	-0.6863711	-0.73748823	0.720480687	-0.0663796	0.09223813
250	-0.29789	-0.71699059	0.640944039	0.66807665	0.07439103
251	-0.3654865	-0.63830278	0.372890455	0.9673556	0.67109862
252	-0.4923443	-0.61344782	0.028273827	-1.4523962	0.17840524
253	0.0658773	-0.72502118	0.381226081	0.92453617	0.03730959
254	-0.1127374	-0.59541423	0.946579678	-0.5760923	0.26725345
255	-0.426149	-0.45689273	0.987597283	0.88805726	0.00752737
256	-0.3635811	-0.39550859	0.351250163	2.27453619	0.00101821
257	-0.0898691	-0.29221708	0.664843945	1.02489415	0.03078293
258	0.27159106	-0.19584372	0.388174192	0.2333572	0.08202226
259	0.57445875	-0.20860288	0.329886851	-1.8860444	0.00994804
260	0.21422248	-0.13734909	0.48427324	1.99083929	0.00241464
261	0.9562952	-0.13249046	0.534860731	-3.1074844	0.00016967
262	8.2108839	-0.06466346	0.150864875	-1.088927	0.04164029
263	7.50887997	-0.08721696	0.222068503	-1.9734492	0.19294362
264	0.74541044	-0.08141071	0.453554102	0.7267838	0.56327752

265	0.10688794	-0.06652268	0.346586472	-0.0815035	0.20372667
266	1.1120154	-0.04155947	0.127552091	0.56707129	0.02417833
267	11.4687496	-0.01612829	0.239171843	1.33414901	0.5830849
268	9.89266157	-0.18399807	0.145696622	2.86610676	0.25069181
269	-0.4292839	-0.2532281	0.135936706	2.36040557	0.00023475
270	-0.4429058	-0.27728953	0.349712059	-0.5148745	0.74663804
271	-0.0942325	-0.31323334	0.203104387	0.06610254	0.29762562
272	-0.173767	-0.32538853	0.18586079	0.56716846	0.06254717
273	1.66531046	-0.27973186	0.236097224	2.92973048	0.00482223
274	7.8238196	-0.23255877	0.099509552	1.93942831	0.48972687
275	6.08124176	-0.49622648	0.137327858	9.85523349	0.92397153
276	0.0762253	-0.51371405	0.577737997	1.84199416	0.00013621
277	-0.420885	-0.56510166	0.097646304	-0.6174182	0.05056715
278	-0.608602	-0.56442668	0.126811873	-0.8645607	0.56221623
279	-0.4420039	-0.42381091	0.13319105	-0.5376593	0.82201746
280	-0.0699298	-0.35910387	0.109069316	0.7203471	0.0466045
281	1.55328797	-0.33673545	0.39329527	10.9314605	0.00078343
282	1.19303204	-0.42739011	0.045301825	-2.1994015	0.01211763
283	0.0673593	-0.36541363	0.452980524	0.91864908	0.11225407
284	1.36069281	-0.3956599	0.537141844	1.46469465	0.04402084
285	0.70317396	-0.39544306	0.161807978	2.64200414	0.52698682
286	-0.5042305	-0.11378847	0.000495284	1.27327395	9.2521E-06
287	0.16995112	-0.10637761	0.181014908	-0.2548885	0.82950512
288	0.15468008	-0.05426159	0.259728004	1.26148239	0.05650136
289	-0.7225852	-0.05800584	0.308968424	-0.5420294	0.96243056
290	-0.5558224	-0.04901645	0.014545078	-0.773598	0.5382592
291	0.25628569	-0.10420559	0.073673431	1.73819088	0.00060905
292	0.0794255	-0.02208473	0.696750464	2.09244817	0.00145561
293	-0.4354634	0.157417065	0.018608097	3.80294495	0.00031244
294	0.02660487	0.134135694	0.338654596	0.15943017	0.18954482
295	2.45462201	0.156337681	0.940993463	0.57299851	0.50455674
296	2.2436078	0.246564524	0.589698346	0.44055024	0.58117591
297	0.38281833	0.254106432	0.639650597	2.37880382	3.5799E-05
298	0.36652547	0.246021375	0.626784709	0.16985045	0.94904731
299	1.58458321	0.259077456	0.55612723	0.59336694	0.1017717
300	4.53899212	0.230967641	0.166361031	1.39121575	0.56387986
301	2.71095895	0.117481396	0.195816063	3.57842153	0.85545364
302	-0.7994368	0.110751016	0.553944826	0.50771512	0.00096024
303	-0.8349578	0.028578698	0.003275348	0.50087058	0.00103793
304	-0.5634691	-0.07794692	0.272224591	1.44002688	0.00640339
305	-0.7380253	-0.057313	0.377577372	2.21399619	0.05599769

306	-1.0507499	-0.06621936	0.195742807	3.36854194	0.05111039
307	-2.3672964	-0.07712928	0.510607629	5.27458293	0.04497648
308	-3.6120297	-0.17635739	0.424952966	7.15477676	0.02559526
309	-3.3671997	-0.22353444	0.406013015	0.36525857	0.37572236
310	-1.7534593	-0.34376122	0.053879075	4.67230234	0.0513666
311	-0.1256134	-0.30591199	0.477399412	0.2382897	0.51438533
312	0.09059695	-0.21771722	0.4975155	0.63531734	0.11193512
313	1.84038632	-0.14297756	0.455998755	-0.060007	0.80476144
314	2.19954651	-0.13911926	0.852203059	-1.719597	0.54673997
315	0.53518354	-0.09310047	0.238614019	0.8355323	0.99172787
316	0.51544869	-0.06564412	0.324235505	0.76002164	0.16187196
317	0.16506909	-0.10108569	0.539601445	-0.3571716	0.29750011
318	-0.1034868	-0.11828329	0.007180899	0.57837425	0.00589652
319	-1.1346479	0.032977719	0.177137318	2.68873165	0.00085963
320	-1.9560186	0.119218371	0.015329994	0.83256119	0.01790816
321	-0.9190015	0.032022176	0.164103655	-3.7312968	0.01019389
322	-0.1304681	0.112628219	0.21765352	0.86378406	0.32671279
323	0.13638804	0.185637655	0.189885978	0.55229908	0.26302874
324	-0.0294639	0.196159421	0.416903177	-0.5159675	0.71029704
325	0.49865262	0.271076554	0.005432279	-0.4509287	0.3428402
326	1.22834762	0.295658843	0.163146337	-0.0404825	0.09195309
327	0.54677521	0.290302209	0.529291637	-0.0570946	0.33600478
328	-0.2949365	0.272365328	0.492225647	1.88158953	0.0368268
329	0.05382201	0.263207233	0.914790195	0.03367408	0.96553373
330	-0.1437386	0.184286752	0.089352658	-2.4214927	0.00011607
331	-0.3379567	0.142387146	0.002985557	0.11379805	0.03628428
332	0.0226197	0.324572748	0.29716259	0.23145735	0.97137407
333	-0.044796	0.207713334	0.966945483	0.10355792	0.74765764
334	-0.4727495	0.157977406	0.547495488	-0.1896655	0.29992684
335	-0.8037399	0.135725589	0.01201032	0.6071482	0.02868659
336	-0.4640601	0.094714418	0.014699398	-0.6178138	0.86133065
337	-0.6334169	0.067002585	0.09177655	-0.1242012	0.83551517
338	0.88415515	-0.06007237	0.264574887	-0.6183893	0.54971239
339	0.00650942	0.177285265	0.893633266	8.34395199	0.0017066
340	-2.4248876	0.108777399	0.237087125	-0.7362488	0.29314548
341	-2.3070199	-0.0236017	0.060374914	-2.8784526	0.24852839
342	-1.9239874	-0.09926664	0.00573584	-2.5367225	0.79719614
343	-0.675962	-0.26994204	0.230406814	-4.8480217	0.00219161
344	2.38416887	-0.39161987	0.291963278	4.01123937	0.00474074
345	0.80288627	-0.53649623	0.341607155	-1.8121427	0.32223432
346	-3.2450542	-1.41553769	0.028986367	-6.0810778	0.03813285
